# Visible-Light Driven Selective C–N Bond Scission in *anti*-Bimane-Like Derivatives

**DOI:** 10.1021/acs.orglett.1c01376

**Published:** 2021-06-02

**Authors:** Nejc Petek, Helena Brodnik, Uroš Grošelj, Jurij Svete, Franc Požgan, Bogdan Štefane

**Affiliations:** Faculty of Chemistry and Chemical Technology, University of Ljubljana, Večna pot 113, 1000 Ljubljana, Slovenia

## Abstract

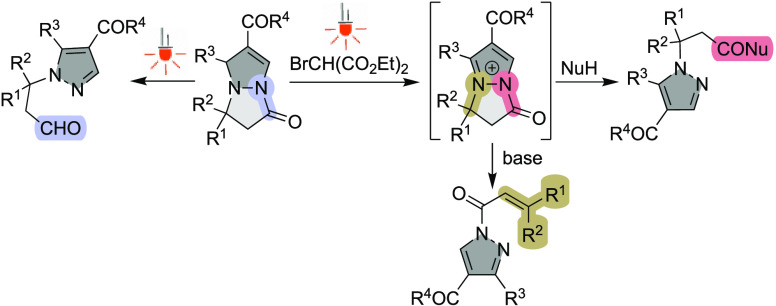

In the present study, we report the photochemical transformation of pyrazolo[1,2-*a*]pyrazolone substrates that reach an excited state upon irradiation with visible light to initiate the homolytic C–N bond cleavage process that yields the corresponding N1-substituted pyrazoles. Moreover, chemoselective heterolytic C–N bond cleavage is possible in the pyrazolo[1,2-*a*]pyrazole core in the presence of bromomalonate.

For organic substrates that do not absorb visible light, it is difficult to use direct energy transfer applying visible light to achieve transformation.^1^ In most cases, due to the poor visible light absorption of the reaction substrates, it is necessary to use photosensitizers to initiate the transformations.^2^ In the past decade, numerous efficient photocatalysts, including iridium, ruthenium, nickel, and copper complexes,^3^ as well as various organic dyes,^4^ have been investigated for visible light induced organic transformations to form carbon–carbon and carbon-heteroatom bonds under very mild reaction conditions. However, the high cost and sometimes complicated preparation of these photocatalysts limit their industrial application, especially for large-scale syntheses.^5^ A novel strategy for radical generation from catalytic visible-light-absorbing dithiocarbamates was recently presented by Melchiorre.^6^ The development of visible light-induced organic reactions using photoactive substrates without external photosensitizers or photocatalysts is considered a promising research direction, thus offering a cost-effective and more environmentally friendly approach to organic synthesis.^7^ Our group has explored the potential of some azomethine imines as 1,3-dipoles in [3 + 2]-annulation reactions.^8^ Recently, we have developed a visible-light-induced aerobic oxidation of N1-substituted pyrazolidin-3-ones to afford the corresponding azomethine imines, which can be further reacted in situ with ynones under copper-catalyzed [3 + 2] cycloaddition conditions to give the corresponding pyrazolo[1,2-*a*]pyrazoles.^9^*N*,*N*-Bicyclic pyrazolidin-3-ones, that is, pyrazolo[1,2-*a*]pyrazol-1-one derivatives exhibit pronounced bioactivity and have attracted much attention in drug development.^10^ Among them, pyrazole derivatives have been given special consideration in cancer therapy.^11^ Stoichiometric oxidation-ring opening (Br_2_, CAN, O_2_/Cu^2+^) of the corresponding pyrazolo[1,2-*a*]pyrazoles in the presence of water as a nucleophile (Scheme 1a) was previously explored by us and others and leads to N1-substituted pyrazoles.^12^ Since pyrazolo[1,2-*a*]pyrazoles absorb in the visible frequency range (up to 420 nm), we envisioned that visible-light induced transformations of the pyrazolo[1,2-*a*]pyrazole core could provide a mild and economical route to valuable N1-substituted pyrazole derivatives, as shown in Scheme 1b.

**Scheme 1:**
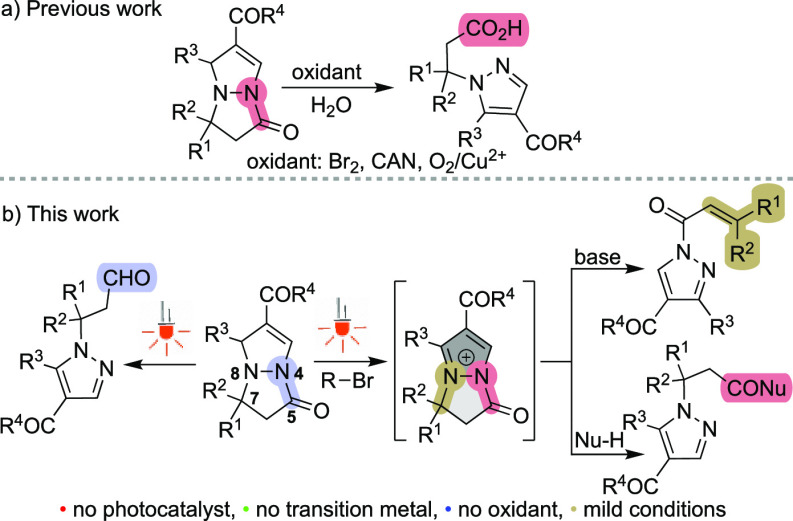
Ring Scission of Pyrazolo[1,2-*a*]pyrazoles

To investigate the feasibility of the proposed strategy, we chose **1a** (R^1^ = Me, R^2^ = 4-Cl-C_6_H_4_) with the absorption maxima at 360 nm (ε_max_ = 8400 M^–1^ cm^–1^, λ_em_ = 535 with Θ_f_ = 0.11)^13^ as a model substrate for visible-light-induced C–N bond cleavage in the pyrazolo[1,2-*a*]pyrazole core. Surprisingly, irradiation of **1a** with 400 nm 3 W LEDs for 24 h in DCM at 25 °C led to the formation of the corresponding aldehyde **2a** in 78% yield. Careful examination of different solvents (THF, EtOAc, MeCN, acetone, MeOH, and DMF) revealed that DCM performed best in this protocol. Moreover, a control experiment with longer wavelengths (450 nm LEDs) slowed the conversion. The reaction was additionally tested in dichloroethane at 50 °C for 12 and 24 h, resulting in lower yields of **2a** with notable side reactions (for details, see Table SI1). Next, various substituted pyrazolo[1,2-*a*]pyrazoles **1** were investigated to evaluate the generality of the transformation. Here, C1 phenyl-, heteroaryl-, and alkyl-substituted substrates afforded the corresponding aldehydes **2a**–**g** in moderate to good yields (Scheme 2). When vinyl-derived substituents were introduced onto the C1 position of the pyrazolo[1,2-*a*]pyrazole scaffold, the ring expansion products **3** were formed, together with the formation of products **2** (Scheme 2, examples **3h** and **3i**). The formation of ring-expansion products **3** commenced via C1**–**N8 homolytic bond cleavage followed by radical 7-endo-trig cyclization.^13^

**Scheme 2:**
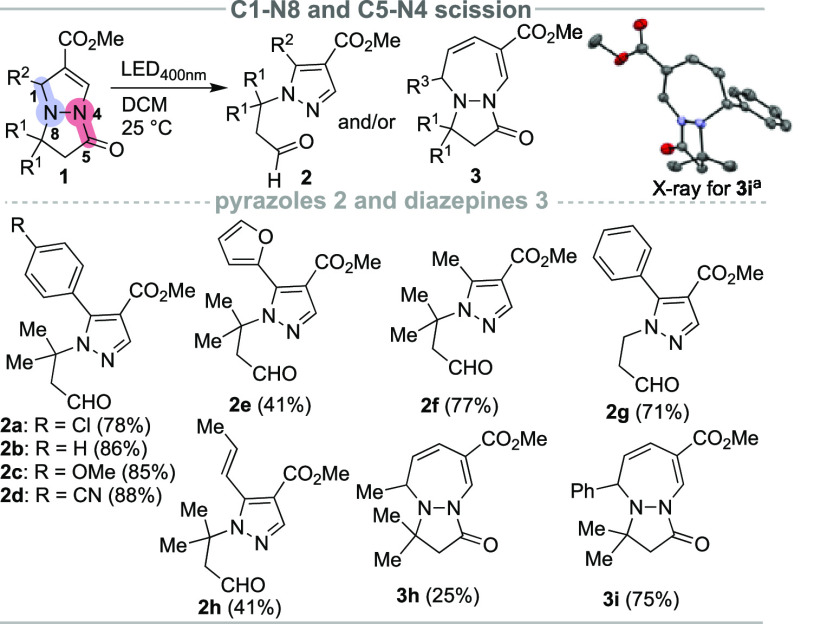
C1–N8 and C5–N4 Bond Cleavage of Pyrazolo[1,2-*a*]pyrazoles Hydrogens are omitted for clarity. **1** (0.5 mmol), DCM (2.5 mL), LED_400nm_, 25 °C, under N_2_ for 24–48 h.

Considering the ability of **1a** to act as a reducing agent in the excited state (*E*_ox_^*^ ∼ – 1.8 V vs SCE),^13^ the photoreduction of activated alkyl bromides, such as diethyl bromomalonate (*E*_red_ = −0.62 V vs SCE) or 2-bromoacetophenone (*E*_red_ = −1.46 V vs SCE), would be possible.^14^ For details and discussion on the optimization studies, see Supporting Information. Detailed screening of the reaction conditions revealed that **1a** could be converted to the N1-acryloyl-substituted pyrazole **4a** and isolated in 78% yield when irradiated with blue light (450 nm) in the presence of diethyl bromomalonate (2.0 equiv) and 2,6-lutidine (1.5 equiv) as the base in DCM. To explore the substrate scope, the above optimized reaction conditions were applied to a variety of substituted pyrazolo[1,2-*a*]pyrazoles **1** (Scheme 3). Bicycles **1** with electron-withdrawing groups on the benzene ring, such as chloro and cyano, and electron-donating substituents, such as methoxy and methyl, were well-tolerated in the present transformation and showed no obvious difference in reactivity, as the corresponding products **4a**–**g** were isolated in good yields. Moreover, the reaction result was not altered when a naphthalene unit (example **4j**) was introduced. Notably, it was also possible to extend the substrate range to carbonyl, aminocarbonyl, and carbamate substituents on both pyrazole rings in bicyclic substrates, resulting in good yields of the corresponding products **4k**, **4l**, and **4n**. Alkyl substitution was also tolerated as product **4i** was isolated in a 65% yield. Unfortunately, the styryl-substituted pyrazolo[1,2-*a*]pyrazole **1** gave product **4h** in a rather low 30% isolated yield. When the *N*-methylpyrrole substituted bicycle **1** was reacted under standard conditions, the corresponding *N*-acryloyl substituted pyrazole **4o** was isolated in a 15% yield, together with the C5′ malonyl substituted derivative **4o′** in a 50% yield. The coupling of this electron-deficient malonate radical at the C2 position with electron-rich arenes, such as pyrroles, thiophenes, and furans under visible light-mediated conditions was documented by Stephenson,^15^ Trapp,^16^ Noël,^17^ and Wu.^18^ To demonstrate the scalability of the method, a gram-scale experiment was performed to give **4a** with a comparable 80% product yield.^13^

**Scheme 3:**
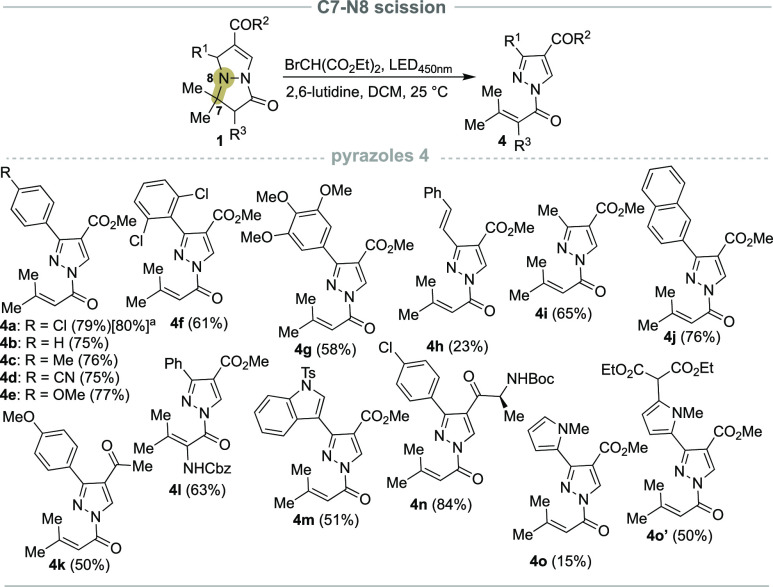
C7–N8 Bond Cleavage of Pyrazolo[1,2-*a*]pyrazoles Gram scale yield. **1** (0.5 mmol), diethyl bromomalonate (2.0 equiv), 2,6-lutidine (1.5 equiv), DCM (2.5 mL), N_2_, 18 h.

Optimization studies revealed that the presence of nucleophiles, such as water (Table SI2, entry 10)^13^ in the reaction mixture favored the formation of 5a as the major product. This suggests the possibility of regioselective C5–N4 photoinduced nucleophilic ring opening of pyrazolo[1,2-*a*]pyrazoles **1**. To explore the substrate and nucleophile range for these types of transformations, we investigated the reaction of **1a** with various nucleophiles (Scheme 4). In the presence of diethyl bromomalonate (2.0 equiv) in DCM under LED_450nm_ irradiation for 19 h at 25 °C, followed by the addition of water (10 equiv), substrate **1a** was successfully transformed to the corresponding acid **5a** in an excellent 97% yield after isolation (Scheme 4). It is worth noting that in this case no additional base was required. In addition to water (Scheme 4, examples **5a**, **5g**, and **5i**), other nucleophiles were also introduced. The reaction proved to be equally successful in the presence of methanol and *p*-cresol, obtaining the corresponding esters **5b**, **5c**, **5h**, **5j**, and **5k** in good to excellent yields. Aliphatic amines and anilines were also tolerated in this protocol, giving the desired amides **5d** and **5e** in reasonable 64% and 63% yields, respectively. Moreover, l-alanine methyl ester was successfully coupled under the developed protocol, obtaining product **5f** in a 60% yield. In addition, the reaction result was not significantly altered by the substitution pattern on the pyrazolo[1,2-*a*]pyrazole core, as exemplified by products **5g**–**k**. Interestingly, when bicycles **1** were reacted in THF as the chosen solvent, the corresponding terminal halohydrin esters **5l** and **5m** formed in reasonable yields as a result of tetrahydrofuran ring opening induced by the pyrazolium intermediate **I2** (Scheme 5).

**Scheme 4:**
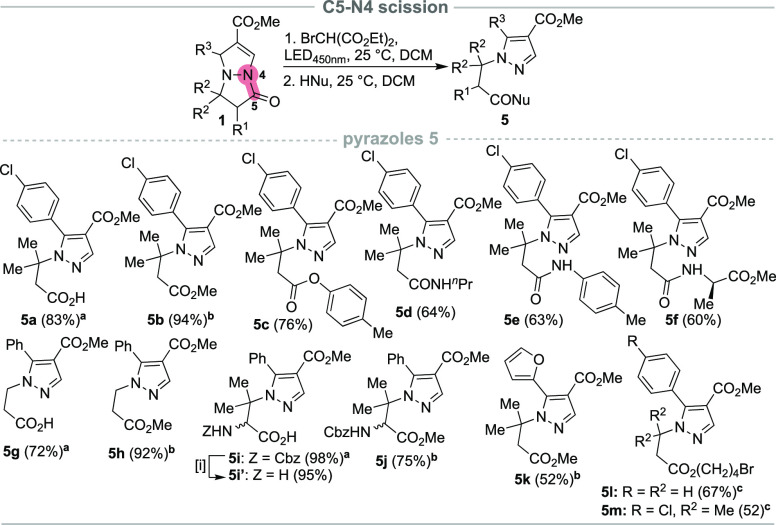
C5–N4 Bond Cleavage of Pyrazolo[1,2-*a*]pyrazoles Degassed Me_2_CO (H_2_O, 10 equiv). MeOH (anhydrous, degassed, 2.5 mL). THF (anhydrous, degassed, 2.5 mL). [i] H_2_ (2 bar), 10% Pd(C), 6 h. **1** (0.5 mmol), diethyl bromomalonate (2.0 equiv), DCM (anhydrous, degassed, 2.5 mL), N_2,_ 18 h, HNu (2.0 equiv).

**Scheme 5:**
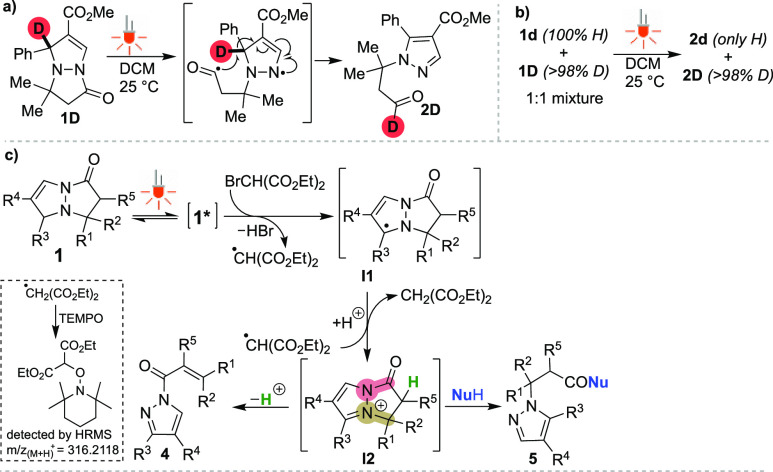
Mechanistic Insight

To gain more insight into the photoinduced transformation of pyrazolo[1,2-*a*]pyrazoles **1**, several control experiments were conducted. First, an experiment was performed with on/off irradiation with visible light. As shown in Figure SI2,^13^ continuous irradiation with visible light is essential for successful transformation. A reaction with deuterated solvent (CD_2_Cl_2_) was also performed in which no deuterated aldehyde **2D** was detected. However, when the C1-deuterated substrate **1D** (Scheme 5a) was reacted under the standard reaction conditions, only deuterated aldehyde **2D** was obtained. Furthermore, a crossover experiment with equimolar mixture of **1d** (100% H on C1) and **1D** (>98% D on C1) under the standard reaction conditions resulted in no crossover product being detected in the crude reaction mixture (Scheme 5b). To clarify from which excited state of substrates **1** do the aldehyde products **2** originate from, the reaction of **1a** was caried out in the presence of a triplet-annihilator, *trans*-stilbene (*E*_T_ = 49.3 kcal/mol). It is noteworthy that 1 equiv of *trans*-stilbene inhibits the reaction (Figure SI1),^13^ and a significant amount of *cis*-stilbene (70%) is produced during the reaction. The above experiments are consistent with visible light excitation of bicycle **1** to the S^1^ excited state, followed by ISC to the T^1^ excited state, which derives the corresponding biradical intermediate after C5–N4 bond cleavage. Subsequently, 1,5-hydrogen shift and aromatization gives the desired products, pyrazoles **2** (Scheme 5a). In the case of C1-vinyl derived substrates **1**, homolytic C1–N8 bond cleavage becomes the competitive reaction process yielding diazepine products **3** upon 7-endo-trig cyclization (Scheme SI9).^13^ In addition, the reaction of **1a** with diethyl bromomalonate was also studied more in detail. The reaction of **1a** with diethyl bromomalonate was tested under standard reaction conditions in the presence of TEMPO as a radical scavenging reagent (Scheme 5c). The formation of the TEMPO-malonate product can be clearly identified by HRMS analysis (exact mass: 316.2118 [C_16_H_30_NO_5_]) in the crude reaction mixture.^13^ This result and the formation of diethyl malonate adduct **4o′** (Scheme 3) suggest that the malonyl radical is most likely involved in this transformation. The Stern–Volmer plot suggests that the interaction between the excited pyrazolo[1,2-*a*]pyrazoles **1** and diethyl bromomalonate (Figure SI9)^13^ exists. Following the reaction progress by ^1^H NMR shows the formation of intermediate **I2** (Figure SI3),^13^ which upon the addition of water converts to a corresponding carboxylic acid **5** or into product **4** upon addition of 2,6-lutidine as the base. On the basis of these experiments and related literature precedents,^15−18^ a possible reaction mechanism is shown in Scheme 5c. The pyrazolo[1,2-*a*]pyrazole **1** was first excited with visible light to form the excited 1*, which underwent single electron transfer (SET) with diethyl bromomalonate to generate the corresponding radical cation of **1**. The mesolytic loss of a bromide ion from the bromomalonate radical anion would then provide the malonate radical. Deprotonation of the radical cation intermediate of **1** yields the corresponding radical intermediate **I1**, which in turn reacts with the malonyl radical by SET to furnish the cationic intermediate **I2**. The pyrazolium intermediate **I2** can provide products **4** under basic reaction conditions or alternatively give products **5** in the presence of nucleophiles.

In summary, we have demonstrated a novel visible-light-induced transformation of substituted pyrazolo[1,2-*a*]pyrazoles **1**. Excitation leads to chemoselective C–N bond cleavage of the pyrazolo[1,2-*a*]pyrazole scaffold, resulting in densely substituted pyrazoles. The reaction outcome depends on the nature of the substrates and the reaction protocol, thus providing a versatile approach for functionalized pyrazole derivatives originating from readily available starting materials. Further investigations into applications of this methodology are currently ongoing.

## Supporting Information Available

Materials and methods, experimental procedures, mechanistic and optimization studies, characterization data, and ^1^H NMR and ^13^C NMR spectra (PDF)

## Accession Codes

CCDC 2077375–2077377 contain the supplementary crystallographic data for this paper. These data can be obtained free of charge via www.ccdc.cam.ac.uk/data_request/cif, or by emailing data_request@ccdc.cam.ac.uk, or by contacting The Cambridge Crystallographic Data Centre, 12 Union Road, Cambridge CB2 1EZ, UK; fax: +44 1223 336033.
