# Supplementary material for: Visible-Light Driven Selective C–N Bond Scission in anti-Bimane-Like Derivatives
Source: Org Lett. 2021 Jun 2;23(14):5294–8. doi: 10.1021/acs.orglett.1c01376 (PMC8832495; doi:10.1021/acs.orglett.1c01376)
Supplement: Supplementary file 1 — ol1c01376_si_001.pdf [file ol1c01376_si_001.pdf]

Supporting Information

Visible-Light Driven Selective C–N Bond Scission in *anti*-Bimane-Like Derivatives

Nejc Petek, Helena Brodnik, Uroš Grošelj, Jurij Svete, Franc Požgan, Bogdan Štefane*

Faculty of Chemistry and Chemical Technology, University of Ljubljana, Večna pot 113, 1000 Ljubljana, Slovenia

Table of contents

1. General Information 2
2. Preparation and characterization of azomethine imines (AMI) and compounds 1 3
3. Optimization studies for the synthesis of pyrazoles 2 7
4. General procedure for the synthesis of aldehydes 2 and diazepines 3 8
5. Characterization data of novel compounds 2 and 3 9
6. Optimization studies for the synthesis of pyrazoles 4 11
7. General procedure for the synthesis of pyrazoles 4 12
8. Characterization data of novel compounds 4 12
9. General procedure for the synthesis of pyrazoles 5 15
10. Characterization data of novel compounds 5 16
11. Gram-scale synthesis of 4a 19
12. Mechanistic studies 20
13. Absorption and emission measurements 24
14. Stern–Volmer quenching experiments 25
15. Cyclic Voltammograms 26
16. X-ray crystallography data 27
17. NMR spectra 29
18. References 84

1. General Information

Reactions were carried out in borosilicate vials in a commercially available SynLED Parallel Photoreactor (465–470 nm, 130–140 lm, Sigma-Aldrich, St. Louis, MO, USA), and a custom made photoreactor, cooled with a cooling block to sustain a reaction temperature of 25 °C (room temperature). Vials are placed approximately 2 mm above LEDs with no filter applied. LEDs used: 400 nm (Edison, LT-1467, UV, wavelength of peak intensity 395–410 nm, radiantflux 350mW), 450 nm (ProLight Opto, PM2B-3-LBS-SD, blue, wavelength of peak intensity 445–455 nm, 39.8–51.7 lm) and 510 nm (Seoul Semiconductor, G42180, green, wavelength of peak intensity 505–520 nm, 70 lm). Cyclic voltammograms were recorded on ElectraSyn 2.0 (IKA®-Werke GmbH & Co. KG, Staufen, Germany) on glassy carbon working electrode with Pt plated counter electrode and Ag wire quasi-reference electrode. Anhydrous solvents were used. The NMR spectra were recorded in deuterated solvents with Me_4Si as the internal standard on a Bruker Avance III UltraShield 500 plus instruments (Bruker, Billerica, MA, USA) at 500 MHz for ^1H and at 126 MHz for ^{13}C nuclei, respectively. Data for ^1H NMR are reported as chemical shifts (δ) in ppm, multiplicity (bs = broad singlet, s = singlet, d = doublet, t = triplet, q = quartet, m = multiplet), coupling constant and integration. Data for ^{13}C are reported as chemical shift (δ) in ppm. Mass spectra were recorded on Agilent 6224 Accurate Mass TOF LC/MS spectrometer (Agilent Technologies, Santa Clara, CA, USA) and IR spectra on a Bruker FTIR Alpha Platinum spectrophotometer (Bruker, Billerica, MA, USA). Melting points were determined on a Kofler hot-stage microscope. Absorption spectra were recorded on Cary 50 Bio UV-VIS Spectrophotometer (Agilent Technologies, Santa Clara, CA, USA). Photoluminescence spectra were recorded on Cary Eclipse Fluorescence Spectrophotometer (Agilent Technologies, Santa Clara, CA, USA). Thin-layer chromatography (TLC) was performed on aluminum backed silica plates (0.2 mm, 60 F254, Sigma-Aldrich, St. Louis, MO, USA). Visualization of TLC (254 nm, Camag, Muttenz, Switzerland) was performed by fluorescence quenching or with potassium permanganate stains. Column chromatography (CC) was performed on silica gel (particle size: 35–70 µm, Sigma-Aldrich, St. Louis, MO, USA). Commercially available compounds were used without further purification. Compounds **AMI** and **1** were prepared according to the established literature procedures as described herein.

2. Preparation and characterization of azomethine imines (AMI) and compounds 1

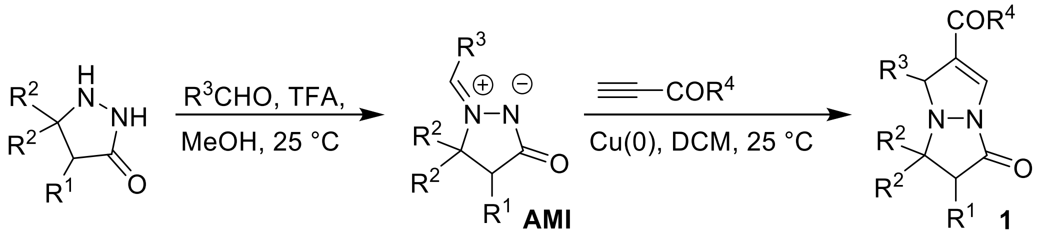

Scheme SI1: Reaction scheme for the preparation of azomethine imines and pyrazolo[1,2-*a*]pyrazoles **1**.

Compounds **AMI1-15** were prepared according to established literature procedures. ^{1}H NMR data of known compounds are in agreement with reported values.

Compounds **1a**–**r** were prepared according to the procedure, which has been slightly modified from the literature.[9] A mixture of **AMI** (2.0 mmol), dipolarophile (2.4 mmol), copper powder (80 mg), and CH_2Cl_2 (10 mL) was stirred at 25 °C for 24 h. The catalyst and impurities were removed by CC (DCM/MeOH, 40:1) to give pure products **1**.

(*Z*)-1-(4-Chlorobenzylidene)-5,5-dimethyl-3-oxopyrazolidin-1-ium-2-ide (**AMI1**).[1]

(*Z*)-1-Benzylidene-5,5-dimethyl-3-oxopyrazolidin-1-ium-2-ide (**AMI2**).[2]

(*Z*)-1-(4-Methoxybenzylidene)-5,5-dimethyl-3-oxopyrazolidin-1-ium-2-ide (**AMI3**).[1]

(*Z*)-1-(4-Cyanobenzylidene)-5,5-dimethyl-3-oxopyrazolidin-1-ium-2-ide (**AMI4**).[1]

(*Z*)-1-(Furan-2-ylmethylene)-5,5-dimethyl-3-oxopyrazolidin-1-ium-2-ide (**AMI5**).[3]

(*Z*)-1-Ethylidene-5,5-dimethyl-3-oxopyrazolidin-1-ium-2-ide (**AMI6**).[4]

(*Z*)-1-Benzylidene-3-oxopyrazolidin-1-ium-2-ide (**AMI7**).[5]

(*Z*)-3,3-Dimethyl-5-oxo-2-[(*E*)-3-phenylallylidene]pyrazolidin-2-ium-1-ide (**AMI8**).[6]

(*Z*)-5,5-Dimethyl-1-(4-methylbenzylidene)-3-oxopyrazolidin-1-ium-2-ide (**AMI9**).[1]

(*Z*)-1-(2,6-Dichlorobenzylidene)-5,5-dimethyl-3-oxopyrazolidin-1-ium-2-ide (**AMI10**).[7]

(*Z*)-5,5-Dimethyl-3-oxo-1-(3,4,5-trimethoxybenzylidene)pyrazolidin-1-ium-2-ide (**AMI11**). [7]

(*Z*)-5,5-Dimethyl-1-(naphthalen-2-ylmethylene)-3-oxopyrazolidin-1-ium-2-ide (**AMI12**).[6]

(*Z*)-1-Benzylidene-4-(benzyloxycarbonylamino)-5,5-dimethyl-3-oxopyrazolidin-1-ium-2-ide (**AMI13**).[8]

(*Z*)-5,5-dimethyl-3-oxo-1-((1-tosyl-1*H*-indol-3-yl)methylene)pyrazolidin-1-ium-2-ide (**AMI14**). Prepared according to an established literature procedure[7] from 5,5-dimethylpyrazolidin-3-one (570 mg, 5.0 mmol) and 1-tosyl-1*H*-indole-3-carbaldehyde (1.57 g, 5.25 mmol); 1.364 g (69% yield); light brown solid, mp 163–164 °C; ν_{max}/cm^{-1} (ATR)

1652, 1589, 1172, 1091, 667; δ_H (500 MHz; $CDCl_3$; Me_4Si) 9.22 (s, 1H), 8.05 (d, *J* = 8.3 Hz, 1H), 7.87 (d, *J* = 8.3 Hz, 2H), 7.68 (d, *J* = 7.8 Hz, 1H), 7.41 (t, *J* = 7.6 Hz, 1H), 7.35 (t, *J* = 7.5 Hz, 1H), 7.30 (s, 1H), 7.25 (d, *J* = 8.3 Hz, 2H), 2.81 (s, 2H), 2.34 (s, 3H), 1.75 (s, 6H); δ_C (126 MHz; $CDCl_3$; Me_4Si) 181.5, 145.7, 134.7, 134.3, 132.6, 130.1, 128.0, 127.2, 125.8, 124.0, 120.2, 117.9, 114.0, 111.7, 72.5, 45.2, 29.0, 21.6; HRMS (ESI) m/z: $[M + H]^+$ Calcd for $C_{21}H_{22}N_3O_3S$ 396.1376; Found 396.1369.

(*Z*)-5,5-dimethyl-1-((1-methyl-1*H*-pyrrol-2-yl)methylene)-3-oxopyrazolidin-1-ium-2-ide (**AMI15**). Prepared according to an established literature procedure[7] from 5,5-dimethylpyrazolidin-3-one (570 mg, 5.0 mmol) and 1-methylpyrrole-2-carboxaldehyde (573 mg, 5.25 mmol); additionally purified by CC (DCM/MeOH, 10:1); 749 mg (73% yield); light brown solid, mp 188–189 °C; ν_{max}/cm^{-1} (ATR) 3081, 2970, 1644, 1602, 1324, 1308, 1072, 1052, 682 ; δ_H (500 MHz; $CDCl_3$; Me_4Si) 7.87 (dd, *J* = 4.1, 1.5 Hz, 1H), 7.14 (s, 1H), 6.92 – 6.88 (m, 1H), 6.29 (dd, *J* = 4.0, 2.6 Hz, 1H), 3.82 (s, 3H), 2.69 (s, 2H), 1.68 (s, 6H); δ_C (126 MHz; $CDCl_3$; Me_4Si) 180.8, 129.8, 124.5, 121.88, 121.85, 119.4, 110.5, 71.4, 45.4, 34.5, 28.8; HRMS (ESI) m/z: $[M + H]^+$ Calcd for $C_{11}H_{16}N_3O$ 206.1288; Found 206.1289.

Methyl 1-(4-chlorophenyl)-7,7-dimethyl-5-oxo-6,7-dihydro-1*H*,5*H*-pyrazolo[1,2-*a*]pyrazole-2-carboxylate (**1a**). Prepared according to the general procedure from **AMI1**. ^1H NMR data is in agreement with the reported values.[9]

Methyl 7,7-dimethyl-5-oxo-1-phenyl-6,7-dihydro-1*H*,5*H*-pyrazolo[1,2-*a*]pyrazole-2-carboxylate (**1b**). Prepared according to the general procedure from **AMI2**. ^1H NMR data is in agreement with the reported values.[9]

Methyl 1-(4-methoxyphenyl)-7,7-dimethyl-5-oxo-6,7-dihydro-1*H*,5*H*-pyrazolo[1,2-*a*]pyrazole-2-carboxylate (**1c**). Prepared according to the general procedure from **AMI3** (464 mg, 2.0 mmol) and methyl propiolate (214 µL, 2.4 mmol); 485 mg (77% yield); yellow solid, mp 86–87 °C; ν_{max}/cm^{-1} (ATR) 3080, 1689, 1601, 1325, 1262, 1247, 1224, 1192, 1170; δ_H (500 MHz; $CDCl_3$; Me_4Si) 7.49 (d, *J* = 1.5 Hz, 1H), 7.38 – 7.34 (m, 2H), 6.90 – 6.85 (m, 2H), 5.43 (d, *J* = 1.2 Hz, 1H), 3.80 (s, 3H), 3.62 (s, 3H), 2.86 (d, *J* = 15.7 Hz, 1H), 2.39 (d, *J* = 15.7 Hz, 1H), 1.23 (s, 3H), 1.15 (s, 3H); δ_C (126 MHz; $CDCl_3$; Me_4Si) 166.4, 164.2, 159.2, 134.1, 129.2, 128.9, 117.0, 113.8, 64.3, 64.0, 55.2, 51.5, 49.5, 25.0, 19.0; HRMS (ESI) m/z: $[M + H]^+$ Calcd for $C_{17}H_{21}N_2O_4$ 317.1496; Found 317.1492.

Methyl 1-(4-cyanophenyl)-7,7-dimethyl-5-oxo-6,7-dihydro-1*H*,5*H*-pyrazolo[1,2-*a*]pyrazole-2-carboxylate (**1d**). Prepared according to the general procedure from **AMI4** (454 mg, 2.0 mmol) and methyl propiolate (214 µL, 2.4 mmol); 380 mg (61% yield); yellow solid, mp 104–105 °C; ν_{max}/cm^{-1} (ATR) 2229, 1740, 1684, 1600, 1327, 1197; δ_H (500 MHz; $CDCl_3$; Me_4Si) 7.69 – 7.59 (m, 4H), 7.52 (d, *J* = 1.4 Hz, 1H), 5.52 (d, *J* = 1.1 Hz, 1H), 3.64 (s, 3H), 2.88 (d, *J* = 15.8 Hz, 1H), 2.43 (d, *J* = 15.8 Hz, 1H), 1.24 (s, *J* = 18.0 Hz, 3H), 1.13 (s, *J* = 20.0 Hz, 3H); δ_C (126 MHz; $CDCl_3$; Me_4Si) 166.5, 163.8, 147.2, 132.3, 129.9, 128.7, 118.8, 115.7, 111.8, 64.6, 64.1, 51.7, 49.1, 24.9, 19.0; HRMS (ESI) m/z: $[M + H]^+$ Calcd for $C_{17}H_{18}N_3O_3$ 312.1343; Found 312.1337.

Methyl 1-(furan-2-yl)-7,7-dimethyl-5-oxo-6,7-dihydro-1*H*,5*H*-pyrazolo[1,2-*a*]pyrazole-2-carboxylate (**1e**). Prepared according to the general procedure from **AMI5** (384 mg, 2.0

mmol) and methyl propiolate (214 µL, 2.4 mmol); 418 mg (76% yield); yellow solid, mp 131–132 °C; ν_{max}/cm^{-1} (ATR) 3078, 1732, 1688, 1601, 1206, 748; δ_H (500 MHz; $CDCl_3$; Me_4Si) 7.57 (d, *J* = 1.5 Hz, 1H), 7.40 (d, *J* = 1.1 Hz, 1H), 6.35 (dd, *J* = 3.2, 1.9 Hz, 1H), 6.27 (d, *J* = 3.2 Hz, 1H), 5.58 (d, *J* = 1.2 Hz, 1H), 3.70 (s, 3H), 2.87 (d, *J* = 15.7 Hz, 1H), 2.39 (d, *J* = 15.7 Hz, 1H), 1.31 (s, 3H), 1.22 (s, 3H); δ_C (126 MHz; $CDCl_3$; Me_4Si) 166.8, 164.0, 153.5, 142.6, 131.1, 113.2, 110.6, 107.3, 64.6, 58.1, 51.7, 49.1, 24.7, 18.8; HRMS (ESI) m/z: $[M + H]^+$ Calcd for $C_{14}H_{17}N_2O_4$ 277.1183; Found 277.1182.

Methyl 1,7,7-trimethyl-5-oxo-6,7-dihydro-1*H*,5*H*-pyrazolo[1,2-*a*]pyrazole-2-carboxylate (**1f**). Prepared according to the general procedure from **AMI6**. ^{1}H NMR data is in agreement with the reported values.[3]

Methyl 5-oxo-1-phenyl-6,7-dihydro-1*H*,5*H*-pyrazolo[1,2-*a*]pyrazole-2-carboxylate (**1g**). Prepared according to the general procedure from **AMI7**. ^{1}H NMR data is in agreement with the reported values.[10]

Methyl (*E*)-7,7-dimethyl-5-oxo-1-(prop-1-en-1-yl)-6,7-dihydro-1*H*,5*H*-pyrazolo[1,2-*a*]pyrazole-2-carboxylate (**1h**). Prepared by stirring a solution of 5,5-dimethylpyrazolidin-3-one (570 mg, 5.0 mmol), crotonaldehyde (435 µL, 5.25 mmol) and TFA (10 µL) in MeOH (10 mL) at 25 °C for 6 h. After evaporating the volatiles, copper powder (200 mg), CH_2Cl_2 (25 mL) and methyl propiolate (535 µL, 6.0 mmol) were added and the mixture was stirred at 25 °C for 24 h. The catalyst and impurities were removed by CC on silica (DCM/MeOH, 40:1) and the filtrate evaporated in vacuo to give **1h** as an oil, which slowly crystalized. 850 mg (68% yield); yellow solid; mp 57–58 °C; ν_{max}/cm^{-1} (ATR) 2966, 1731, 1695, 1598; δ_H (500 MHz; $CDCl_3$; Me_4Si) 7.39 (d, *J* = 1.5 Hz, 1H), 5.78 (dqd, *J* = 13.0, 6.5, 0.7 Hz, 1H), 5.55 (ddq, *J* = 15.1, 6.8, 1.5 Hz, 1H), 4.93 (d, *J* = 6.9 Hz, 1H), 3.73 (s, 3H), 2.84 (d, *J* = 15.7 Hz, 1H), 2.36 (d, *J* = 15.6 Hz, 1H), 1.73 (dd, *J* = 6.4, 1.3 Hz, 3H), 1.31 (s, 3H), 1.14 (s, 3H); δ_C (126 MHz; $CDCl_3$; Me_4Si) 166.6, 164.3, 130.7, 129.7, 128.2, 115.9, 64.4, 62.5, 51.5, 49.4, 25.0, 19.0, 17.7; HRMS (ESI) m/z: $[M + H]^+$ Calcd for $C_{13}H_{19}N_2O_3$ 251.1390; Found 251.1389.

Methyl (*E*)-7,7-dimethyl-5-oxo-1-styryl-6,7-dihydro-1*H*,5*H*-pyrazolo[1,2-*a*]pyrazole-2-carboxylate (**1i**). Prepared according to the general procedure from **AMI8** (456 mg, 2.0 mmol) and methyl propiolate (214 µL, 2.4 mmol); 620 mg (99% yield); yellow oil; ν_{max}/cm^{-1} (ATR) 2975, 1696, 1600, 1205, 728; δ_H (500 MHz; $CDCl_3$; Me_4Si) 7.44 (d, *J* = 1.5 Hz, 1H), 7.43 – 7.39 (m, 2H), 7.34 – 7.29 (m, 2H), 7.26 – 7.22 (m, 1H), 6.73 (d, *J* = 15.7 Hz, 1H), 6.32 (dd, *J* = 15.8, 6.6 Hz, 1H), 5.15 (d, *J* = 6.6 Hz, 1H), 3.72 (s, 3H), 2.89 (d, *J* = 15.7 Hz, 1H), 2.40 (d, *J* = 15.7 Hz, 1H), 1.33 (s, 3H), 1.18 (s, 3H); δ_C (126 MHz; $CDCl_3$; Me_4Si) 166.7, 164.2, 136.6, 131.7, 129.8, 128.7, 128.6, 127.8, 126.7, 115.7, 64.5, 62.6, 51.6, 49.4, 25.0, 19.1; HRMS (ESI) m/z: $[M + H]^+$ Calcd for $C_{18}H_{21}N_2O_3$ 313.1547; Found 313.1544.

Methyl 7,7-dimethyl-1-(4-methylphenyl)-5-oxo-6,7-dihydro-1*H*,5*H*-pyrazolo[1,2-*a*]pyrazole-2-carboxylate (**1j**). Prepared according to the general procedure from **AMI9** (432 mg, 2.0 mmol) and methyl propiolate (214 µL, 2.4 mmol); 440 mg (73% yield); yellow solid, mp 154–155 °C; ν_{max}/cm^{-1} (ATR) 3081, 1685, 1601, 1191; δ_H (500 MHz; $CDCl_3$; Me_4Si) 7.50 (d, *J* = 1.4 Hz, 1H), 7.32 (d, *J* = 8.0 Hz, 2H), 7.15 (d, *J* = 7.9 Hz, 2H), 5.43 (s, 1H), 3.62 (s, 3H), 2.86 (d, *J* = 15.7 Hz, 1H), 2.38 (d, *J* = 15.7 Hz, 1H), 2.34 (s, 3H), 1.23 (s, 3H), 1.15 (s, 3H); δ_C (126 MHz; $CDCl_3$;

Me_4Si) 166.6, 164.2, 139.1, 137.6, 129.4, 129.2, 127.7, 116.9, 64.5, 64.3, 51.5, 49.4, 24.9, 21.2, 19.0; HRMS (ESI) m/z: $[M + H]^+$ Calcd for $C_{17}H_{21}N_2O_3$ 301.1547; Found 301.1554.

Methyl 1-(2,6-dichlorophenyl)-6,7-dihydro-7,7-dimethyl-5-oxo-1*H*,5*H*-pyrazolo[1,2-*a*]pyrazole-2-carboxy-late (**1k**).[7] Prepared from **AMI10** according to a literature procedure.[7] ^{1}H NMR data is in agreement with the reported values.[7]

Methyl 7,7-dimethyl-5-oxo-1-(3,4,5-trimethoxyphenyl)-6,7-dihydro-1*H*,5*H*-pyrazolo[1,2-*a*]pyrazole-2-carboxylate (**1l**). Prepared according to the general procedure from **AMI11**. ^{1}H NMR data is in agreement with the reported values.[11]

Methyl 7,7-dimethyl-1-(naphthalen-2-yl)-5-oxo-6,7-dihydro-1*H*,5*H*-pyrazolo[1,2-*a*]pyrazole-2-carboxylate (**1m**). Prepared according to general procedure from **AMI12** (504 mg, 2.0 mmol) and methyl propiolate (214 µL, 2.4 mmol); 500 mg (74% yield); yellow solid, amorphic; ν_{max}/cm^{-1} (ATR) 1690, 1599, 1204, 758; δ_H (500 MHz; $CDCl_3$; Me_4Si) 7.87 (s, 1H), 7.86 – 7.81 (m, 3H), 7.61 (dd, *J* = 8.5, 1.7 Hz, 1H), 7.56 (d, *J* = 1.4 Hz, 1H), 7.50 – 7.44 (m, 2H), 5.65 (d, *J* = 1.0 Hz, 1H), 3.59 (s, 3H), 2.89 (d, *J* = 15.8 Hz, 1H), 2.42 (d, *J* = 15.7 Hz, 1H), 1.29 (s, 3H), 1.14 (s, 3H); δ_C (126 MHz; $CDCl_3$; Me_4Si) 166.5, 164.1, 139.3, 133.2, 133.2, 129.5, 128.3, 128.1, 127.8, 126.9, 126.1, 126.0, 125.7, 116.8, 64.8, 64.5, 51.5, 49.5, 25.0, 19.1; HRMS (ESI) m/z: $[M + H]^+$ Calcd for $C_{20}H_{21}N_2O_3$ 337.1547; Found 337.1547.

6-Acetyl-5-(4-methoxyphenyl)-3,3-dimethyl-2,3-dihydro-1*H*,5*H*-pyrazolo[1,2-*a*]pyrazol-1-one (**1n**). Prepared according to the general procedure from **AMI3** (464 mg, 2.0 mmol) and 3-butyn-2-one (188 µL, 2.4 mmol); 590 mg (98% yield); yellow solid, mp 147–149 °C; ν_{max}/cm^{-1} (ATR) 2970, 1722, 1652, 1234; δ_H (500 MHz; $CDCl_3$; Me_4Si) 7.49 (d, *J* = 1.2 Hz, 1H), 7.38 – 7.32 (m, 2H), 6.89 – 6.83 (m, 2H), 5.48 (s, 1H), 3.79 (s, 3H), 2.88 (d, *J* = 15.8 Hz, 1H), 2.41 (d, *J* = 15.7 Hz, 1H), 2.19 (s, 3H), 1.20 (s, 3H), 1.16 (s, 3H); δ_C (126 MHz; $CDCl_3$; Me_4Si) 193.0, 167.4, 159.1, 134.2, 129.2, 128.8, 126.6, 113.7, 64.5, 63.8, 55.2, 49.4, 26.9, 24.9, 18.9; HRMS (ESI) m/z: $[M + H]^+$ Calcd for $C_{17}H_{21}N_2O_3$ 301.1547; Found 301.1543.

Methyl (1*S**,6*R**)-6-benzyloxycarbonylamino-7,7-dimethyl-5-oxo-1-phenyl-6,7-dihydro-1*H*,5*H*-pyrazolo[1,2-*a*]pyrazole-2-carboxylate (**1o**). Prepared according to the general procedure from **AMI13**. ^{1}H NMR data is in agreement with the reported values.[12]

Methyl 7,7-dimethyl-5-oxo-1-(1-tosyl-1*H*-indol-3-yl)-6,7-dihydro-1*H*,5*H*-pyrazolo[1,2-*a*]pyrazole-2-carboxylate (**1p**). Prepared according to the general procedure from **AMI14** (790 mg, 2.0 mmol) and methyl propiolate (214 µL, 2.4 mmol); 612 mg (64% yield); yellow solid, mp 200–201 °C; ν_{max}/cm^{-1} (ATR) 1726, 1687, 1600, 1174, 745; δ_H (500 MHz; $CDCl_3$; Me_4Si) 7.94 (d, *J* = 8.3 Hz, 1H), 7.72 (d, *J* = 8.1 Hz, 2H), 7.68 (d, *J* = 7.9 Hz, 1H), 7.65 (s, 1H), 7.56 (s, 1H), 7.29 (t, *J* = 7.7 Hz, 1H), 7.24 – 7.14 (m, 3H), 5.74 (s, 1H), 3.54 (s, 3H), 2.85 (d, *J* = 15.8 Hz, 1H), 2.41 (d, *J* = 15.8 Hz, 1H), 2.32 (s, 3H), 1.26 (s, 3H), 1.10 (s, 3H); δ_C (126 MHz; $CDCl_3$; Me_4Si) 166.5, 163.8, 144.9, 135.7, 135.2, 129.8 (2C), 129.1, 126.8, 125.0, 124.8, 123.2, 122.9, 120.9, 115.4, 113.8, 64.4, 57.6, 51.4, 49.4, 24.9, 21.6, 18.9; HRMS (ESI) m/z: $[M + H]^+$ Calcd for $C_{25}H_{26}N_3O_5S$ 480.1588; Found 480.1578.

tert-Butyl ((*S*)-1-((*S*)-1-(4-chlorophenyl)-7,7-dimethyl-5-oxo-6,7-dihydro-1*H*,5*H*-pyrazolo[1,2-*a*]pyrazol-2-yl)-1-oxopropan-2-yl)carbamate (**1q**) and *tert*-butyl ((*S*)-1-((*R*)-1-(4-chlorophenyl)-7,7-dimethyl-5-oxo-6,7-dihydro-1*H*,5*H*-pyrazolo[1,2-*a*]pyrazol-2-yl)-1-oxopropan-2-yl)carbamate (**1q'**). Prepared according to the general procedure from **AMI1** (558.2 mg, 2.36 mmol) and *tert*-butyl (*S*)-(3-oxopent-4-yn-2-yl)carbamate (560 mg, 2.83 mmol, prepared according to the literature);[13] ^{1}H NMR analysis of the crude reaction mixture revealed that **1q** and **1q'** are formed in a 1.8:1 ratio. After work-up the crude product weas triturated with diethyl ether. Product **1q** was collected by filtration. Stereochemistry of the isomers was assigned by optical rotation.[11] 240 mg (24% yield); yellow solid, mp 204–205 °C; $[\alpha]^{25}_{589}$ +587 (*c* 0.50, MeOH); ν_{max}/cm^{-1} (ATR) 3306, 3077, 2971, 1721, 1702, 1647, 1583, 1237, 1161, 1014; δ_H (500 MHz; $CDCl_3$; Me_4Si) 7.64 (s, 1H), 7.39 (d, *J* = 8.2 Hz, 2H), 7.30 (d, *J* = 8.2 Hz, 2H), 5.55 (s, 1H), 5.16 (d, *J* = 7.2 Hz, 1H), 4.71 – 4.57 (m, 1H), 2.87 (d, *J* = 15.9 Hz, 1H), 2.45 (d, *J* = 15.9 Hz, 1H), 1.42 (s, 9H), 1.24 – 1.10 (m, 9H); δ_C (126 MHz; $CDCl_3$; Me_4Si) 194.8, 166.8, 155.0, 140.4, 133.6, 129.3, 129.0, 128.6, 122.8, 79.8, 64.4, 64.0, 51.7, 49.3, 28.3, 25.0, 19.6, 19.0; HRMS (ESI) m/z: $[M + H]^+$ Calcd for $C_{22}H_{29}ClN_3O_4$ 434.1841; Found 434.1833.

Methyl 7,7-dimethyl-1-(1-methyl-1*H*-pyrrol-2-yl)-5-oxo-6,7-dihydro-1*H*,5*H*-pyrazolo[1,2-*a*]pyrazole-2-carboxylate (**1r**). Prepared according to the general procedure from **AMI15** (410 mg, 2.0 mmol) and methyl propiolate (214 µL, 2.4 mmol). The reaction mixture was filtered and evaporated in vacuo. Product **1r** was additionally purified by CC (EA/PE, 1:2). 408 mg (71% yield); orange solid, mp 98–100 °C; ν_{max}/cm^{-1} (ATR) 2967, 1694, 1597, 1199; δ_H (500 MHz; $CDCl_3$; Me_4Si) 7.48 (d, *J* = 1.6 Hz, 1H), 6.58 – 6.53 (m, 1H), 6.12 (dd, *J* = 3.5, 1.9 Hz, 1H), 6.06 – 6.02 (m, 1H), 5.64 (d, *J* = 1.4 Hz, 1H), 3.69 (s, 3H), 3.65 (s, 3H), 2.84 (d, *J* = 15.8 Hz, 1H), 2.42 (d, *J* = 15.8 Hz, 1H), 1.25 (s, *J* = 6.5 Hz, 3H), 1.17 (s, 3H); δ_C (126 MHz; $CDCl_3$; Me_4Si) 165.9, 163.9, 129.9, 129.1, 123.9, 115.5, 109.8, 106.6, 63.9, 58.4, 51.6, 49.7, 34.7, 25.1, 19.1; HRMS (ESI) m/z: $[M + H]^+$ Calcd for $C_{15}H_{20}N_3O_3$ 290.1499; Found 290.1496.

3. Optimization studies for the synthesis of pyrazoles 2

Under irradiation alone, **1a** could be converted to the substituted pyrazole **2a**. While the reaction took place in all solvents tested, namely DCM, THF, acetone, MeOH, toluene, MeCN DMF and ethyl acetate, DCM gave the optimal yield. Optimization studies revealed that increased reaction temperature resulted in side reactions. Varying the light source from 400 nm to 450 nm proved less effective (Table SI1).

Table SI1: Optimization studies for the synthesis of **2a**.

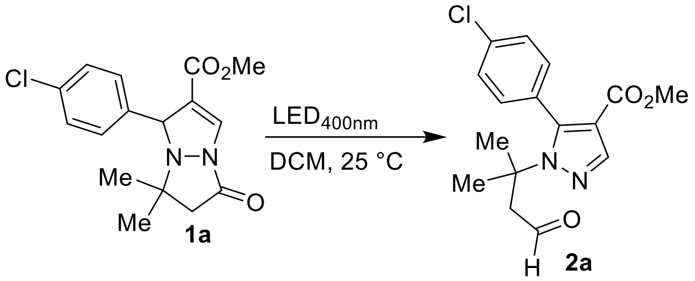

entry	deviation from standard conditions	yield **2a** (%)[a]
1	no deviation	82 (78)[b]
2	THF	66
3	acetone	50
4	MeOH	31
5	toluene	28
6	MeCN	49
7	DMF	40
8	ethyl acetate	49
9	DCE, 50 °C, 12 h	62
10	DCE, 50 °C	52
11	LED_{450nm}	28

Standard reaction conditions: **1a** (0.5 mmol), DCM (anhydrous, degassed, 2.5 mL), LED_{400nm}, 25 °C, under N_2, 24 h. [a] ^{1}H NMR yields were determined with 1,3,5-trimethoxybenzene as an internal standard. [b] Isolated yield of pure product.

4. General procedure for the synthesis of aldehydes 2 and diazepines 3

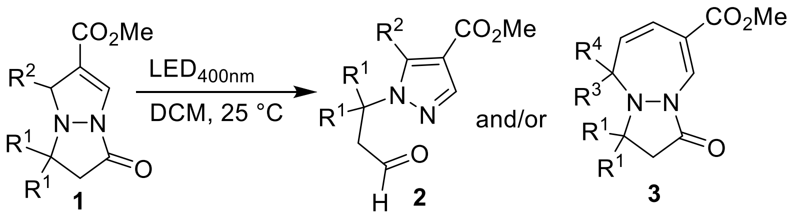

Scheme SI2: Synthesis of compounds **2** and **3**.

A dried 8 mL vial was charged with **1** (0.5 mmol) and dry DCM (2.5 mL) and sealed off with a screw cap with a septum. The resulting solution was degassed via needle by three freeze-pump-thaw cycles and irradiated with $LED_{400\ nm}$ under nitrogen atmosphere for 24–48 h at 25 °C. Products **2** and **3** were obtained by evaporation of the solvent and purification by CC (EA/PE).

5. Characterization data of novel compounds 2 and 3

Methyl 5-(4-chlorophenyl)-1-(2-methyl-4-oxobutan-2-yl)-1*H*-pyrazole-4-carboxylate (**2a**). Prepared according to the general procedure from **1a** (161 mg, 0.5 mmol); CC (EA/PE, 1:2); 126 mg (78% yield); colorless oil; ν_{max}/cm^{-1} (ATR) 2950, 1715, 1546; δ_H (500 MHz; $CDCl_3$; Me_4Si) 9.71 (t, *J* = 1.9 Hz, 1H), 7.90 (s, 1H), 7.44 (d, *J* = 8.4 Hz, 2H), 7.28 (d, *J* = 8.5 Hz, 2H), 3.63 (s, 3H), 2.94 (d, *J* = 1.9 Hz, 2H), 1.48 (s, 6H); δ_C (126 MHz; $CDCl_3$; Me_4Si) 199.2, 163.1, 144.6, 139.4, 135.5, 131.6, 129.9, 128.5, 115.1, 63.4, 55.1, 51.1, 29.6; HRMS (ESI) m/z: $[M + H]^+$ Calcd for $C_{16}H_{18}ClN_2O_3$ 321.1000; Found 321.1000.

Methyl 1-(2-methyl-4-oxobutan-2-yl)-5-phenyl-1*H*-pyrazole-4-carboxylate (**2b**). Prepared according to the general procedure from **1b** (143 mg, 0.5 mmol); CC (EA/PE, 1:2); 123 mg (86% yield); white solid; mp 86–87 °C; ν_{max}/cm^{-1} (ATR) 2984, 1720, 1713, 1546; δ_H (500 MHz; $CDCl_3$; Me_4Si) 9.71 (t, *J* = 2.1 Hz, 1H), 7.91 (s, 1H), 7.50 – 7.42 (m, 3H), 7.36 – 7.30 (m, 2H), 3.61 (s, 3H), 2.91 (d, *J* = 2.1 Hz, 2H), 1.47 (s, 6H); δ_C (126 MHz; $CDCl_3$; Me_4Si) 199.5, 163.2, 145.9, 139.3, 131.3, 130.2, 129.2, 128.0, 114.9, 63.2, 55.1, 51.0, 29.5; HRMS (ESI) m/z: $[M + H]^+$ Calcd for $C_{16}H_{19}N_2O_3$ 287.1390; Found 287.1387.

Methyl 5-(4-methoxyphenyl)-1-(2-methyl-4-oxobutan-2-yl)-1*H*-pyrazole-4-carboxylate (**2c**). Prepared according to the general procedure from **1c** (158 mg, 0.5 mmol); CC (EA/PE, 1:1); 134 mg (85% yield); white solid; mp 122–123 °C; ν_{max}/cm^{-1} (ATR) 2841, 1730, 1715, 1612, 1553; δ_H (500 MHz; $CDCl_3$; Me_4Si) 9.70 (t, *J* = 2.0 Hz, 1H), 7.89 (s, 1H), 7.23 (d, *J* = 8.7 Hz, 2H), 6.98 (d, *J* = 8.7 Hz, 2H), 3.86 (s, 3H), 3.63 (s, 3H), 2.91 (d, *J* = 1.9 Hz, 2H), 1.49 (s, 6H); δ_C (126 MHz; $CDCl_3$; Me_4Si) 199.6, 163.3, 160.1, 145.9, 139.2, 131.4, 123.0, 114.9, 113.6, 63.1, 55.2, 55.1, 51.0, 29.5; HRMS (ESI) m/z: $[M + H]^+$ Calcd for $C_{17}H_{21}N_2O_4$ 317,1496; Found 317.1491.

Methyl 5-(4-cyanophenyl)-1-(2-methyl-4-oxobutan-2-yl)-1*H*-pyrazole-4-carboxylate (**2d**). Prepared according to the general procedure from **1d** (156 mg, 0.5 mmol); CC (EA/PE, 1:1); 137 mg (88% yield); white solid; mp 133–134 °C; ν_{max}/cm^{-1} (ATR) 2986, 2227, 1712, 1553; δ_H (500 MHz; $CDCl_3$; Me_4Si) 9.72 (t, *J* = 1.6 Hz, 1H), 7.91 (s, 1H), 7.76 (d, *J* = 8.3 Hz, 2H), 7.51 (d, *J* = 8.3 Hz, 2H), 3.63 (s, 3H), 2.99 (d, *J* = 1.5 Hz, 2H), 1.46 (s, 6H); δ_C (126 MHz; $CDCl_3$; Me_4Si) 198.9, 162.9, 143.7, 139.5, 136.6, 131.8, 131.2, 118.2, 115.1, 113.3, 63.5, 55.0, 51.2, 29.7; HRMS (ESI) m/z: $[M + H]^+$ Calcd for $C_{17}H_{18}N_3O_3$ 312.1343; Found 312.1341.

Methyl 5-(furan-2-yl)-1-(2-methyl-4-oxobutan-2-yl)-1*H*-pyrazole-4-carboxylate (**2e**). Prepared according to the general procedure from **1e** (138 mg, 0.5 mmol); CC (EA/PE, 1:2); 57 mg (41% yield); colorless oil; ν_{max}/cm^{-1} (ATR) 2951, 1715, 1537; δ_H (500 MHz; $CDCl_3$; Me_4Si) 9.68 (t, *J* = 2.1 Hz, 1H), 7.92 (s, 1H), 7.62 (d, *J* = 1.7 Hz, 1H), 6.62 (d, *J* = 3.3 Hz, 1H), 6.58 (dd, *J* = 3.3, 1.9 Hz, 1H), 3.72 (s, 3H), 2.92 (d, *J* = 2.1 Hz, 2H), 1.54 (s, 6H); δ_C (126 MHz; $CDCl_3$; Me_4Si) 199.5, 162.8, 143.3, 140.7, 139.3, 134.6, 117.0, 114.2, 111.5, 63.0, 54.6, 51.4, 28.4; HRMS (ESI) m/z: $[M + H]^+$ Calcd for $C_{14}H_{17}N_2O_4$ 277.1183; Found 277.1180.

Methyl 5-methyl-1-(2-methyl-4-oxobutan-2-yl)-1*H*-pyrazole-4-carboxylate (**2f**). Prepared according to the general procedure from **1f** (112 mg, 0.5 mmol); CC (EA/PE, 1:2); 86 mg (77% yield); colorless oil; ν_{max}/cm^{-1} (ATR) 2951, 1707, 1551; δ_H (500 MHz; $CDCl_3$; Me_4Si) 9.73 (t, *J* = 2.0 Hz, 1H), 7.75 (s, 1H), 3.81 (s, 3H), 3.01 (d, *J* = 2.0 Hz, 2H), 2.77 (s, 3H), 1.76 (s, 6H); δ_C (126

MHz; $CDCl_3$; Me_4Si) 199.4, 164.3, 142.8, 139.1, 113.3, 62.0, 54.6, 51.0, 28.2, 12.8; HRMS (ESI) m/z: $[M + H]^+$ Calcd for $C_{11}H_{17}N_2O_3$ 225.1234; Found 225.1233.

Methyl 1-(3-oxopropyl)-5-phenyl-1*H*-pyrazole-4-carboxylate (**2g**). Prepared according to the general procedure from **1g** (129 mg, 0.5 mmol); CC (EA/PE, 1:1); 92 mg (71% yield); white solid; mp 102–103 °C; ν_{max}/cm^{-1} (ATR) 2824, 2723, 1712, 1549; δ_H (500 MHz; $CDCl_3$; Me_4Si) 9.73 (s, 1H), 7.98 (s, 1H), 7.53 – 7.44 (m, 3H), 7.42 – 7.34 (m, 2H), 4.27 (t, *J* = 6.8 Hz, 2H), 3.68 (s, 3H), 3.00 (td, *J* = 6.7, 0.6 Hz, 2H); δ_C (126 MHz; $CDCl_3$; Me_4Si) 198.9, 163.3, 146.2, 141.4, 129.9, 129.6, 128.6, 128.5, 112.6, 51.1, 43.0, 43.0; HRMS (ESI) m/z: $[M + H]^+$ Calcd for $C_{14}H_{15}N_2O_3$ 259.1077; Found 259.1078.

Methyl (*E*)-1-(2-methyl-4-oxobutan-2-yl)-5-(prop-1-en-1-yl)-1*H*-pyrazole-4-carboxylate (**2h**) and methyl 3,3-dimethyl-1-oxo-5-methyl-2,3-dihydro-1*H*,5*H*-pyrazolo[1,2-*a*][1,2]diazepine-8-carboxylate (**3h**). Prepared according to the general procedure from **1h** (125 mg, 0.5 mmol). Purification by CC (EA/PE, 1:1) gave products **2h** and **3h**. **2h**; 51 mg (41% yield); colorless oil; ν_{max}/cm^{-1} (ATR) 2949, 1716, 1535; δ_H (500 MHz; $CDCl_3$; Me_4Si) 9.69 (t, *J* = 2.1 Hz, 1H), 7.78 (s, 1H), 6.36 (dq, *J* = 15.8, 1.6 Hz, 1H), 6.21 (dq, *J* = 15.8, 6.6 Hz, 1H), 3.77 (s, 3H), 2.98 (d, *J* = 2.1 Hz, 2H), 1.97 (dd, *J* = 6.6, 1.7 Hz, 3H), 1.73 (s, 6H); δ_C (126 MHz; $CDCl_3$; Me_4Si) 199.6, 163.8, 143.5, 139.7, 137.4, 117.8, 113.4, 62.3, 54.5, 51.1, 28.7, 19.0; HRMS (ESI) m/z: $[M + H]^+$ Calcd for $C_{13}H_{19}N_2O_3$ 251.1390; Found 251.1387. **3h**; 31 mg (25% yield); colorless oil; ν_{max}/cm^{-1} (ATR) 2973, 1702, 1640, 1604, 1197; δ_H (500 MHz; $CDCl_3$; Me_4Si) 8.16 (d, *J* = 1.3 Hz, 1H), 6.47 (dd, *J* = 11.8, 1.4 Hz, 1H), 6.02 (dd, *J* = 11.8, 7.4 Hz, 1H), 4.14 (p, *J* = 6.7 Hz, 1H), 3.78 (s, 3H), 2.73 (d, *J* = 17.0 Hz, 1H), 2.29 (d, *J* = 17.1 Hz, 1H), 1.45 (s, 3H), 1.26 (s, 3H), 1.12 (d, *J* = 6.5 Hz, 3H); δ_C (126 MHz; $CDCl_3$; Me_4Si) 172.1, 167.7, 134.6, 131.7, 121.4, 109.8, 59.9, 58.3, 52.0, 43.4, 31.1, 23.9, 15.7; HRMS (ESI) m/z: $[M + H]^+$ Calcd for $C_{13}H_{19}N_2O_3$ 251.1390; Found 251.1388.

Methyl 3,3-dimethyl-1-oxo-5-phenyl-2,3-dihydro-1*H*,5*H*-pyrazolo[1,2-*a*][1,2]diazepine-8-carboxylate (**3i**) Prepared according to the general procedure from **1i** (156 mg, 0.5 mmol); LED_{450nm} were used; CC (EA/PE, 1:2); 117 mg (75% yield); pale yellow solid; mp 151–152 °C; ν_{max}/cm^{-1} (ATR) 2973, 1704, 1628, 1601; δ_H (500 MHz; $CDCl_3$; Me_4Si) 8.28 (d, *J* = 1.1 Hz, 1H), 7.32 – 7.27 (m, 3H), 7.17 – 7.12 (m, 2H), 6.60 (dd, *J* = 12.0, 0.5 Hz, 1H), 6.11 (dd, *J* = 12.0, 7.2 Hz, 1H), 5.06 (d, *J* = 7.2 Hz, 1H), 3.84 (s, 3H), 1.86 (d, *J* = 16.5 Hz, 1H), 1.65 (d, *J* = 16.7 Hz, 1H), 1.58 (s, 3H), 1.22 (s, 3H); δ_C (126 MHz; $CDCl_3$; Me_4Si) 173.3, 167.6, 139.5, 133.0, 132.9, 129.4, 128.8, 128.5, 120.7, 111.5, 67.4, 61.8, 52.1, 42.1, 30.5, 23.8; HRMS (ESI) m/z: $[M + H]^+$ Calcd for $C_{18}H_{21}N_2O_3$ 313.1547; Found 313.1543.

6. Optimization studies for the synthesis of pyrazoles 4

In the presence of diethyl bromomalonate, **1a** could be converted to the N1-acryloyl substituted pyrazole **4a**. Optimization studies revealed that solvents such as MeCN, toluene, and $CHCl_3$ were less suitable, resulting in lower conversions. Considering the fact that an equivalent of HBr is generated in this reaction, a series of bases were subsequently introduced to promote the conversion. The reaction carried out without a base proved to be inefficient, while pyridine allowed the formation of **4a** in a low 38% yield. Furthermore, when inorganic bases (Cs_2CO_3, K_2CO_3 and KH_2PO_4) were examined, Cs_2CO_3 proved to be the most effective as it gave the desired product **4a** in reasonable 50–60% yield. Varying the light source above 450 nm proved less effective, as no product formation was observed at 510 nm. Moreover, for the reaction to be productive the exclusion of oxygen was necessary, since no product was detected in the case when the reaction was run under air atmosphere. It should be mentioned that the presence of moisture decreased the yield of **4a** substantially (Table SI2, entry 10).

Table SI2: Optimization studies for the synthesis of **4a**.

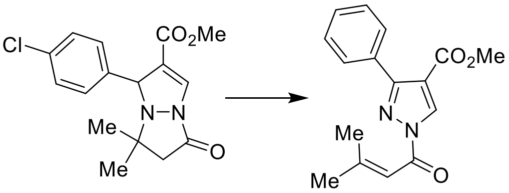

entry	deviation from standard conditions	yield **4a** (%)[a]
1	MeCN, Cs_2CO_3 (1.0 equiv)	54
2	toluene, Cs_2CO_3 (1.0 equiv)	50
3	$CHCl_3$, Cs_2CO_3 (1.0 equiv)	47
4	Cs_2CO_3 (1.0 equiv)	64
5	KH_2PO_4 or K_2CO_3 (1.0 equiv)	0
6	no deviation	85(78)[b][80][c]
7	pyridine (1.5 equiv)	38
8	no base	0
9	diethyl bromomalonate (1.5 equiv)	55
10	H_2O (10 equiv)	24[d]
11	air atmosphere	0
12	400 nm, (510 nm)	75 (0)
13	no light	0

Standard reaction conditions: **1a** (0.5 mmol), diethyl bromomalonate (2.0 equiv), 2,6-lutidine (1.5 equiv), DCM (anhydrous, degassed, 2.5 mL), LED_{450nm}, 25 °C, under N_2, 18 h. [a]^{1}H NMR yields were determined with 1,3,5-trimethoxybenzene as an internal standard. [b]Isolated yield of pure product. [c]3.3 mmol scale yield. [d]**5a** is present as the major product.

7. General procedure for the synthesis of pyrazoles 4

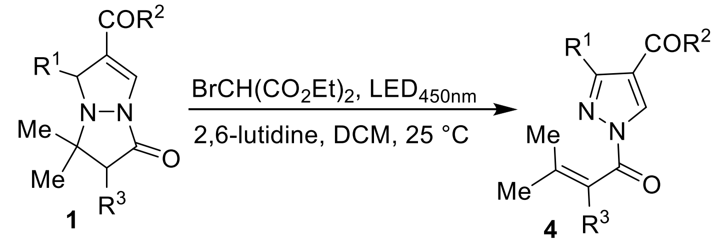

Scheme SI3: Synthesis of compounds **4**.

A dried 8 mL vial was charged with **1** (0.5 mmol) and dry DCM (2.5 mL) and sealed off with a screw cap with a septum. The resulting solution was degassed via needle by three freeze-pump-thaw cycles. Diethyl bromomalonate (185 µL, 92% purity, 2.0 equiv) and 2,6-lutidine (87 µL, 1.5 equiv) were added. The solution was irradiated with a blue $LED_{450\,nm}$ under nitrogen atmosphere for 18 h at 25 °C. Products **4** were isolated by CC or CC (EA/PE) and dried under vacuum. In the optimization studies, inorganic bases were added prior to degassing.

8. Characterization data of novel compounds 4

Methyl 3-(4-chlorophenyl)-1-(3-methylbut-2-enoyl)-1*H*-pyrazole-4-carboxylate (**4a**). Prepared according to the general procedure from **1a** (161 mg, 0.5 mmol); CC (EA/PE, 1:6); 126 mg (79% yield); white solid; mp 119–121 °C; ν_{max}/cm^{-1} (ATR) 1729, 1717, 1625, 1147, 1133; δ_H (500 MHz; $CDCl_3$; Me_4Si) 8.84 (s, 1H), 7.83 – 7.76 (m, 2H), 7.46 – 7.38 (m, 2H), 7.18 – 7.14 (m, 1H), 3.83 (s, 3H), 2.36 (d, *J* = 1.1 Hz, 3H), 2.11 (d, *J* = 1.2 Hz, 3H); δ_C (126 MHz; $CDCl_3$; Me_4Si) 165.7, 162.9, 162.2, 153.7, 135.3, 134.2, 130.7, 129.8, 128.3, 114.8, 113.2, 51.8, 28.7, 21.7; HRMS (ESI) m/z: $[M + H]^+$ Calcd for $C_{16}H_{16}ClN_2O_3$ 319.0844; Found 319.0841.

Methyl 1-(3-methylbut-2-enoyl)-3-phenyl-1*H*-pyrazole-4-carboxylate (**4b**). Prepared according to the general procedure from **1b** (143 mg, 0.5 mmol); CC (EA/PE, 1:7); 126 mg (75% yield); white solid; mp 109–110 °C; ν_{max}/cm^{-1} (ATR) 1731, 1712, 1628, 1138; δ_H (500 MHz; $CDCl_3$; Me_4Si) 8.87 (s, 1H), 7.88 – 7.81 (m, 2H), 7.51 – 7.43 (m, 3H), 7.23 – 7.18 (m, 1H), 3.84 (s, 3H), 2.39 (d, *J* = 1.0 Hz, 3H), 2.13 (d, *J* = 1.0 Hz, 3H); δ_C (126 MHz; $CDCl_3$; Me_4Si) 165.3, 163.0, 162.4, 154.8, 134.0, 131.4, 129.3, 129.2, 128.0, 114.9, 113.4, 51.7, 28.6, 21.7; HRMS (ESI) m/z: $[M + H]^+$ Calcd for $C_{16}H_{17}N_2O_3$ 285.1234; Found 285.1233.

Methyl 1-(3-methylbut-2-enoyl)-3-(p-tolyl)-1*H*-pyrazole-4-carboxylate (**4c**). Prepared according to the general procedure from **1j** (150 mg, 0.5 mmol); CC (EA/PE, 1:2); 113 mg (76% yield); white solid; mp 109–110 °C; ν_{max}/cm^{-1} (ATR) 1736, 1711, 1630, 1141, 1125; δ_H (500 MHz; $CDCl_3$; Me_4Si) 8.86 (s, 1H), 7.74 (d, *J* = 8.1 Hz, 2H), 7.30 – 7.26 (m, 2H), 7.23 – 7.20 (m, 1H), 3.84 (s, 3H), 2.43 (s, 3H), 2.38 (d, *J* = 0.7 Hz, 3H), 2.12 (d, *J* = 0.8 Hz, 3H); δ_C (126 MHz; $CDCl_3$; Me_4Si) 165.1, 163.1, 162.4, 154.9, 139.2, 134.0, 129.2, 128.8, 128.5, 114.9, 113.5, 51.7, 28.6, 21.7, 21.4; HRMS (ESI) m/z: $[M + H]^+$ Calcd for $C_{17}H_{19}N_2O_3$ 299.1390; Found 299.1389.

Methyl 3-(4-cyanophenyl)-1-(3-methylbut-2-enoyl)-1*H*-pyrazole-4-carboxylate (**4d**). Prepared according to the general procedure from **1d** (156 mg, 0.5 mmol); CC (EA/PE, 1:4); 116 mg (75%

yield); white solid; mp 150–151 °C; ν_{max}/cm^{-1} (ATR) 2224, 1732, 1709, 1626, 1142; δ_H (500 MHz; $CDCl_3$; Me_4Si) 8.88 (s, 1H), 8.02 (d, *J* = 8.4 Hz, 2H), 7.75 (d, *J* = 8.4 Hz, 2H), 7.20 – 7.12 (m, 1H), 3.85 (s, 3H), 2.38 (d, *J* = 0.8 Hz, 3H), 2.14 (d, *J* = 0.9 Hz, 3H); δ_C (126 MHz; $CDCl_3$; Me_4Si) 166.4, 162.7, 162.0, 152.8, 135.9, 134.4, 131.8, 130.1, 118.8, 114.9, 113.0, 112.7, 51.9, 28.7, 21.8; HRMS (ESI) m/z: $[M + H]^+$ Calcd for $C_{17}H_{16}N_3O_3$ 310.1186; Found 310.1182.

Methyl 3-(4-methoxyphenyl)-1-(3-methylbut-2-enoyl)-1*H*-pyrazole-4-carboxylate (**4e**). Prepared according to the general procedure from **1c** (158 mg, 0.5 mmol); CC (EA/PE, 1:5); 121 mg (77% yield); white solid; mp 108–109 °C; ν_{max}/cm^{-1} (ATR) 1734, 1706, 1616, 1138; δ_H (500 MHz; $CDCl_3$; Me_4Si) 8.85 (s, 1H), 7.86 – 7.78 (m, 2H), 7.22 – 7.19 (m, 1H), 7.03 – 6.95 (m, 2H), 3.88 (s, 3H), 3.84 (s, 3H), 2.38 (d, *J* = 0.7 Hz, 3H), 2.12 (d, *J* = 0.8 Hz, 3H); δ_C (126 MHz; $CDCl_3$; Me_4Si) 165.0, 163.1, 162.4, 160.5, 154.5, 134.1, 130.7, 123.8, 114.7, 113.5, 113.5, 55.3, 51.7, 28.6, 21.6; HRMS (ESI) m/z: $[M + H]^+$ Calcd for $C_{17}H_{19}N_2O_4$ 315.1339; Found 315.1334.

Methyl 3-(2,6-dichlorophenyl)-1-(3-methylbut-2-enoyl)-1*H*-pyrazole-4-carboxylate (**4f**). Prepared according to the general procedure from **1k** (178 mg, 0.5 mmol); CC (EA/PE, 1:7); 108 mg (61% yield); white solid; mp 140–141 °C; ν_{max}/cm^{-1} (ATR) 1726, 1628, 1361, 1153; δ_H (500 MHz; $CDCl_3$; Me_4Si) 8.91 (s, 1H), 7.45 – 7.41 (m, 2H), 7.36 – 7.32 (m, 1H), 7.15 – 7.12 (m, 1H), 3.76 (s, 3H), 2.39 (d, *J* = 0.8 Hz, 3H), 2.10 (d, *J* = 0.8 Hz, 3H); δ_C (126 MHz; $CDCl_3$; Me_4Si) 165.8, 162.2, 162.1, 150.5, 135.7, 132.7, 130.8, 130.6, 127.8, 116.5, 113.3, 51.7, 28.6, 21.8; HRMS (ESI) m/z: $[M + H]^+$ Calcd for $C_{16}H_{15}N_2Cl_2O_3$ 353.0454; Found 353.0449.

Methyl 1-(3-methylbut-2-enoyl)-3-(3,4,5-trimethoxyphenyl)-1*H*-pyrazole-4-carboxylate (**4g**). Prepared according to the general procedure from **1l** (188 mg, 0.5 mmol); CC (EA/PE, 2:5); 108 mg (58% yield); white solid; mp 104–105 °C; ν_{max}/cm^{-1} (ATR) 1732, 1712, 1617, 1419, 1398, 1117; δ_H (500 MHz; $CDCl_3$; Me_4Si) 8.85 (d, *J* = 3.7 Hz, 1H), 7.18 (dd, *J* = 5.0, 3.8 Hz, 1H), 7.16 (s, 2H), 3.93 (s, 6H), 3.90 (s, 3H), 3.84 (s, 3H), 2.37 (d, *J* = 1.0 Hz, 3H), 2.12 (d, *J* = 1.1 Hz, 3H); δ_C (126 MHz; $CDCl_3$; Me_4Si) 165.5, 163.0, 162.3, 154.5, 152.9, 139.1, 134.4, 126.6, 114.9, 113.3, 106.9, 60.9, 56.3, 51.8, 28.6, 21.7; HRMS (ESI) m/z: $[M + H]^+$ Calcd for $C_{19}H_{23}N_2O_6$ 375.1551; Found 375.1546.

Methyl (*E*)-1-(3-methylbut-2-enoyl)-3-styryl-1*H*-pyrazole-4-carboxylate (**4h**) and methyl (*Z*)-1-(3-methylbut-2-enoyl)-3-styryl-1*H*-pyrazole-4-carboxylate (**4h'**). Prepared according to the general procedure from **1i** (156 mg, 0.5 mmol); **4h** : **4h'** = 3.3 : 1; CC (EA/PE, 1:6); **4h**: 36 mg (23% yield); white solid; mp 93–94 °C; ν_{max}/cm^{-1} (ATR) 1712, 1628, 1552, 1365, 1222, 1101; δ_H (500 MHz; $CDCl_3$; Me_4Si) 8.75 (s, 1H), 7.69 (d, *J* = 16.4 Hz, 1H), 7.66 – 7.57 (m, 3H), 7.38 (t, *J* = 7.4 Hz, 2H), 7.31 (t, *J* = 7.2 Hz, 1H), 7.20 (s, 1H), 3.89 (s, 3H), 2.36 (s, 3H), 2.15 (s, 3H); δ_C (126 MHz; $CDCl_3$; Me_4Si) 165.2, 163.3, 162.4, 152.2, 136.6, 134.0, 133.2, 128.7, 128.5, 127.2, 117.4, 114.9, 113.5, 51.7, 28.7, 21.7; HRMS (ESI) m/z: $[M + H]^+$ Calcd for $C_{18}H_{19}N_2O_3$ 311.1390; Found 311.1393.

Methyl 3-methyl-1-(3-methylbut-2-enoyl)-1*H*-pyrazole-4-carboxylate (**4i**). Prepared according to the general procedure from **1f** (112 mg, 0.5 mmol); CC (EA/PE, 1:6); 72 mg (65% yield); white solid; mp 66–67 °C; ν_{max}/cm^{-1} (ATR) 1732, 1703, 1630, 1564, 1273, 1139, 1100; δ_H (500

MHz; $CDCl_3$; Me_4Si) 8.69 (s, 1H), 7.06 (s, 1H), 3.85 (s, 3H), 2.51 (s, 3H), 2.33 (s, 3H), 2.10 (s, 3H); δ_C (126 MHz; $CDCl_3$; Me_4Si) 164.6, 163.5, 162.3, 153.5, 132.9, 115.6, 113.5, 51.5, 28.5, 21.6, 13.8; HRMS (ESI) m/z: $[M + H]^+$ Calcd for $C_{11}H_{15}N_2O_3$ 223.1077; Found 223.1076.

Methyl 1-(3-methylbut-2-enoyl)-3-(naphthalen-2-yl)-1*H*-pyrazole-4-carboxylate (**4j**). Prepared according to the general procedure from **1m** (168 mg, 0.5 mmol); CC (EA/PE, 1:7); 127 mg (76% yield); white solid; mp 120–121 °C; ν_{max}/cm^{-1} (ATR) 1728, 1709, 1117; δ_H (500 MHz; $CDCl_3$; Me_4Si) 8.89 (s, 1H), 8.38 (s, 1H), 7.95 – 7.84 (m, 4H), 7.54 – 7.46 (m, 2H), 7.23 (s, 1H), 3.83 (s, 3H), 2.37 (d, *J* = 0.5 Hz, 3H), 2.11 (d, *J* = 0.5 Hz, 3H); δ_C (126 MHz; $CDCl_3$; Me_4Si) 165.4, 163.1, 162.4, 154.8, 134.2, 133.7, 133.0, 129.1, 128.8, 128.7, 127.7, 127.5, 126.8, 126.7, 126.2, 115.2, 113.4, 51.8, 28.7, 21.7; HRMS (ESI) m/z: $[M + H]^+$ Calcd for $C_{20}H_{19}N_2O_3$ 335.1390; Found 335.1385.

1-(4-Acetyl-3-(4-methoxyphenyl)-1*H*-pyrazol-1-yl)-3-methylbut-2-en-1-one (**4k**). Prepared according to the general procedure from **1n** (150 mg, 0.5 mmol); CC (EA/PE, 1:4); 75 mg (50% yield); white solid; mp 139–140 °C; ν_{max}/cm^{-1} (ATR) 3122, 1710, 1686, 1614, 1147; δ_H (500 MHz; $CDCl_3$; Me_4Si) 8.82 (s, 1H), 7.77 – 7.71 (m, 2H), 7.20 – 7.15 (m, 1H), 7.00 – 6.93 (m, 2H), 3.86 (s, 3H), 2.48 (s, 3H), 2.37 (d, *J* = 0.9 Hz, 3H), 2.11 (d, *J* = 1.0 Hz, 3H); δ_C (126 MHz; $CDCl_3$; Me_4Si) 192.4, 165.3, 162.6, 160.5, 154.1, 133.6, 130.7, 124.0, 123.1, 113.5, 113.5, 55.3, 29.4, 28.6, 21.7; HRMS (ESI) m/z: $[M + H]^+$ Calcd for $C_{17}H_{19}N_2O_3$ 299.1390; Found 299.1387.

Methyl 1-(2-(((benzyloxycarbonylamino)-3-methylbut-2-enoyl)-3-phenyl-1*H*-pyrazole-4-carboxylate (**4l**). Prepared according to the general procedure from **1o** (218 mg, 0.5 mmol); CC (EA/PE, 1:3); 136 mg (63% yield); white solid; mp 106–107 °C; ν_{max}/cm^{-1} (ATR) 3296, 1718, 1692, 1518, 1269; δ_H (500 MHz; $CDCl_3$; Me_4Si) 8.80 (s, 1H), 7.70 (d, *J* = 6.8 Hz, 2H), 7.47 – 7.34 (m, 3H), 7.29 – 7.17 (m, 5H), 6.62 (s, 1H), 5.03 (s, 2H), 3.82 (s, 3H), 2.04 (s, 3H), 1.94 (s, 3H); δ_C (126 MHz; $CDCl_3$; Me_4Si) 162.66, 162.32, 155.33, 154.49, 143.71, 135.71, 135.35, 130.92, 129.36, 128.51, 128.30, 128.24, 127.97, 122.41, 114.75, 77.24, 67.46, 51.76, 20.99, 20.90; HRMS (ESI) m/z: $[M - Cbz + H]^+$ Calcd for $C_{11}H_{11}N_2O_2$ 203.0815; Found 203.0816.

Methyl 1-(3-methylbut-2-enoyl)-3-(1-tosyl-1H-indol-3-yl)-1*H*-pyrazole-4-carboxylate (**4m**). Prepared according to the general procedure from **1p** (240 mg, 0.5 mmol); CC (EA/PE, 1:3); 122 mg (51% yield); white solid; mp 199–200 °C; ν_{max}/cm^{-1} (ATR) 1715, 1623, 1139; δ_H (500 MHz; $CDCl_3$; Me_4Si) 8.87 (s, 1H), 8.85 (s, 1H), 8.29 (d, *J* = 7.1 Hz, 1H), 8.05 (d, *J* = 7.6 Hz, 1H), 7.86 (d, *J* = 8.4 Hz, 2H), 7.35 (dqd, *J* = 14.7, 7.3, 1.2 Hz, 2H), 7.22 (d, *J* = 8.2 Hz, 2H), 7.20 – 7.18 (m, 1H), 3.91 (s, 3H), 2.37 (d, *J* = 0.9 Hz, 3H), 2.32 (s, 3H), 2.14 (d, *J* = 1.0 Hz, 3H); δ_C (126 MHz; $CDCl_3$; Me_4Si) 165.4, 163.0, 162.2, 148.0, 145.1, 135.1, 134.8, 134.0, 129.9 (2C), 129.1, 127.1, 125.0, 123.8, 122.6, 115.0, 113.4, 113.3, 112.8, 51.9, 28.8, 21.7, 21.6; HRMS (ESI) m/z: $[M + H]^+$ Calcd for $C_{25}H_{24}N_3O_5S$ 478.1431; Found 478.1420.

tert-Butyl (*S*)-(1-(3-(4-chlorophenyl)-1-(3-methylbut-2-enoyl)-1*H*-pyrazol-4-yl)-1-oxopropan-2-yl)carbamate (**4n**). Prepared according to the general procedure from **1q** (217 mg, 0.5 mmol); CC (EA/PE, 1:3); 182 mg (84% yield); white solid; mp 134–135 °C; $[\alpha]_{589}^{25}$ -31 (*c* 0.25, MeOH); ν_{max}/cm^{-1} (ATR) 3327, 1714, 1688, 1666, 1631, 1139; δ_H (500 MHz; $CDCl_3$; Me_4Si) 8.95

(s, 1H), 7.75 – 7.69 (m, 2H), 7.41 (d, *J* = 8.5 Hz, 2H), 7.15 (s, 1H), 5.33 (d, *J* = 7.5 Hz, 1H), 4.93 (p, *J* = 7.1 Hz, 1H), 2.37 (s, 3H), 2.12 (s, 3H), 1.44 (s, 10H), 1.41 (d, *J* = 7.1 Hz, 3H); δ_C (126 MHz; $CDCl_3$; Me_4Si) 194.4, 166.2, 162.1, 155.2, 154.0, 135.5, 133.3, 130.7, 129.9, 128.3, 119.6, 113.1, 80.0, 53.5, 28.7, 28.3, 21.8, 19.4; HRMS (ESI) m/z: $[M – Boc]^+$ Calcd for $C_{17}H_{19}ClN_3O_2$ 332.1160; Found 332.1153.

Methyl 3-(1-methyl-1*H*-pyrrol-2-yl)-1-(3-methylbut-2-enoyl)-1*H*-pyrazole-4-carboxylate (**4o**) and diethyl 2-(5-(4-(methoxycarbonyl)-1-(3-methylbut-2-enoyl)-1*H*-pyrazol-3-yl)-1-methyl-1*H*-pyrrol-2-yl)malonate (**4o'**). Prepared according to the general procedure from **1r** (145 mg, 0.5 mmol), diethyl bromomalonate (370 µL, 92% purity, 4.0 equiv) and 2,6-lutidine (174 µL, 3.0 equiv); products were separated by CC (EA/PE, 2:7); **4o**; 22 mg (15% yield); white solid; mp 98–99 °C; ν_{max}/cm^{-1} (ATR) 1711, 1624, 1123; δ_H (500 MHz; $CDCl_3$; Me_4Si) 8.82 (s, 1H), 7.12 (s, 1H), 6.79 (d, *J* = 2.0 Hz, 1H), 6.76 (s, 1H), 6.25 – 6.16 (m, 1H), 3.83 (s, 3H), 3.78 (s, 3H), 2.36 (s, 3H), 2.09 (s, 3H); δ_C (126 MHz; $CDCl_3$; Me_4Si) 165.0, 162.8, 162.3, 147.4, 133.5, 125.4, 123.1, 115.7, 113.9, 113.4, 107.7, 51.8, 36.1, 28.7, 21.6; HRMS (ESI) m/z: $[M + H]^+$ Calcd for $C_{15}H_{18}N_3O_3$ 288.1343; Found 288.1338. **4o'**; 112 mg (50% yield); yellow oil; ν_{max}/cm^{-1} (ATR) 1716, 1627, 1133; δ_H (500 MHz; $CDCl_3$; Me_4Si) 8.83 (s, 1H), 7.16 – 7.07 (m, 1H), 6.64 (d, *J* = 3.9 Hz, 1H), 6.32 (d, *J* = 3.9 Hz, 1H), 4.79 (s, 1H), 4.26 (qdd, *J* = 14.3, 9.0, 5.4 Hz, 4H), 3.81 (s, 3H), 3.63 (s, 3H), 2.36 (d, *J* = 1.0 Hz, 3H), 2.09 (d, *J* = 1.1 Hz, 3H), 1.31 (t, *J* = 7.1 Hz, 6H); δ_C (126 MHz; $CDCl_3$; Me_4Si) 167.3, 165.4, 162.6, 162.2, 147.3, 133.4, 126.3, 124.8, 116.2, 113.3, 112.6, 109.6, 62.0, 51.8, 51.1, 32.8, 28.7, 21.7, 14.1; HRMS (ESI) m/z: $[M + H]^+$ Calcd for $C_{22}H_{28}N_3O_7$ 446.1922; Found 446.1911.

9. General procedure for the synthesis of pyrazoles 5

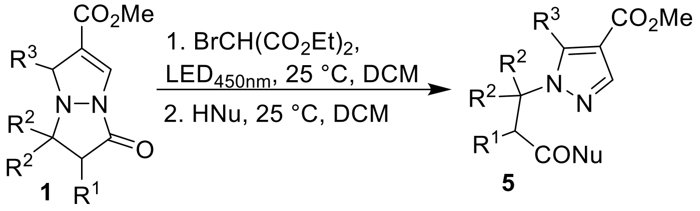

Scheme SI4: Synthesis of compounds **5**.

A dried 8 mL vial was charged with **1** (0.5 mmol) and dry DCM (2.5 mL) as the solvent unless noted otherwise. The vial was sealed off with a screw cap with a septum. The resulting solution was degassed via needle by three freeze-pump-thaw cycles. Diethyl bromomalonate (185 µL, 92% purity, 2.0 equiv) was then added. The solution was irradiated with a blue $LED_{450\,nm}$ under nitrogen atmosphere for 18 h at 25 °C. If necessary, a solution of a nucleophile (2.0 equiv) in dry DCM (1.0 mL) was added and the reaction mixture stirred for an additional 1 h at 25 °C without irradiation. Products **5** were isolated by CC and dried under vacuum unless noted otherwise.

10. Characterization data of novel compounds 5

3-(5-(4-Chlorophenyl)-4-(methoxycarbonyl)-1*H*-pyrazol-1-yl)-3-methylbutanoic acid (**5a**). Prepared according to the general procedure from **1a** (161 mg, 0.5 mmol). Acetone (2.5 mL) with water (90 µL, 10 equiv) is used as the solvent. the reaction mixture was evaporated and product **5a** was purified by extraction in DCM. The crude mixture was extracted with saturated aqueous sodium bicarbonate (20 mL). The aqueous phase was acidified with sodium hydrogen sulphate (1M, 25 mL) and extracted with DCM (2 x 20 mL). The organic phase was dried over anhydrous sodium sulphate, filtrated and the solvent evaporated in vacuo. 140 mg (83% yield); white solid; mp 146–147 °C; ν_{max}/cm^{-1} (ATR) 1704, 1224; δ_H (500 MHz; $CDCl_3$; Me_4Si) 7.95 (s, 1H), 7.43 (d, *J* = 8.0 Hz, 2H), 7.28 (d, *J* = 8.0 Hz, 2H), 3.64 (s, 3H), 2.98 (s, 2H), 1.48 (s, 6H); δ_C (126 MHz; $CDCl_3$; Me_4Si) 174.2, 163.1, 145.0, 139.4, 135.5, 131.5, 129.8, 128.4, 114.9, 63.3, 51.2, 47.5, 29.5; HRMS (ESI) m/z: $[M + H]^+$ Calcd for $C_{16}H_{18}ClN_2O_4$ 337.0950; Found 337.0948.

Methyl 5-(4-chlorophenyl)-1-(4-methoxy-2-methyl-4-oxobutan-2-yl)-1*H*-pyrazole-4-carboxylate (**5b**). Prepared according to the general procedure from **1a** (161 mg, 0.5 mmol). Anhydrous MeOH (2.5 mL) is used as solvent. CC (EA/PE, 1:4); 165 mg (94% yield); white solid; mp 82–84 °C; ν_{max}/cm^{-1} (ATR) 1731, 1708, 1219; δ_H (500 MHz; $CDCl_3$; Me_4Si) 7.91 (s, 1H), 7.45 – 7.40 (m, 2H), 7.33 – 7.29 (m, 2H), 3.63 (s, 3H), 3.60 (s, 3H), 2.87 (s, 2H), 1.49 (s, 6H); δ_C (126 MHz; $CDCl_3$; Me_4Si) 170.3, 163.3, 144.8, 139.4, 135.3, 131.5, 130.3, 128.3, 114.6, 63.1, 51.6, 51.0, 46.7, 29.5; HRMS (ESI) m/z: $[M + H]^+$ Calcd for $C_{17}H_{20}ClN_2O_4$ 351.1106; Found 351.1106.

Methyl 5-(4-chlorophenyl)-1-(2-methyl-4-oxo-4-(p-tolyloxy)butan-2-yl)-1*H*-pyrazole-4-carboxylate (**5c**). Prepared according to the general procedure from **1a** (161 mg, 0.5 mmol). *p*-Cresol (108 mg, 2.0 equiv) is used as the nucleophile. CC (EA/PE, 1:3); 162 mg (76% yield); white solid; mp 102–103 °C; ν_{max}/cm^{-1} (ATR) 1765, 1704, 1126; δ_H (500 MHz; $CDCl_3$; Me_4Si) 7.95 (s, 1H), 7.41 (d, *J* = 8.5 Hz, 2H), 7.31 (d, *J* = 8.5 Hz, 2H), 7.14 (d, *J* = 8.2 Hz, 2H), 6.86 (d, *J* = 8.4 Hz, 2H), 3.62 (s, 3H), 3.12 (s, 2H), 2.32 (s, 3H), 1.56 (s, 6H); δ_C (126 MHz; $CDCl_3$; Me_4Si) 168.7, 163.2, 148.1, 144.9, 139.4, 135.6, 135.3, 131.6, 130.2, 129.9, 128.3, 121.1, 114.7, 63.2, 51.1, 47.0, 29.6, 20.9; HRMS (ESI) m/z: $[M + H]^+$ Calcd for $C_{23}H_{24}ClN_2O_4$ 427.1419; Found 427.1414.

Methyl 5-(4-chlorophenyl)-1-(2-methyl-4-oxo-4-(propylamino)butan-2-yl)-1*H*-pyrazole-4-carboxylate (**5d**) Prepared according to the general procedure from **1a** (161 mg, 0.5 mmol). *n*-Propylamine (83 µL, 2.0 equiv) is used as the nucleophile. CC (EA/PE, 1:1); 121 mg (64% yield); white solid; mp 93–95 °C; ν_{max}/cm^{-1} (ATR) 3304, 2972, 1720, 1642, 1231; δ_H (500 MHz; $CDCl_3$; Me_4Si) 7.98 (s, 1H), 7.43 – 7.37 (m, 2H), 7.25 – 7.19 (m, 2H), 5.98 (s, 1H), 3.62 (s, 3H), 3.09 (dd, *J* = 13.1, 6.9 Hz, 2H), 2.86 (s, 2H), 1.42 (s, 6H), 1.41 – 1.33 (m, 2H), 0.80 (t, *J* = 7.4 Hz, 3H); δ_C (126 MHz; $CDCl_3$; Me_4Si) 169.3, 163.0, 145.5, 139.8, 135.4, 131.4, 130.0, 128.3, 114.8, 64.6, 51.1, 49.6, 41.0, 29.7, 22.7, 11.3; HRMS (ESI) m/z: $[M + H]^+$ Calcd for $C_{19}H_{25}ClN_3O_3$ 378.1579; Found 378.1572.

Methyl 5-(4-chlorophenyl)-1-(2-methyl-4-oxo-4-(p-tolylamino)butan-2-yl)-1*H*-pyrazole-4-carboxylate (**5e**). Prepared according to the general procedure from **1a** (161 mg, 0.5 mmol). *p*-Toluidine (108 mg, 2.0 equiv) is used as the nucleophile. CC (EA/PE, 1:3); 134 mg (63% yield); white solid; mp 94–95 °C; ν_{max}/cm^{-1} (ATR) 2984, 1720, 1651, 1220; δ_H (500 MHz; $CDCl_3$; Me_4Si) 8.09 (s, 1H), 7.93 (s, 1H), 7.38 (d, *J* = 8.0 Hz, 2H), 7.24 (d, *J* = 8.0 Hz, 2H), 7.16 (d, *J* = 8.0 Hz, 2H),

7.08 (d, *J* = 8.0 Hz, 2H), 3.63 (s, 3H), 3.07 (s, 2H), 2.29 (s, 3H), 1.45 (s, 6H); δ_C (126 MHz; $CDCl_3$; Me_4Si) 167.5, 162.9, 145.8, 139.9, 135.5, 135.3, 133.8, 131.4, 129.6, 129.5, 128.4, 119.5, 115.2, 65.0, 51.2, 50.9, 29.7, 20.9; HRMS (ESI) m/z: $[M + H]^+$ Calcd for $C_{23}H_{25}ClN_3O_3$ 426.1579; Found 426.1572.

Methyl (*S*)-5-(4-chlorophenyl)-1-(4-((1-methoxy-1-oxopropan-2-yl)amino)-2-methyl-4-oxobutan-2-yl)-1*H*-pyrazole-4-carboxylate (**5f**). Prepared according to the general procedure from **1a** (161 mg, 0.5 mmol). The solution of the nucleophile was prepared by stirring L-alanine methyl ester hydrochloride (140 mg, 2.0 equiv) with DBU (150 µL, 2.0 equiv) in dry DCM (1 mL) overnight. CC (EA/PE, 1:1); 126 mg (60% yield); colorless oil; $[\alpha]_{589}^{25}$ -26 (*c* 0.17, MeOH); ν_{max}/cm^{-1} (ATR) 3335, 2951, 1718, 1656, 1210, 1158, 729; δ_H (500 MHz; $CDCl_3$; Me_4Si) 7.99 (s, 1H), 7.41 (d, *J* = 8.7 Hz, 2H), 7.32 – 7.28 (m, 1H), 7.26 – 7.22 (m, 1H), 6.52 (d, *J* = 7.4 Hz, 1H), 4.47 (p, *J* = 7.2 Hz, 1H), 3.70 (s, 3H), 3.63 (s, 3H), 3.00 (d, *J* = 13.7 Hz, 1H), 2.85 (d, *J* = 13.7 Hz, 1H), 1.43 (s, 6H), 1.28 (d, *J* = 7.2 Hz, 3H); δ_C (126 MHz; $CDCl_3$; Me_4Si) 173.0, 168.9, 163.1, 145.4, 139.7, 135.3, 131.6, 131.4, 130.1, 128.3, 128.3, 114.8, 64.4, 52.3, 51.09, 49.1, 47.8, 29.8, 29.4, 18.1; HRMS (ESI) m/z: $[M + H]^+$ Calcd for $C_{20}H_{25}ClN_3O_5$ 422.1477; Found 422.1473.

3-(4-(Methoxycarbonyl)-5-phenyl-1*H*-pyrazol-1-yl)propanoic acid (**5g**). Prepared according to the general procedure from **1g** (129 mg, 0.5 mmol). Acetone (2.5 mL) with water (90 µL, 10 equiv) was used as the solvent. Reaction mixture was evaporated and product **5g** was purified by extraction whit DCM (20 mL) and washed with saturated aqueous sodium bicarbonate (20 mL). The aqueous phase was acidified with sodium hydrogen sulphate (1M, 25 mL) and extracted with DCM (2 x 20 mL). The organic phase was dried over anhydrous sodium sulphate, filtrated and the solvent evaporated in vacuo. 99 mg (72% yield); white solid; mp 111–112 °C; ν_{max}/cm^{-1} (ATR) 2950, 1718, 1189; δ_H (500 MHz; $CDCl_3$; Me_4Si) 9.05 (bs, 1H), 8.01 (s, 1H), 7.51 – 7.45 (m, 3H), 7.38 – 7.33 (m, 2H), 4.24 (t, *J* = 6.8 Hz, 2H), 3.68 (s, 3H), 2.88 (t, *J* = 6.8 Hz, 2H); δ_C (126 MHz; $CDCl_3$; Me_4Si) 175.3, 163.3, 146.5, 141.5, 129.8, 129.6, 128.5, 128.4, 112.6, 51.2, 44.6, 34.1; HRMS (ESI) m/z: $[M + H]^+$ Calcd for $C_{14}H_{15}N_2O_4$ 275.1026; Found 275.1023.

Methyl 1-(3-methoxy-3-oxopropyl)-5-phenyl-1*H*-pyrazole-4-carboxylate (**5h**). Prepared according to the general procedure from **1g** (129 mg, 0.5 mmol). Anhydrous MeOH (2.5 mL) was used as the solvent. CC (EA/PE, 1:2); 133 mg (92% yield); colorless oil; ν_{max}/cm^{-1} (ATR) 2952, 1712, 1203; δ_H (500 MHz; $CDCl_3$; Me_4Si) 7.99 (s, *J* = 7.2 Hz, 1H), 7.53 – 7.44 (m, 3H), 7.42 – 7.35 (m, 2H), 4.24 (t, *J* = 7.1 Hz, 2H), 3.67 (s, 3H), 3.63 (s, 3H), 2.87 (t, *J* = 7.1 Hz, 2H); δ_C (126 MHz; $CDCl_3$; Me_4Si) 170.9, 163.3, 146.3, 141.5, 129.9, 129.5, 128.7, 128.4, 112.5, 51.9, 51.1, 44.8, 33.9; HRMS (ESI) m/z: $[M + H]^+$ Calcd for $C_{15}H_{17}N_2O_4$ 289.1183; Found 289.1174.

(*RS*)-2-(((Benzyloxy)carbonyl)amino)-3-(4-(methoxycarbonyl)-5-phenyl-1*H*-pyrazol-1-yl)-3-methylbutanoic acid (**5i**). Prepared according to the general procedure from **1o** (218 mg, 0.5 mmol). Acetone (2.5 mL) with water (90 µL, 10 equiv) was used as the solvent. Reaction mixture was evaporated and product **5i** purified by extraction with DCM (20 mL). The crude mixture was extracted with saturated aqueous sodium bicarbonate (20 mL). The aqueous phase was acidified with sodium hydrogen sulphate (1M, 25 mL) and extracted with DCM (2 x 20 mL). The organic phase was dried over anhydrous sodium sulphate, filtrated and the solvent evaporated in vacuo. 221 mg (98% yield); white solid; mp 138–139 °C; ν_{max}/cm^{-1} (ATR) 3401, 2943, 1721, 1514, 697; δ_H (500 MHz; $CDCl_3$; Me_4Si) 10.38 (bs, 1H), 7.87 (d, *J* = 4.0 Hz,

1H), 7.51 – 7.18 (m, 10H), 6.30 (d, *J* = 9.8 Hz, 1H), 5.13 (d, *J* = 12.2 Hz, 1H), 5.08 (d, *J* = 12.2 Hz, 1H), 4.70 (d, *J* = 9.8 Hz, 1H), 3.58 (s, 3H), 1.48 (s, 3H), 1.46 (s, 3H); δ_C (126 MHz; $CDCl_3$; Me_4Si) 173.8, 163.2, 156.6, 146.6, 139.7, 136.0, 130.9, 130.3, 129.8, 129.3, 128.5, 128.2, 128.1, 128.0, 128.0, 114.7, 67.4, 67.0, 62.8, 51.1, 27.8, 27.3; HRMS (ESI) m/z: $[M + H]^+$ Calcd for $C_{24}H_{26}N_3O_6$ 452.1816; Found 452.1818.

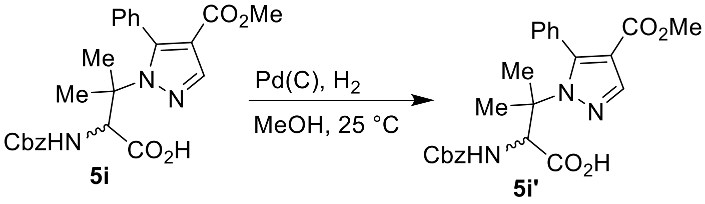

Scheme SI5: Deprotection of compound **5i**.

(*RS*)-2-Amino-3-(4-(methoxycarbonyl)-5-phenyl-1*H*-pyrazol-1-yl)-3-methylbutanoic acid (**5i'**). **5i** (180 mg, 0.4 mmol) was dissolved in MeOH (10 mL). Palladium on carbon (10%, 40 mg) was added and the suspension was hydrogenated at 2.0 bar at 25 °C for 6 h. The reaction mixture was then filtered through a pad of silica and washed with MeOH (10 mL). After evaporation of the solvent, product **5i'** can be further purified by CC (DCM/MeOH, 8:1) to give a white powder. 120 mg (95% yield); white solid; mp 166–167 °C; ν_{max}/cm^{-1} (ATR) 3115, 2989, 2950, 1721, 1618, 1547; δ_H (500 MHz; CD_3OD; Me_4Si) 7.98 (s, 1H), 7.50 – 7.39 (m, 4H), 7.35 – 7.29 (m, 1H), 4.12 (bs, 1H), 3.54 (s, 3H), 3.34 (s, 1H), 1.60 (s, 3H), 1.38 (s, 3H); δ_C (126 MHz; $CDCl_3$; Me_4Si) 172.4, 165.1, 148.6, 141.7, 133.0, 132.3, 131.3, 130.5, 129.3, 116.3, 67.4, 64.9, 51.8, 50.2, 29.6, 25.5; HRMS (ESI) m/z: $[M + H]^+$ Calcd for $C_{16}H_{20}N_3O_4$ 318.1448; Found 318.1448.

Methyl (*RS*)-1-(3-(((benzyloxy)carbonyl)amino)-4-methoxy-2-methyl-4-oxobutan-2-yl)-5-phenyl-1*H*-pyrazole-4-carboxylate (**5j**). Prepared according to the general procedure from **1o** (218 mg, 0.5 mmol). Anhydrous MeOH (2.5 mL) was used as the solvent. CC (EA/PE, 1:4); 175 mg (75% yield); white solid; mp 132–133 °C; ν_{max}/cm^{-1} (ATR) 3307, 1738, 1719, 1151; δ_H (500 MHz; $CDCl_3$; Me_4Si) 7.86 (s, 1H), 7.50 – 7.27 (m, 10H), 6.19 (d, *J* = 9.8 Hz, 1H), 5.11 (s, 2H), 4.65 (d, *J* = 9.9 Hz, 1H), 3.63 (s, 3H), 3.60 (s, 3H), 1.50 (s, 3H), 1.45 (s, 3H); δ_C (126 MHz; $CDCl_3$; Me_4Si) 170.1, 163.2, 156.3, 146.7, 139.5, 136.1, 131.2, 130.2, 129.9, 129.3, 128.6, 128.3, 128.1, 128.0, 127.97, 114.6, 67.2, 66.8, 62.7, 52.6, 51.0, 27.4, 27.2; HRMS (ESI) m/z: $[M + H]^+$ Calcd for $C_{25}H_{28}N_3O_6$ 466.1973; Found 466.1965.

Methyl 5-(furan-2-yl)-1-(4-methoxy-2-methyl-4-oxobutan-2-yl)-1*H*-pyrazole-4-carboxylate (**5k**). Prepared according to the general procedure from **1e** (138 mg, 0.5 mmol). Anhydrous MeOH (2.5 mL) was used as the solvent. CC (EA/PE, 1:4); 80 mg (52% yield); pale yellow oil; ν_{max}/cm^{-1} (ATR) 2952, 1722, 1213, 1145; δ_H (500 MHz; $CDCl_3$; Me_4Si) 7.92 (s, 1H), 7.63 – 7.60 (m, 1H), 6.61 – 6.59 (m, 1H), 6.56 (dd, *J* = 3.3, 1.9 Hz, 1H), 3.70 (s, 3H), 3.59 (s, 3H), 2.90 (s, 2H), 1.55 (s, 6H); δ_C (126 MHz; $CDCl_3$; Me_4Si) 170.3, 163.0, 143.1, 141.1, 139.2, 134.8, 116.6, 114.0, 111.4, 62.8, 51.6, 51.3, 46.4, 28.1; HRMS (ESI) m/z: $[M + H]^+$ Calcd for $C_{15}H_{19}N_2O_5$ 307.1288; Found 307.1284.

Methyl 1-(3-(4-bromobutoxy)-3-oxopropyl)-5-phenyl-1*H*-pyrazole-4-carboxylate (**5l**). Prepared according to the general procedure from **1g** (129 mg, 0.5 mmol). Anhydrous THF (2.5 mL) was used as the solvent. CC (EA/PE, 2:5); 137 mg (67% yield); colorless oil; ν_{max}/cm^{-1}

(ATR) 2951, 1716, 1552; δ_H (500 MHz; $CDCl_3$; Me_4Si) 7.99 (s, 1H), 7.53 – 7.45 (m, 3H), 7.42 – 7.35 (m, 2H), 4.24 (t, *J* = 7.0 Hz, 2H), 4.07 (t, *J* = 6.3 Hz, 2H), 3.67 (s, 3H), 3.39 (t, *J* = 6.6 Hz, 2H), 2.86 (t, *J* = 7.0 Hz, 2H), 1.91 – 1.82 (m, 2H), 1.78 – 1.69 (m, 2H); δ_C (126 MHz; $CDCl_3$; Me_4Si) 170.5, 163.3, 146.2, 141.4, 129.9, 129.5, 128.6, 128.4, 112.5, 63.8, 51.1, 44.9, 34.0, 33.0, 29.1, 27.1; HRMS (ESI) m/z: $[M + H]^+$ Calcd for $C_{18}H_{22}BrN_2O_4$ 409.0757; Found 409.0751.

Methyl 1-(4-(4-bromobutoxy)-2-methyl-4-oxobutan-2-yl)-5-(4-chlorophenyl)-1*H*-pyrazole-4-carboxylate (**5m**). Prepared according to the general procedure from **1a** (161 mg, 0.5 mmol). Anhydrous THF (2.5 mL) was used as the solvent. CC (EA/PE, 1:4); 122 mg (52% yield); colorless oil; ν_{max}/cm^{-1} (ATR) 2950, 1719, 1547; δ_H (500 MHz; $CDCl_3$; Me_4Si) 7.90 (s, 1H), 7.43 (d, *J* = 8.5 Hz, 2H), 7.31 (d, *J* = 8.5 Hz, 2H), 4.03 (t, *J* = 6.3 Hz, 2H), 3.63 (s, 3H), 3.40 (t, *J* = 6.6 Hz, 2H), 2.87 (s, 2H), 1.91 – 1.82 (m, 2H), 1.76 – 1.68 (m, 2H), 1.49 (s, 6H); δ_C (126 MHz; $CDCl_3$; Me_4Si) 169.8, 163.2, 144.8, 139.3, 135.3, 131.6, 130.2, 128.3, 114.7, 63.5, 63.1, 51.1, 46.9, 33.0, 29.5, 29.2, 27.2; HRMS (ESI) m/z: $[M + H]^+$ Calcd for $C_{20}H_{25}BrClN_2O_4$ 471.0681; Found 471.0672.

11. Gram-scale synthesis of 4a

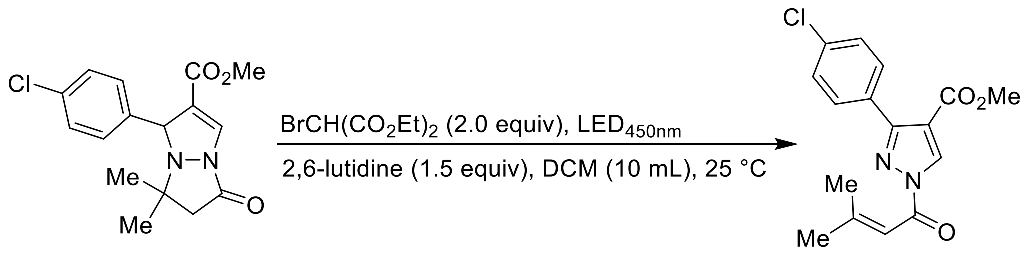

1a (3.0 mmol) **4a** (80%)

Scheme SI6: Synthesis of compound **4a** on a gram-scale.

Modified general procedure was applied on a gram-scale synthesis of **4a**. A dried 25 mL glass tube was charged with **1a** (962 mg, 3.0 mmol), diethyl bromomalonate (1.11 mL, 92% purity, 2.0 equiv), 2,6-lutidine (522 µL, 1.5 equiv) and dry DCM (10 mL) and sealed off with a septum. The resulting solution was degassed via needle by three freeze-pump-thaw cycles and irradiated with a blue $LED_{450\ nm}$ under nitrogen atmosphere for 8 days at room temperature. The product **4a** was purified by CC (EA/PE, 1:6) and isolated as a white solid (764 mg, 80%). For characterization see section 8.

12. Mechanistic studies

12.1 Synthesis of deuterated analogue **2D**

Deuterated substrate **1D** was prepared according to a slightly modified general procedure in order to determine the origin of the aldehyde proton in compounds **2**. After irradiation, **2D** was isolated in a high yield with unchanged isotopic purity.

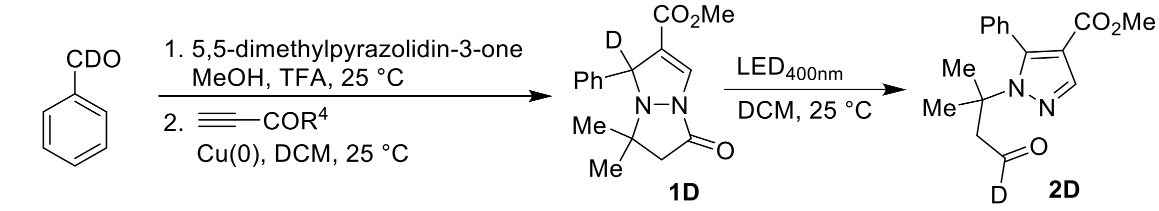

> 98 atom% D   > 98 atom% D   > 98 atom% D

Scheme SI7: Synthesis of deuterated compounds **1D** and **2D**.

Methyl 7,7-dimethyl-5-oxo-1-phenyl-6,7-dihydro-1*H*,5*H*-pyrazolo[1,2-*a*]pyrazole-2-carboxylate-1-*d* (**1D**). Prepared by stirring a solution of 5,5-dimethylpyrazolidin-3-one (342 mg, 3.0 mmol), benzaldehyde-α-d_1 (337 mg, 3.15 mmol, 98 atom% D) and TFA (10 µL) in MeOH (6 mL) at 25 °C for 24 h. After evaporating the volatiles, copper powder (120 mg), CH_2Cl_2 (15 mL) and methyl propiolate (321 µL, 3.6 mmol) were added and the mixture was stirred at 25 °C for 24 h. The catalyst and impurities were removed by CC on silica (DCM/MeOH, 40:1) and the filtrate evaporated in vacuo to give **1D** as yellow crystals. 517 mg (60% yield); yellow solid; mp 153–154 °C; ν_{max}/cm^{-1} (ATR) 3074, 1787, 1735, 1688, 1595; δ_H (500 MHz; $CDCl_3$; Me_4Si) 7.52 (s, 1H), 7.46 – 7.42 (m, 2H), 7.38 – 7.32 (m, 2H), 7.31 – 7.27 (m, 1H), 3.62 (s, 3H), 2.87 (d, *J* = 15.7 Hz, 1H), 2.39 (d, *J* = 15.7 Hz, 1H), 1.24 (s, 3H), 1.15 (s, 3H); δ_C (126 MHz; $CDCl_3$; Me_4Si) 166.6, 164.1, 141.9, 129.5, 128.4, 127.9, 127.8, 116.7, 64.5, 64.2 (J_{CD} = 21.0 Hz)51.5, 49.4, 24.9, 19.0; HRMS (ESI) m/z: [M + H]$^+$ Calcd for $C_{16}H_{18}DN_2O_3$ 288.1453; Found 288.1454. According to ^{1}H NMR and HRMS, isotopic purity is estimated to be > 98 atom% D.

Methyl 1-(2-methyl-4-oxobutan-2-yl-4-*d*)-5-phenyl-1*H*-pyrazole-4-carboxylate (**2D**) Prepared according to the general procedure from **1D** (144 mg, 0.5 mmol); CC(EA/PE, 1:3); 123 mg (86% yield); white solid; mp 97–98 °C; ν_{max}/cm^{-1} (ATR) 2977, 2125, 1716, 1701, 1545; δ_H (500 MHz; $CDCl_3$; Me_4Si) 7.91 (s, 1H), 7.52 – 7.40 (m, 3H), 7.37 – 7.29 (m, 2H), 3.61 (s, 3H), 2.91 (s, 2H), 1.47 (s, 6H); δ_C (126 MHz; $CDCl_3$; Me_4Si) 199.2 (J_{CD} = 27.0 Hz), 163.2, 145.9, 139.3, 131.3, 130.2, 129.2, 128.1, 114.9, 63.3, 54.9 (J_{CD} = 3.5 Hz), 51.0, 29.5; HRMS (ESI) m/z: [M + H]$^+$ Calcd for $C_{16}H_{18}DN_2O_3$ 288.1453; Found 288.1453. According to ^{1}H NMR and HRMS analysis, isotopic purity was estimated to be > 98 atom% D.

An additional experiment including a mixture of **1D** and **1d** showed no intermolecular proton/deuterium exchange and both **2D** and **2d** were isolated as isotopically pure (> 98 atom%) substances. Carried out according to the general procedure from **1D** (14.4 mg, 0.1 mmol) and **1d** (15.6 mg, 0.1 mmol); CC (EA/PE, 1:2); 12.4 mg (86% yield) **2D**; 13.3 mg (85% yield) **2d**.

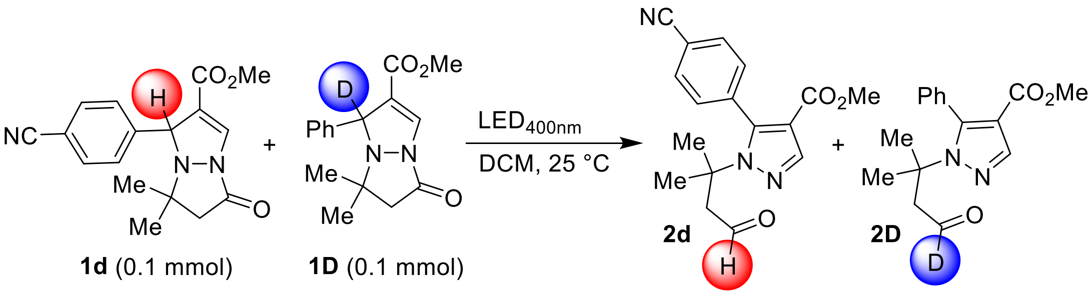

Scheme SI8: Irradiation of a mixture of compounds **1d** and **1D**.

12.2 Reaction monitoring and determination of the triplet state

Transformation from **1a** to **2a** was also monitored by ^{1}H NMR spectroscopy. After following the general procedure with **1a** (9.6 mg, 0.03 mmol) and CD_2Cl_2 (1.0 mL), the reaction mixture was transferred to an NMR tube under argon and irradiated with LED_{400nm}. Progress of the reaction was monitored every hour. A parallel reaction mixture was prepared by adding *trans*-stilbene (5.4 mg, 1.0 equiv). Significant inhibition of the reaction was observed, together with the conversion of *trans* to *cis*-stilbene, which implies the energy transfer took place between the triplet excited state of **1a** and *trans*-stilbene (Figure SI1).[14]

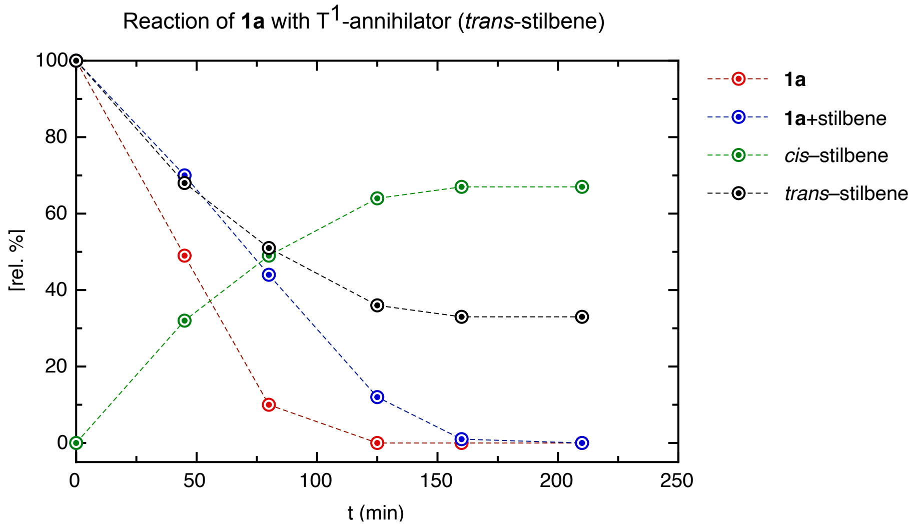

Figure SI1: Reaction progress: (a) **1a** in DCM over time (•), (b) **1a** with *trans*-stilbene in DCM over time (•), *trans*- to *cis*-stilbene conversion over time (• and •).

12.3 Light-dark reaction

To show that the reaction only takes place during irradiation, a reaction following the general procedure with **1a** (32.1 mg, 0.1 mmol) and DCM (2.5 mL) was carried out. It was consecutively either irradiated with LED_{450nm} or kept in darkness. The samples taken at regular intervals were analyzed by ^{1}H NMR spectroscopy to determine the NMR yields of **1a**. The reaction took place only during irradiation periods.

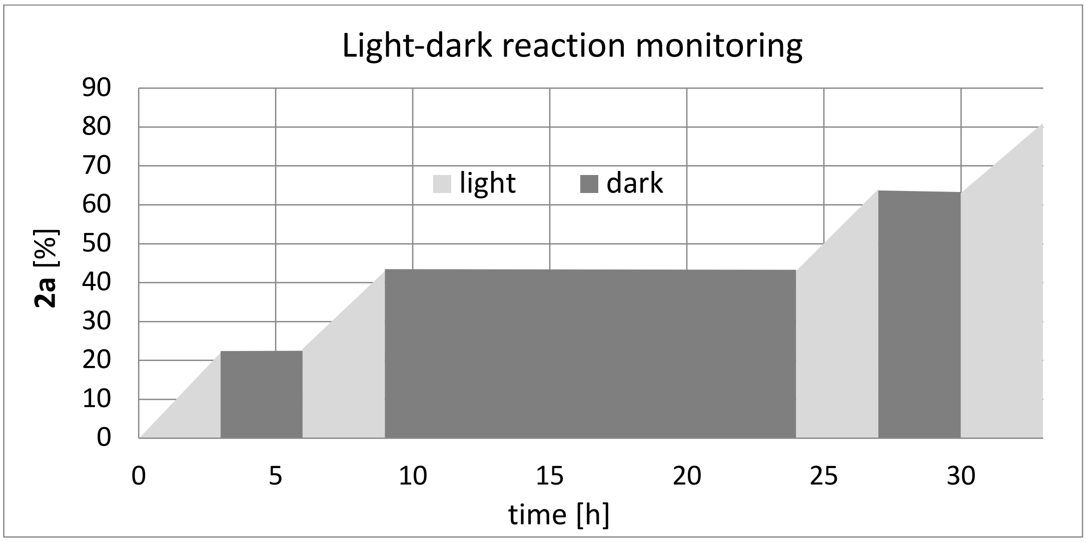

Figure SI2: Light-dark experiment for the formation of **2a**.

12.4 Proposed mechanism for the formation of **3i**

We propose the following reaction pathway for the formation of ring-expansion products **3** commenced via C1–N8 homolytic bond cleavage followed by radical 7-endo-trig cyclization.

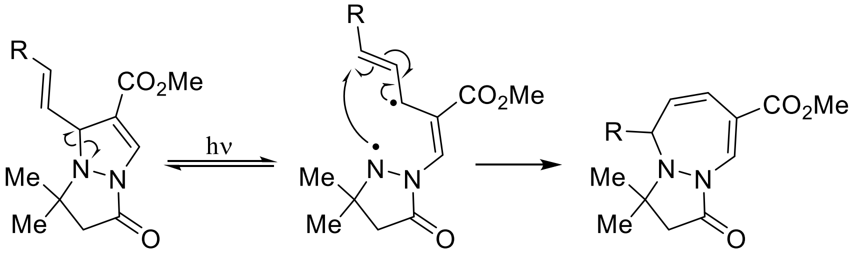

Scheme SI9: Proposed reaction pathway for the formation of compounds **3**.

12.5 Intermediate **I2** detection

Intermediate **I2** was successfully characterized by NMR spectroscopy. A dry 8 mL vial was charged with **1a** (32.1 mg, 0.1 mmol) and CD_3CN (1.0 mL). The vial was sealed off with a screw cap with a septum. The resulting solution was degassed via needle by three freeze-pump-thaw cycles. Diethyl bromomalonate (18.5 µL, 92% purity, 1.0 equiv) was then added. The solution was irradiated with a blue $LED_{450\,nm}$ under nitrogen atmosphere for 18 h at 25 °C. The reaction mixture was then transferred to an NMR tube under argon. ^{1}H NMR revealed a new compound, which corresponds to intermediate **I2**. As the sample was then gradually exposed to moisture, the intensity of signals **I2** decreased to which carboxylic acid **5a** was formed.

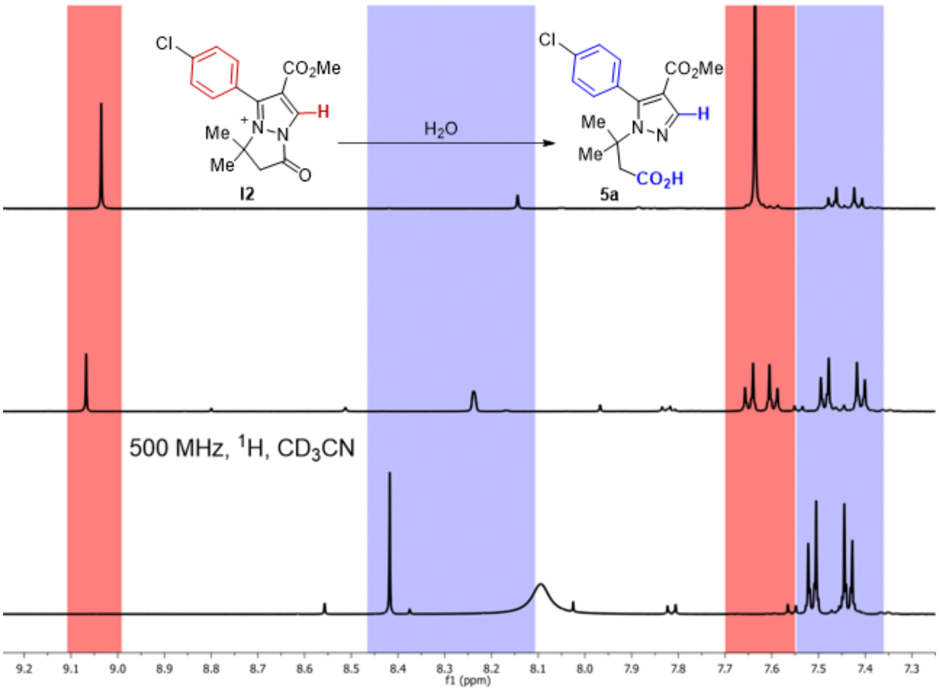

Figure SI3: ^1H NMR spectrum of intermediate **I2** and its gradual conversion to pyrazole **5a**.

12.6 TEMPO reaction

A dry 8 mL vial was charged with **1a** (161 mg, 0.5 mmol), TEMPO (313 mg, 4 equiv) and dry DCM (2.5 mL) and sealed off with a screw cap with a septum. The resulting solution was degassed via needle by three freeze-pump-thaw cycles. Diethyl bromomalonate (185 µL, 92% purity, 2.0 equiv) and 2,6-lutidine (87 µL, 1.5 equiv) were added. The solution was irradiated from below with blue $LED_{450\ nm}$ under nitrogen atmosphere for 18 h at 25 °C. HRMS of the reaction mixture confirmed the presence of a TEMPO-malonate adduct (m/z = 316.2115), which forms from a malonyl radical.

Compound Label	*m/z*	RT	Algorithm	Mass
Cpd 2: C16 H29 N O5	316.2115	0.247	Find by Molecular Feature	315.2042

Compound Identification Results

Ion Mass	Calc Ion Mass	Difference	IonFormula	IonSpecies	Best
316.2115	316.2118	0.4	C16 H30 N O5	(M+H)+	✓
316.2115	316.2118	0.4	C15 H24 N8	(M+H)+	
316.2115	316.2105	-1	C14 H28 N4 O4	(M+H)+	

MS Spectrum

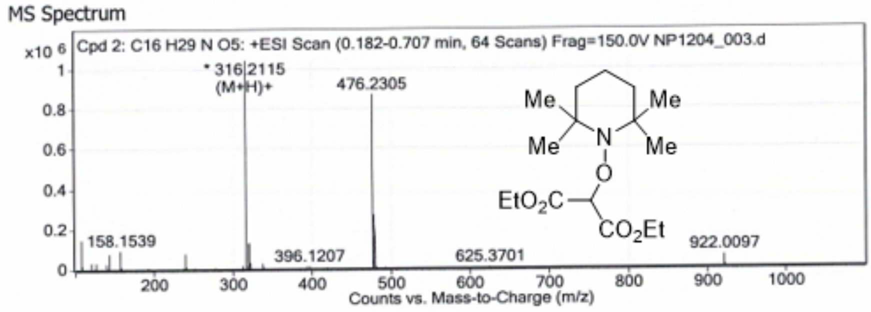

Figure SI4: MS spectrum of a reaction mixture with the TEMPO-malonate adduct.

13. Absorption and emission measurements

Emission spectra of LED were measured and determined to peak at 450 – 455 nm for blue and 510 – 515 nm for green LED.

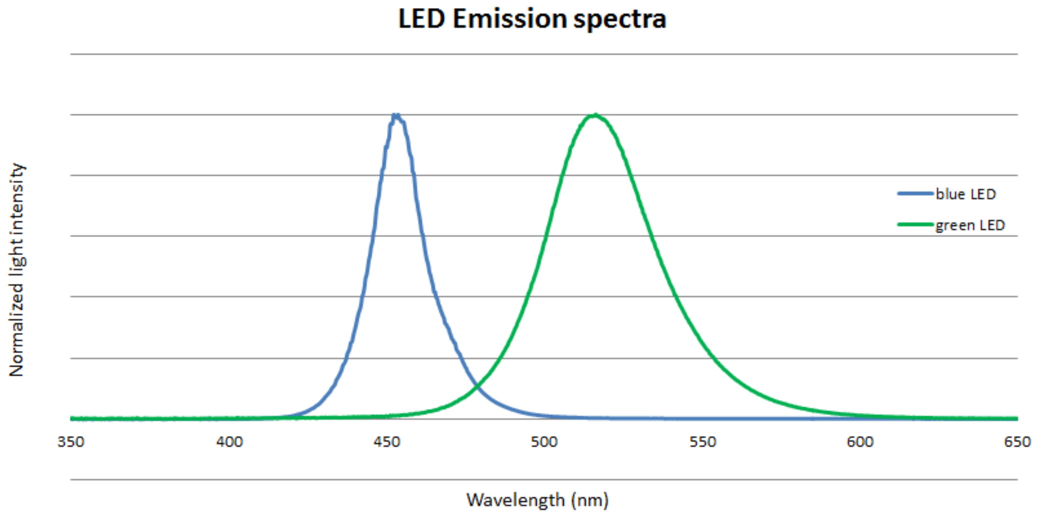

Figure SI5: Emission spectra of blue and green LED.

Absorption spectra of 3 µM solutions of **1** in DCM were measured. The absorption profile that trails into the visible region (around 400 nm) differs little between compounds. The extinction coefficient for compound **1a** was also measured in MeCN (ε = 8400 $M^{-1}cm^{-1}$).

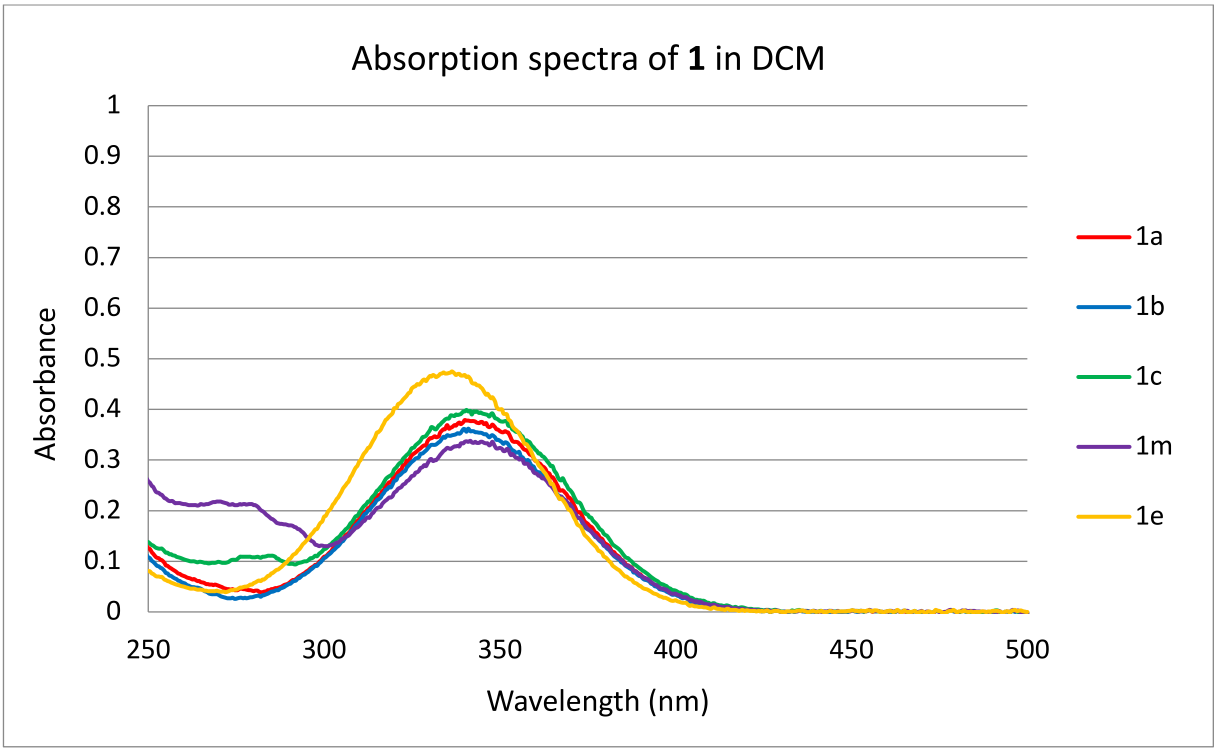

Figure SI6: Absorption spectra of compounds **1**.

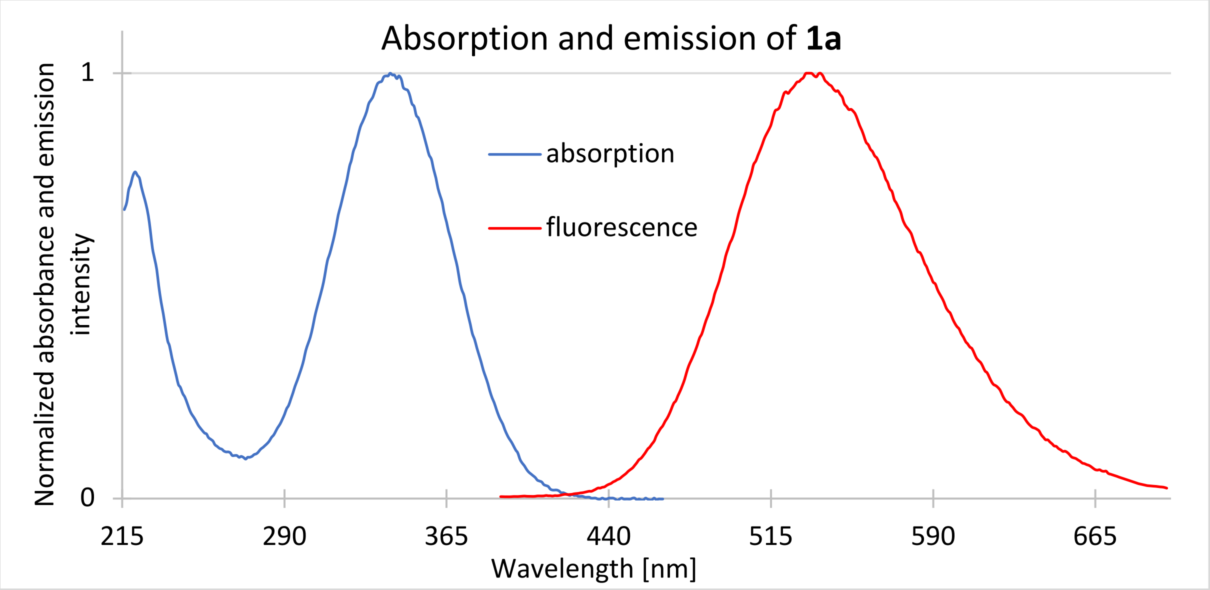

Figure SI7: Absorption and emission spectra of compound **1a**.

Fluorescence quantum yield of compound **1a** in MeCN was determined from the ratio of integrated fluorescence intensity and absorbance.[15] 9-Fluorenone was chosen as a reference with fluorescence quantum yield of 2.7 % in MeCN.[16]

Φ_f = 2.7 % × 2.48 / 0.59 = 11.3 %

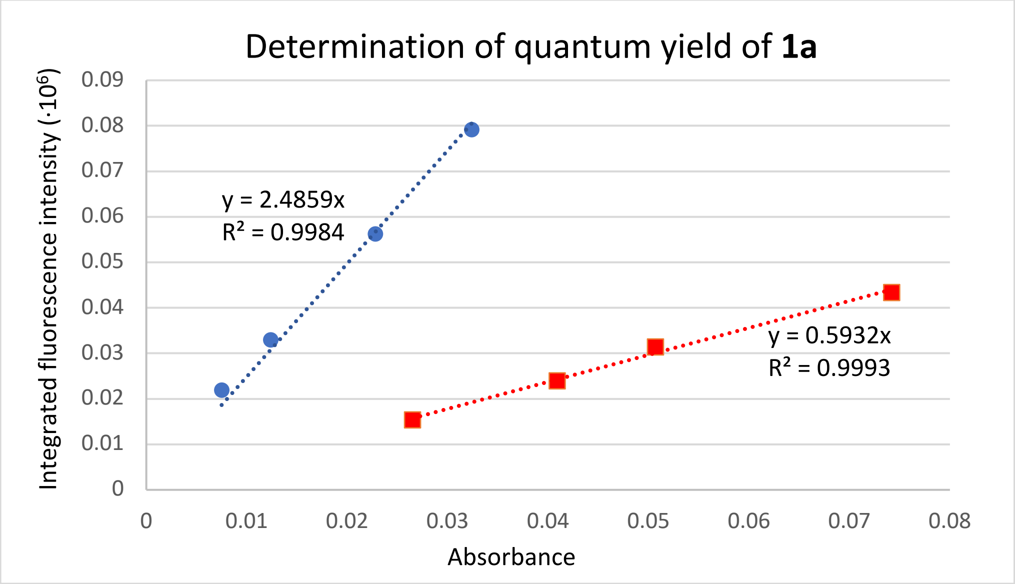

Figure SI8: Plot of integrated fluorescence intensity against absorbance for 9-fluorenone and compound **1a** (● 1a, ■ fluorenone).

14. Stern–Volmer quenching experiments

A 20 µM solution of **1a** in MeCN was degassed and fluorescence of the solution measured under nitrogen atmosphere at different concentrations of diethyl bromomalonate. Fluorescence intensity decreased with increased concentration of diethyl malonate in the solution, which indicates its interaction with **1a***.

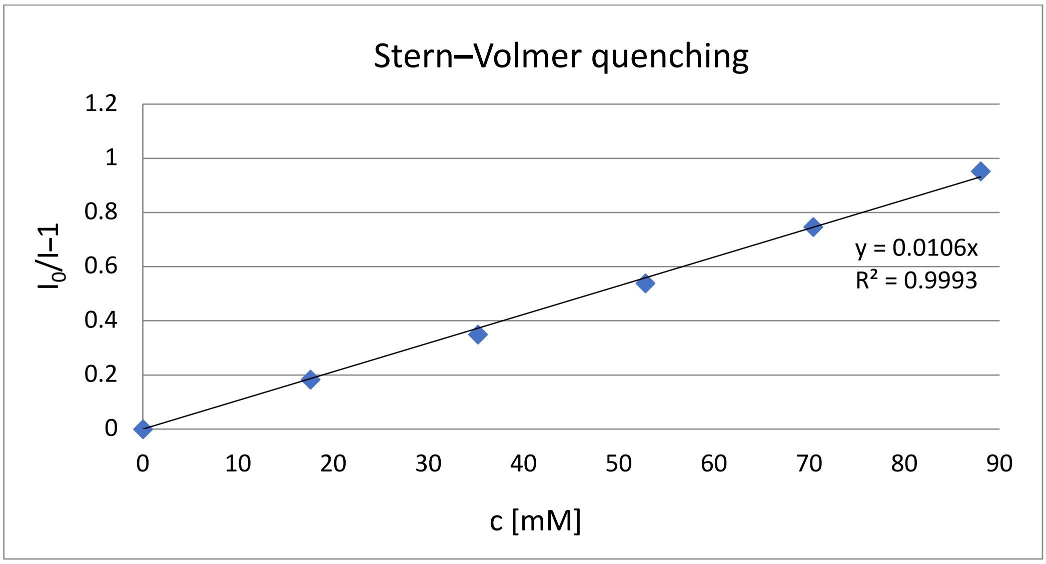

Figure SI9: Stern–Volmer plot of **1a** quenching by diethyl bromomalonate.

15. Cyclic Voltammograms

A cyclic voltammogram of compound **1a** was recorded. A solution of **1a** (10 mM) was prepared in MeCN with Bu_4NBF_4 (50 mM) as an electrolyte. Ferrocene was added as an internal standard. Applied potential was increased from 0 V to 2 V and back to 0 V with a scan rate of 10 mV/s.

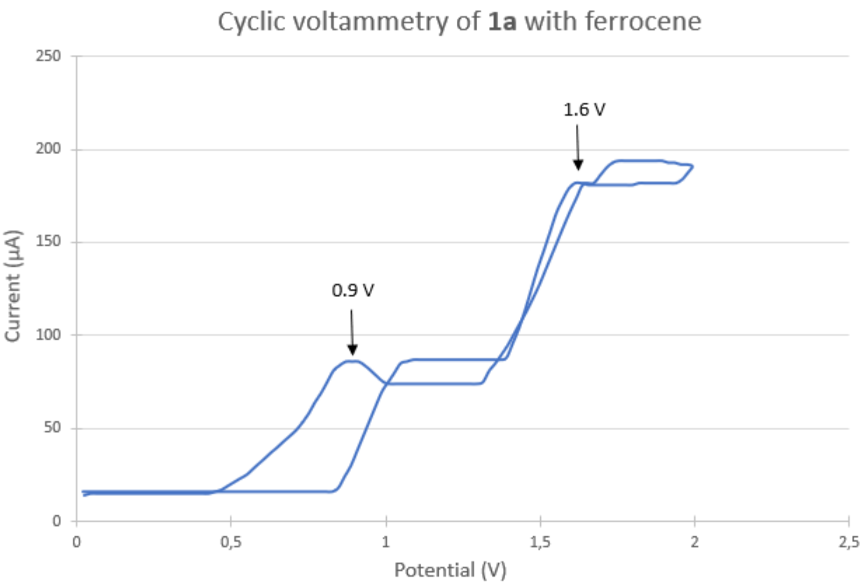

Figure SI10: CV plot of compound **1a** with ferrocene.

$E_{1/2}$ (**Fc⁺/Fc**) = 0.4 V (vs. SCE)[17]

$E_{1/2}$ (**1a**$^{\cdot+}$/**1a**) = 1.1 V (vs. SCE)

From the absorption and emission spectra, we can write:
$E_{0,0}$* = 423 nm = 2.93 eV

$E_{1/2}$ (**1a**$^{\cdot+}$/**1a***) = 1.1 V – 2.9 V (vs. SCE) = –1.8 V (vs. SCE)

16. X-ray crystallography data

Crystals were prepared by slow evaporation of DCM solutions at room temperature. Single-crystal X-ray diffraction data was collected on Agilent Technologies SuperNova Dual diffractometer with an Atlas detector using monochromated Mo-K_{α} radiation (λ = 0.71073 Å) at 293 K. The data was processed using CrysAlis PRO[18]. Using Olex2.1.2.[19], the structures were solved by direct methods implemented in SHELXS[20] or SHELXT[21] and refined by a full-matrix least-squares procedure based on F^2 with SHELXT-2014/7[22]. All nonhydrogen atoms were refined anisotropicallly. Hydrogen atoms were placed in geometrically calculated positions and were refined using a riding model. The drawings and the analysis of bond lengths, angles and intermolecular interactions were carried out using Mercury[23] and Platon[24].

Table SI3: Crystal data and structure refinement for compounds **2c**, **3i** and **4b**.

Compound	**2c**	**3i**	**4b**
Empirical formula	$C_{17}H_{20}N_2O_4$	$C_{18}H_{20}N_2O_3$	$C_{16}H_{16}N_2O_3$
Formula weight	316.35	312.36	284.31
Temperature/K	150.00(10)	150.00(10)	293(2)
Crystal system	triclinic	monoclinic	monoclinic
Space group	P-1	$P2_1/n$	$P2_1/n$
a [Å^3]	9.4508(7)	9.3608(4)	12.3566(6)
b [Å^3]	10.0870(8)	14.3093(5)	9.3671(4)
c [Å^3]	10.1375(7)	12.5589(5)	12.8560(6)
α [°]	62.823(7)	90	90
β [°]	70.139(7)	105.553(4)	106.093(5)
γ [°]	76.003(6)	90	90
V [Å^3]	804.23(12)	1620.62(11)	1429.71(12)
Z	2	4	4
ρ_{calc} [g/cm^3]	1.306	1.280	1.321
μ [mm^{-1}]	0.094	0.088	0.093
F(000)	336.0	664.0	600.0
Crystal size/mm^3	0.5 × 0.4 × 0.2	0.4 × 0.3 × 0.2	0.5 × 0.4 × 0.2
Radiation	MoKα (λ = 0.71073)	MoKα (λ = 0.71073)	MoKα (λ = 0.71073)
Reflections collected	7562	15235	12418
Independent reflections	4148	4401	3848
R_{int}	0.0293	0.0323	0.0297
Data/restraints/parameters	4148/0/213	4401/0/212	3848/0/194
GOF	1.064	1.021	1.027
R_1, wR_2 [I>=2σ (I)]	0.0489, 0.1056	0.0431, 0.1005	0.0416, 0.1023
R_1, wR_2 (all data)	0.0726, 0.1265	0.0595, 0.1106	0.0553, 0.1125
$(\Delta\rho)_{max}$ [e Å^{-3}]	0.27	0.29	0.30
$(\Delta\rho)_{min}$ [e Å^{-3}]	-0.22	-0.21	-0.21

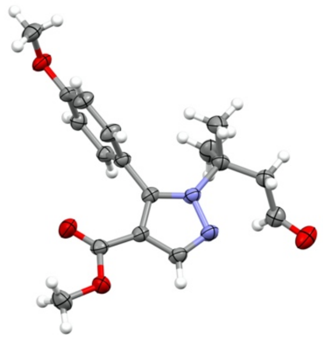

Figure SI11: Molecular structure of product **2c**. Thermal ellipsoids are shown at 50% probability. **CCDC number: 2077376**

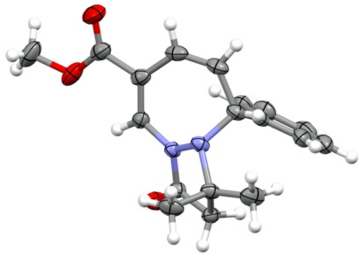

Figure SI12: Molecular structure of product **3i**. Thermal ellipsoids are shown at 50% probability. **CCDC number: 2077377**

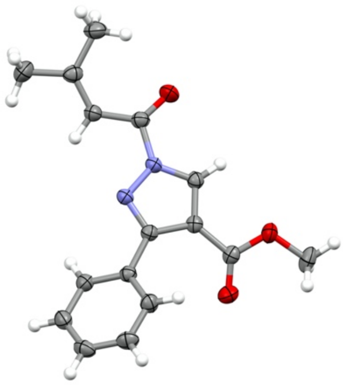

Figure SI13: Molecular structure of product **4b**. Thermal ellipsoids are shown at 50% probability. **CCDC number: 2077375**

17. NMR spectra

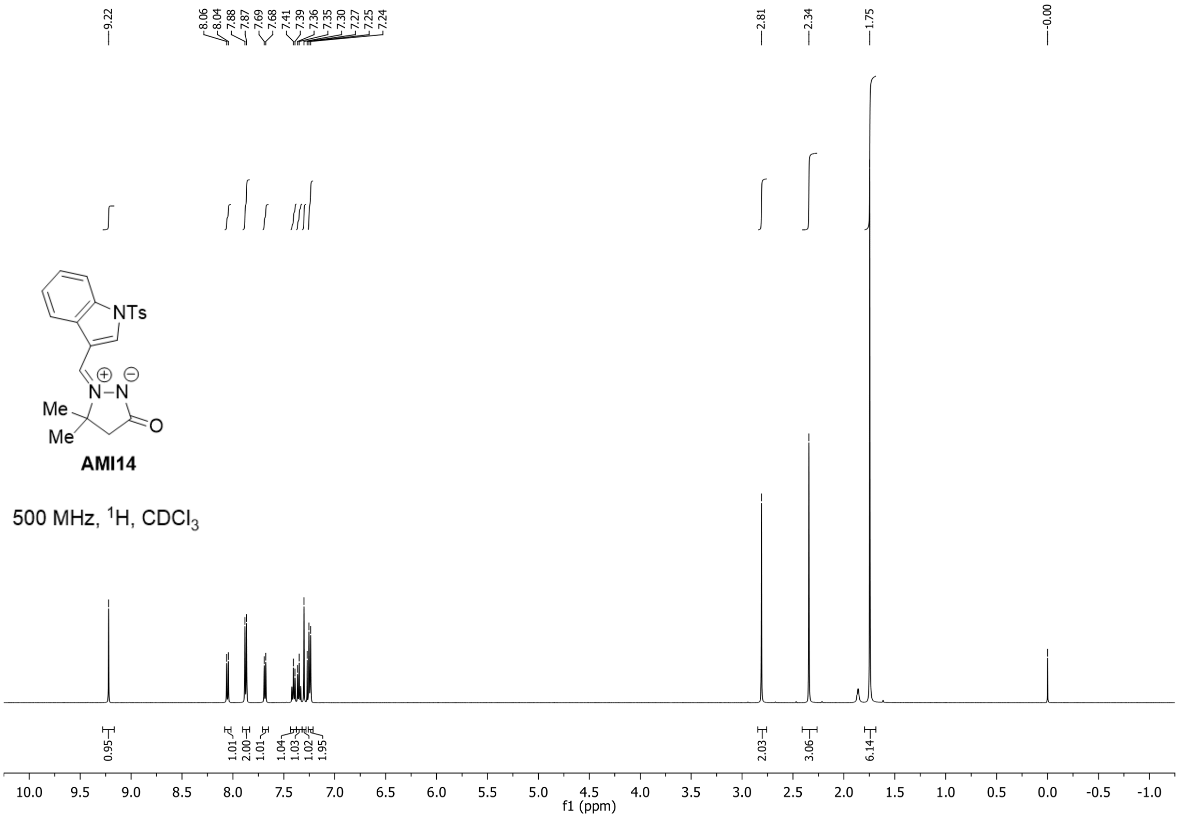

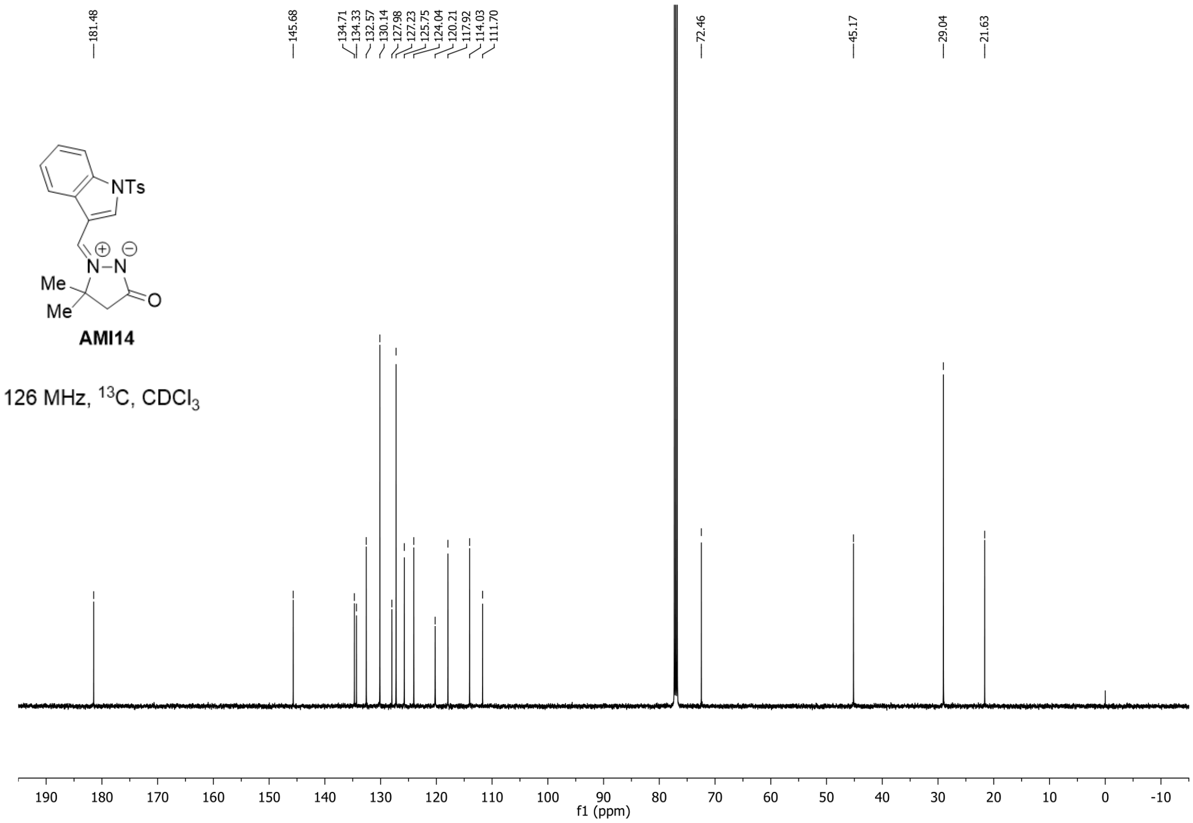

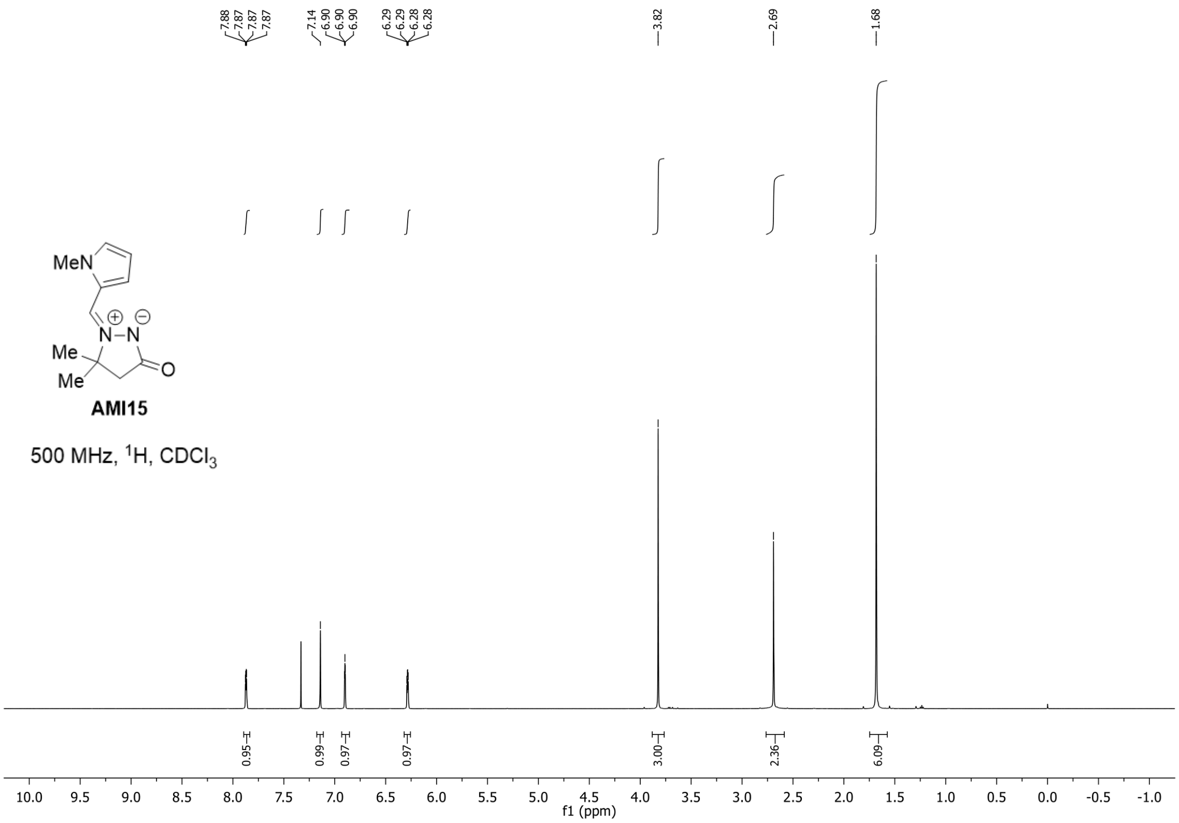

MeN
Me
Me
O
AMI15
500 MHz, ^{1}H, $CDCl_3$
7.88
7.87
7.87
7.87
7.14
6.90
6.90
6.90
6.29
6.29
6.28
6.28
3.82
2.69
1.68
0.95
0.99
0.97
0.97
3.00
2.36
6.09
10.0 9.5 9.0 8.5 8.0 7.5 7.0 6.5 6.0 5.5 5.0 4.5 4.0 3.5 3.0 2.5 2.0 1.5 1.0 0.5 0.0 -0.5 -1.0
f1 (ppm)

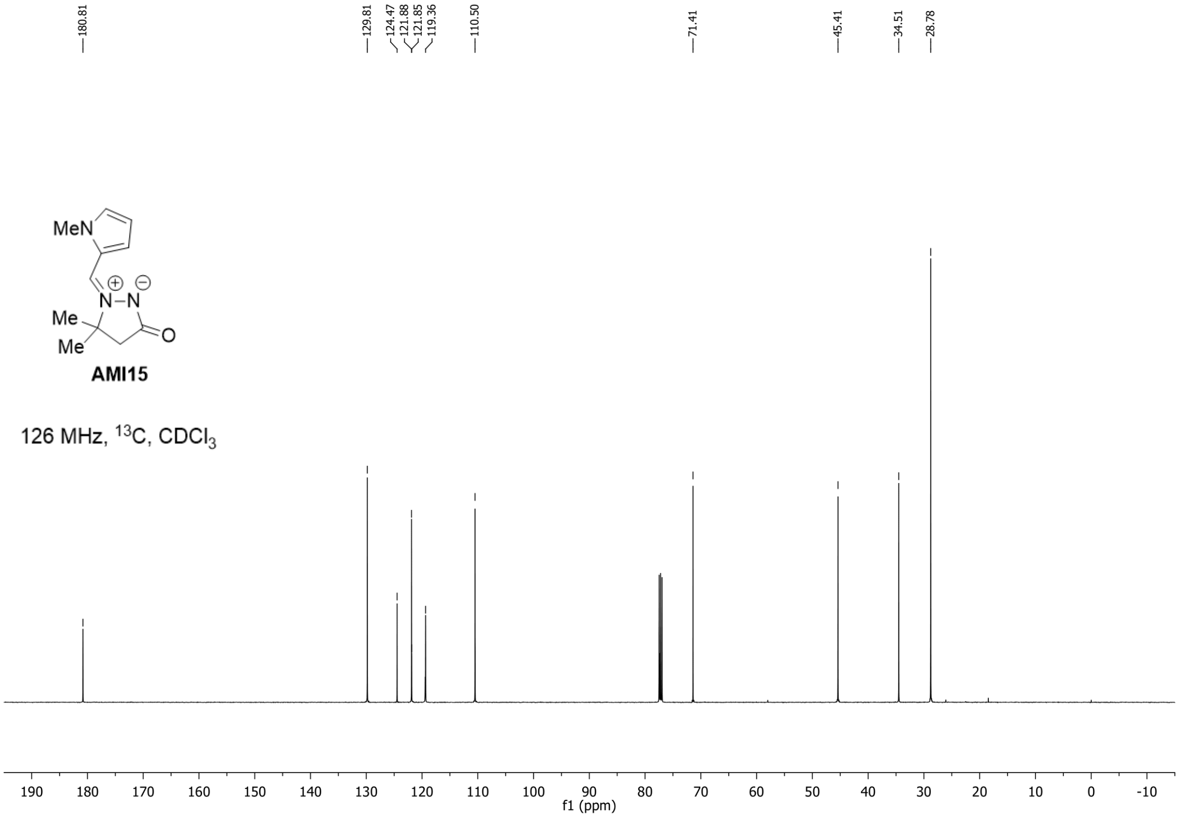

MeN
Me
Me
O
AMI15
126 MHz, ^{13}C, $CDCl_3$
180.81
129.81
124.47
121.88
121.85
119.36
110.50
71.41
45.41
34.51
28.78
190 180 170 160 150 140 130 120 110 100 90 80 70 60 50 40 30 20 10 0 -10
f1 (ppm)

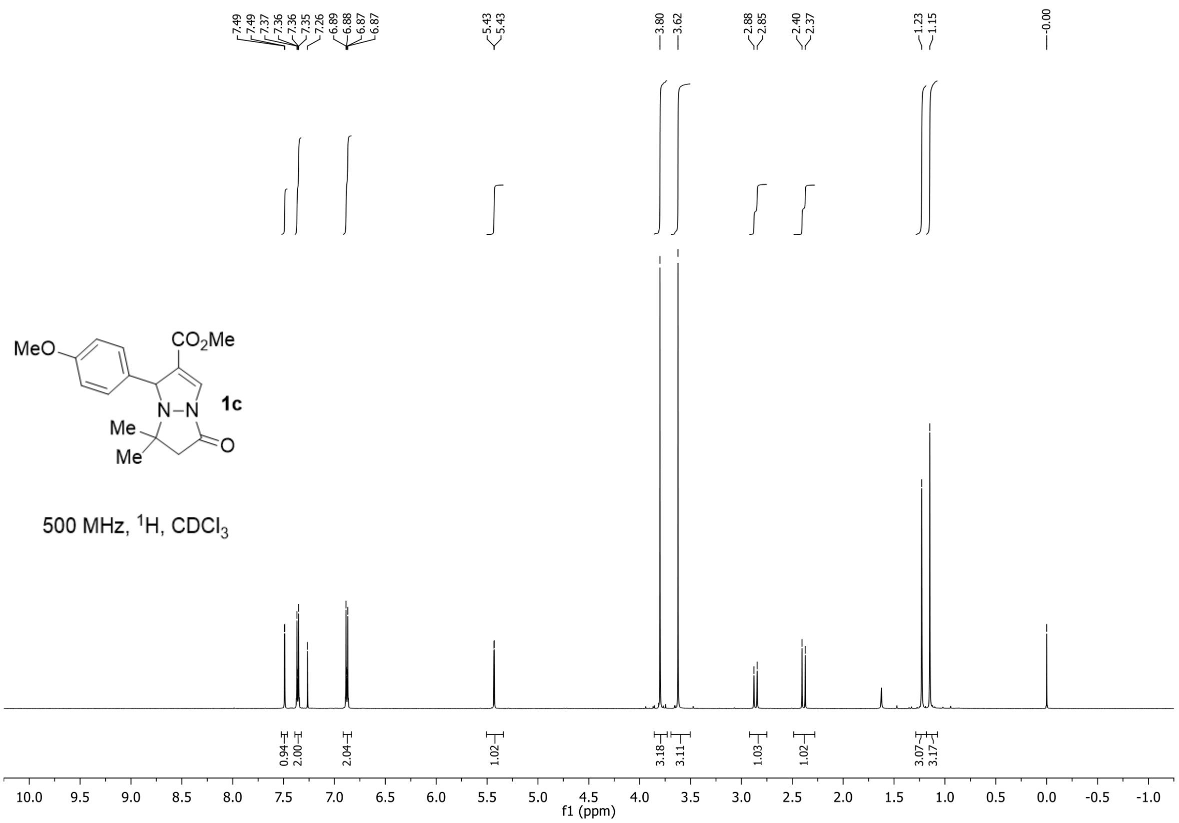
7.49
7.49
7.37
7.36
7.36
7.35
7.26
6.89
6.88
6.87
6.87
5.43
5.43
3.80
3.62
2.88
2.85
2.40
2.37
1.23
1.15
-0.00
MeO
CO2Me
N–N
1c
Me
Me
O
500 MHz, 1H, CDCl3
0.94
2.00
2.04
1.02
3.18
3.11
1.03
1.02
3.07
3.17
f1 (ppm)

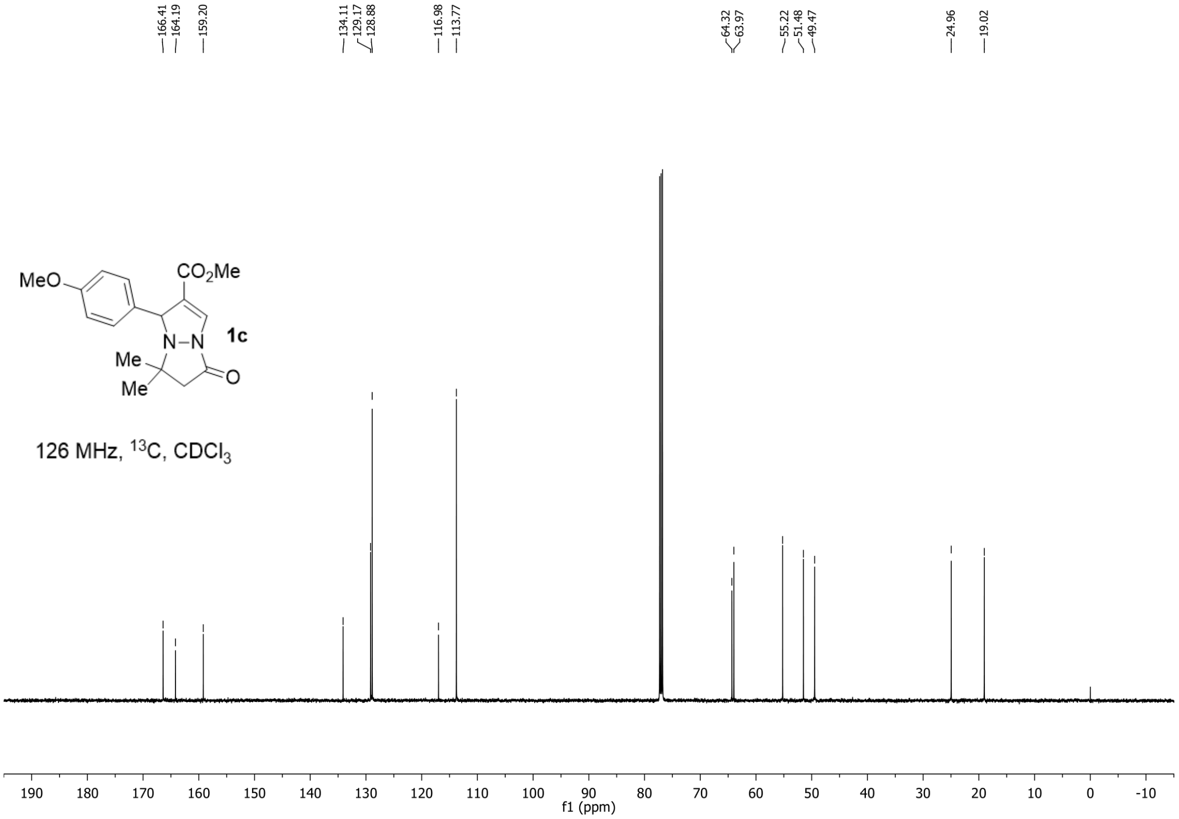
166.41
164.19
159.20
134.11
129.17
128.88
116.98
113.77
64.32
63.97
55.22
51.48
49.47
24.96
19.02
MeO
CO2Me
N–N
1c
Me
Me
O
126 MHz, 13C, CDCl3
f1 (ppm)

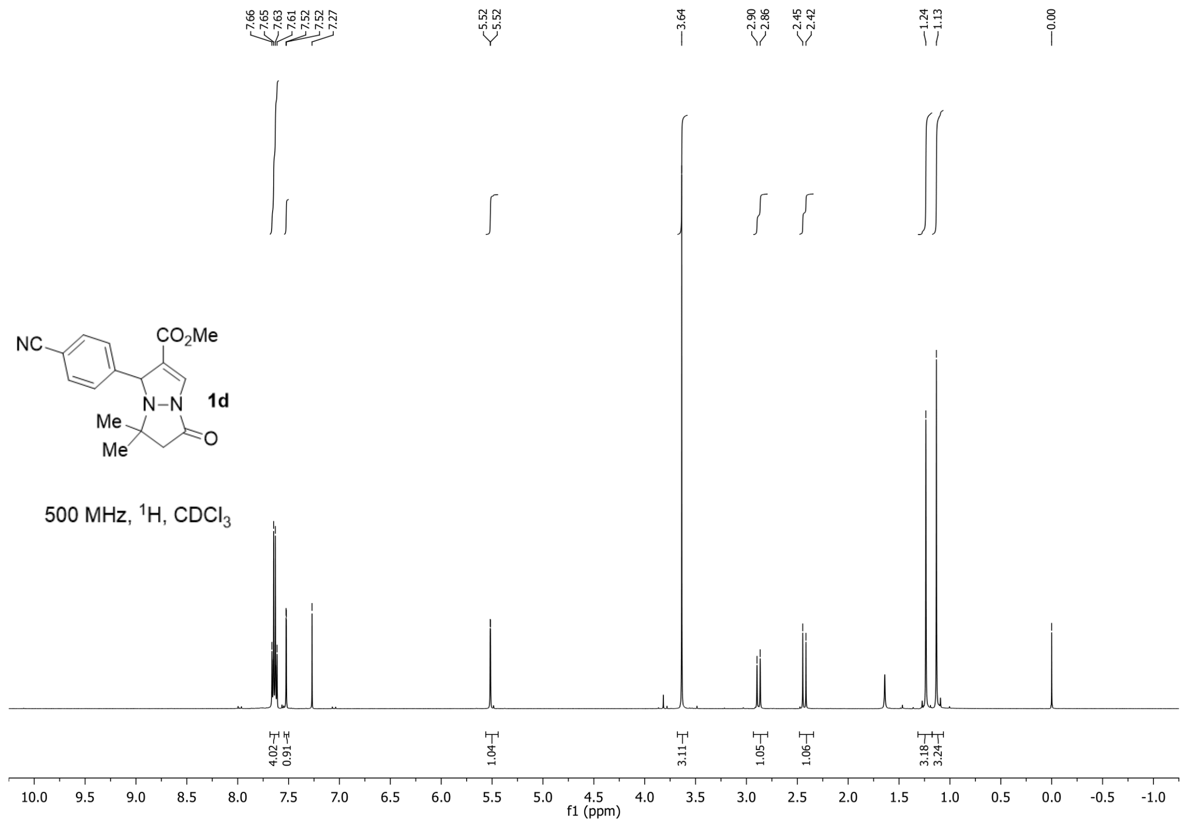
NC
CO2Me
N–N
Me
Me
O
1d
500 MHz, 1H, CDCl3
7.66 7.65 7.63 7.61 7.52 7.52 7.27
5.52 5.52
3.64
2.90 2.86
2.45 2.42
1.24 1.13
0.00
4.02 0.91 1.04 3.11 1.05 1.06 3.18 3.24
f1 (ppm)

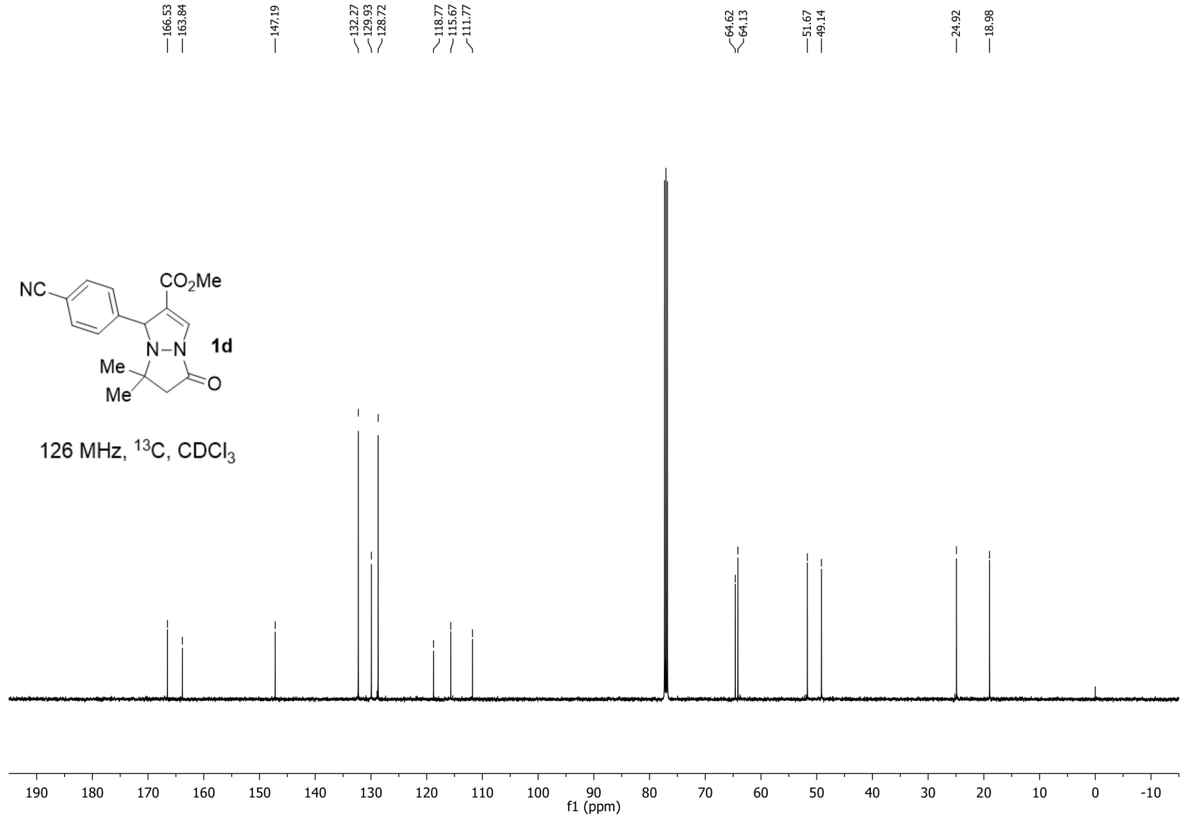
NC
CO2Me
N–N
Me
Me
O
1d
126 MHz, 13C, CDCl3
166.53 163.84
147.19
132.27 129.93 128.72
118.77 115.67 111.77
64.62 64.13
51.67 49.14
24.92
18.98
f1 (ppm)

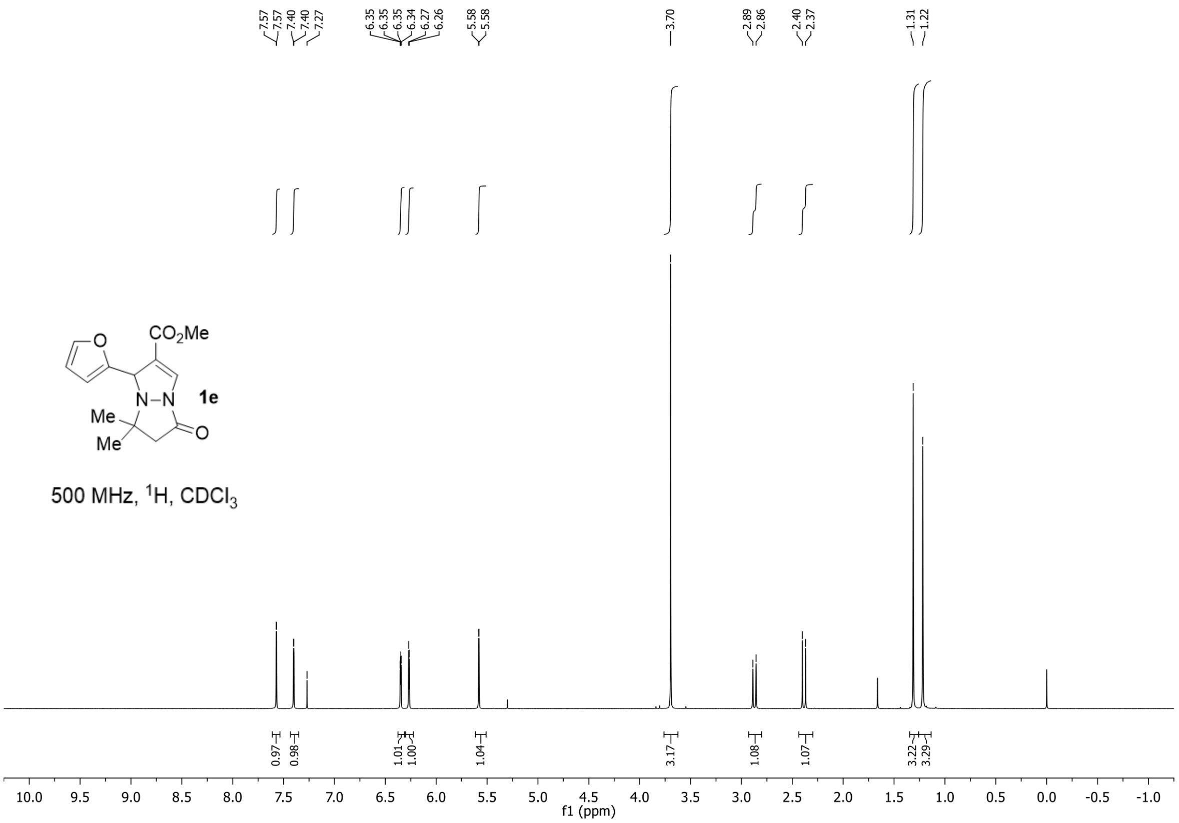
7.57 7.57 7.40 7.40 7.27
6.35 6.35 6.35 6.34 6.27 6.26
5.58 5.58
3.70
2.89 2.86
2.40 2.37
1.31 1.22
CO2Me
O
N–N
Me
Me
O
1e
500 MHz, 1H, CDCl3
0.97 0.98 1.01 1.00 1.04 3.17 1.08 1.07 3.22 3.29
10.0 9.5 9.0 8.5 8.0 7.5 7.0 6.5 6.0 5.5 5.0 4.5 4.0 3.5 3.0 2.5 2.0 1.5 1.0 0.5 0.0 -0.5 -1.0
f1 (ppm)

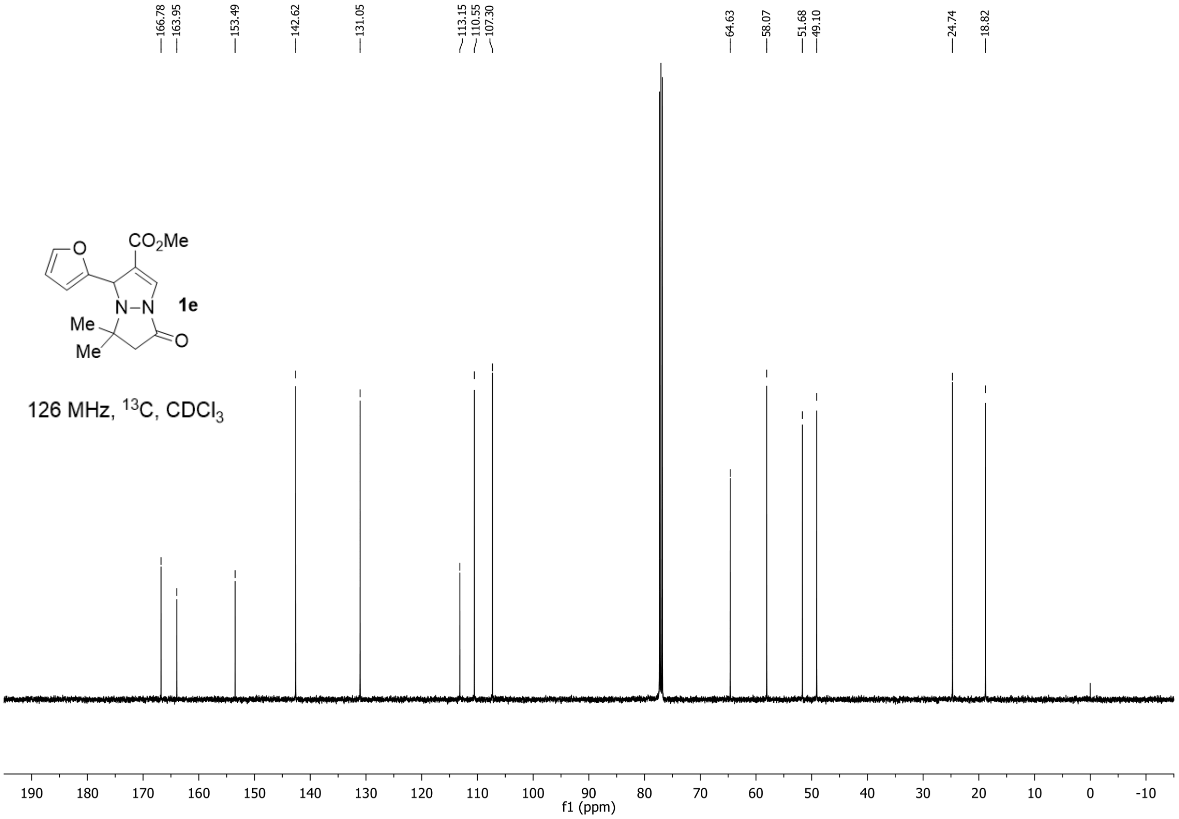
166.78 163.95
153.49
142.62
131.05
113.15 110.55 107.30
64.63
58.07
51.68 49.10
24.74
18.82
CO2Me
O
N–N
Me
Me
O
1e
126 MHz, 13C, CDCl3
190 180 170 160 150 140 130 120 110 100 90 80 70 60 50 40 30 20 10 0 -10
f1 (ppm)

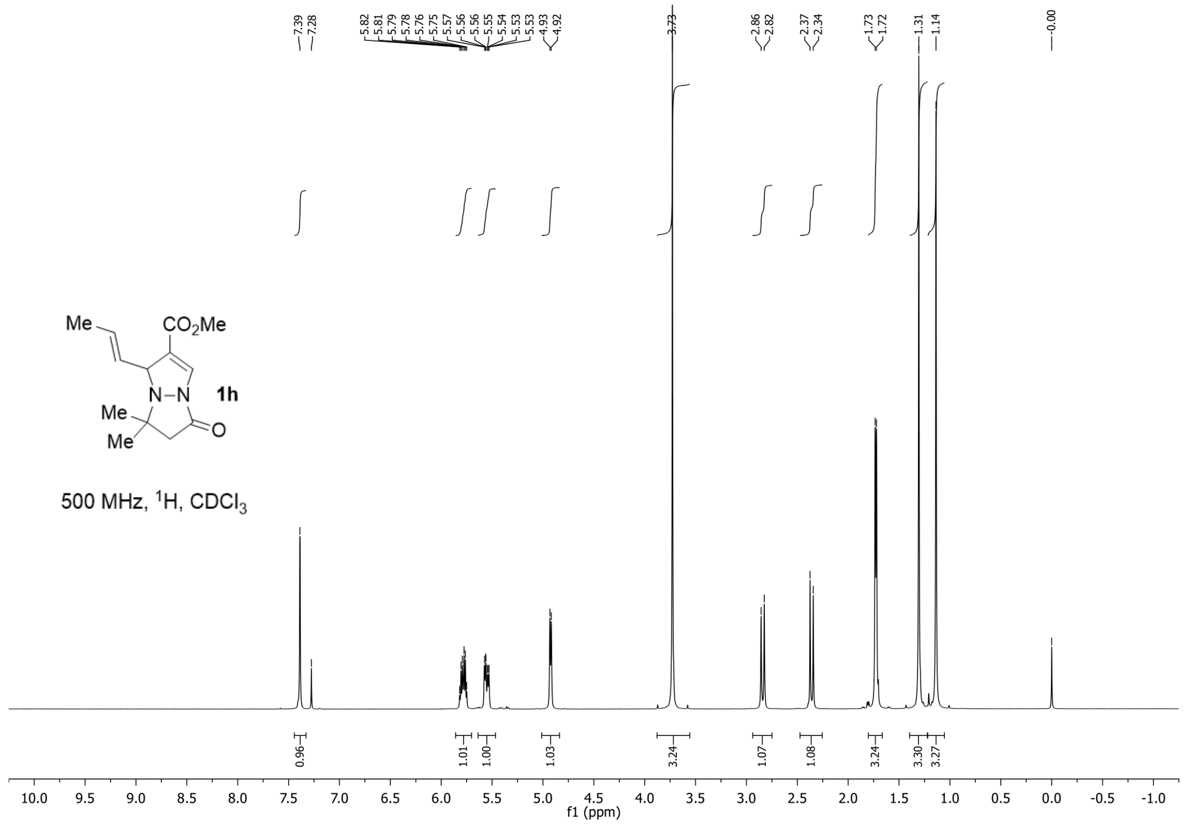
7.39
7.28
5.82
5.81
5.79
5.78
5.76
5.75
5.57
5.56
5.56
5.55
5.54
5.53
5.53
4.93
4.92
3.73
2.86
2.82
2.37
2.34
1.73
1.72
1.31
1.14
0.00
Me
CO2Me
N
N
1h
Me
Me
O
500 MHz, 1H, CDCl3
0.96
1.01
1.00
1.03
3.24
1.07
1.08
3.24
3.30
3.27
f1 (ppm)

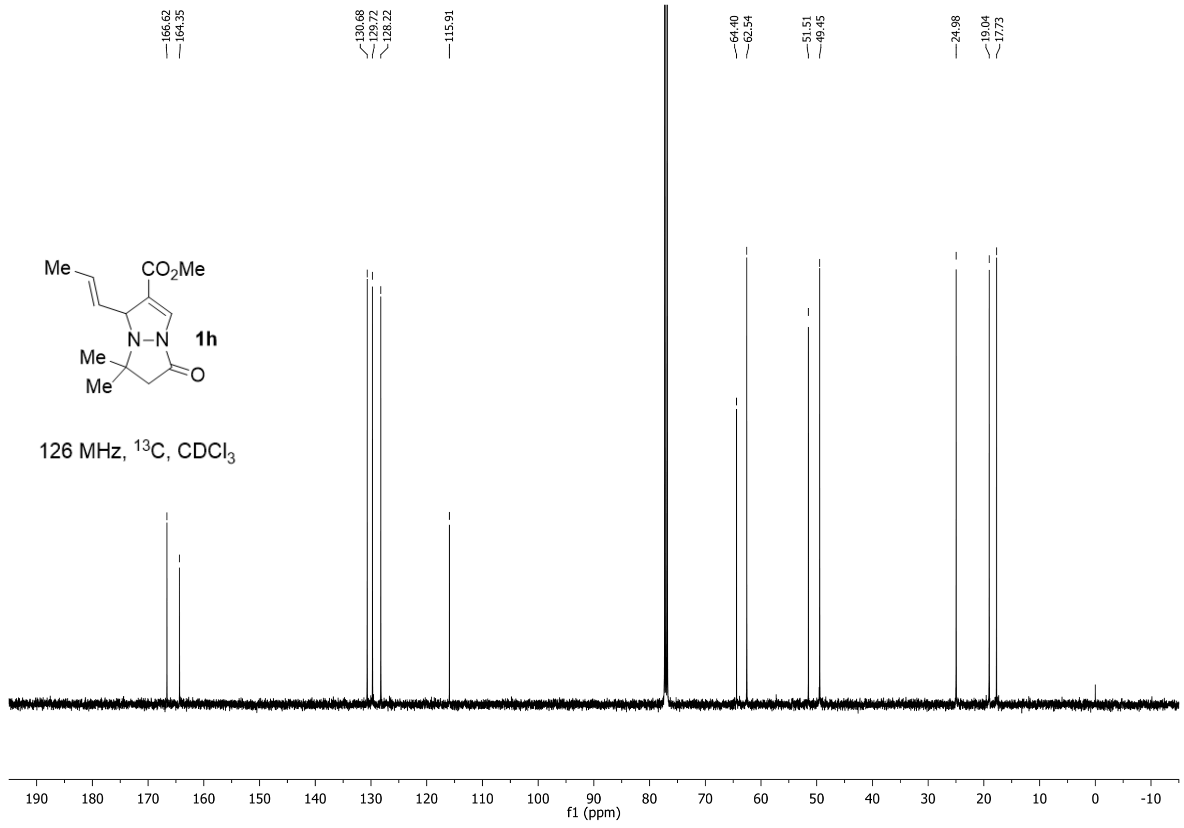
166.62
164.35
130.68
129.72
128.22
115.91
64.40
62.54
51.51
49.45
24.98
19.04
17.73
Me
CO2Me
N
N
1h
Me
Me
O
126 MHz, 13C, CDCl3
f1 (ppm)

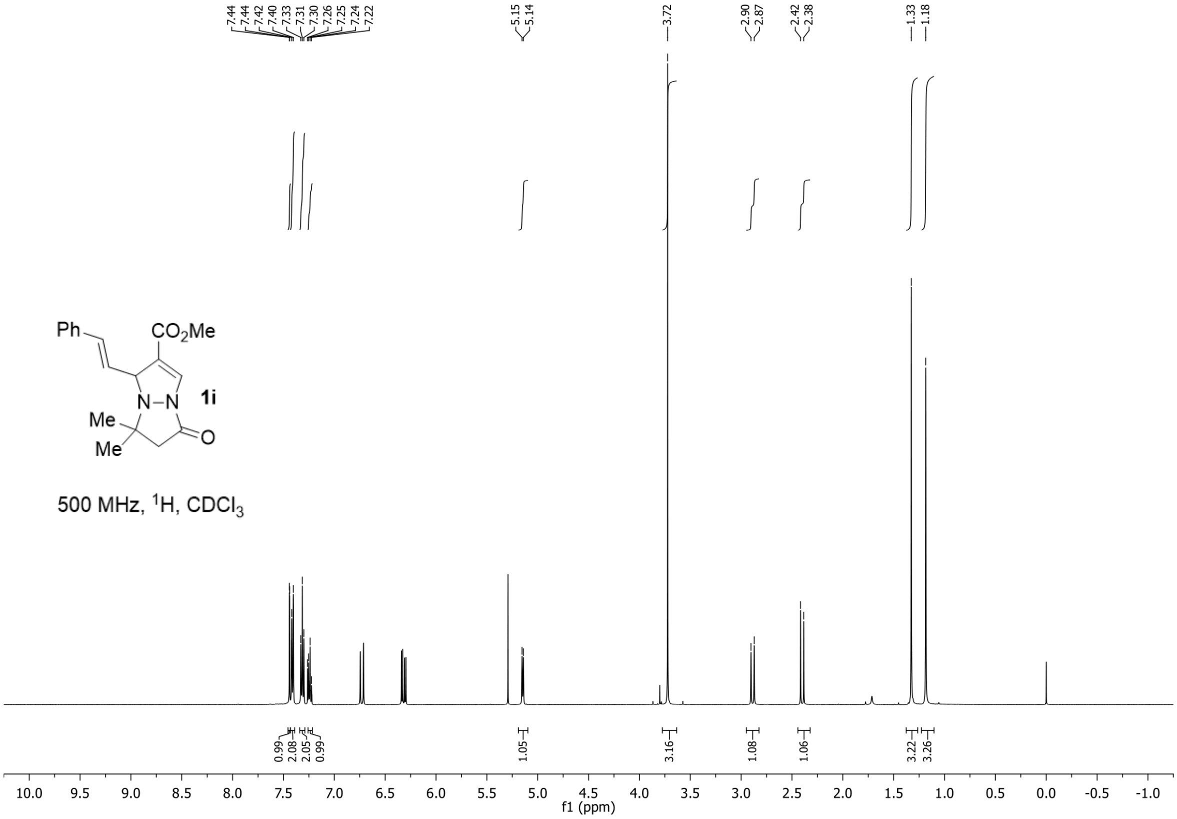
7.44
7.44
7.42
7.40
7.33
7.31
7.30
7.26
7.25
7.24
7.22
5.15
5.14
3.72
2.90
2.87
2.42
2.38
1.33
1.18
Ph
CO2Me
N–N
1i
Me
Me
O
500 MHz, 1H, CDCl3
0.99
2.08
2.05
0.99
1.05
3.16
1.08
1.06
3.22
3.26
f1 (ppm)

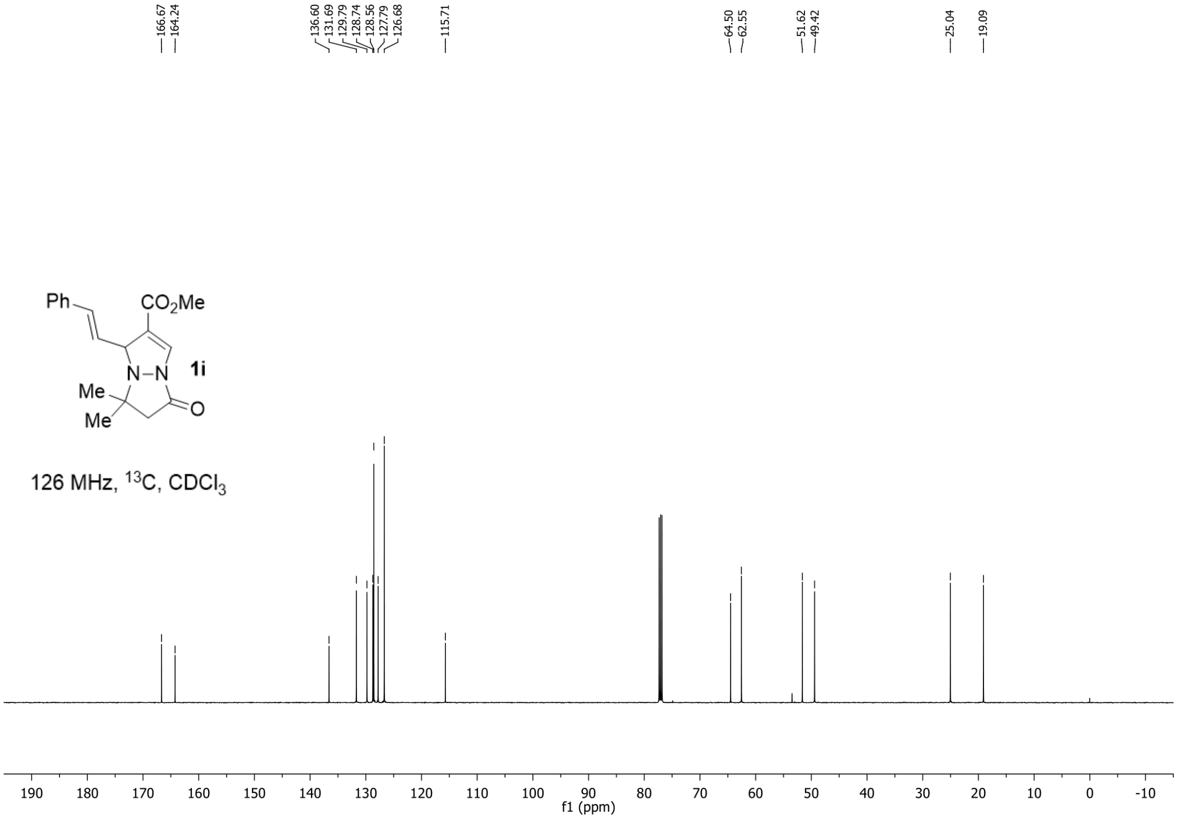
166.67
164.24
136.60
131.69
129.79
128.74
128.56
127.79
126.68
115.71
64.50
62.55
51.62
49.42
25.04
19.09
Ph
CO2Me
N–N
1i
Me
Me
O
126 MHz, 13C, CDCl3
f1 (ppm)

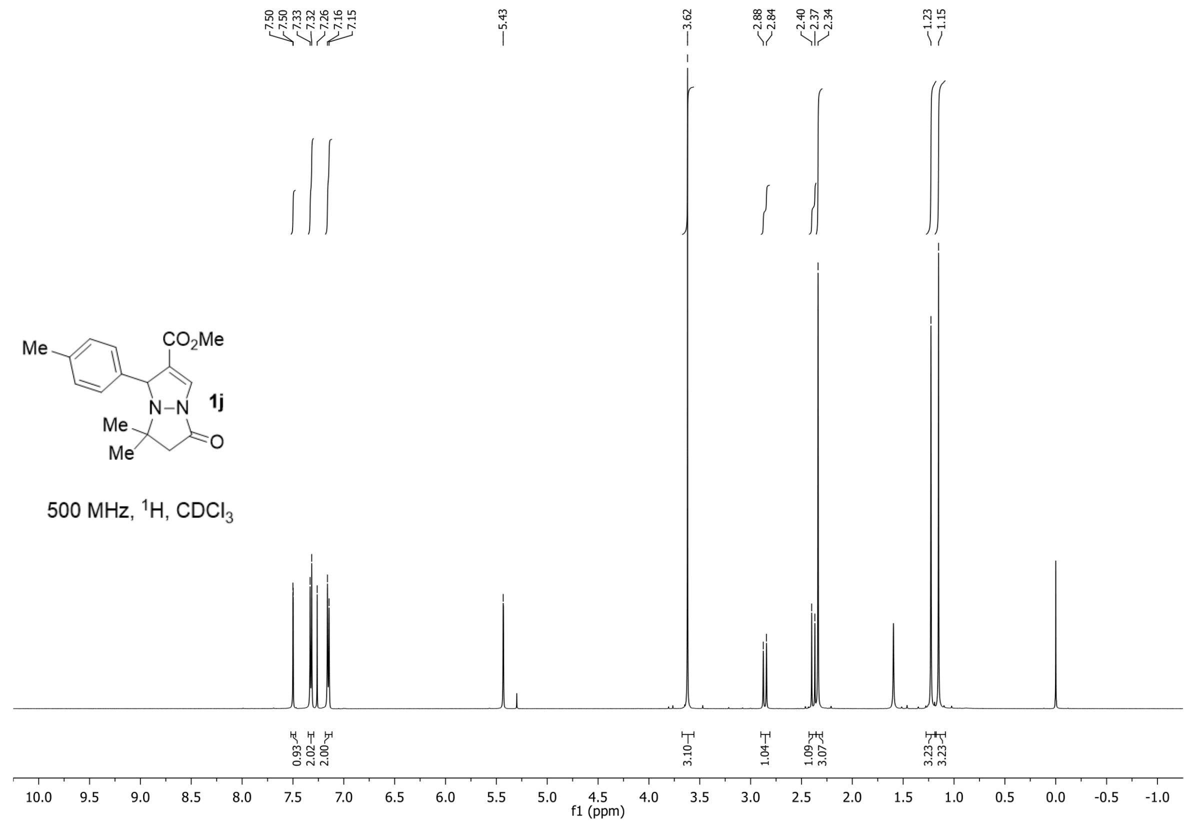
CO2Me
Me
N–N
1j
Me
Me
O
500 MHz, 1H, CDCl3
7.50
7.50
7.33
7.32
7.26
7.16
7.15
5.43
3.62
2.88
2.84
2.40
2.37
2.34
1.23
1.15
0.93
2.02
2.00
3.10
1.04
1.09
3.07
3.23
3.23
f1 (ppm)

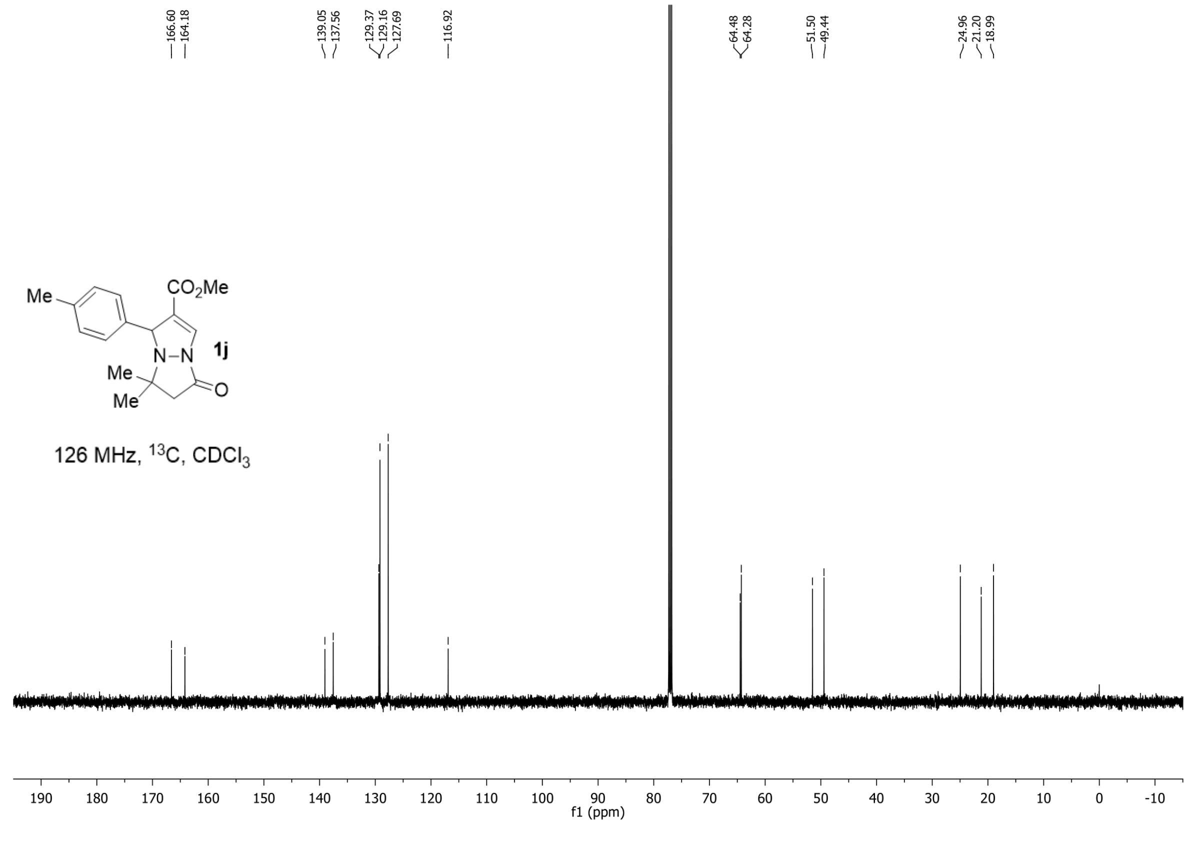
CO2Me
Me
N–N
1j
Me
Me
O
126 MHz, 13C, CDCl3
166.60
164.18
139.05
137.56
129.37
129.16
127.69
116.92
64.48
64.28
51.50
49.44
24.96
21.20
18.99
f1 (ppm)

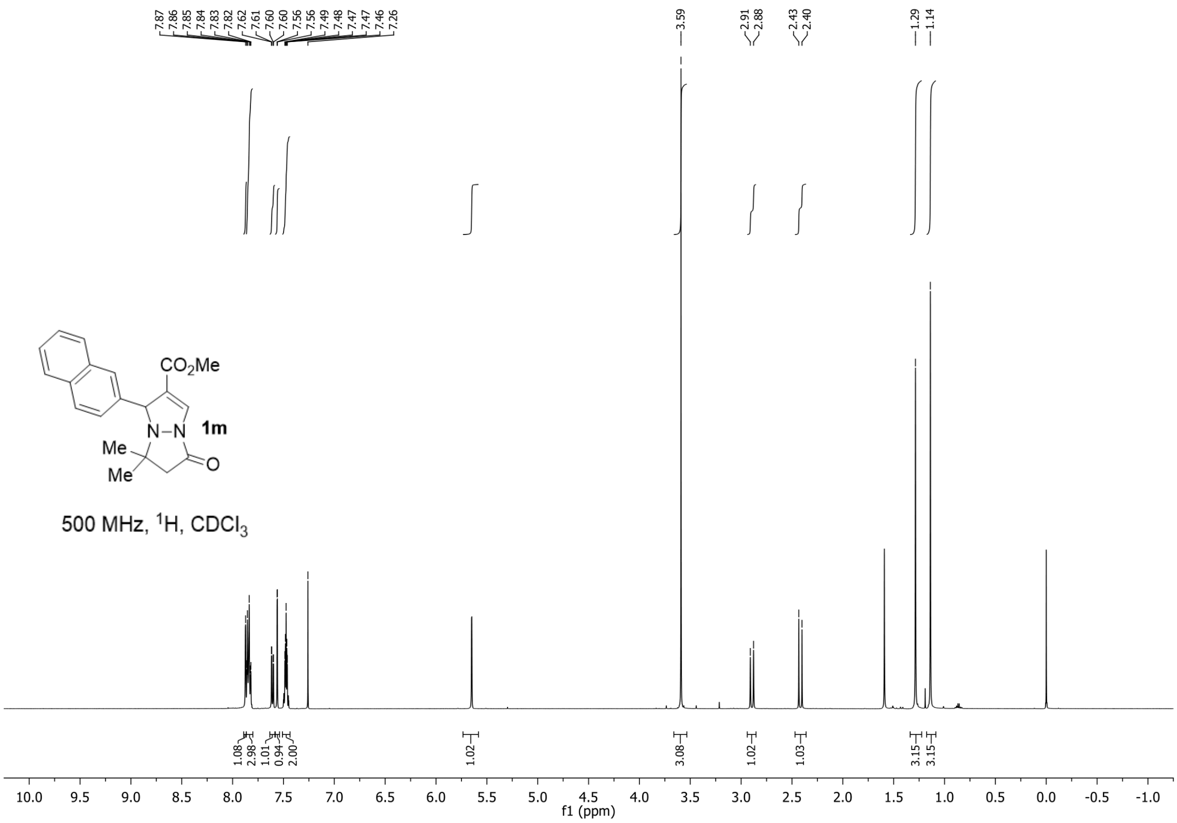

7.87
7.86
7.85
7.84
7.83
7.82
7.62
7.61
7.60
7.60
7.56
7.56
7.49
7.48
7.47
7.47
7.46
7.26
3.59
2.91
2.88
2.43
2.40
1.29
1.14
CO2Me
N–N
1m
Me
Me
O
500 MHz, 1H, CDCl3
1.08
2.98
1.01
0.94
2.00
1.02
3.08
1.02
1.03
3.15
3.15
f1 (ppm)

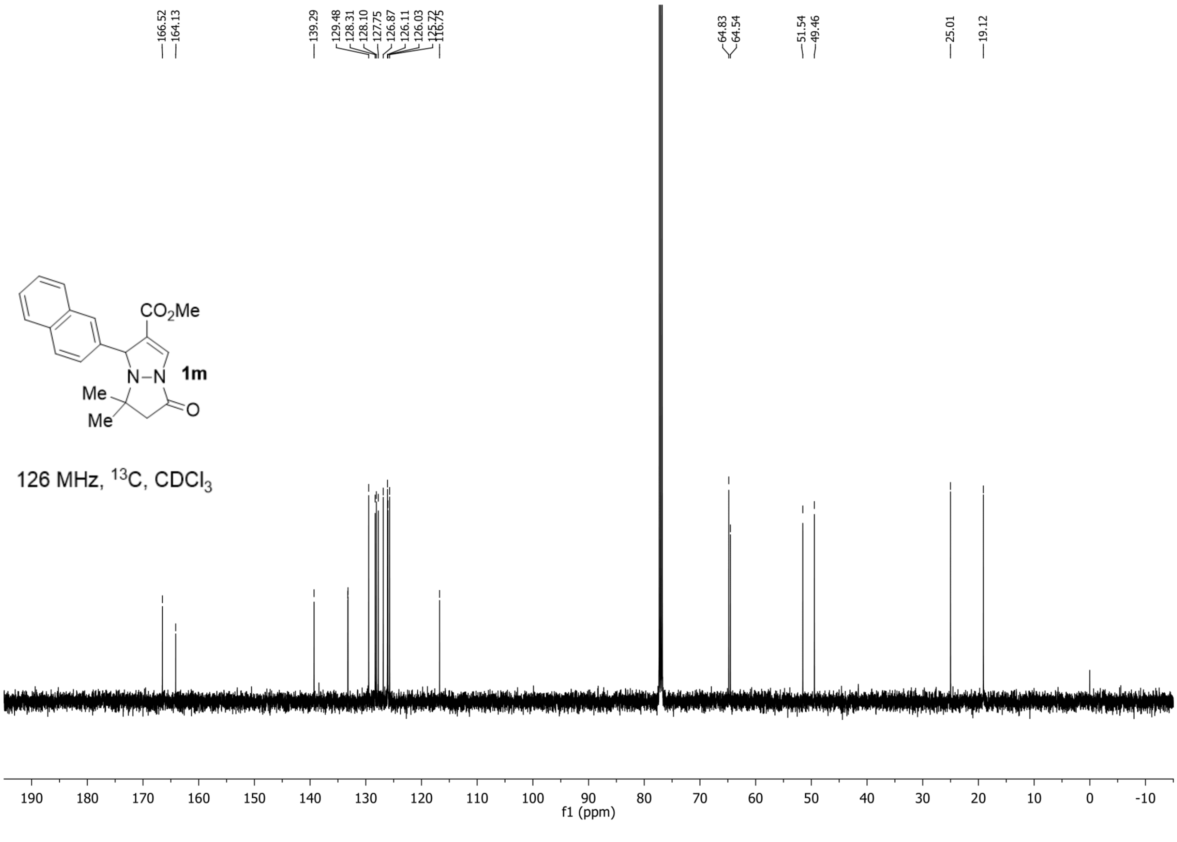

166.52
164.13
139.29
129.48
128.31
128.10
127.75
126.87
126.11
126.03
125.72
116.75
64.83
64.54
51.54
49.46
25.01
19.12
CO2Me
N–N
1m
Me
Me
O
126 MHz, 13C, CDCl3
f1 (ppm)

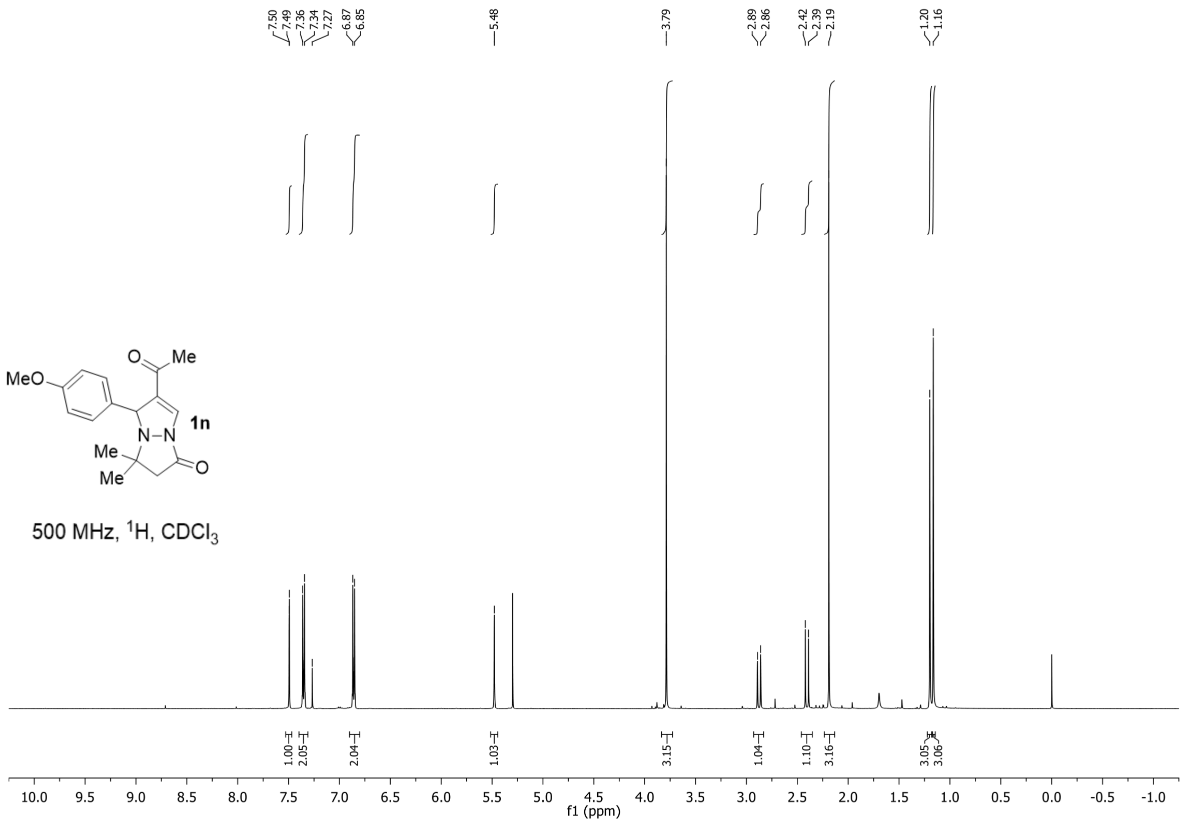

7.50
7.49
7.36
7.34
7.27
6.87
6.85
5.48
3.79
2.89
2.86
2.42
2.39
2.19
1.20
1.16
MeO
O
Me
1n
N–N
Me
Me
O
500 MHz, ^{1}H, CDCl$_3$
1.00
2.05
2.04
1.03
3.15
1.04
1.10
3.16
3.05
3.06
f1 (ppm)

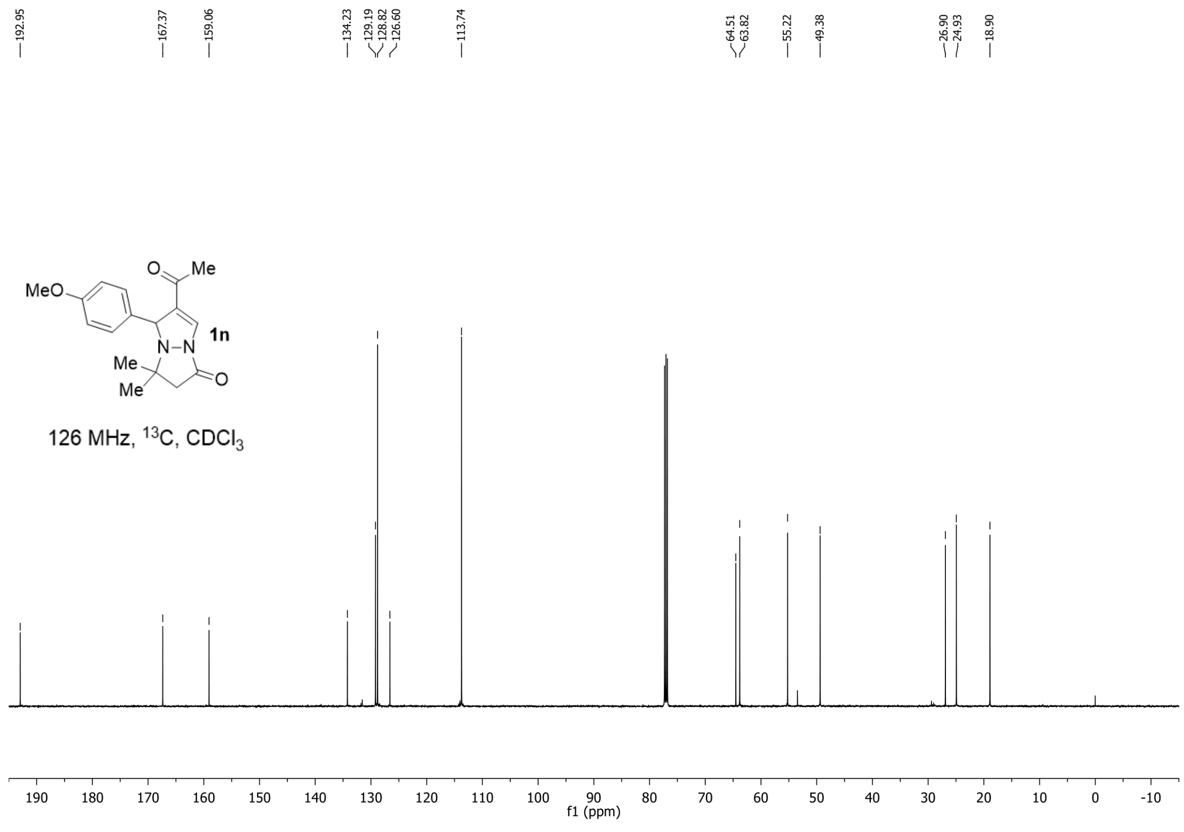

192.95
167.37
159.06
134.23
129.19
128.82
126.60
113.74
64.51
63.82
55.22
49.38
26.90
24.93
18.90
MeO
O
Me
1n
N–N
Me
Me
O
126 MHz, ^{13}C, CDCl$_3$
f1 (ppm)

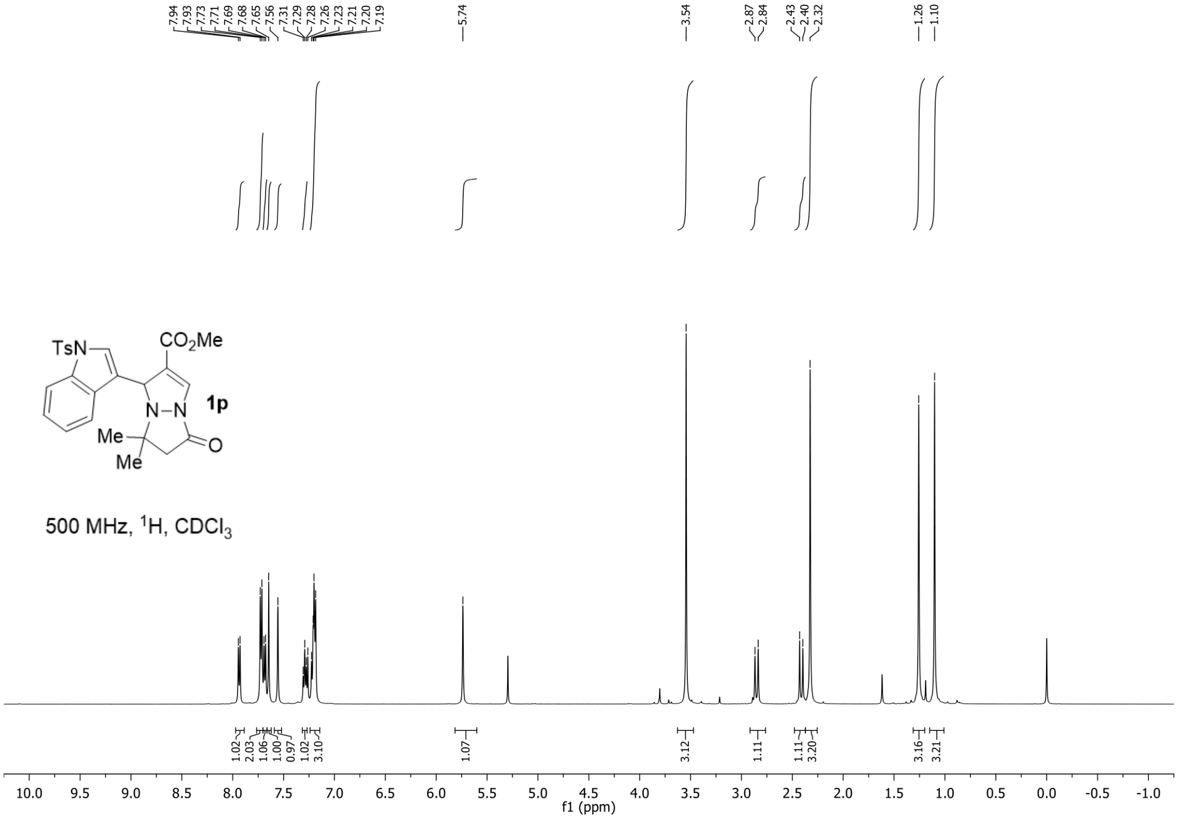
TsN
CO2Me
1p
Me
Me
O
500 MHz, 1H, CDCl3
f1 (ppm)

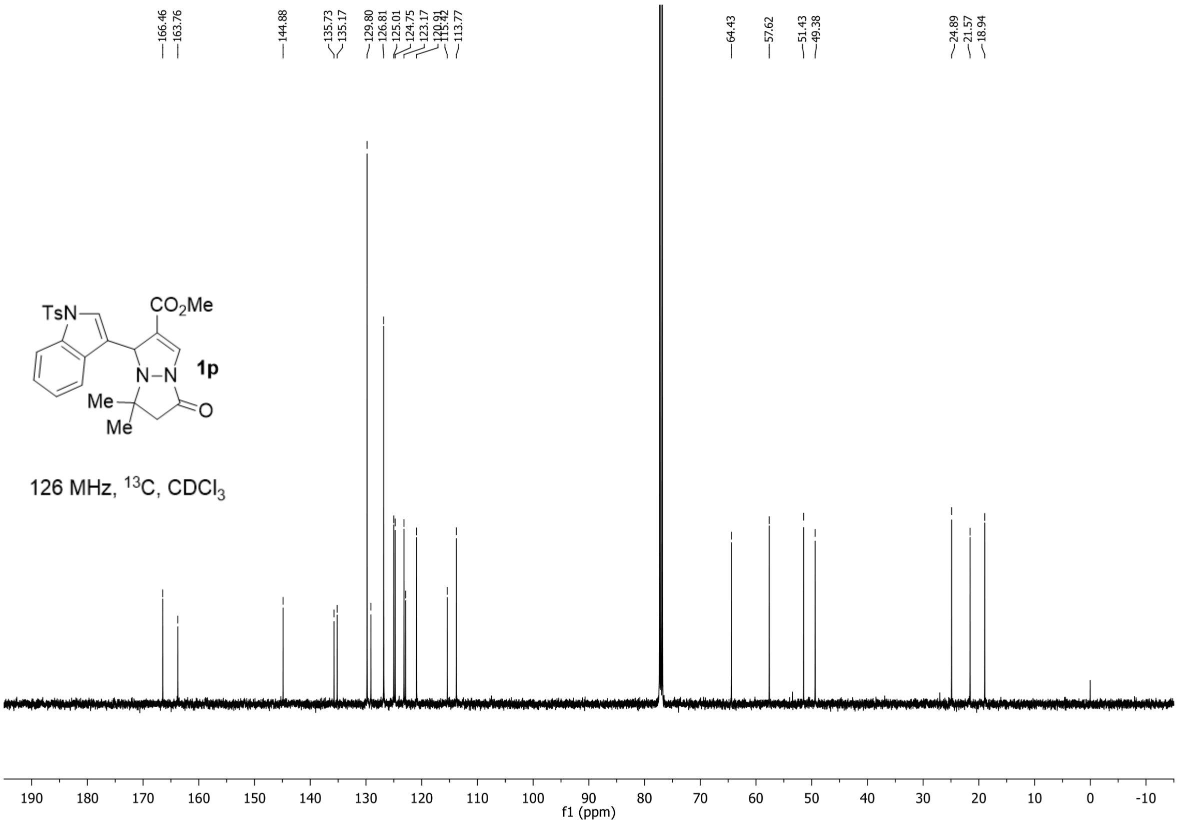
TsN
CO2Me
1p
Me
Me
O
126 MHz, 13C, CDCl3
f1 (ppm)

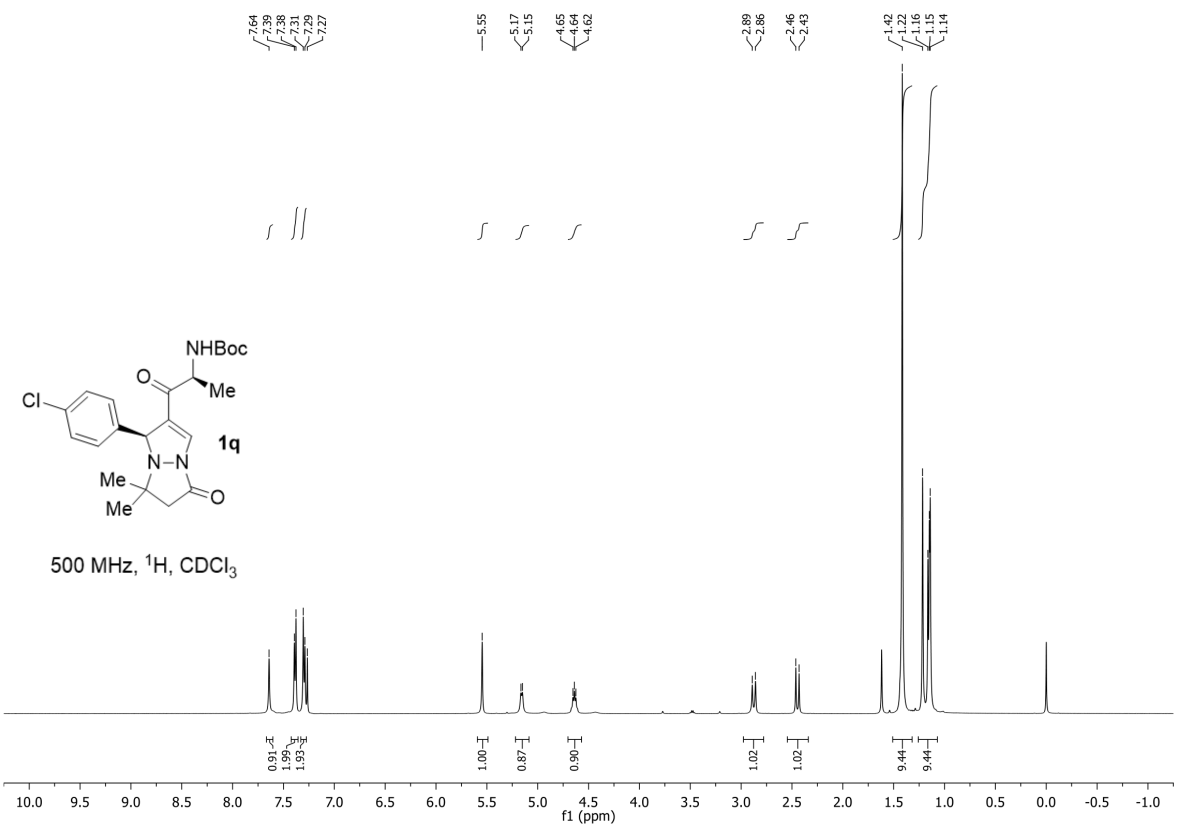
7.64 7.39 7.38 7.31 7.29 7.27
5.55
5.17 5.15
4.65 4.64 4.62
2.89 2.86
2.46 2.43
1.42 1.22 1.16 1.15 1.14
NHBoc
O
Me
Cl
1q
N
N
Me
Me
O
500 MHz, 1H, CDCl3
0.91 1.99 1.93
1.00
0.87
0.90
1.02
1.02
9.44
9.44
10.0 9.5 9.0 8.5 8.0 7.5 7.0 6.5 6.0 5.5 5.0 4.5 4.0 3.5 3.0 2.5 2.0 1.5 1.0 0.5 0.0 -0.5 -1.0
f1 (ppm)

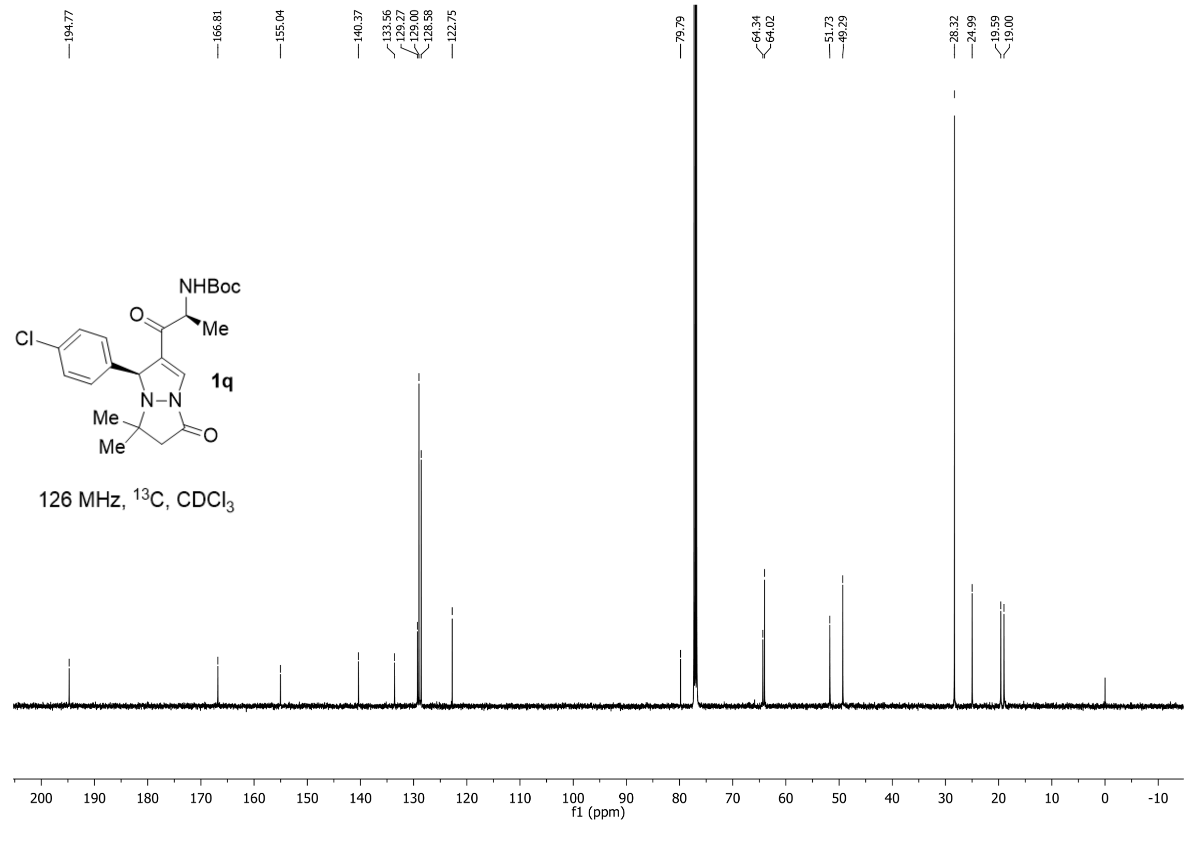
194.77
166.81
155.04
140.37
133.56 129.27 129.00 128.58
122.75
79.79
64.34 64.02
51.73 49.29
28.32 24.99
19.59 19.00
NHBoc
O
Me
Cl
1q
N
N
Me
Me
O
126 MHz, 13C, CDCl3
200 190 180 170 160 150 140 130 120 110 100 90 80 70 60 50 40 30 20 10 0 -10
f1 (ppm)

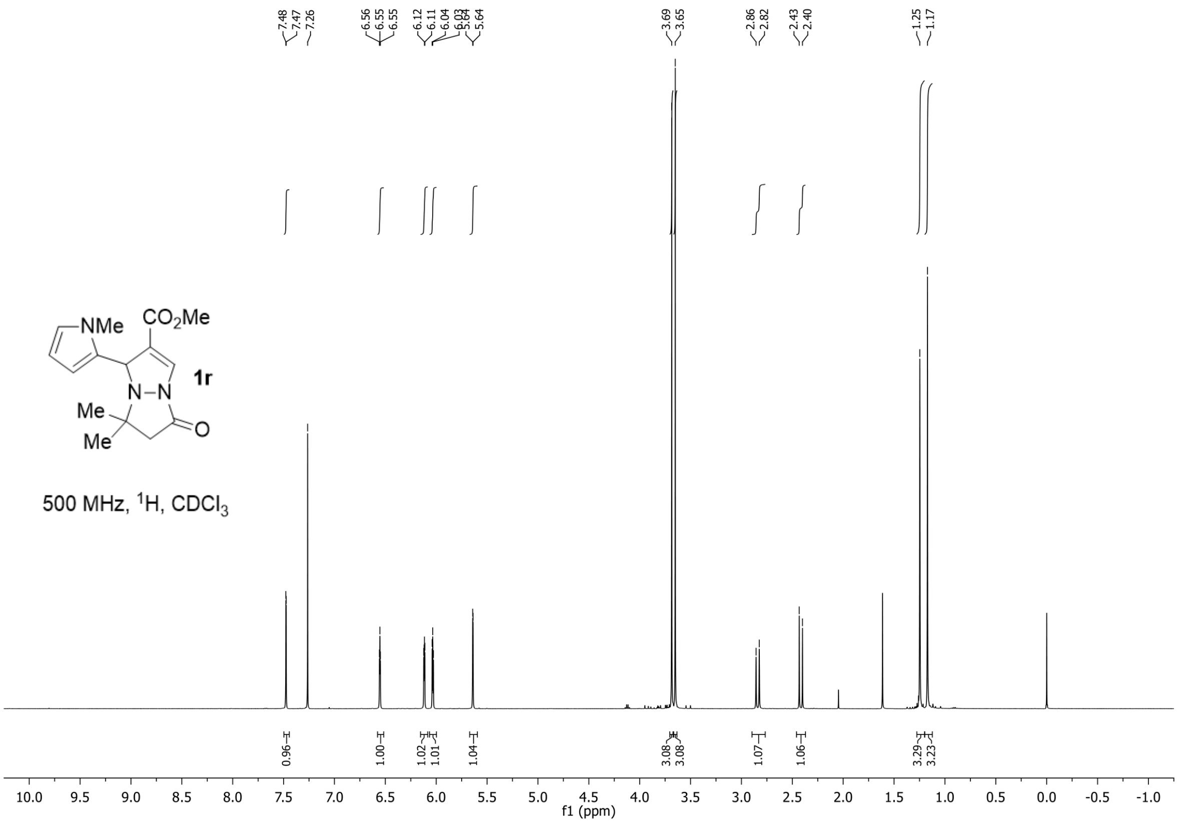
7.48
7.47
7.26
6.56
6.55
6.55
6.12
6.11
6.04
6.03
6.04
5.64
3.69
3.65
2.86
2.82
2.43
2.40
1.25
1.17
NMe
CO_2Me
1r
Me
Me
500 MHz, ^{1}H, $CDCl_3$
0.96
1.00
1.02
1.01
1.04
3.08
3.08
1.07
1.06
3.29
3.23
10.0 9.5 9.0 8.5 8.0 7.5 7.0 6.5 6.0 5.5 5.0 4.5 4.0 3.5 3.0 2.5 2.0 1.5 1.0 0.5 0.0 -0.5 -1.0
f1 (ppm)

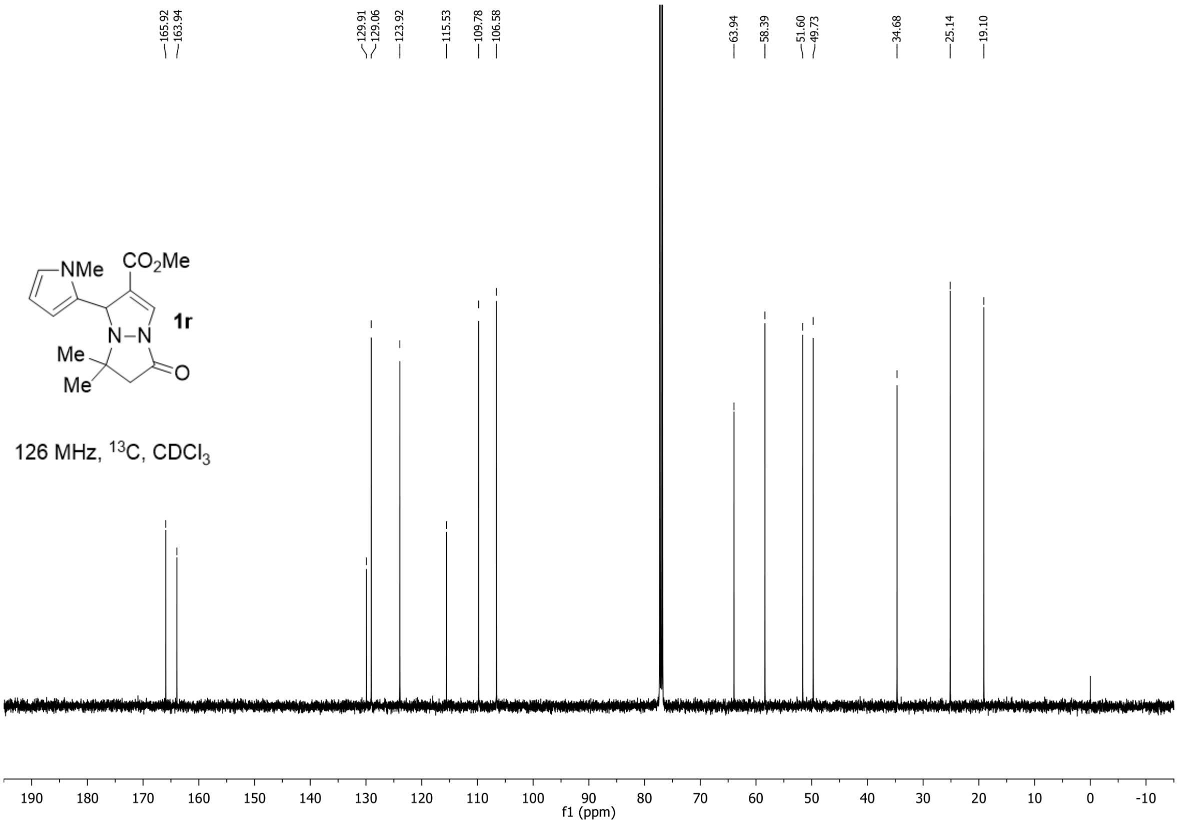
165.92
163.94
129.91
129.06
123.92
115.53
109.78
106.58
63.94
58.39
51.60
49.73
34.68
25.14
19.10
NMe
CO_2Me
1r
Me
Me
126 MHz, ^{13}C, $CDCl_3$
190 180 170 160 150 140 130 120 110 100 90 80 70 60 50 40 30 20 10 0 -10
f1 (ppm)

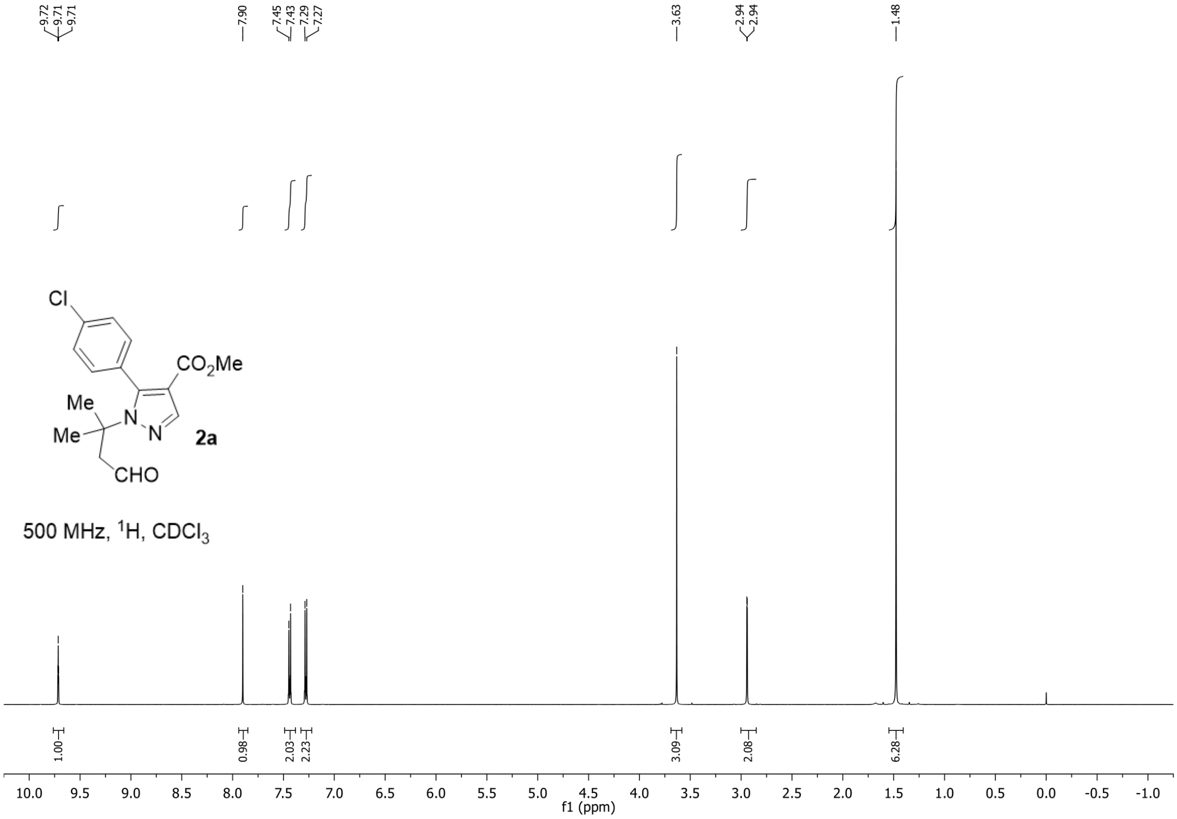

9.72
9.71
9.71
7.90
7.45
7.43
7.29
7.27
3.63
2.94
2.94
1.48
Cl
CO2Me
Me
Me
N
N
2a
CHO
500 MHz, 1H, CDCl3
1.00
0.98
2.03
2.23
3.09
2.08
6.28
f1 (ppm)

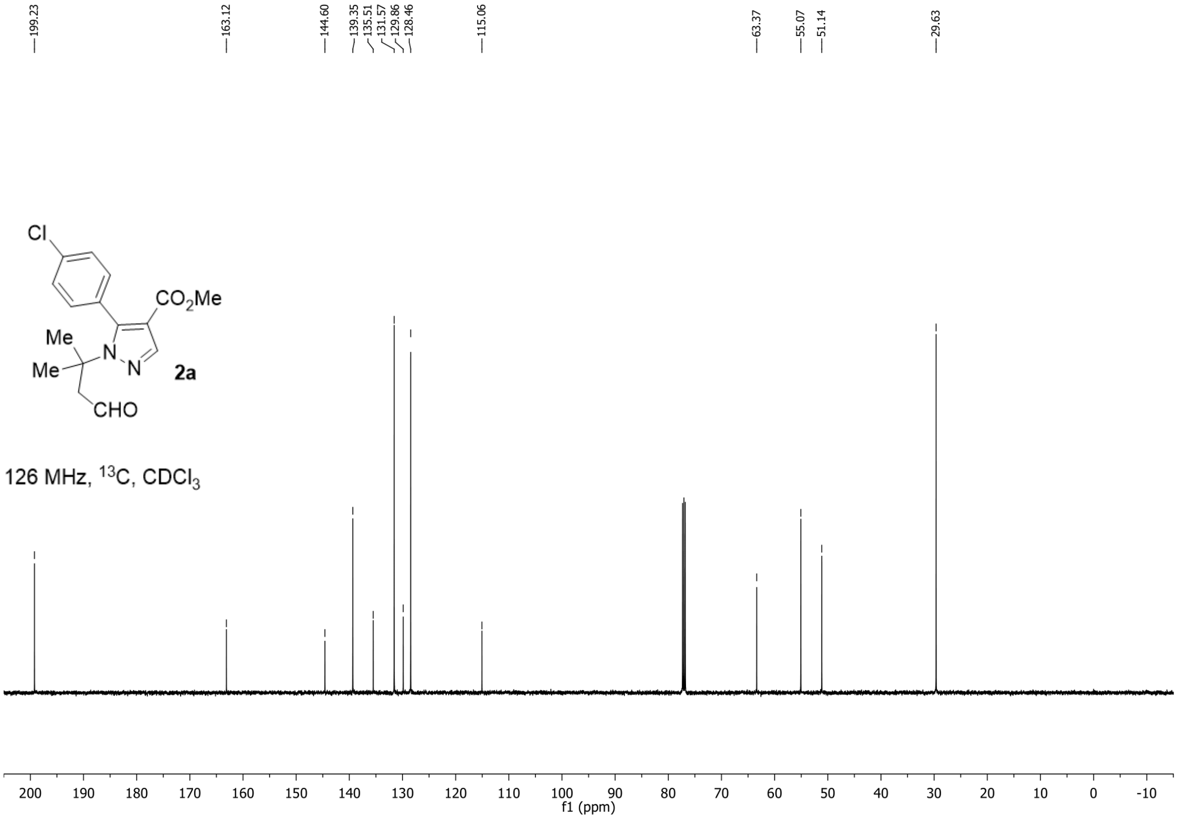

199.23
163.12
144.60
139.35
135.51
131.57
129.86
128.46
115.06
63.37
55.07
51.14
29.63
Cl
CO2Me
Me
Me
N
N
2a
CHO
126 MHz, 13C, CDCl3
f1 (ppm)

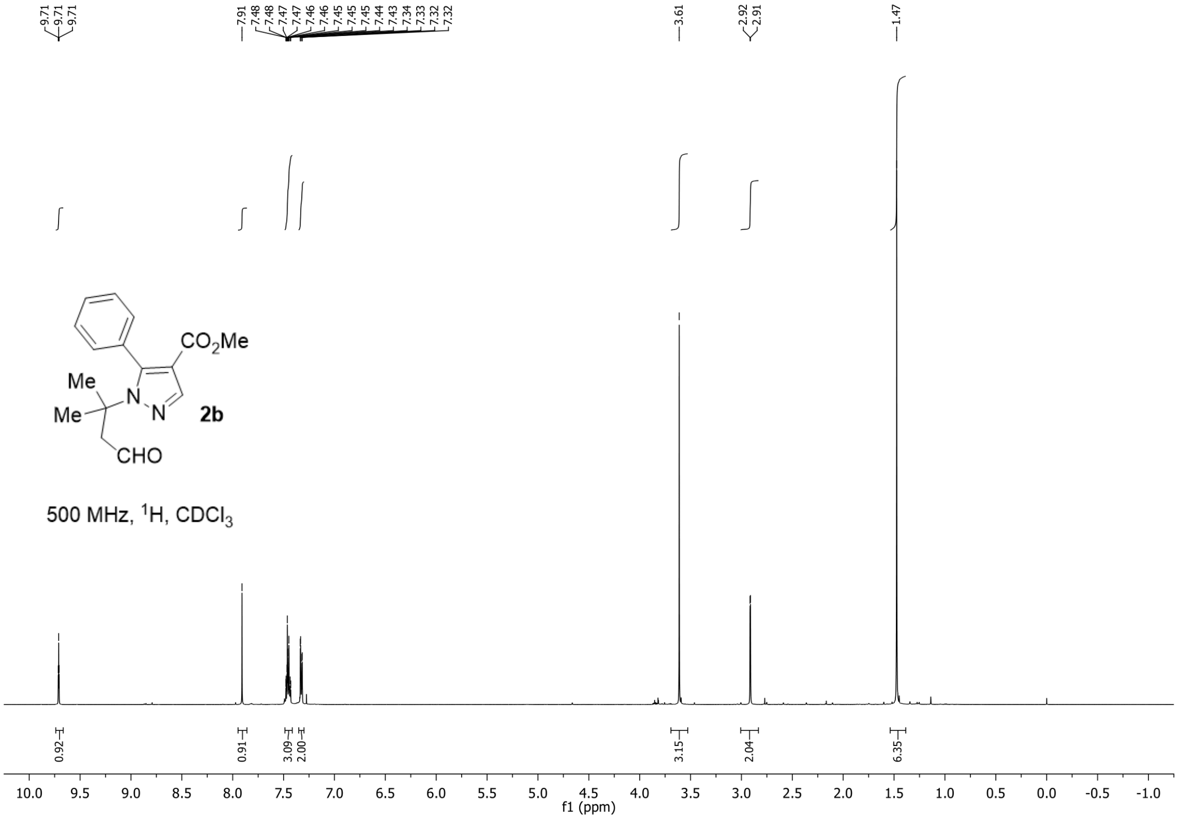
9.71
9.71
9.71
7.91
7.48
7.47
7.47
7.46
7.46
7.45
7.45
7.45
7.44
7.43
7.34
7.33
7.32
7.32
3.61
2.92
2.91
1.47
CO2Me
Me
Me
N
N
2b
CHO
500 MHz, 1H, CDCl3
0.92
0.91
3.09
2.00
3.15
2.04
6.35
f1 (ppm)

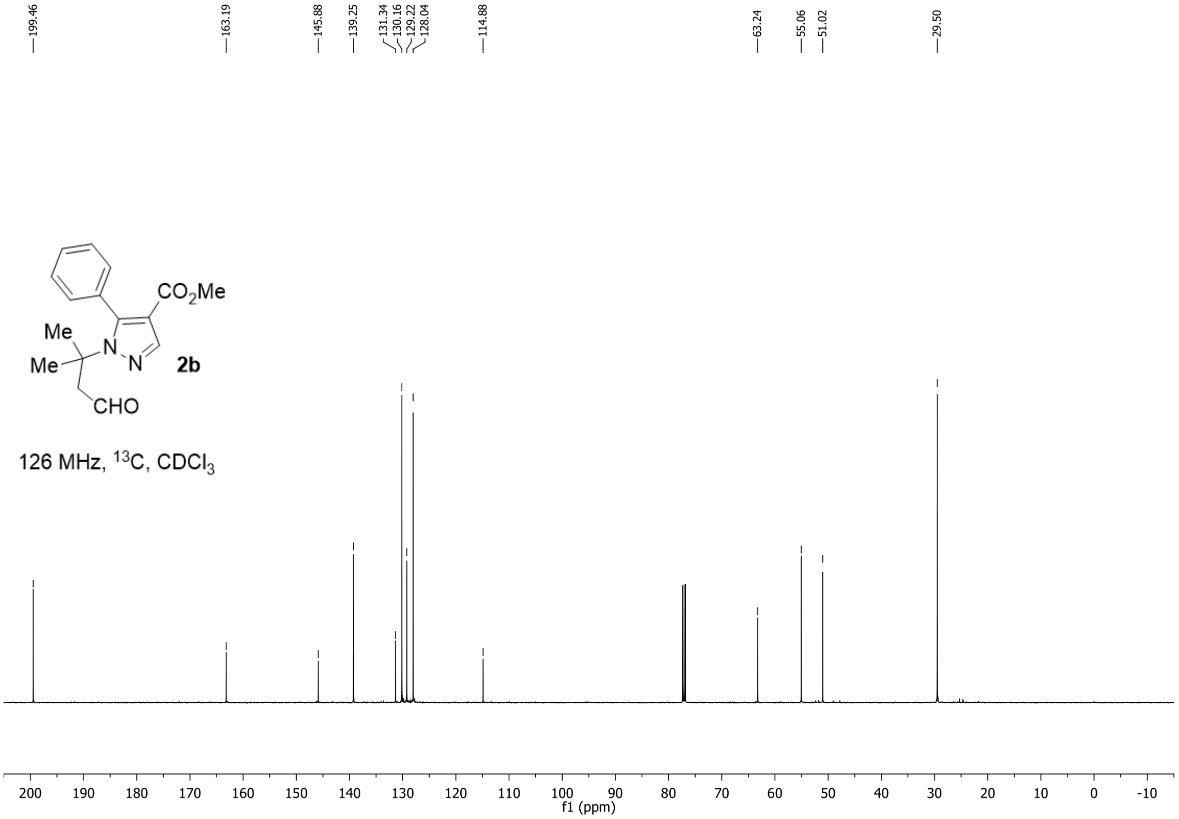
199.46
163.19
145.88
139.25
131.34
130.16
129.22
128.04
114.88
63.24
55.06
51.02
29.50
CO2Me
Me
Me
N
N
2b
CHO
126 MHz, 13C, CDCl3
f1 (ppm)

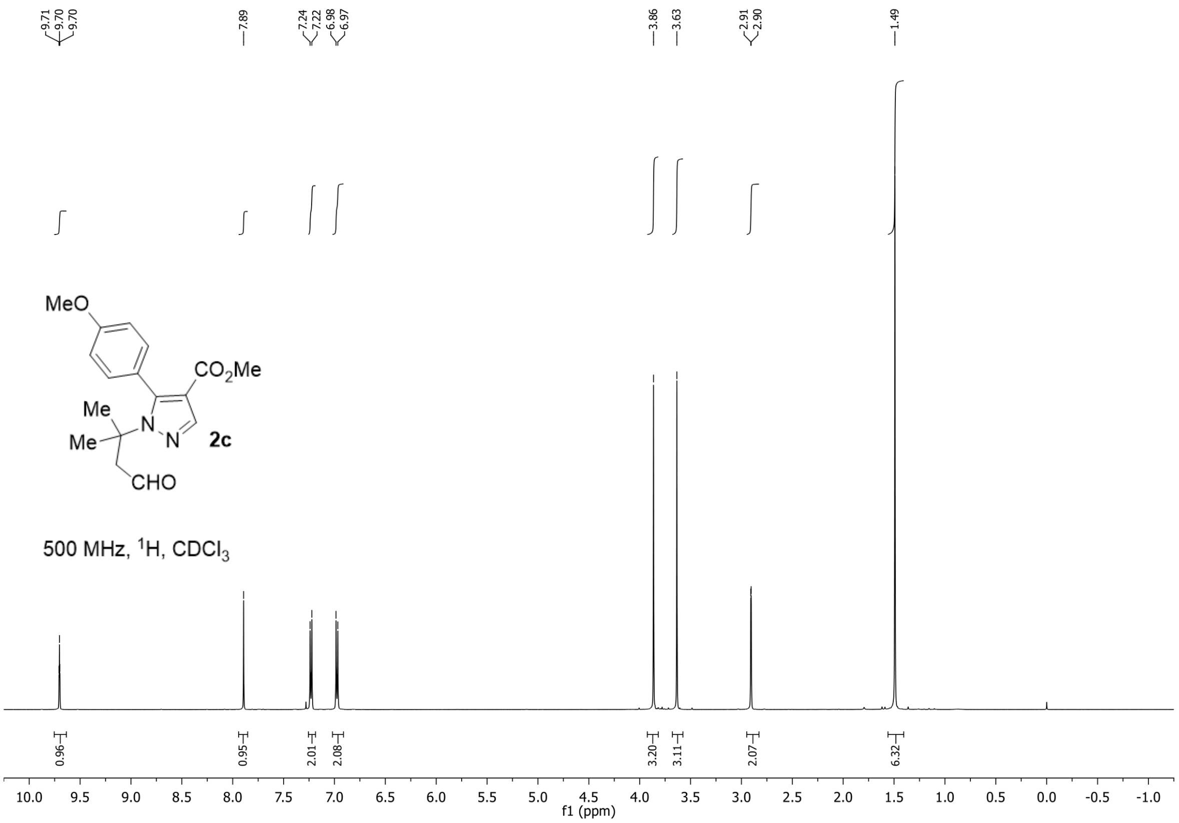

9.71
9.70
9.70
7.89
7.24
7.22
6.98
6.97
3.86
3.63
2.91
2.90
1.49
MeO
CO_2Me
Me
Me
N
N
2c
CHO
500 MHz, ^1H, $CDCl_3$
0.96
0.95
2.01
2.08
3.20
3.11
2.07
6.32
f1 (ppm)

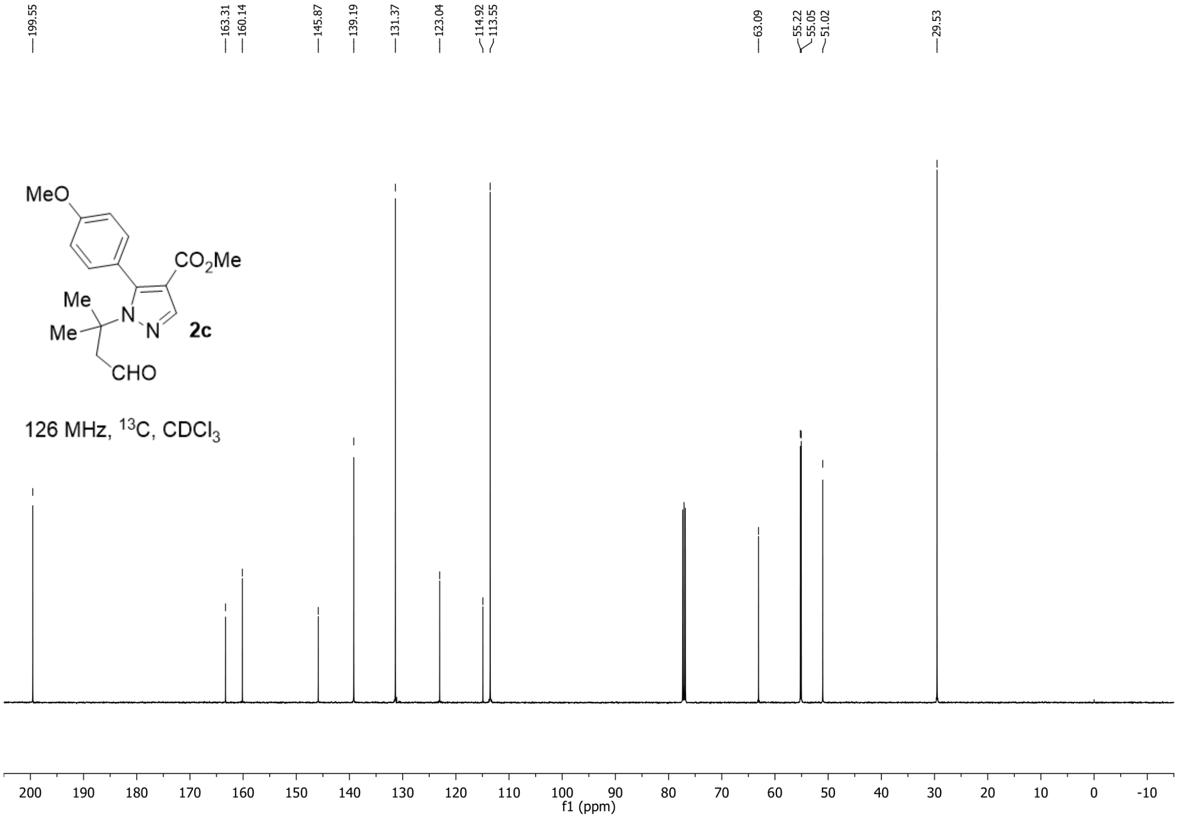

199.55
163.31
160.14
145.87
139.19
131.37
123.04
114.92
113.55
63.09
55.22
55.05
51.02
29.53
MeO
CO_2Me
Me
Me
N
N
2c
CHO
126 MHz, ^{13}C, $CDCl_3$
f1 (ppm)

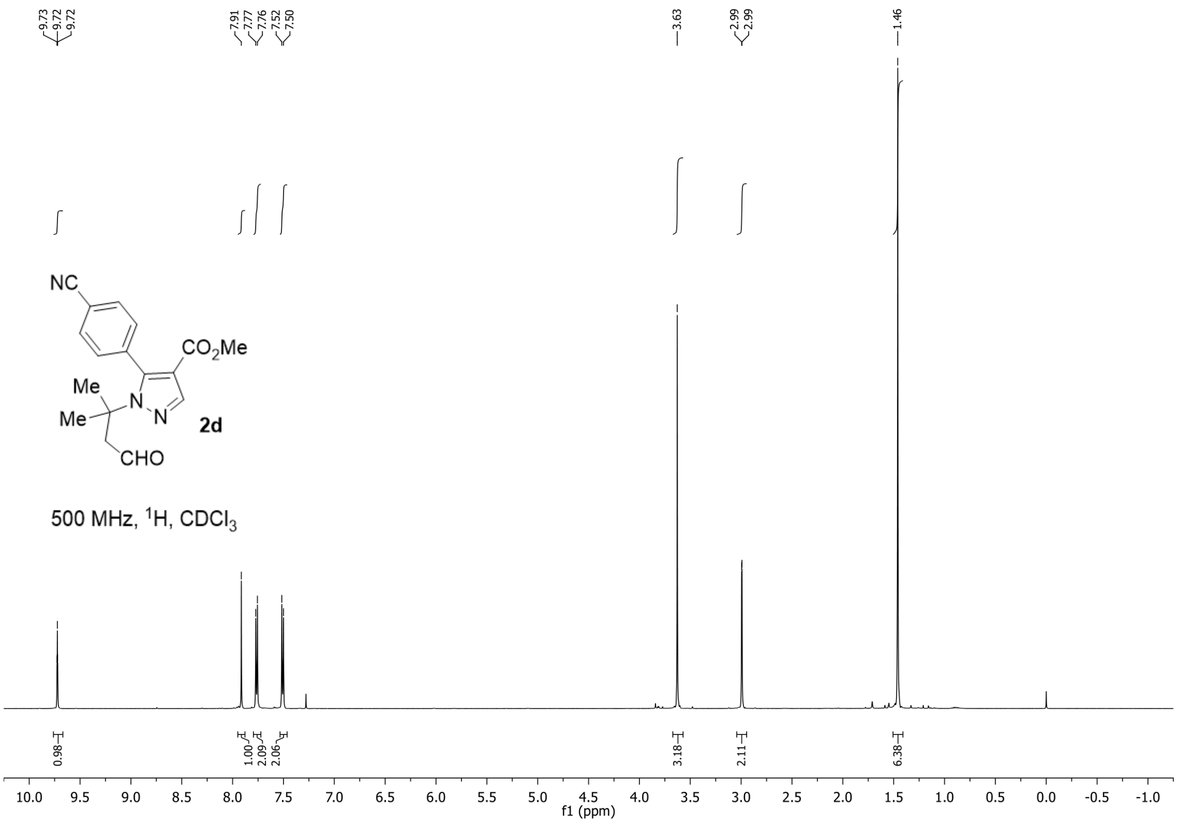
9.73
9.72
9.72
7.91
7.77
7.76
7.52
7.50
3.63
2.99
2.99
1.46
NC
CO2Me
Me
Me
N
N
2d
CHO
500 MHz, 1H, CDCl3
0.98
1.00
2.09
2.06
3.18
2.11
6.38
f1 (ppm)

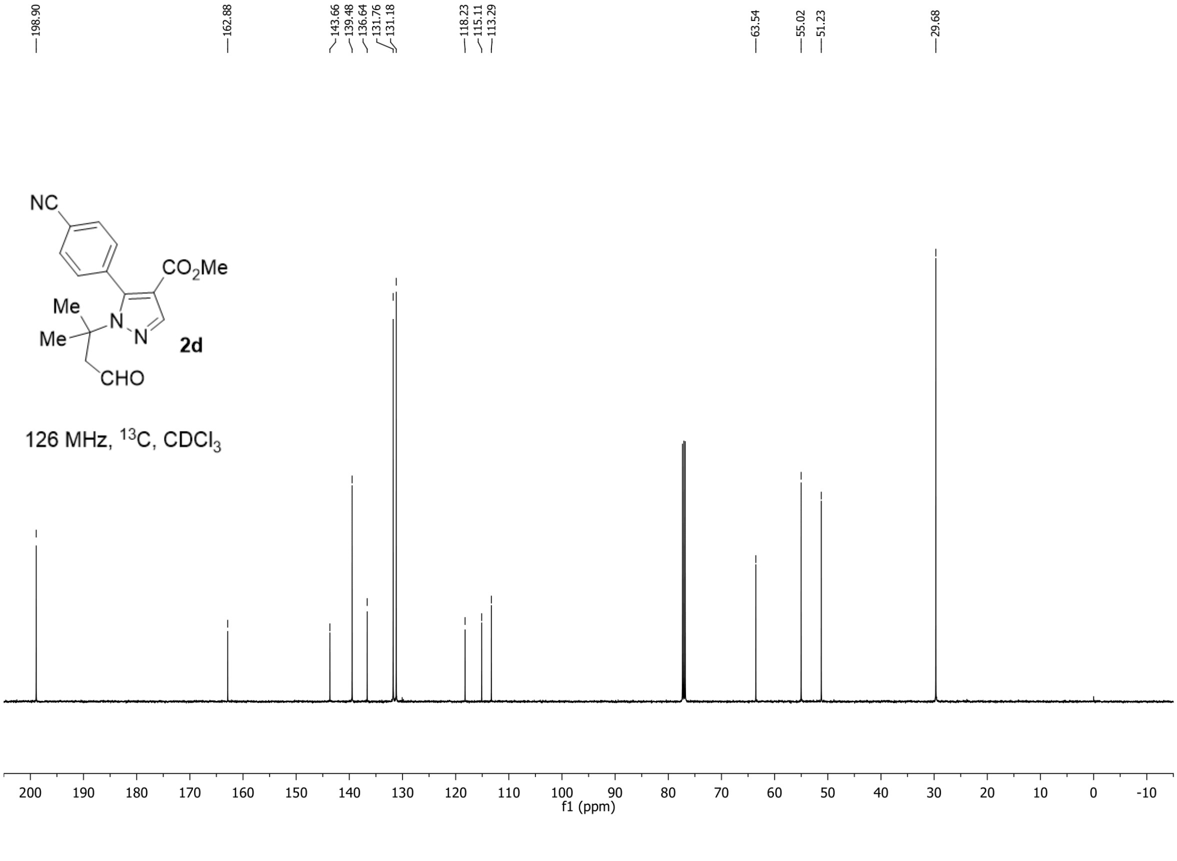
198.90
162.88
143.66
139.48
136.64
131.76
131.18
118.23
115.11
113.29
63.54
55.02
51.23
29.68
NC
CO2Me
Me
Me
N
N
2d
CHO
126 MHz, 13C, CDCl3
f1 (ppm)

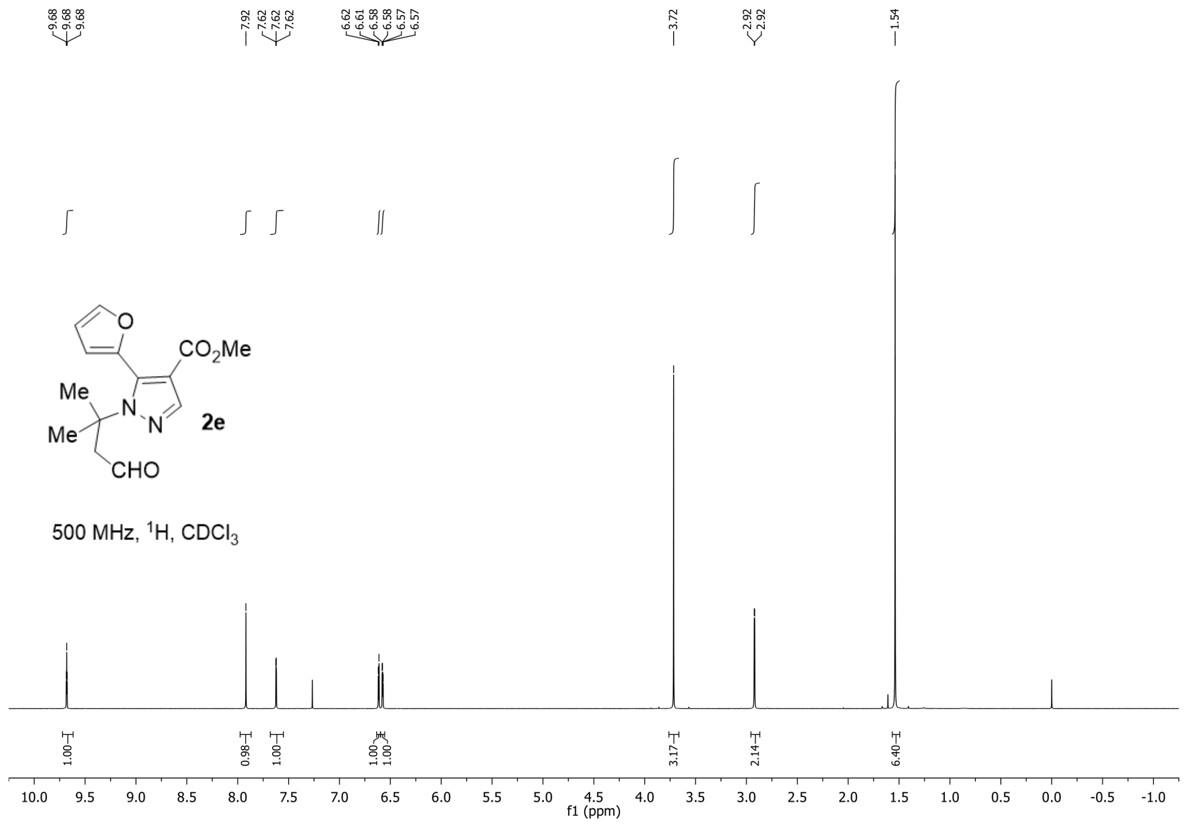
9.68
9.68
9.68
7.92
7.62
7.62
7.62
6.62
6.61
6.58
6.58
6.57
6.57
3.72
2.92
2.92
1.54
CO_2Me
Me
Me
N
N
O
CHO
2e
500 MHz, ^1H, $CDCl_3$
1.00
0.98
1.00
1.00
1.00
3.17
2.14
6.40
f1 (ppm)

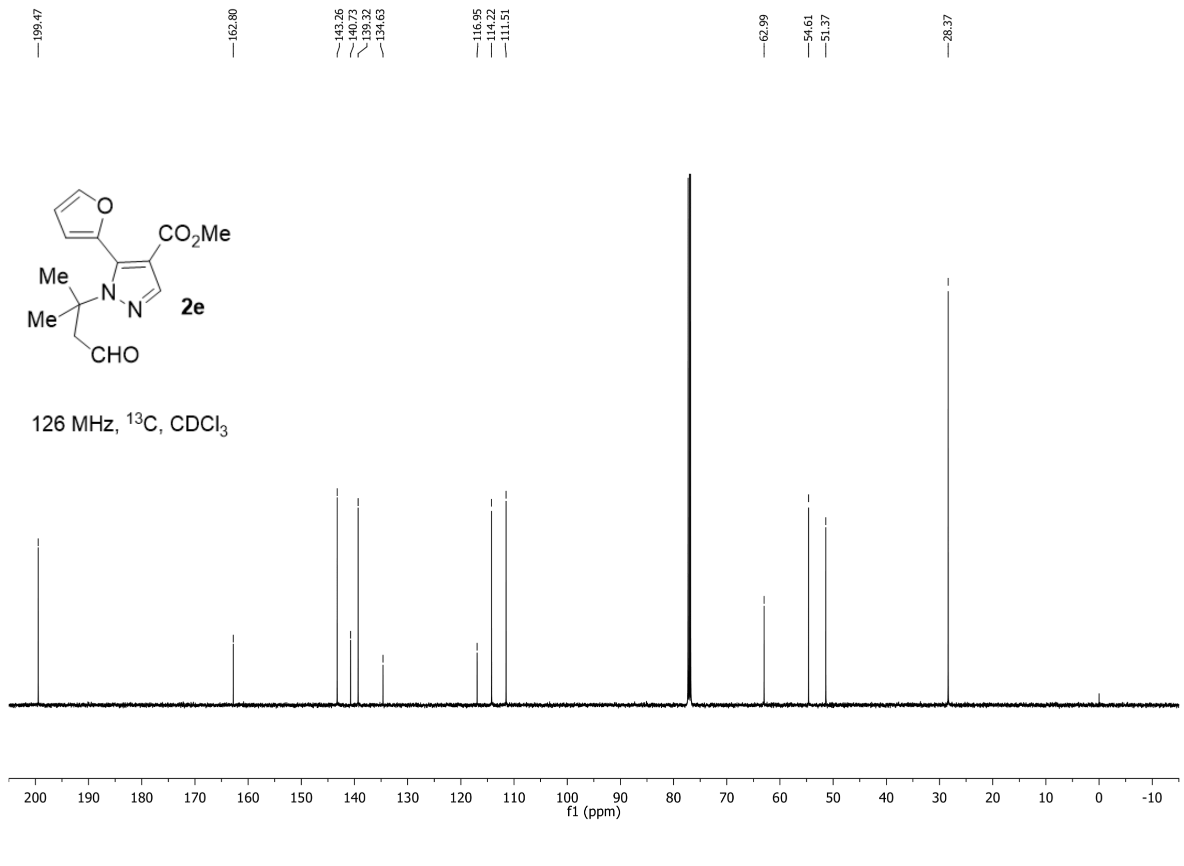
199.47
162.80
143.26
140.73
139.32
134.63
116.95
114.22
111.51
62.99
54.61
51.37
28.37
CO_2Me
Me
Me
N
N
O
CHO
2e
126 MHz, ^{13}C, $CDCl_3$
f1 (ppm)

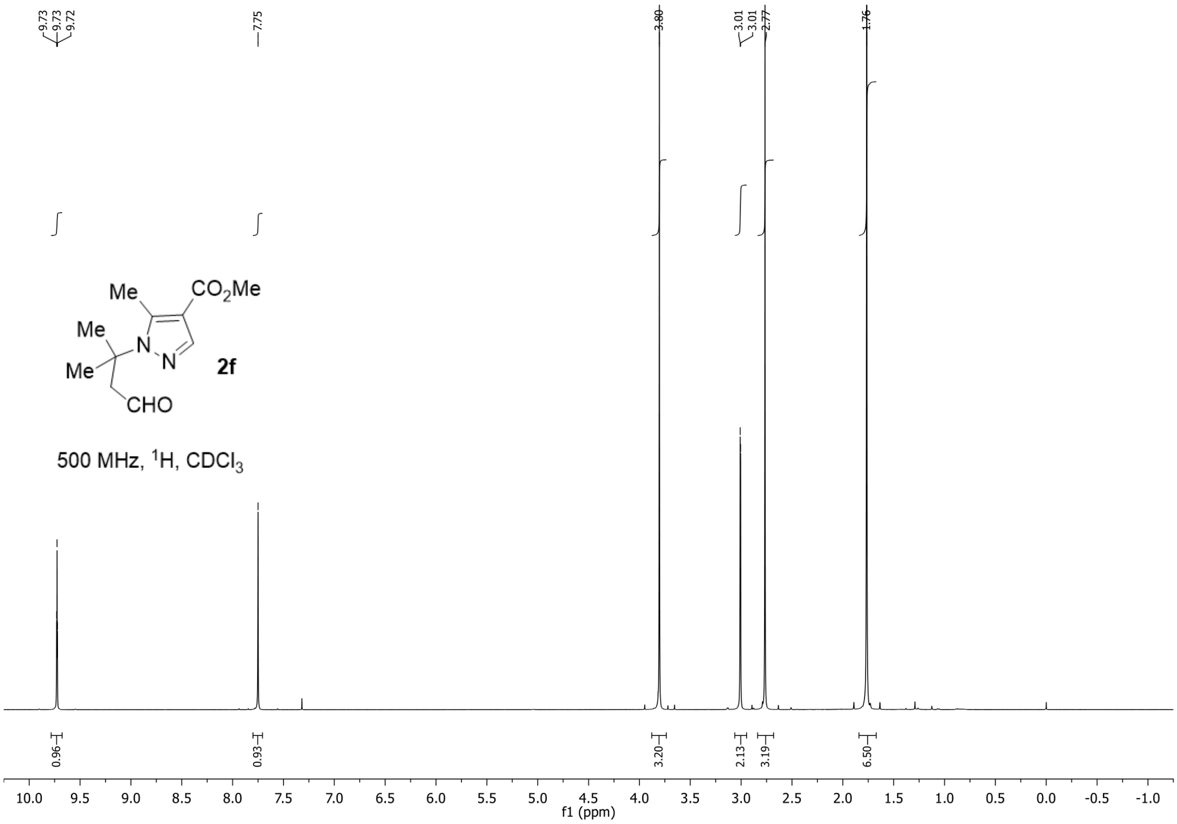

9.73
9.73
9.72
7.75
3.80
3.01
3.01
2.77
1.76
Me
CO2Me
Me
N
N
Me
2f
CHO
500 MHz, 1H, CDCl3
0.96
0.93
3.20
2.13
3.19
6.50
10.0 9.5 9.0 8.5 8.0 7.5 7.0 6.5 6.0 5.5 5.0 4.5 4.0 3.5 3.0 2.5 2.0 1.5 1.0 0.5 0.0 -0.5 -1.0
f1 (ppm)

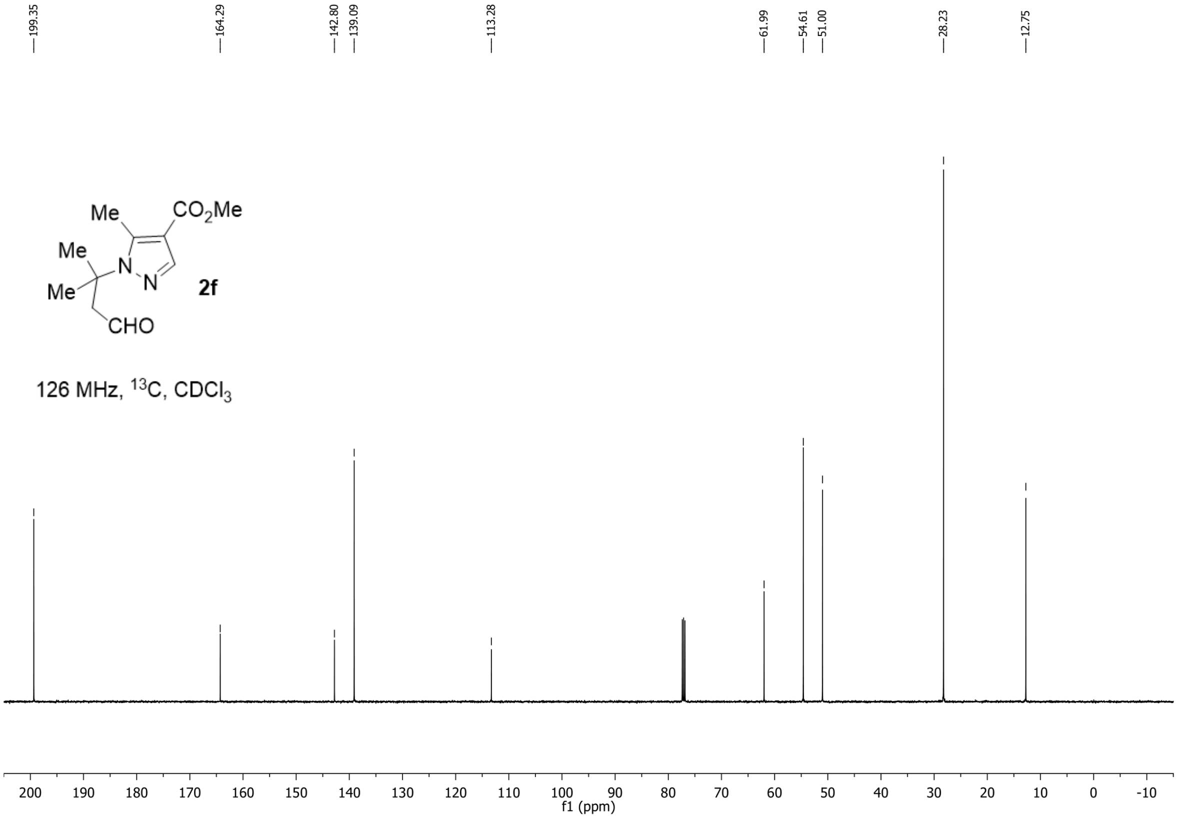

199.35
164.29
142.80
139.09
113.28
61.99
54.61
51.00
28.23
12.75
Me
CO2Me
Me
N
N
Me
2f
CHO
126 MHz, 13C, CDCl3
200 190 180 170 160 150 140 130 120 110 100 90 80 70 60 50 40 30 20 10 0 -10
f1 (ppm)

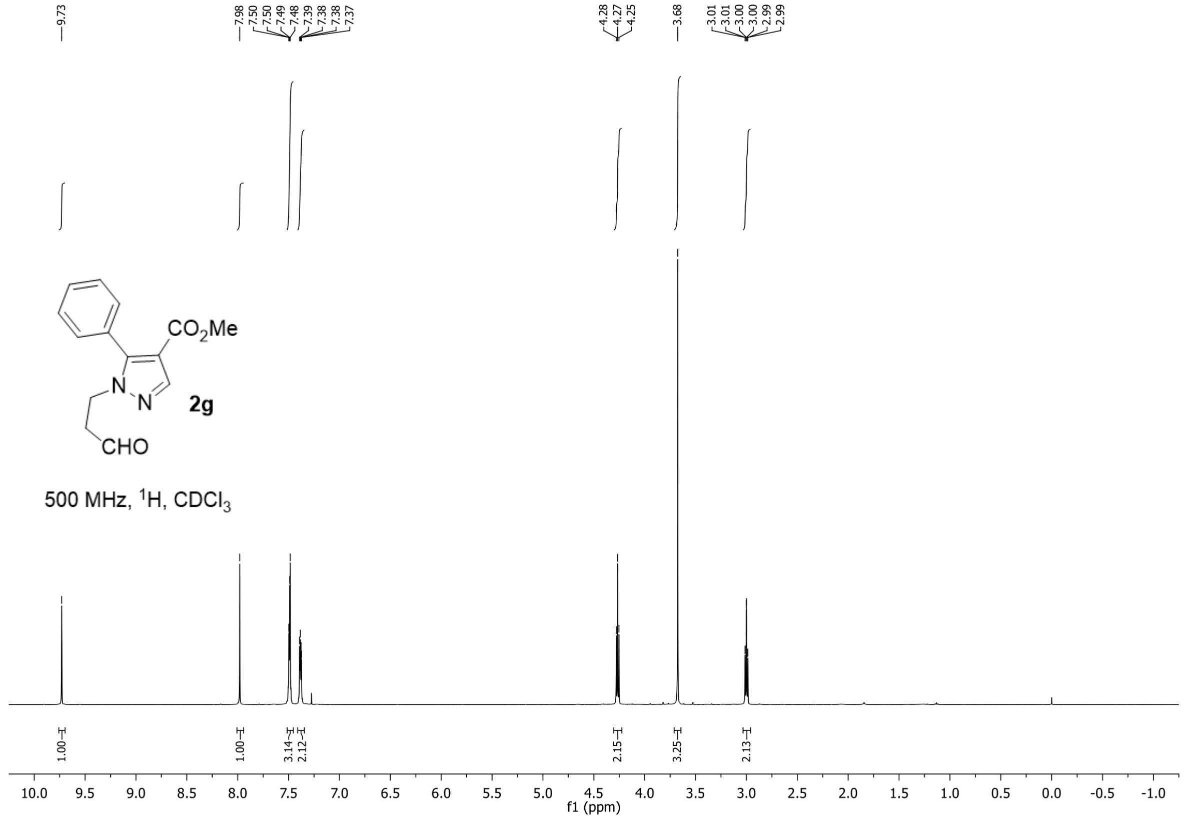
9.73
7.98 7.50 7.50 7.49 7.48 7.39 7.38 7.38 7.37
4.28 4.27 4.25
3.68
3.01 3.01 3.00 3.00 2.99 2.99
CO_2Me
N
N
2g
CHO
500 MHz, ^1H, $CDCl_3$
1.00
1.00
3.14
2.12
2.15
3.25
2.13
f1 (ppm)

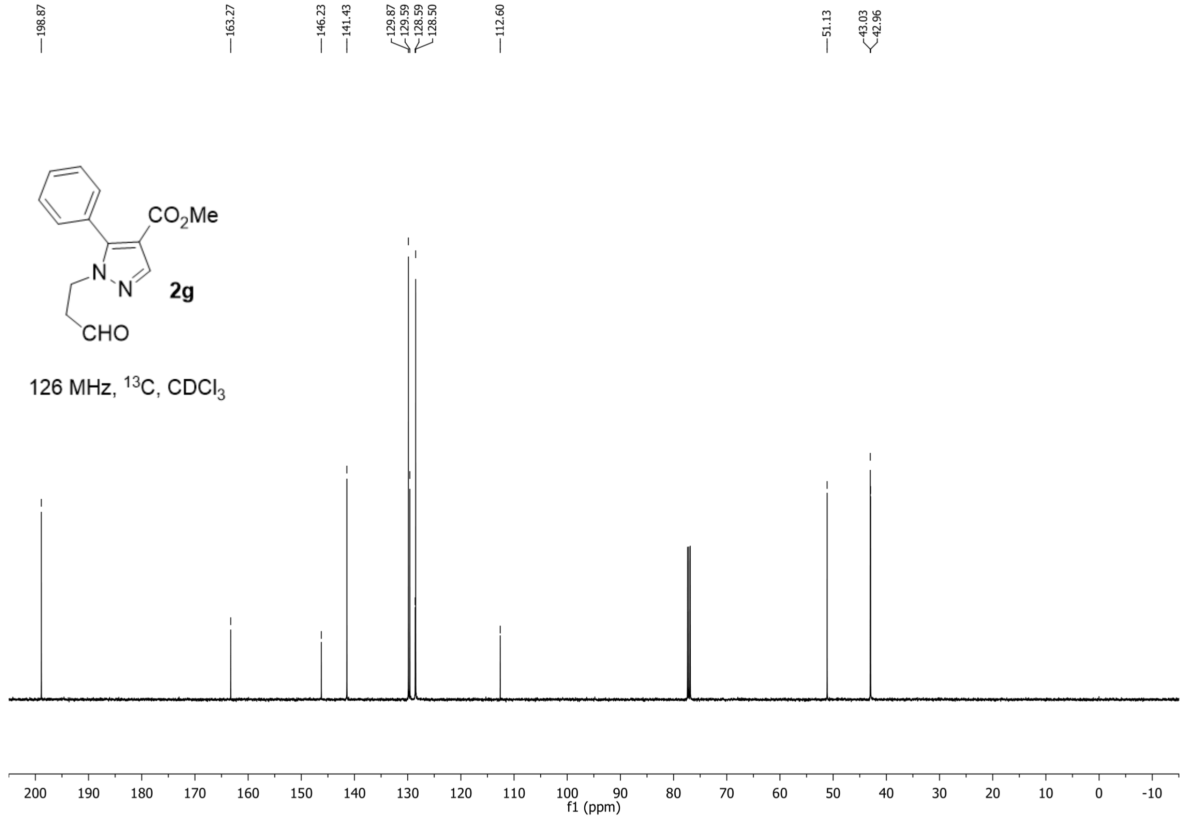
198.87
163.27
146.23
141.43
129.87 129.59 128.59 128.50
112.60
51.13
43.03 42.96
CO_2Me
N
N
2g
CHO
126 MHz, ^{13}C, $CDCl_3$
f1 (ppm)

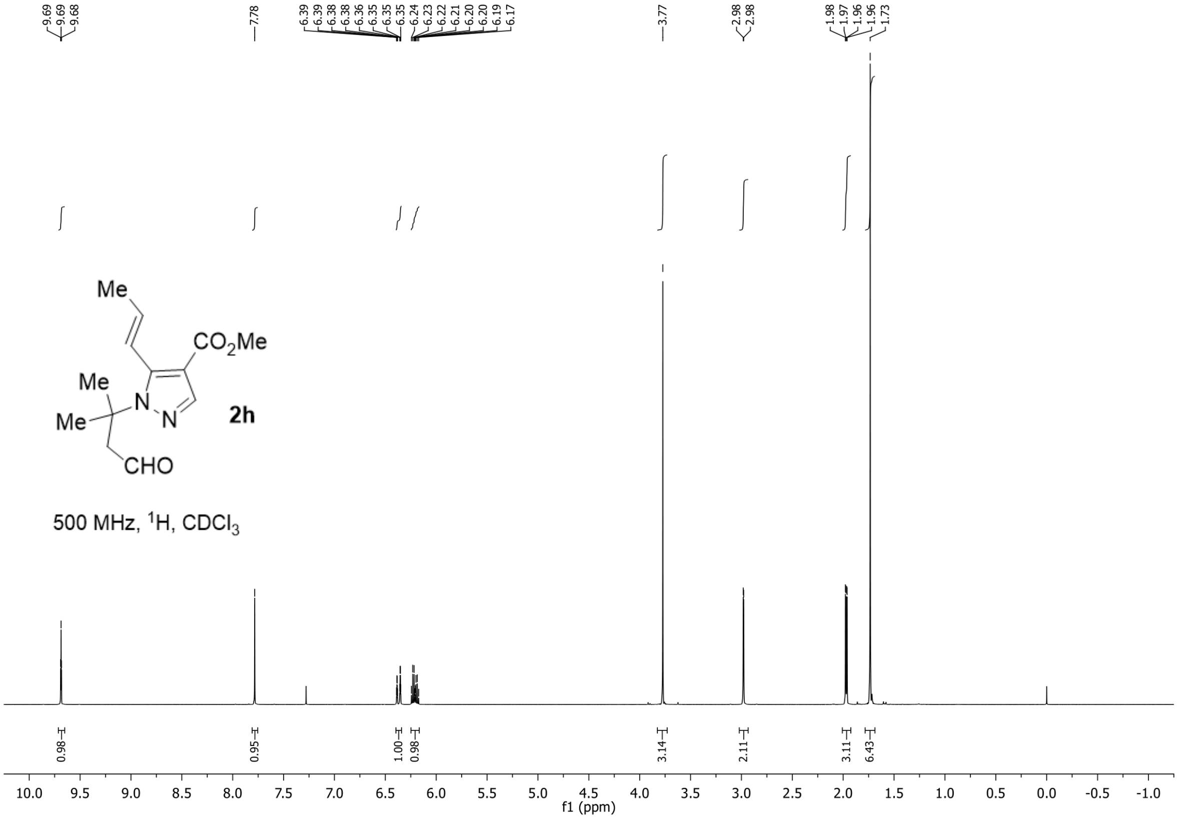

9.69
9.69
9.68
7.78
6.39
6.39
6.38
6.38
6.36
6.35
6.35
6.35
6.24
6.23
6.22
6.21
6.20
6.20
6.19
6.17
3.77
2.98
2.98
1.98
1.97
1.96
1.96
1.73
Me
CO2Me
Me
N
Me
N
2h
CHO
500 MHz, 1H, CDCl3
0.98
0.95
1.00
0.98
3.14
2.11
3.11
6.43
10.0 9.5 9.0 8.5 8.0 7.5 7.0 6.5 6.0 5.5 5.0 4.5 4.0 3.5 3.0 2.5 2.0 1.5 1.0 0.5 0.0 -0.5 -1.0
f1 (ppm)

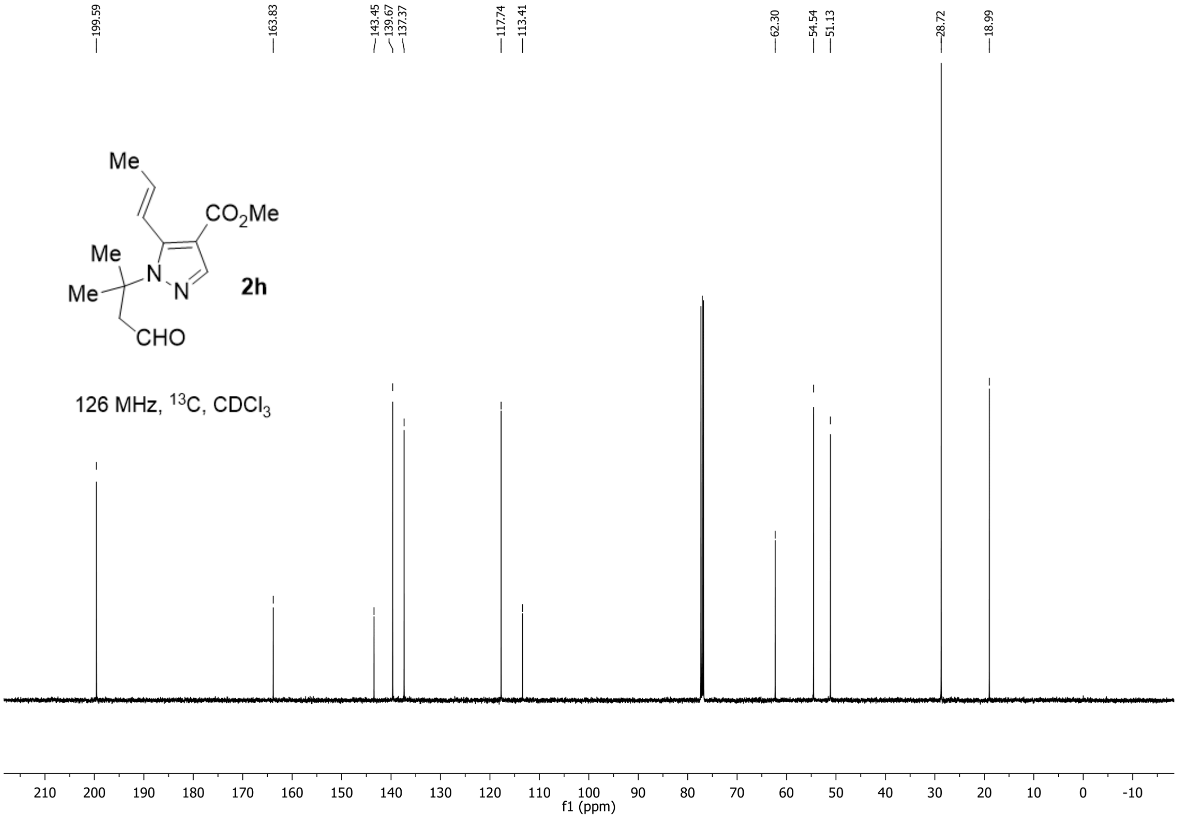

199.59
163.83
143.45
139.67
137.37
117.74
113.41
62.30
54.54
51.13
28.72
18.99
Me
CO2Me
Me
N
Me
N
2h
CHO
126 MHz, 13C, CDCl3
210 200 190 180 170 160 150 140 130 120 110 100 90 80 70 60 50 40 30 20 10 0 -10
f1 (ppm)

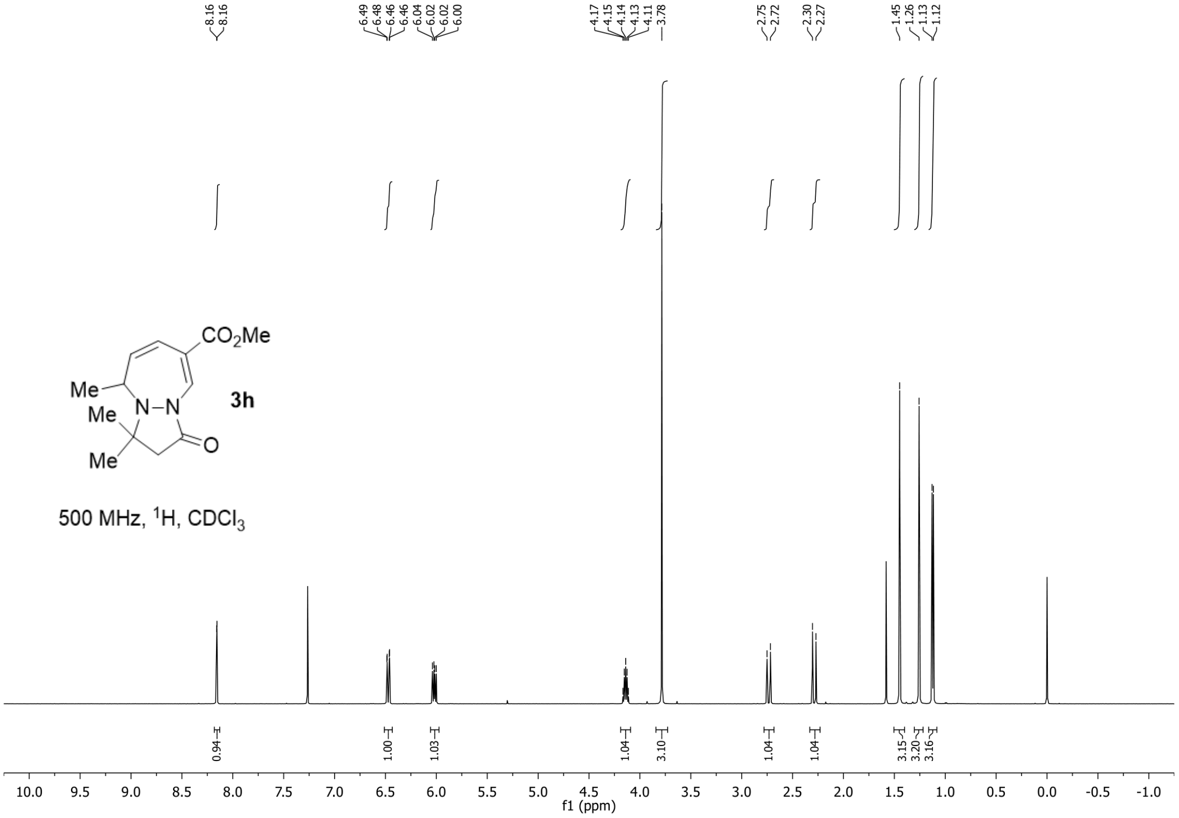
8.16
8.16
6.49
6.48
6.46
6.46
6.04
6.02
6.02
6.00
4.17
4.15
4.14
4.13
4.11
3.78
2.75
2.72
2.30
2.27
1.45
1.26
1.13
1.12
CO2Me
Me
N-N
Me
Me
O
3h
500 MHz, 1H, CDCl3
0.94
1.00
1.03
1.04
3.10
1.04
1.04
3.15
3.20
3.16
f1 (ppm)

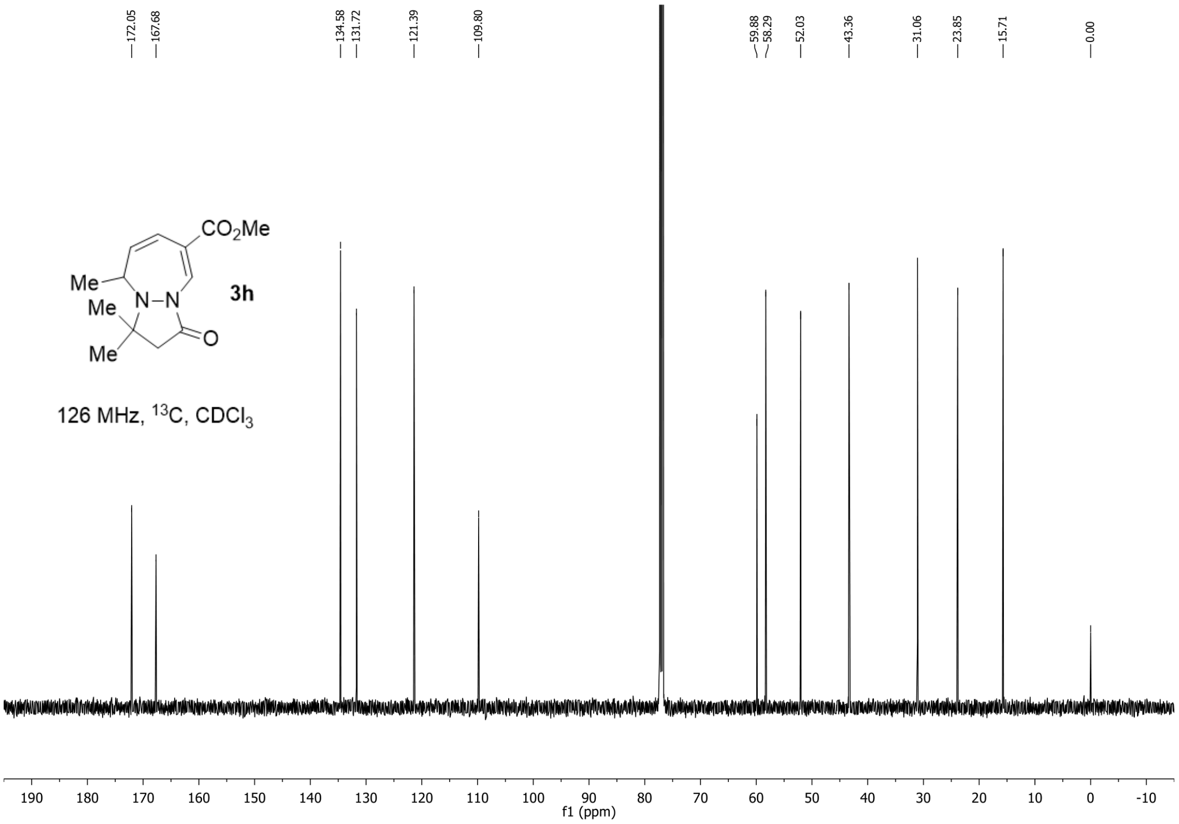
172.05
167.68
134.58
131.72
121.39
109.80
59.88
58.29
52.03
43.36
31.06
23.85
15.71
0.00
CO2Me
Me
N-N
Me
Me
O
3h
126 MHz, 13C, CDCl3
f1 (ppm)

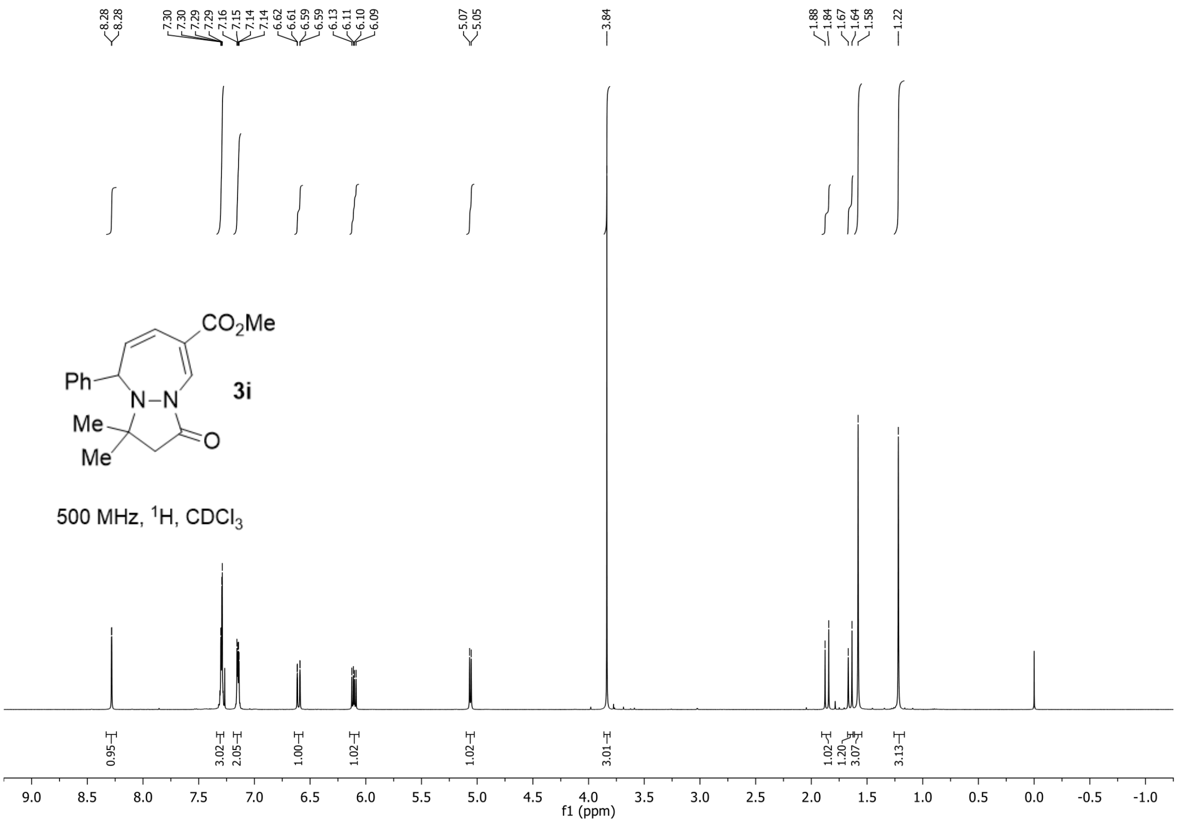
3i
500 MHz, 1H, CDCl3
f1 (ppm)

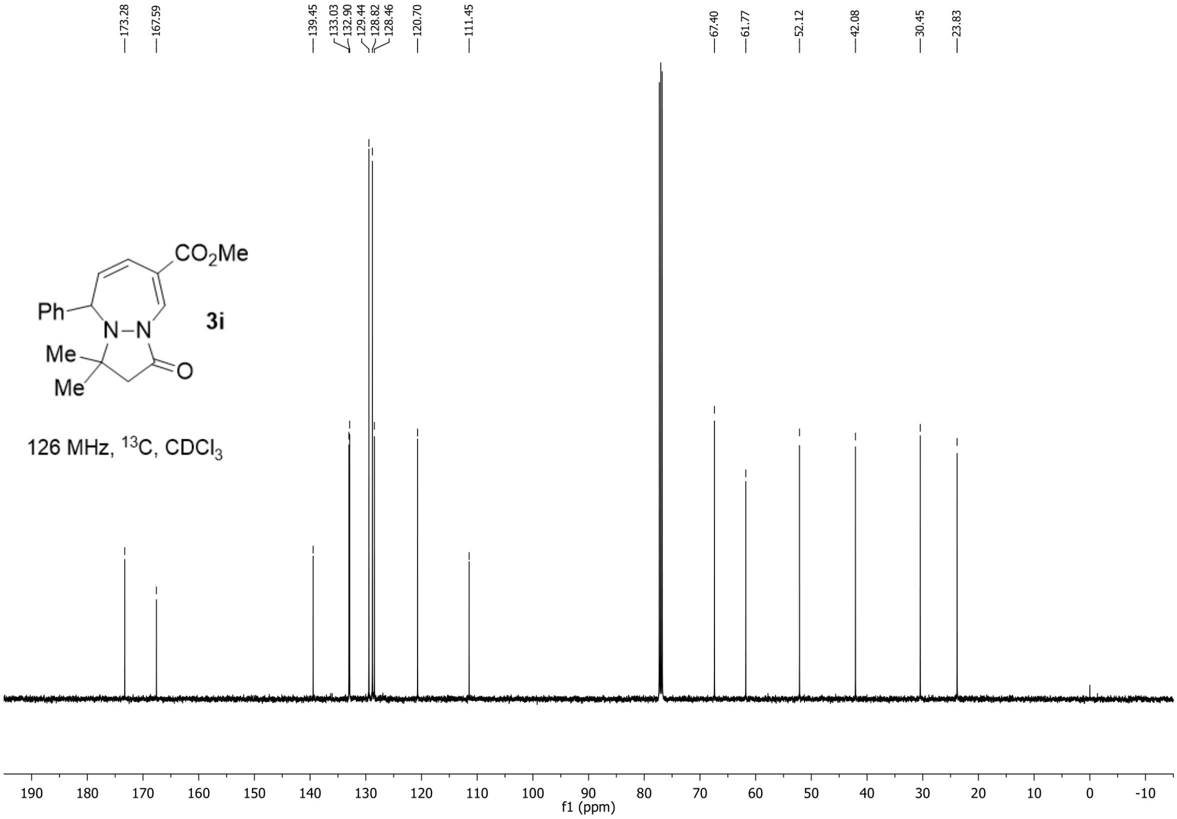
3i
126 MHz, 13C, CDCl3
f1 (ppm)

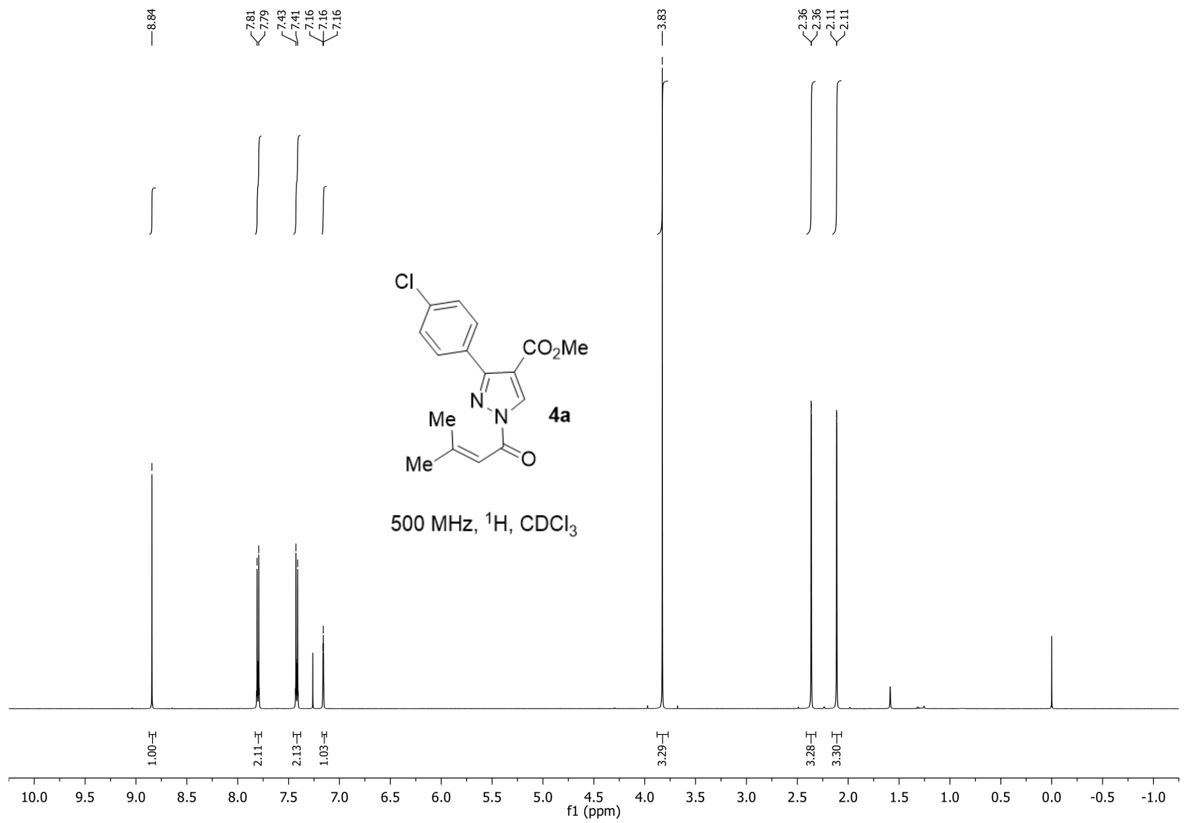

8.84
7.81
7.79
7.43
7.41
7.16
7.16
7.16
3.83
2.36
2.36
2.11
2.11
Cl
CO2Me
N
N
Me
Me
O
4a
500 MHz, 1H, CDCl3
1.00
2.11
2.13
1.03
3.29
3.28
3.30
10.0
9.5
9.0
8.5
8.0
7.5
7.0
6.5
6.0
5.5
5.0
4.5
4.0
3.5
3.0
2.5
2.0
1.5
1.0
0.5
0.0
-0.5
-1.0
f1 (ppm)

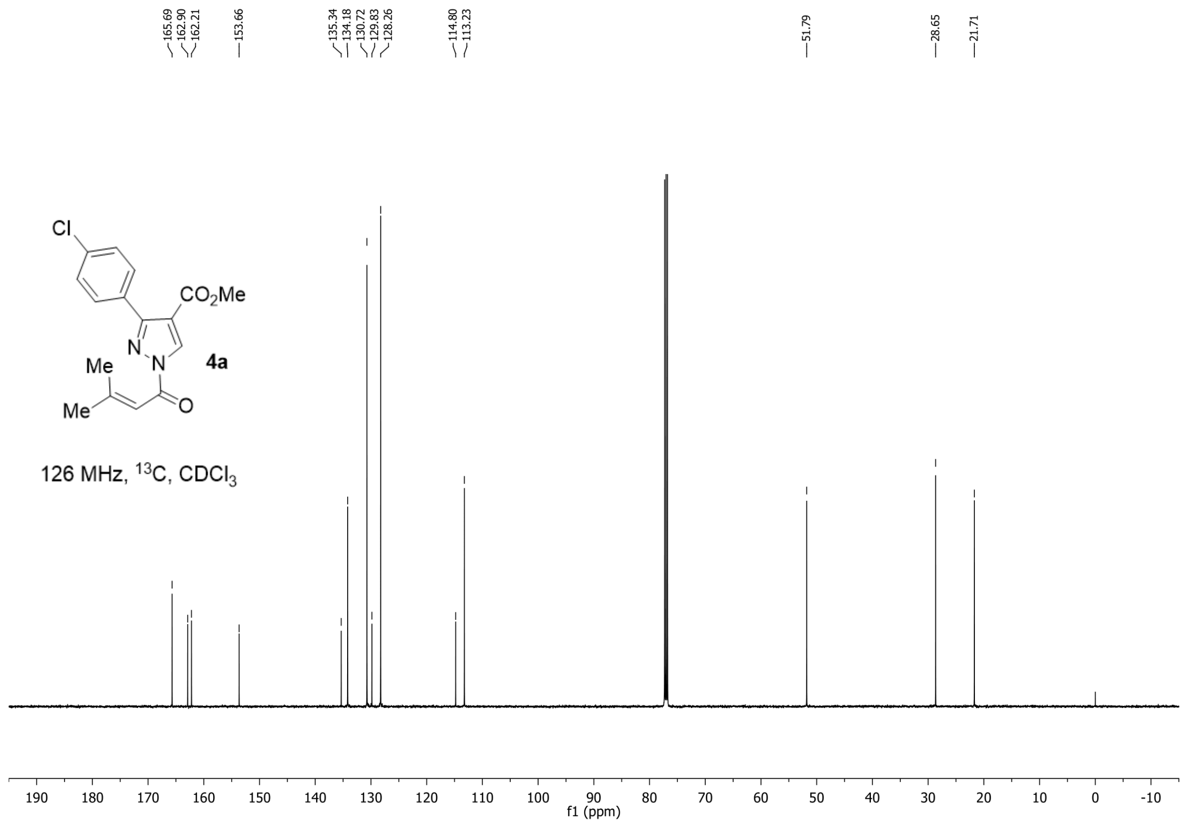

165.69
162.90
162.21
153.66
135.34
134.18
130.72
129.83
128.26
114.80
113.23
51.79
28.65
21.71
Cl
CO2Me
N
N
Me
Me
O
4a
126 MHz, 13C, CDCl3
190
180
170
160
150
140
130
120
110
100
90
80
70
60
50
40
30
20
10
0
-10
f1 (ppm)

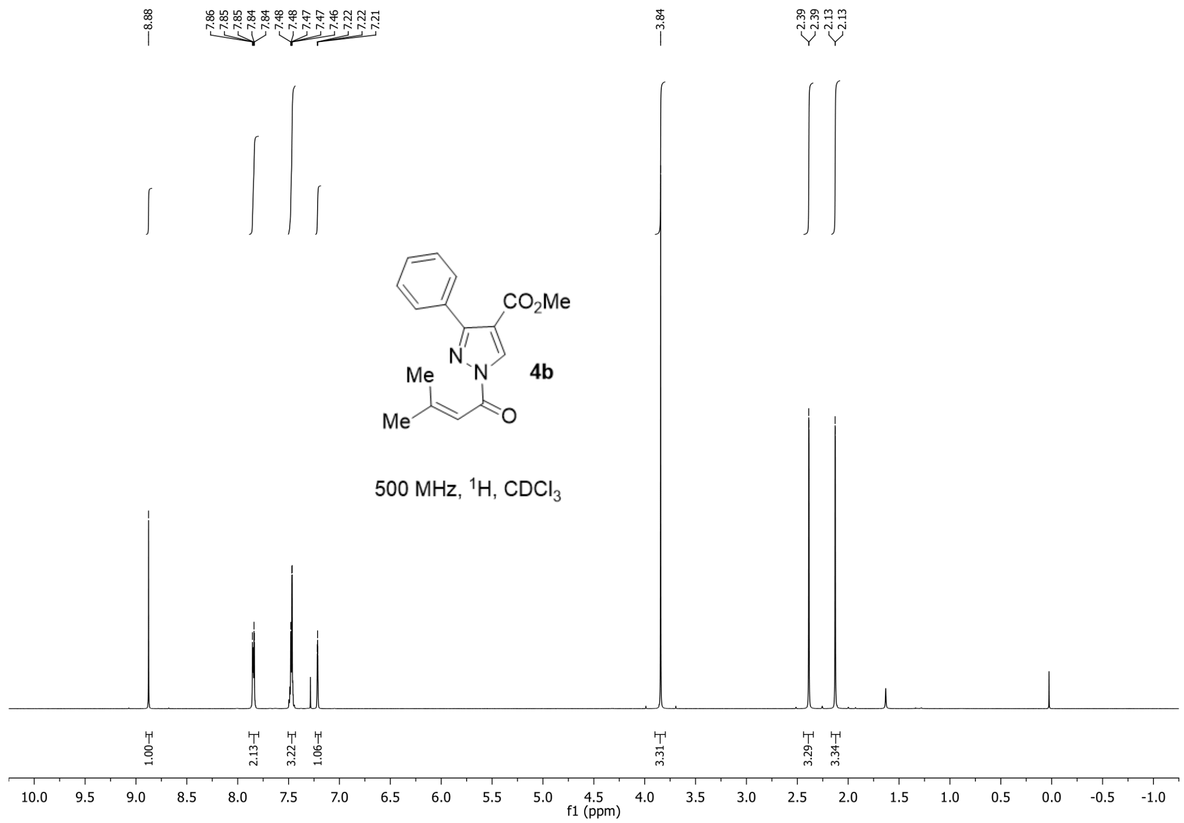
8.88
7.86
7.85
7.85
7.84
7.84
7.48
7.48
7.47
7.47
7.46
7.22
7.22
7.22
7.21
3.84
2.39
2.39
2.13
2.13
CO2Me
N
N
Me
Me
O
4b
500 MHz, 1H, CDCl3
1.00
2.13
3.22
1.06
3.31
3.29
3.34
f1 (ppm)

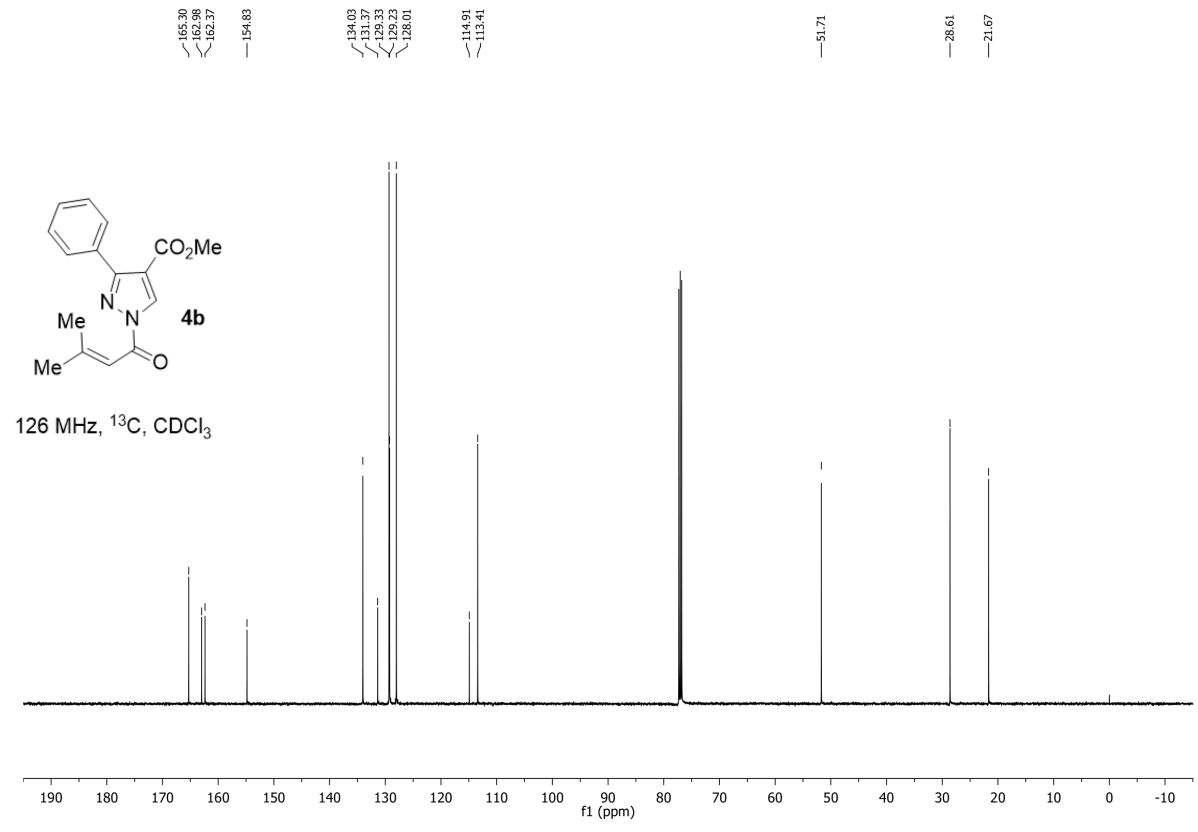
165.30
162.98
162.37
154.83
134.03
131.37
129.33
129.23
128.01
114.91
113.41
51.71
28.61
21.67
CO2Me
N
N
Me
Me
O
4b
126 MHz, 13C, CDCl3
f1 (ppm)

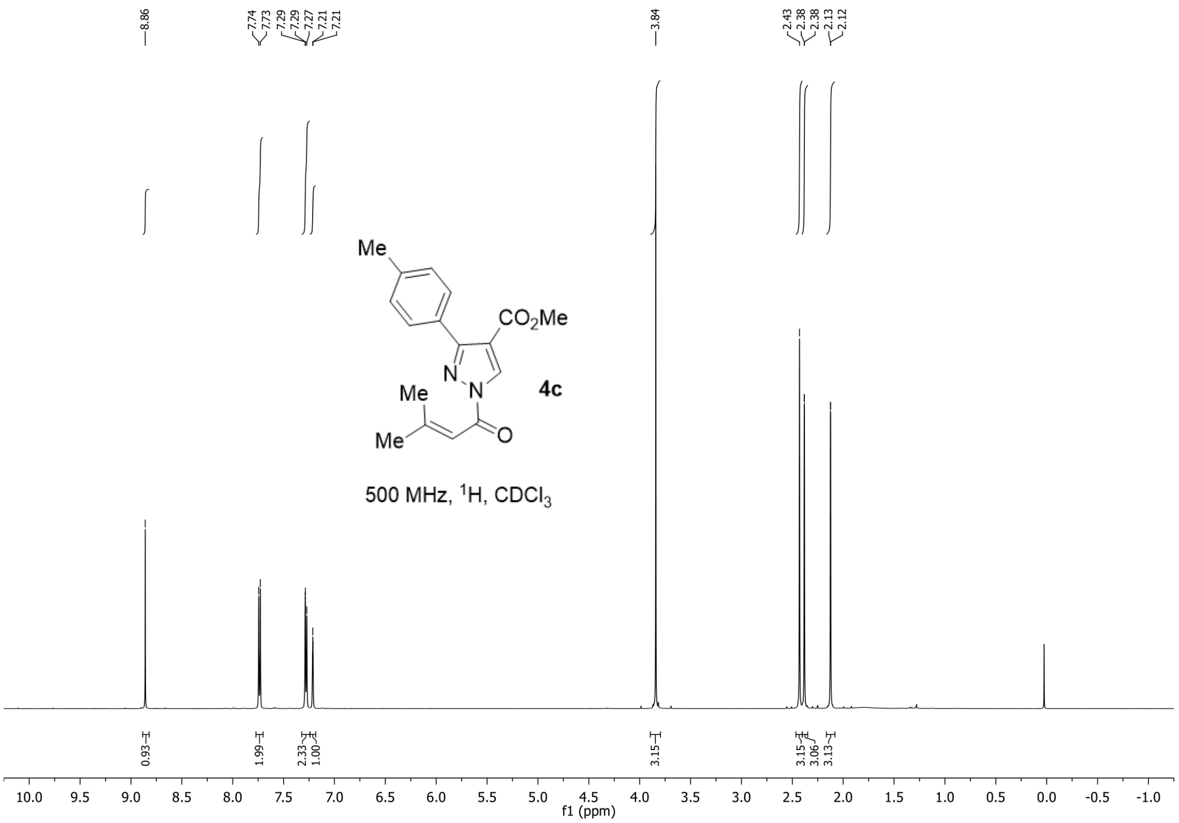

8.86
7.74
7.73
7.29
7.29
7.27
7.21
7.21
3.84
2.43
2.38
2.38
2.13
2.12
Me
CO2Me
N
N
Me
Me
O
4c
500 MHz, 1H, CDCl3
0.93
1.99
2.33
1.00
3.15
3.15
3.06
3.13
f1 (ppm)

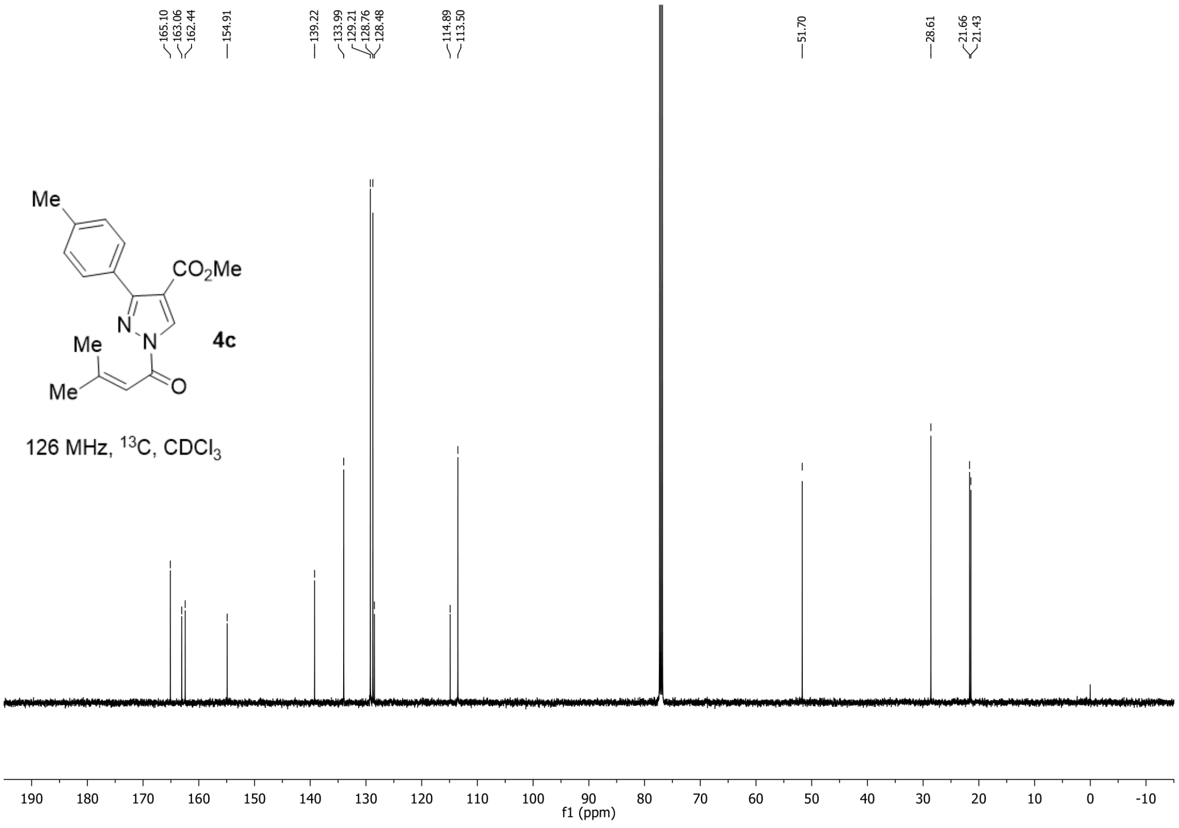

165.10
163.06
162.44
154.91
139.22
133.99
129.21
128.76
128.48
114.89
113.50
51.70
28.61
21.66
21.43
Me
CO2Me
N
N
Me
Me
O
4c
126 MHz, 13C, CDCl3
f1 (ppm)

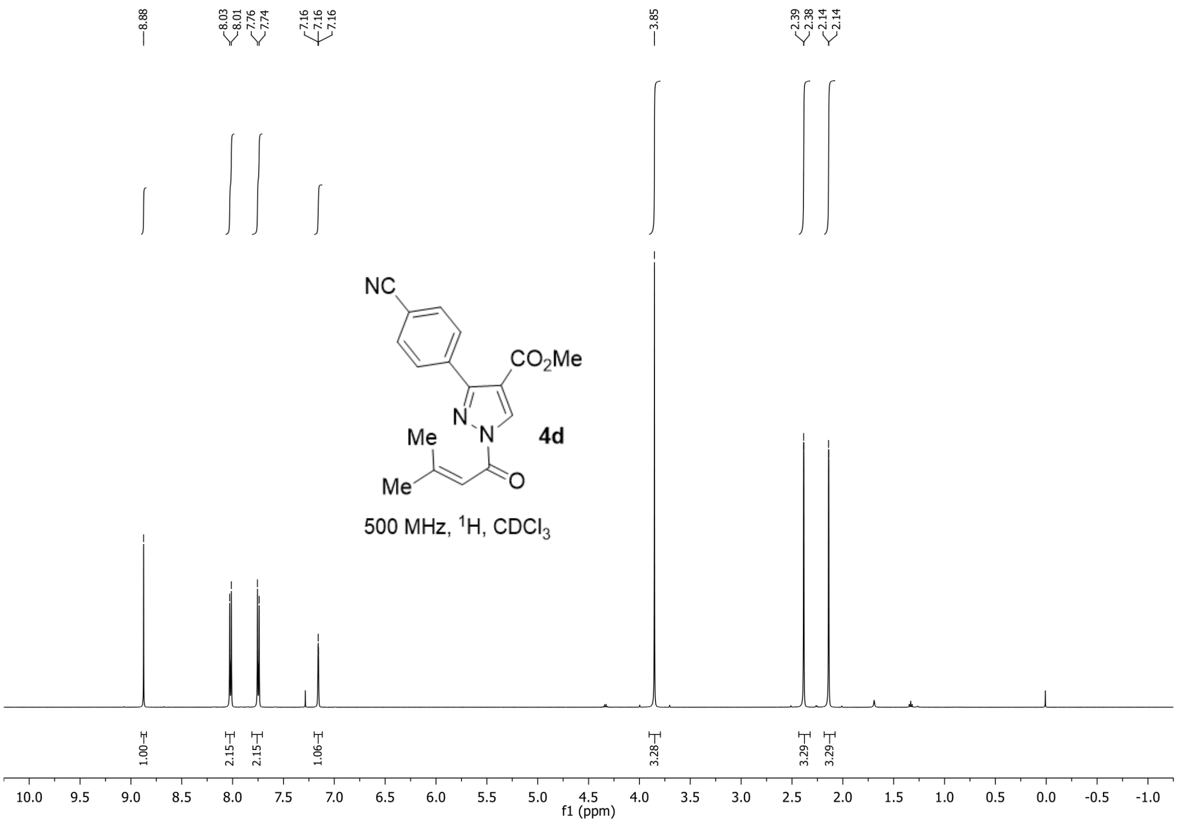
8.88
8.03
8.01
7.76
7.74
7.16
7.16
7.16
3.85
2.39
2.38
2.14
2.14
NC
CO2Me
N
N
Me
Me
O
4d
500 MHz, 1H, CDCl3
1.00
2.15
2.15
1.06
3.28
3.29
3.29
10.0
9.5
9.0
8.5
8.0
7.5
7.0
6.5
6.0
5.5
5.0
4.5
4.0
3.5
3.0
2.5
2.0
1.5
1.0
0.5
0.0
-0.5
-1.0
f1 (ppm)

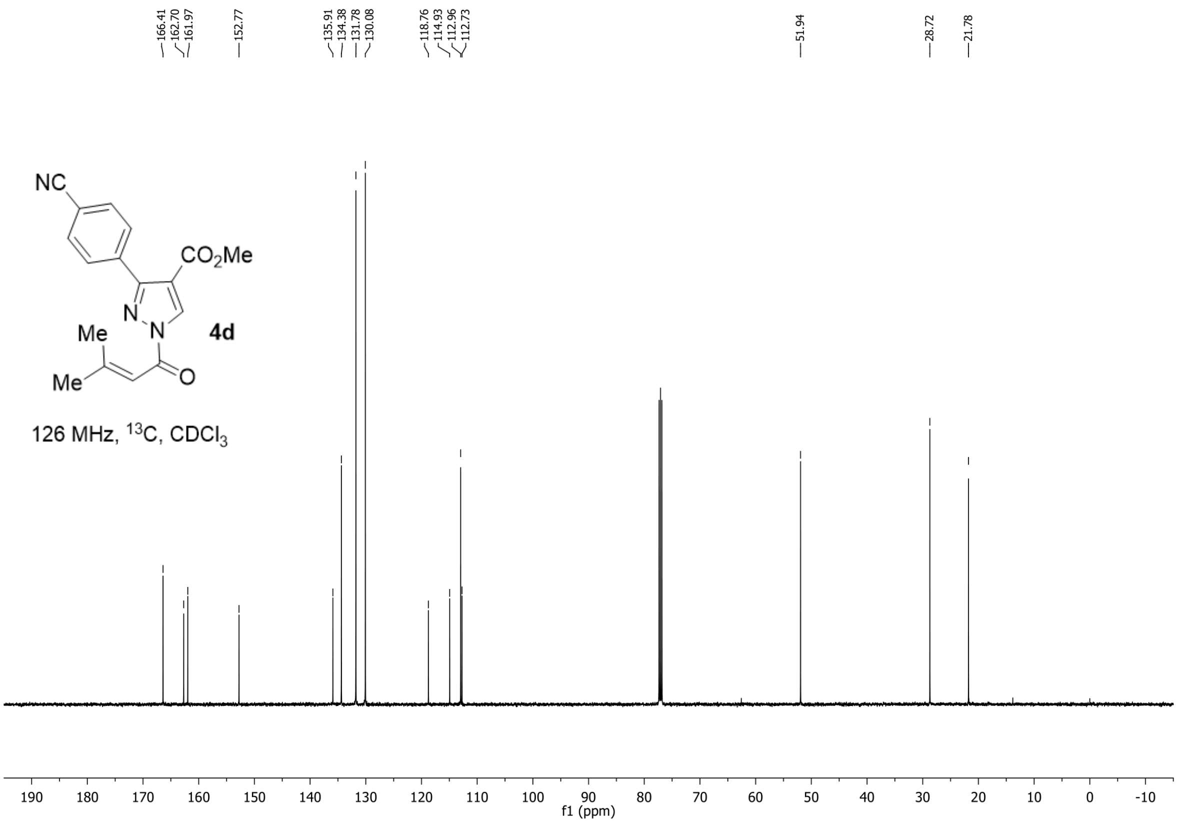
166.41
162.70
161.97
152.77
135.91
134.38
131.78
130.08
118.76
114.93
112.96
112.73
51.94
28.72
21.78
NC
CO2Me
N
N
Me
Me
O
4d
126 MHz, 13C, CDCl3
190
180
170
160
150
140
130
120
110
100
90
80
70
60
50
40
30
20
10
0
-10
f1 (ppm)

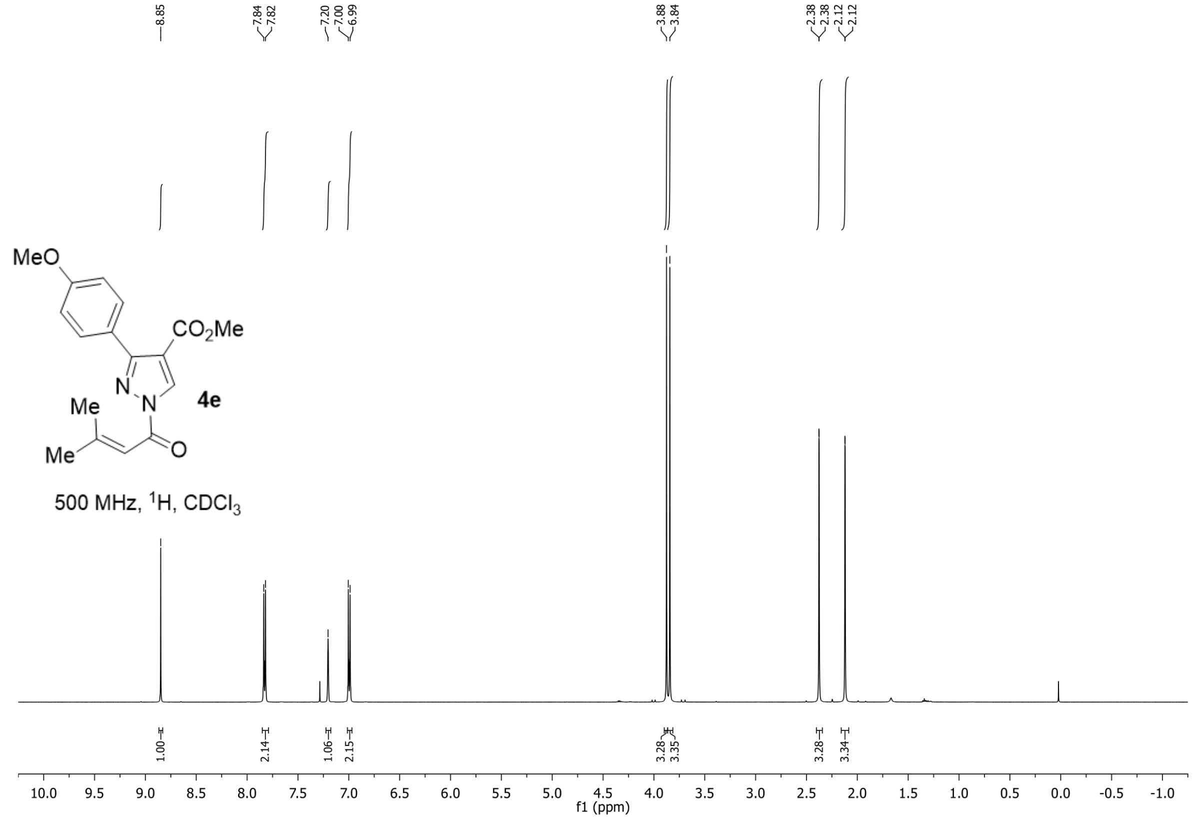
8.85
7.84
7.82
7.20
7.00
6.99
3.88
3.84
2.38
2.38
2.12
2.12
MeO
CO2Me
N
N
Me
Me
O
4e
500 MHz, 1H, CDCl3
1.00
2.14
1.06
2.15
3.28
3.35
3.28
3.34
f1 (ppm)

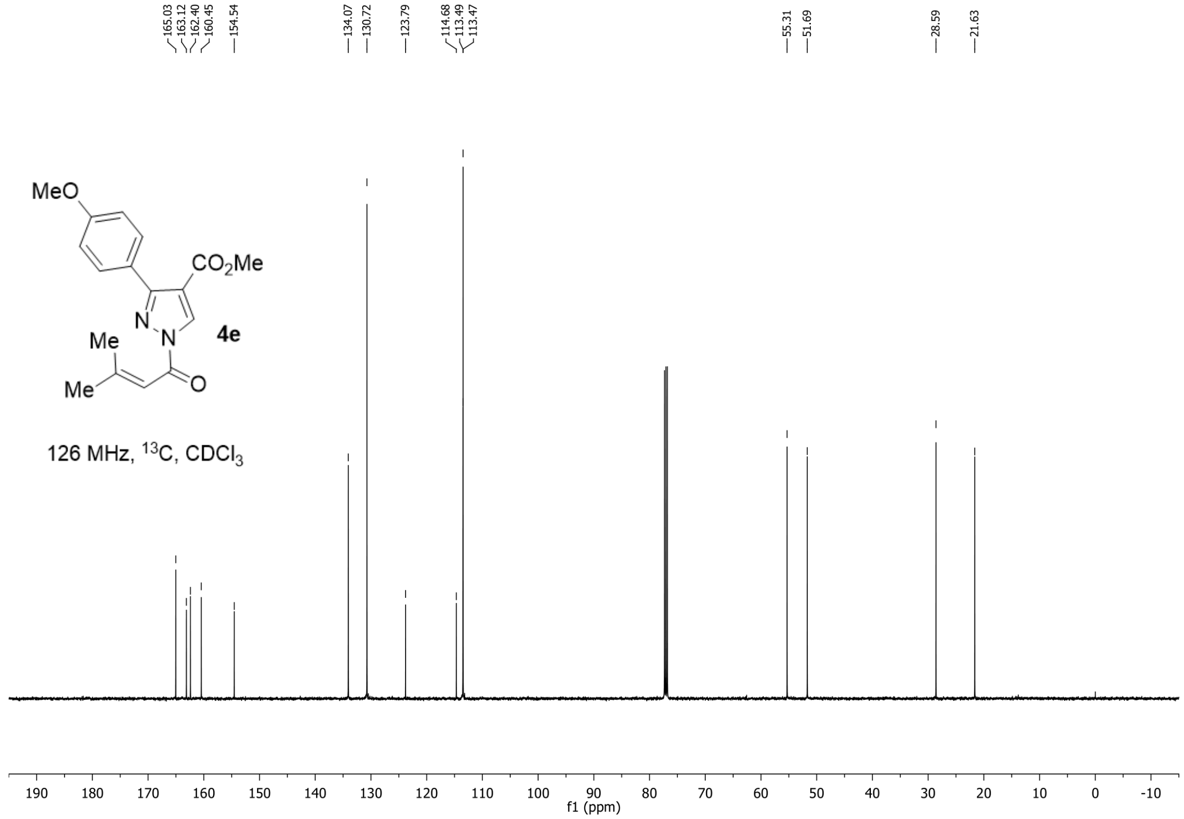
165.03
163.12
162.40
160.45
154.54
134.07
130.72
123.79
114.68
113.49
113.47
55.31
51.69
28.59
21.63
MeO
CO2Me
N
N
Me
Me
O
4e
126 MHz, 13C, CDCl3
f1 (ppm)

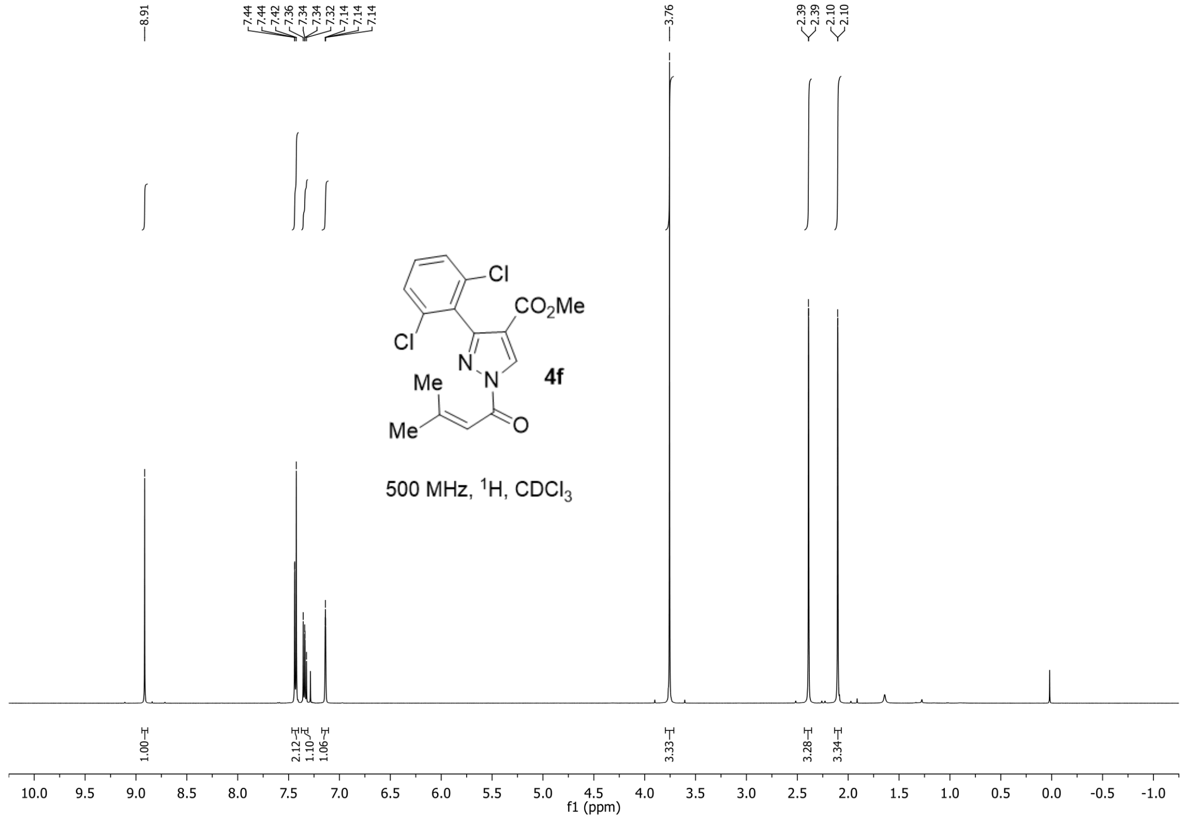

8.91
7.44
7.44
7.42
7.36
7.34
7.34
7.32
7.14
7.14
7.14
3.76
2.39
2.39
2.10
2.10
Cl
CO_2Me
Cl
N
N
Me
Me
O
4f
500 MHz, ^1H, $CDCl_3$
1.00
2.12
1.10
1.06
3.33
3.28
3.34
f1 (ppm)

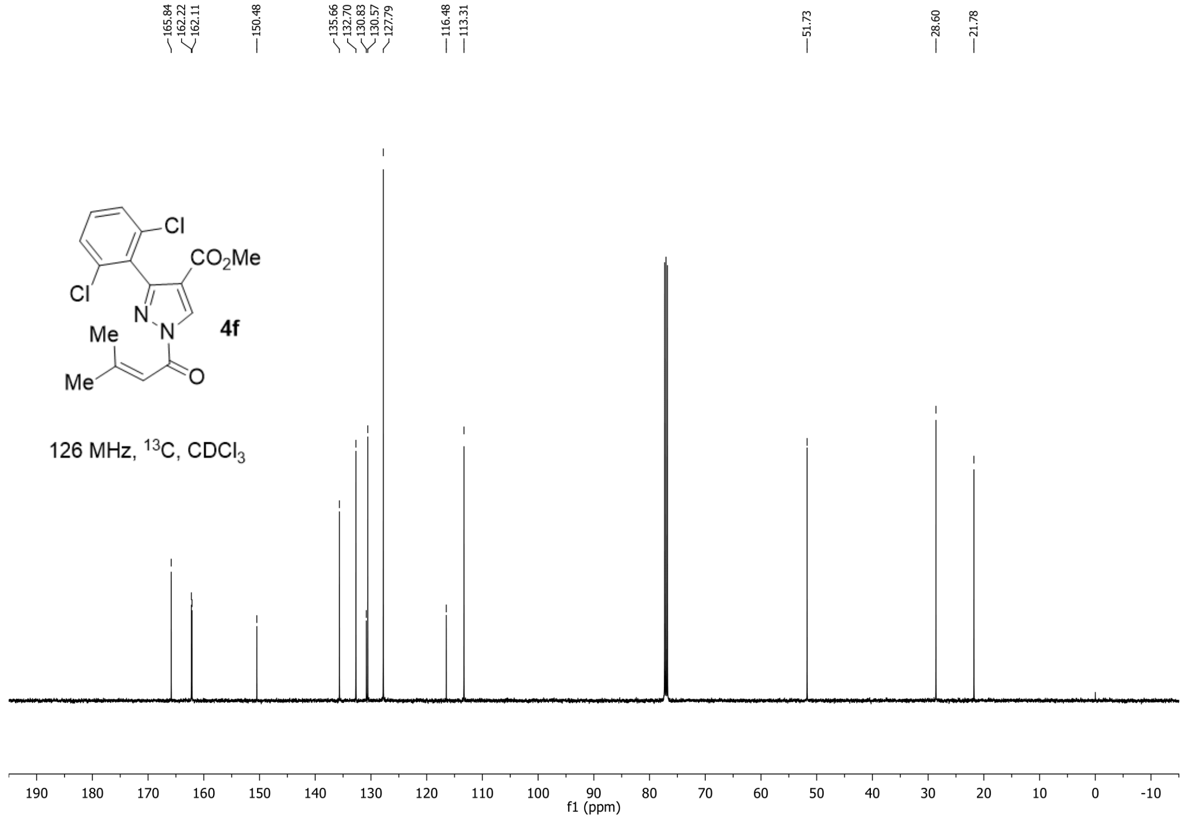

165.84
162.22
162.11
150.48
135.66
132.70
130.83
130.57
127.79
116.48
113.31
51.73
28.60
21.78
Cl
CO_2Me
Cl
N
N
Me
Me
O
4f
126 MHz, ^{13}C, $CDCl_3$
f1 (ppm)

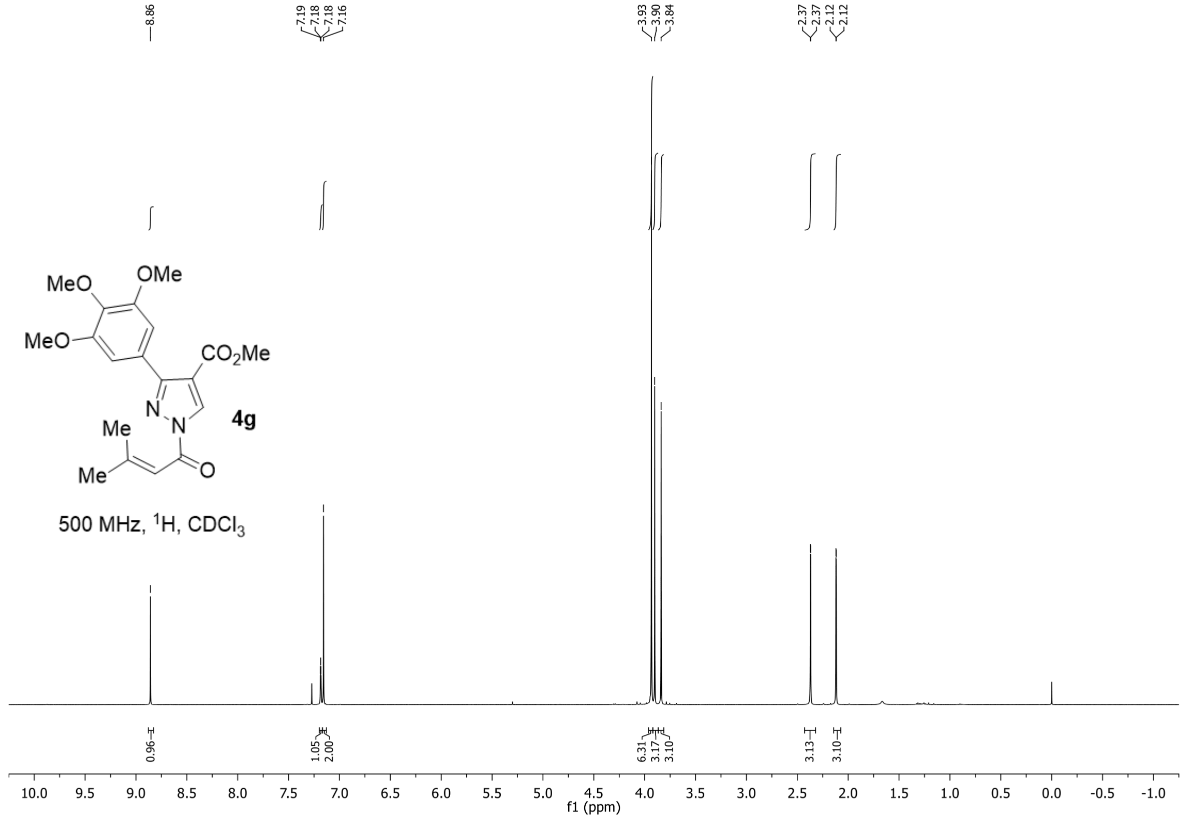
8.86
7.19
7.18
7.18
7.16
3.93
3.90
3.84
2.37
2.37
2.12
2.12
MeO
OMe
MeO
CO_2Me
N
N
Me
4g
Me
O
500 MHz, ^{1}H, $CDCl_3$
0.96
1.05
2.00
6.31
3.17
3.10
3.13
3.10
f1 (ppm)

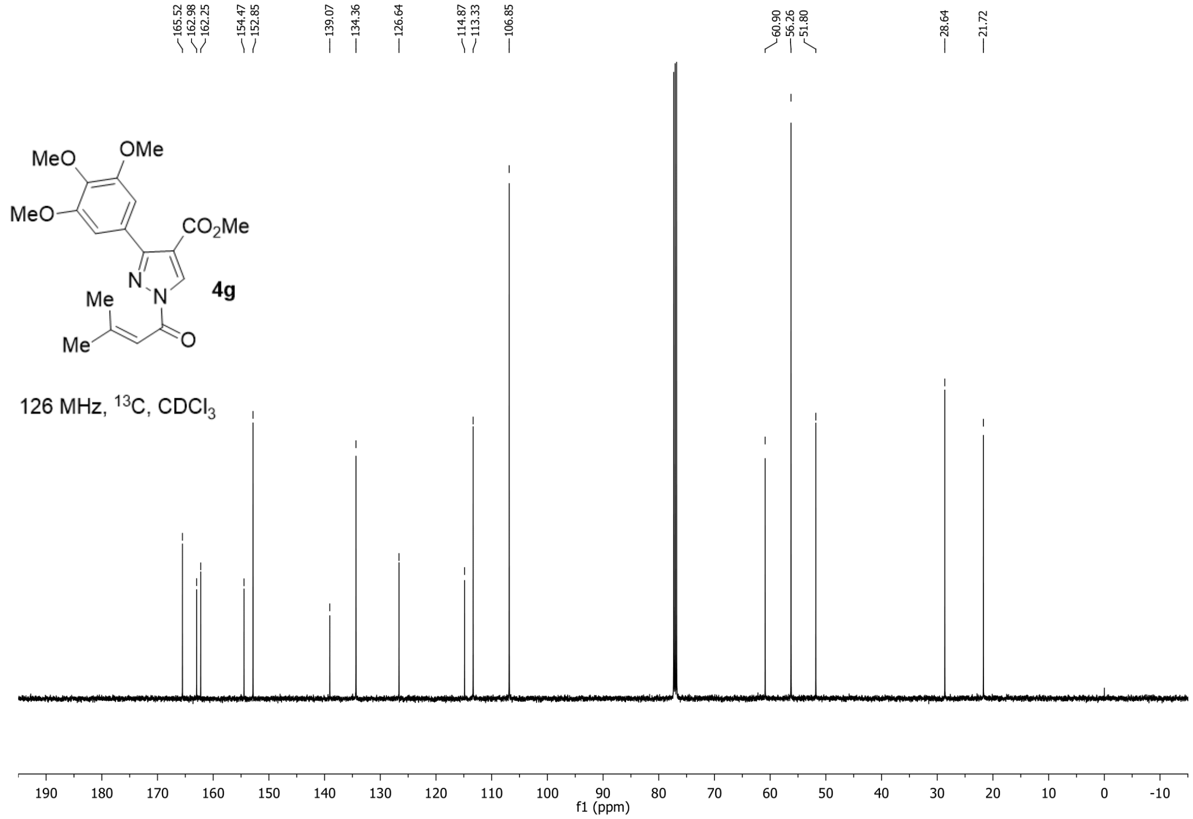
165.52
162.98
162.25
154.47
152.85
139.07
134.36
126.64
114.87
113.33
106.85
60.90
56.26
51.80
28.64
21.72
MeO
OMe
MeO
CO_2Me
N
N
Me
4g
Me
O
126 MHz, ^{13}C, $CDCl_3$
f1 (ppm)

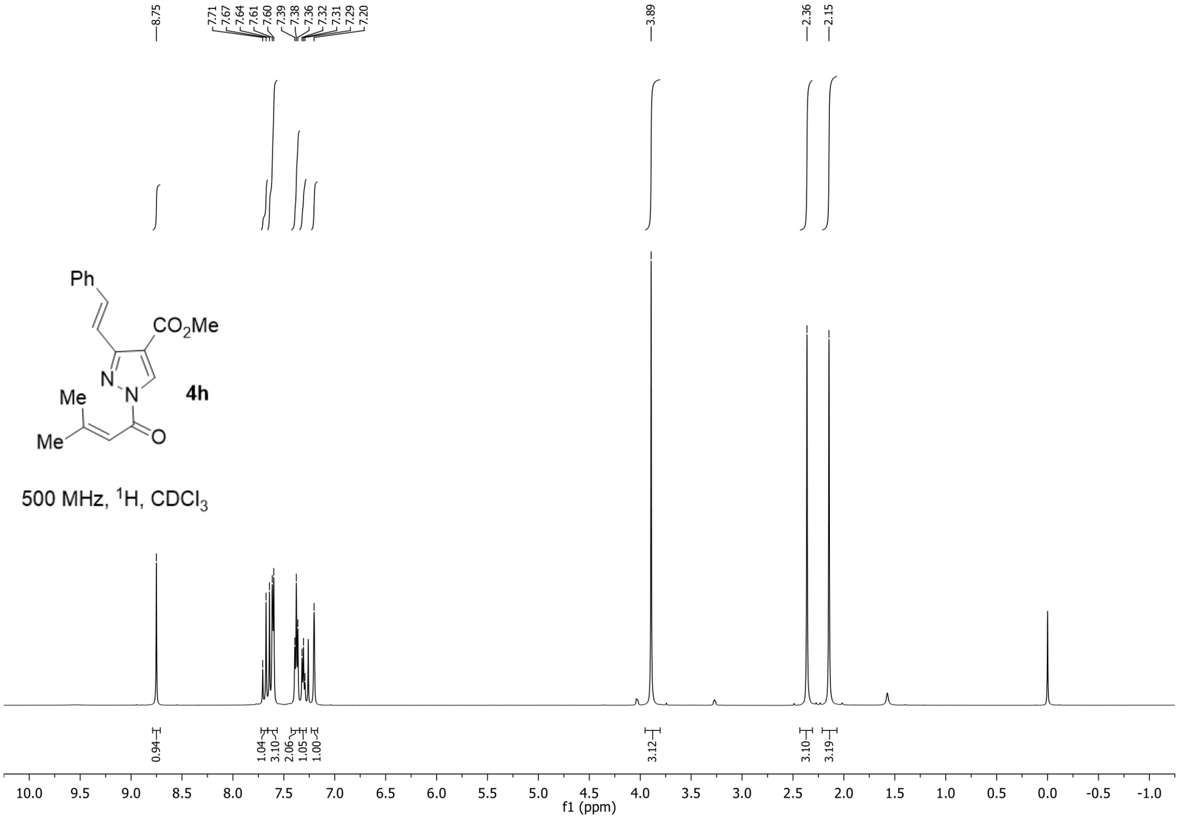
8.75
7.71 7.67 7.64 7.61 7.60 7.39 7.38 7.36 7.32 7.31 7.29 7.20
3.89
2.36
2.15
Ph
CO2Me
N
N
Me
Me
O
4h
500 MHz, 1H, CDCl3
0.94
1.04 3.10 2.06 1.05 1.00
3.12
3.10
3.19
10.0 9.5 9.0 8.5 8.0 7.5 7.0 6.5 6.0 5.5 5.0 4.5 4.0 3.5 3.0 2.5 2.0 1.5 1.0 0.5 0.0 -0.5 -1.0
f1 (ppm)

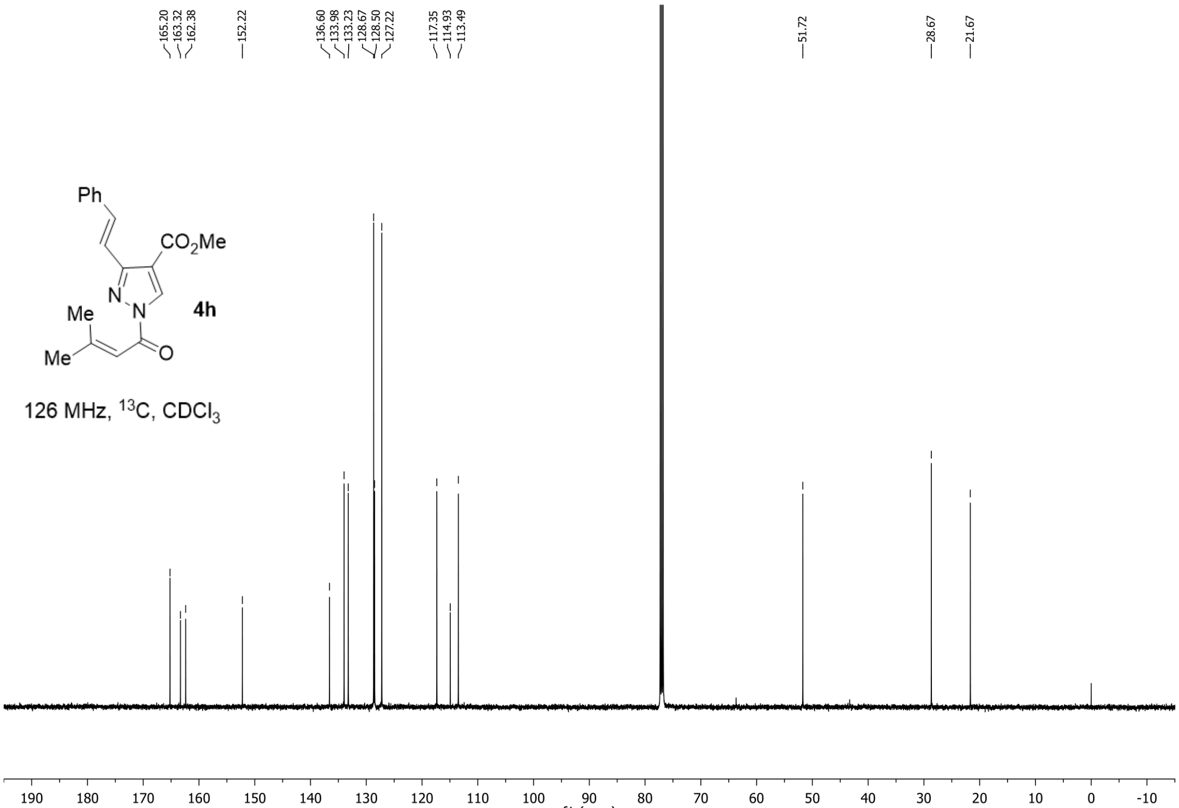
165.20 163.32 162.38
152.22
136.60 133.98 133.23 128.67 128.50 127.22
117.35 114.93 113.49
51.72
28.67
21.67
Ph
CO2Me
N
N
Me
Me
O
4h
126 MHz, 13C, CDCl3
190 180 170 160 150 140 130 120 110 100 90 80 70 60 50 40 30 20 10 0 -10
f1 (ppm)

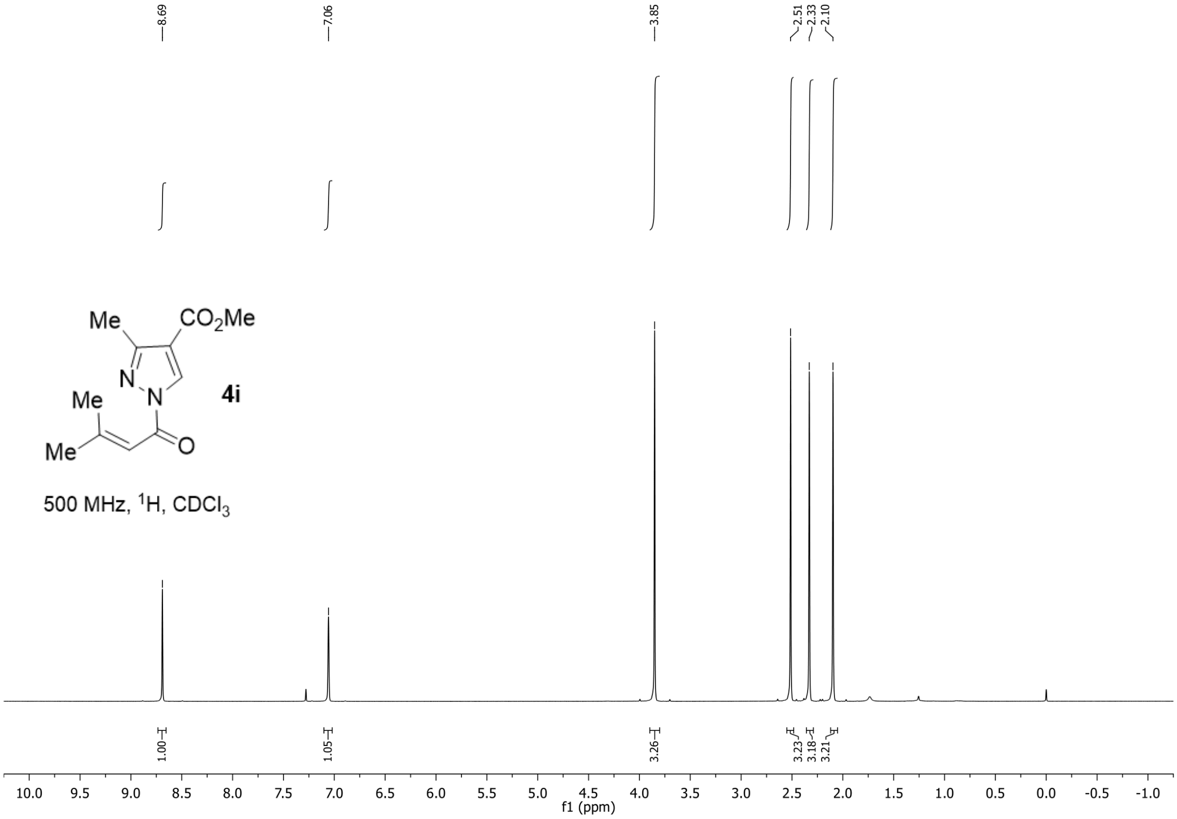
8.69
7.06
3.85
2.51
2.33
2.10
Me
CO2Me
N
N
Me
Me
O
4i
500 MHz, 1H, CDCl3
1.00
1.05
3.26
3.23
3.18
3.21
10.0 9.5 9.0 8.5 8.0 7.5 7.0 6.5 6.0 5.5 5.0 4.5 4.0 3.5 3.0 2.5 2.0 1.5 1.0 0.5 0.0 -0.5 -1.0
f1 (ppm)

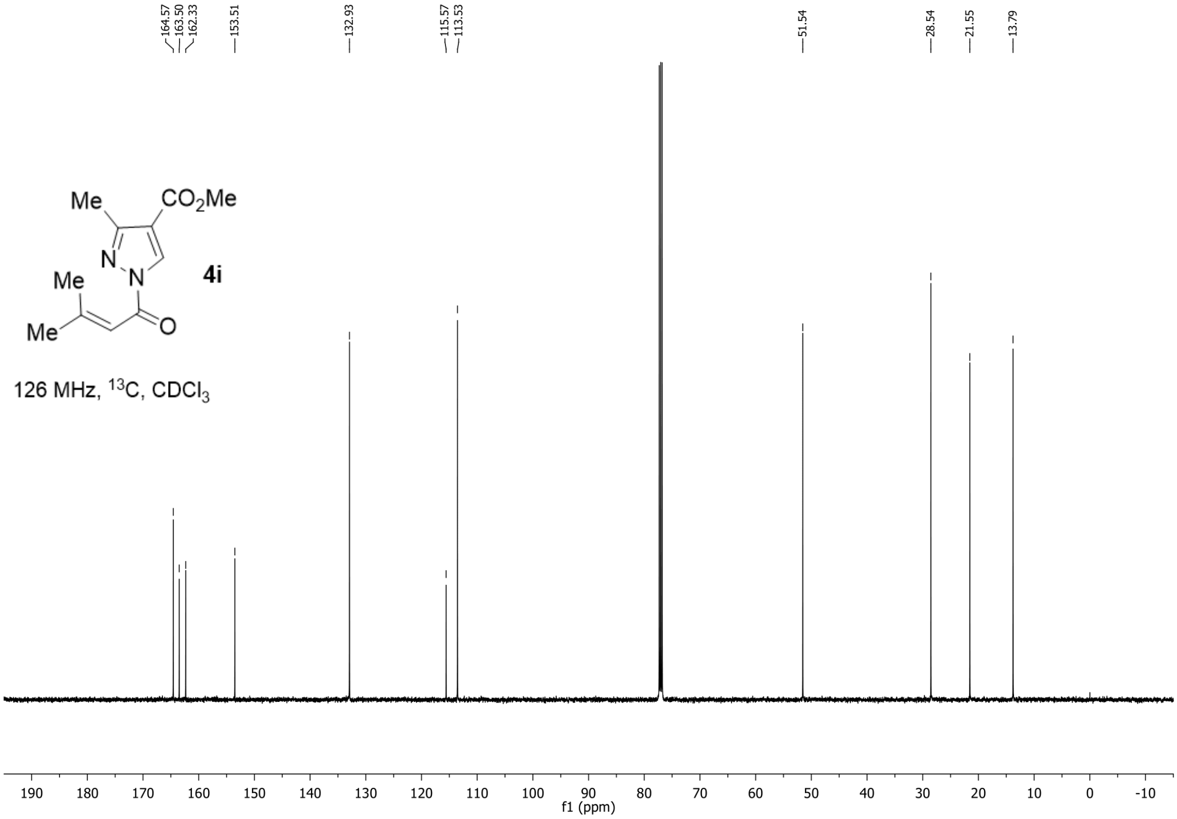
164.57
163.50
162.33
153.51
132.93
115.57
113.53
51.54
28.54
21.55
13.79
Me
CO2Me
N
N
Me
Me
O
4i
126 MHz, 13C, CDCl3
190 180 170 160 150 140 130 120 110 100 90 80 70 60 50 40 30 20 10 0 -10
f1 (ppm)

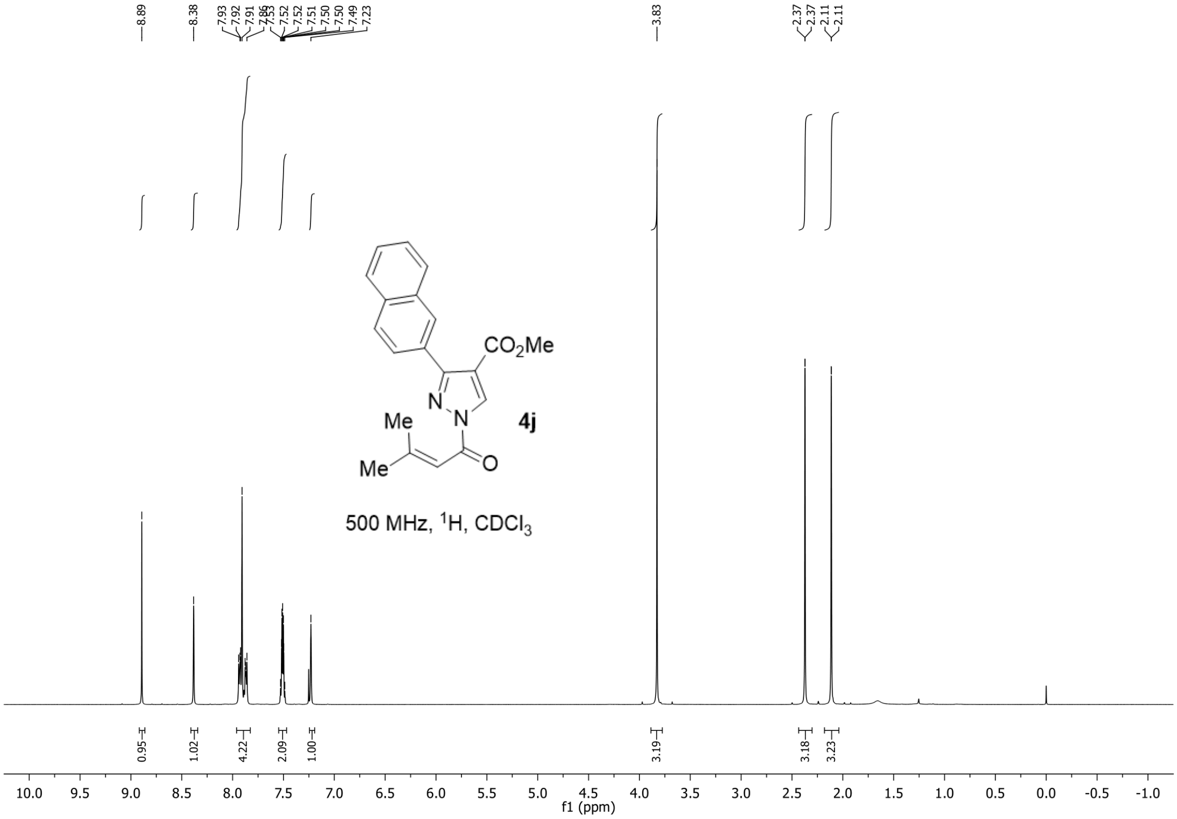

8.89
8.38
7.93
7.92
7.91
7.52
7.52
7.51
7.50
7.50
7.49
7.23
3.83
2.37
2.37
2.11
2.11
CO2Me
N
N
Me
Me
O
4j
500 MHz, 1H, CDCl3
0.95
1.02
4.22
2.09
1.00
3.19
3.18
3.23
f1 (ppm)

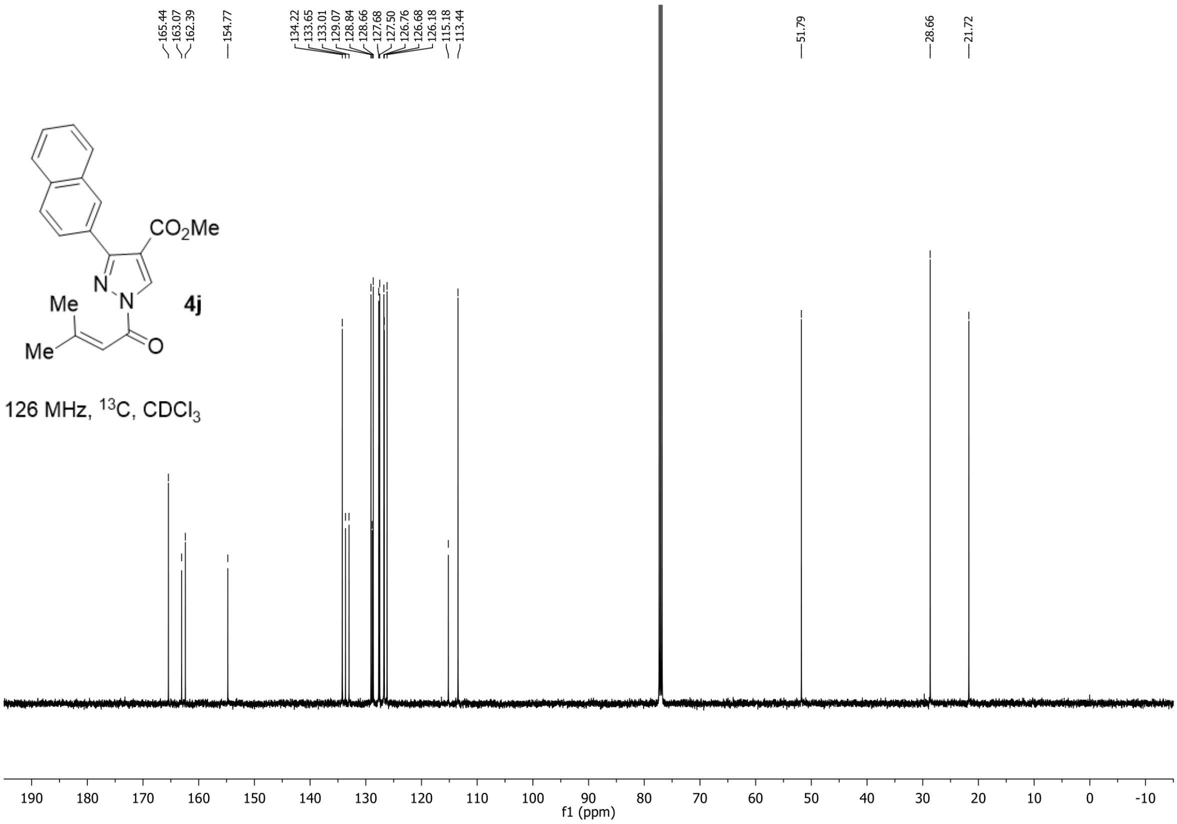

165.44
163.07
162.39
154.77
134.22
133.65
133.01
129.07
128.84
128.66
127.68
127.50
126.76
126.68
126.18
115.18
113.44
51.79
28.66
21.72
CO2Me
N
N
Me
Me
O
4j
126 MHz, 13C, CDCl3
f1 (ppm)

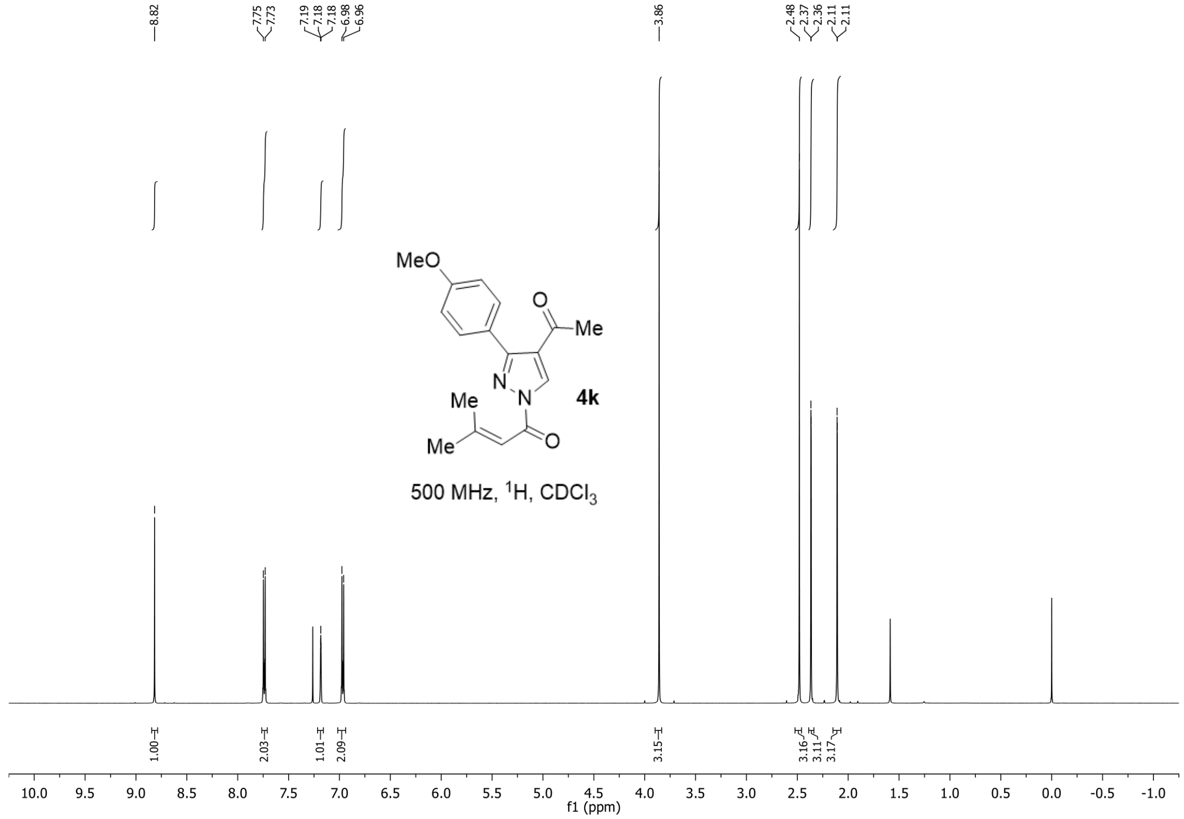
8.82
7.75
7.73
7.19
7.18
7.18
6.98
6.96
3.86
2.48
2.37
2.36
2.11
2.11
MeO
O
Me
N
N
Me
Me
O
4k
500 MHz, ¹H, CDCl₃
1.00
2.03
1.01
2.09
3.15
3.16
3.11
3.17
f1 (ppm)

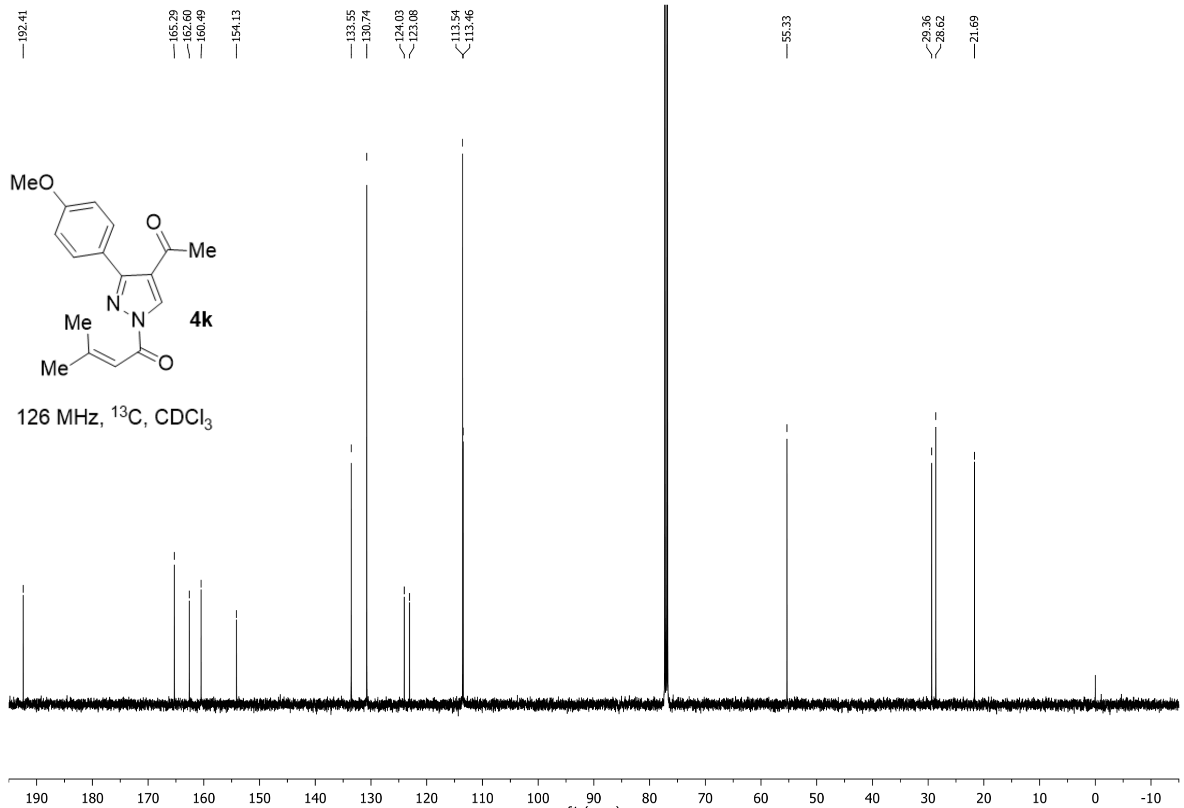
192.41
165.29
162.60
160.49
154.13
133.55
130.74
124.03
123.08
113.54
113.46
55.33
29.36
28.62
21.69
MeO
O
Me
N
N
Me
Me
O
4k
126 MHz, ¹³C, CDCl₃
f1 (ppm)

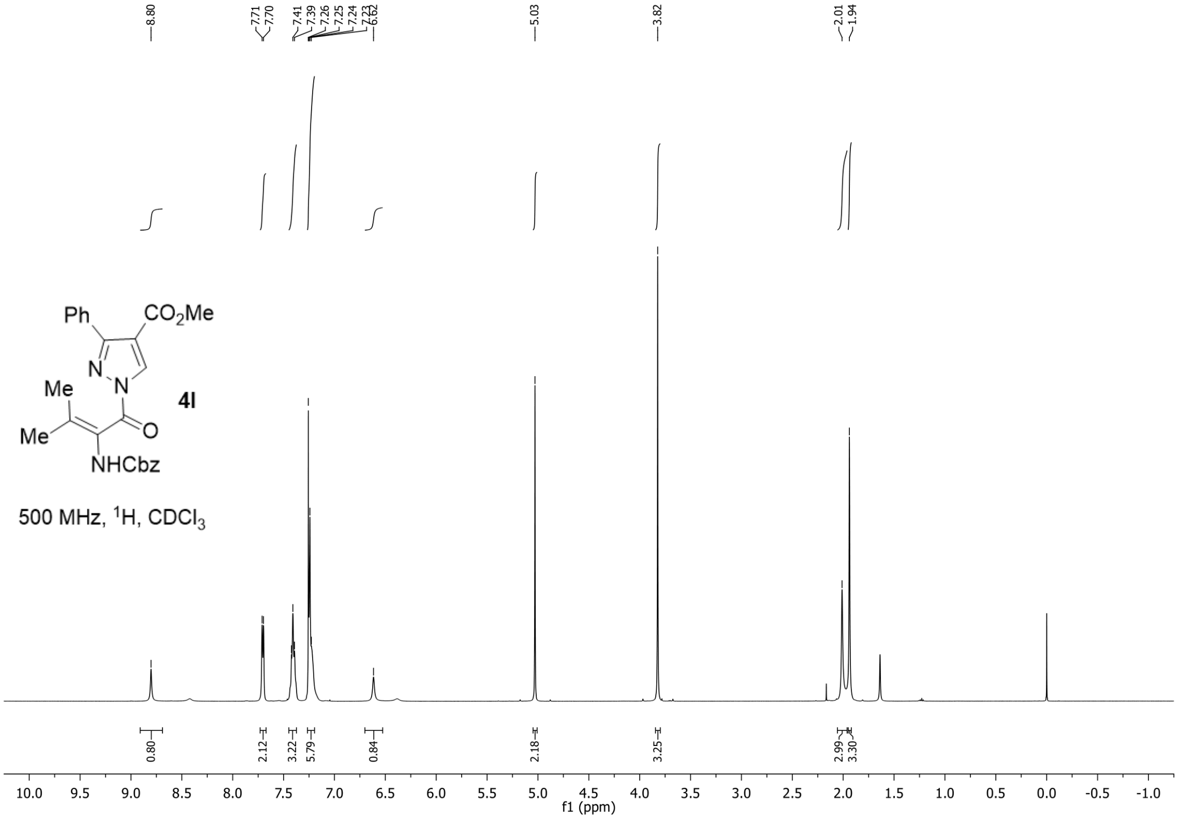
8.80
7.71
7.70
7.41
7.39
7.26
7.25
7.24
7.23
6.62
5.03
3.82
2.01
1.94
Ph
CO2Me
N
N
Me
Me
O
NHCbz
4l
500 MHz, 1H, CDCl3
0.80
2.12
3.22
5.79
0.84
2.18
3.25
2.99
3.30
f1 (ppm)

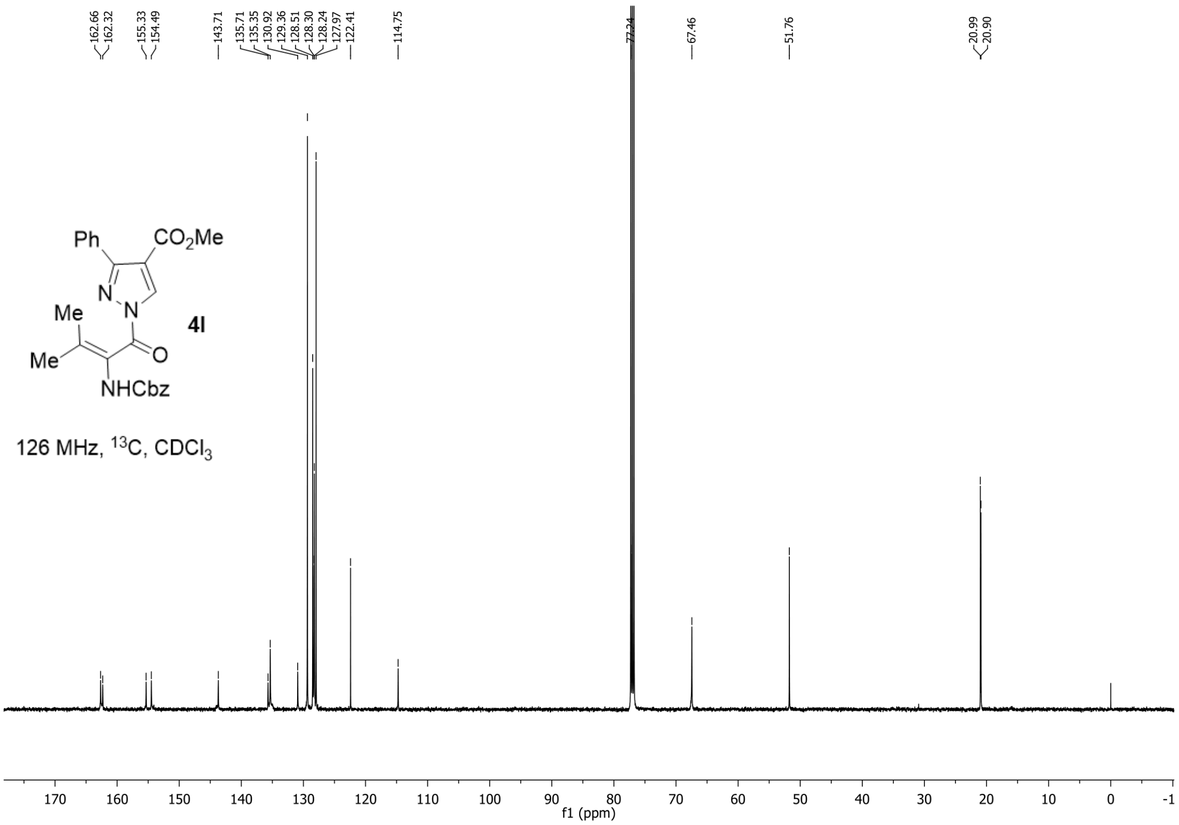
162.66
162.32
155.33
154.49
143.71
135.71
135.35
130.92
129.36
128.51
128.30
128.24
127.97
122.41
114.75
77.24
67.46
51.76
20.99
20.90
Ph
CO2Me
N
N
Me
Me
O
NHCbz
4l
126 MHz, 13C, CDCl3
f1 (ppm)

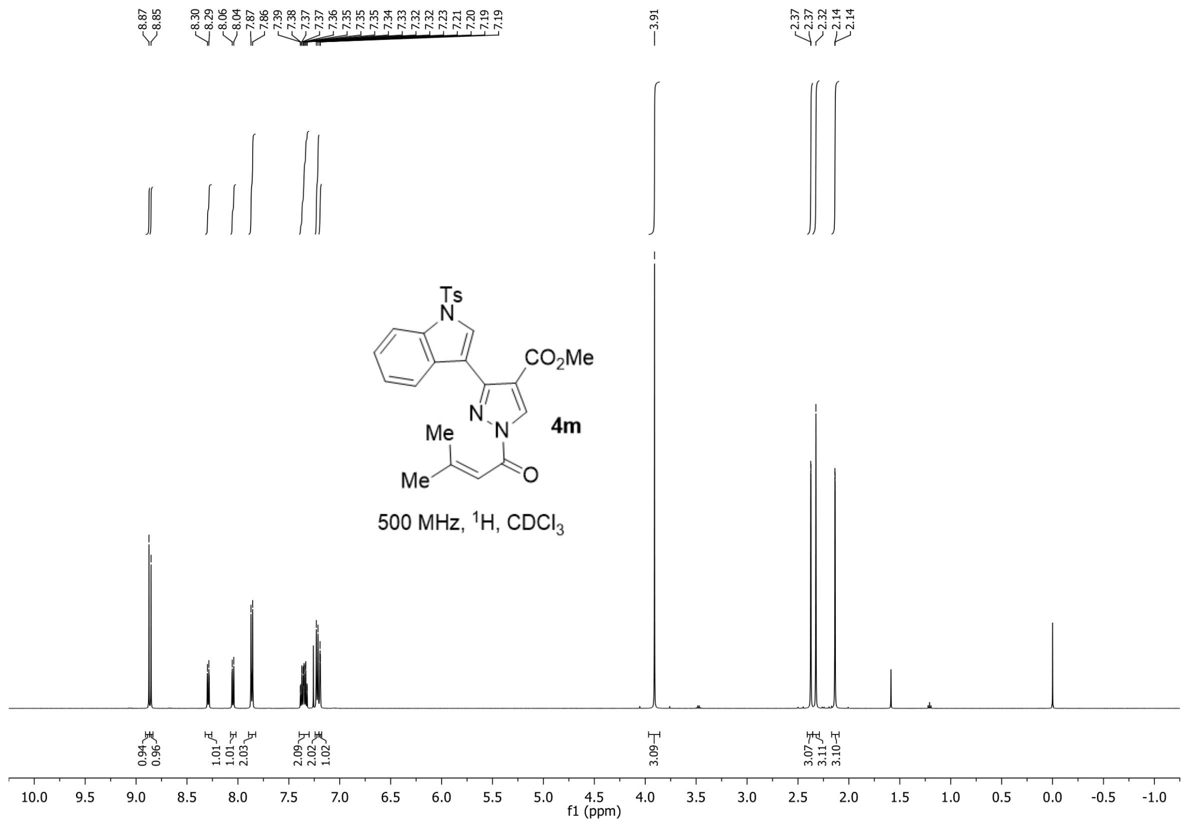
Ts
N
CO2Me
N
N
4m
Me
Me
O
500 MHz, 1H, CDCl3
f1 (ppm)

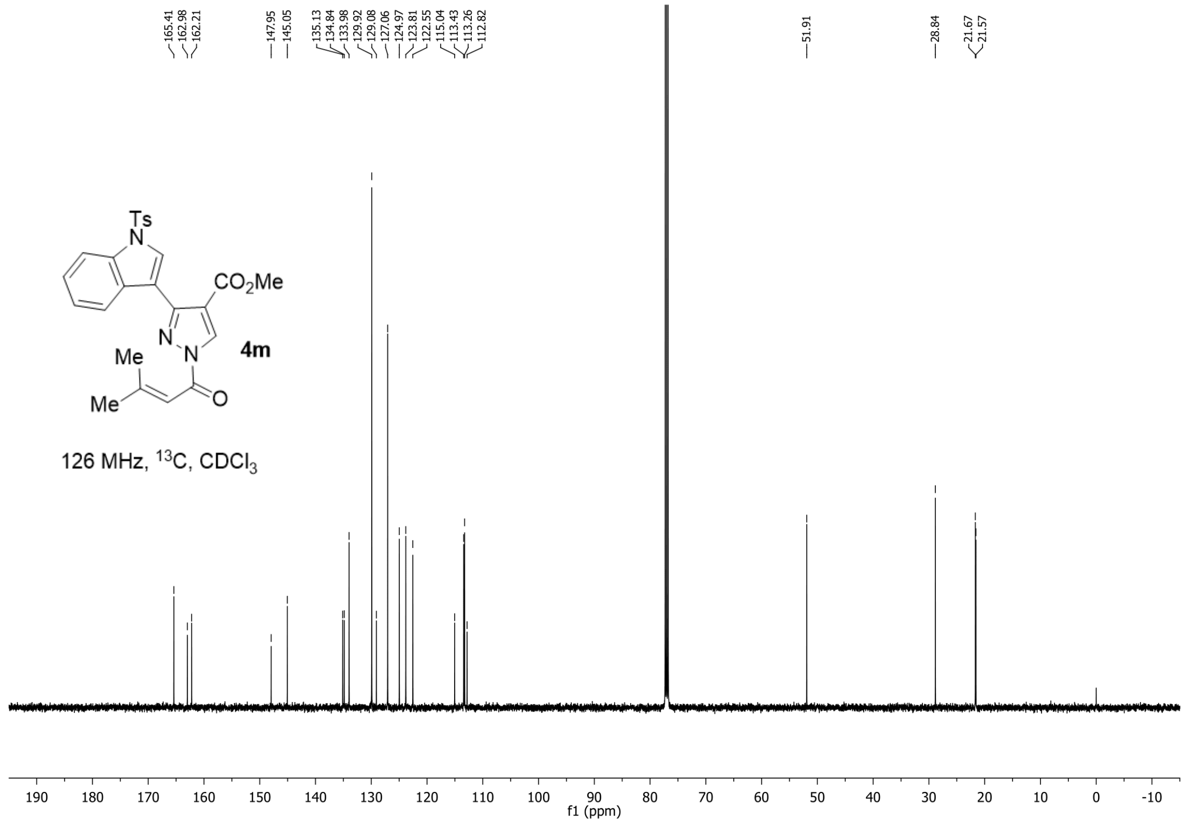
Ts
N
CO2Me
N
N
4m
Me
Me
O
126 MHz, 13C, CDCl3
f1 (ppm)

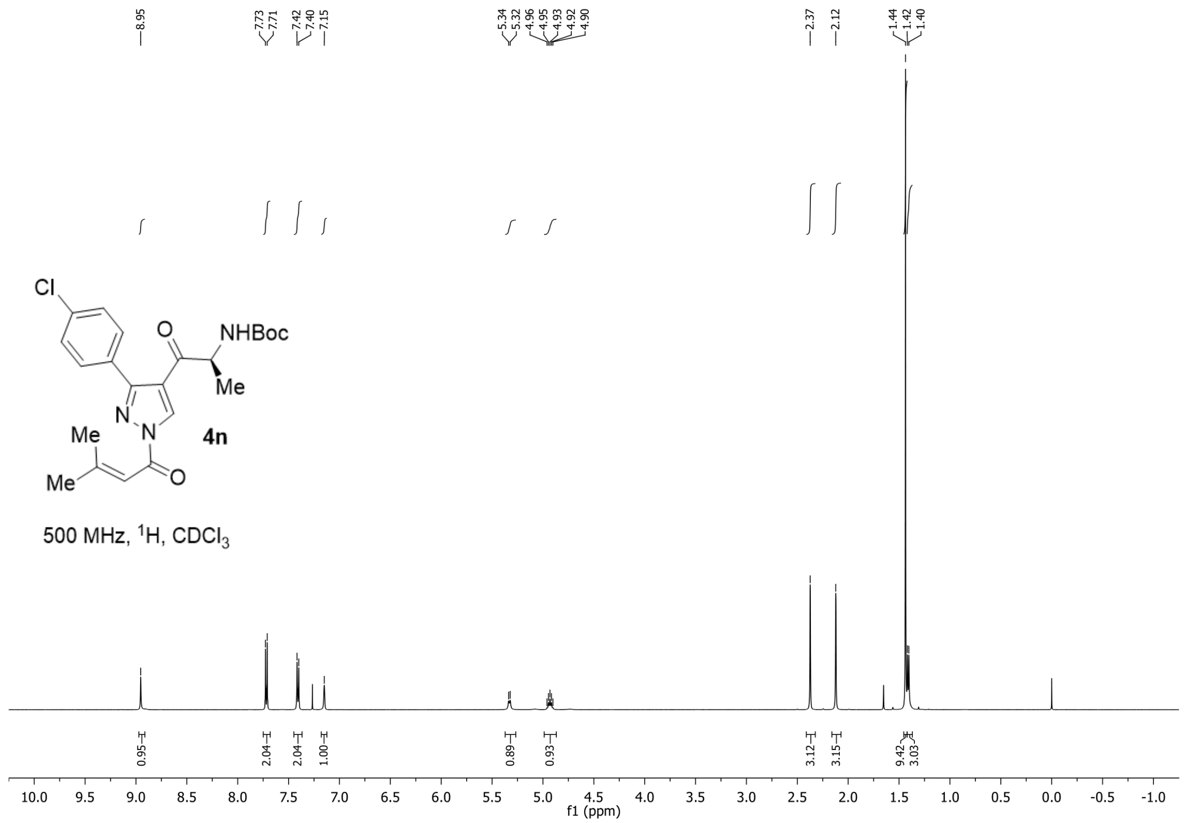
8.95
7.73
7.71
7.42
7.40
7.15
5.34
5.32
4.96
4.95
4.93
4.92
4.90
2.37
2.12
1.44
1.42
1.40
Cl
O
NHBoc
Me
N
N
Me
4n
Me
O
500 MHz, ^{1}H, $CDCl_3$
0.95
2.04
2.04
1.00
0.89
0.93
3.12
3.15
9.42
3.03
10.0 9.5 9.0 8.5 8.0 7.5 7.0 6.5 6.0 5.5 5.0 4.5 4.0 3.5 3.0 2.5 2.0 1.5 1.0 0.5 0.0 -0.5 -1.0
f1 (ppm)

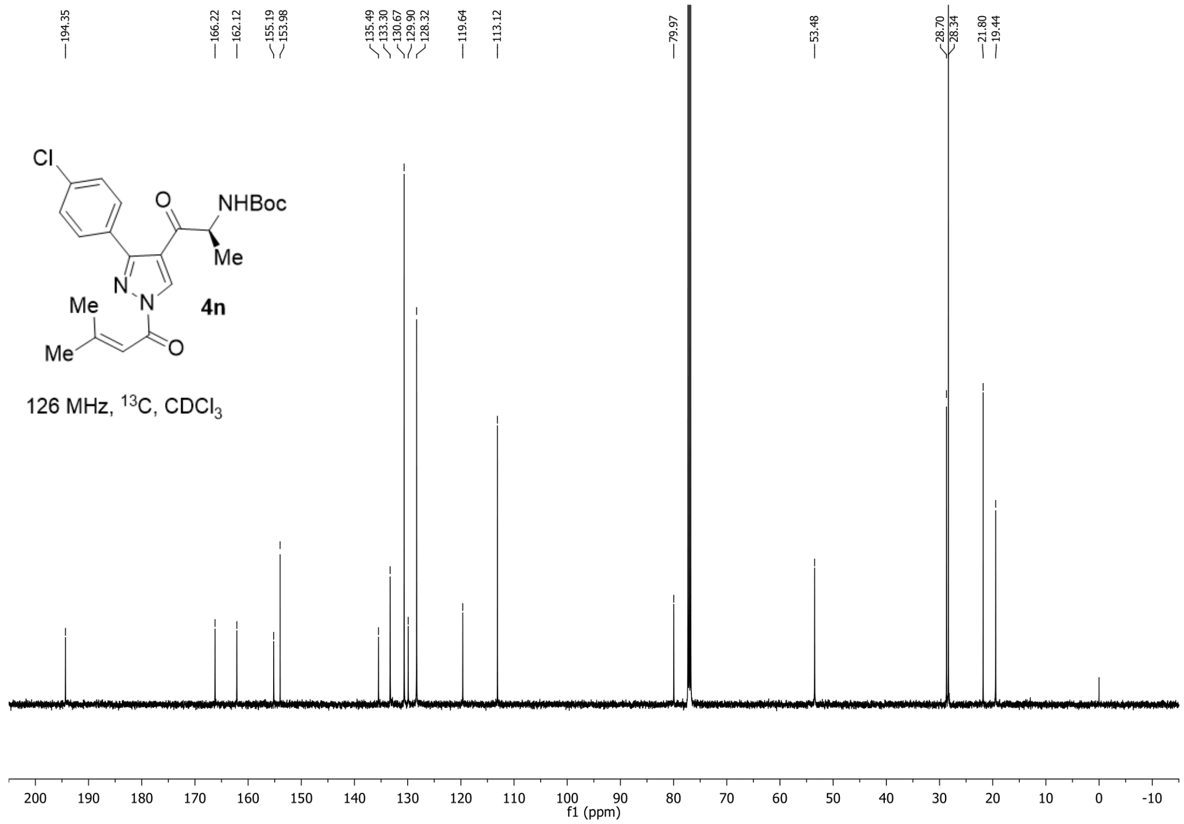
194.35
166.22
162.12
155.19
153.98
135.49
133.30
130.67
129.90
128.32
119.64
113.12
79.97
53.48
28.70
28.34
21.80
19.44
Cl
O
NHBoc
Me
N
N
Me
4n
Me
O
126 MHz, ^{13}C, $CDCl_3$
200 190 180 170 160 150 140 130 120 110 100 90 80 70 60 50 40 30 20 10 0 -10
f1 (ppm)

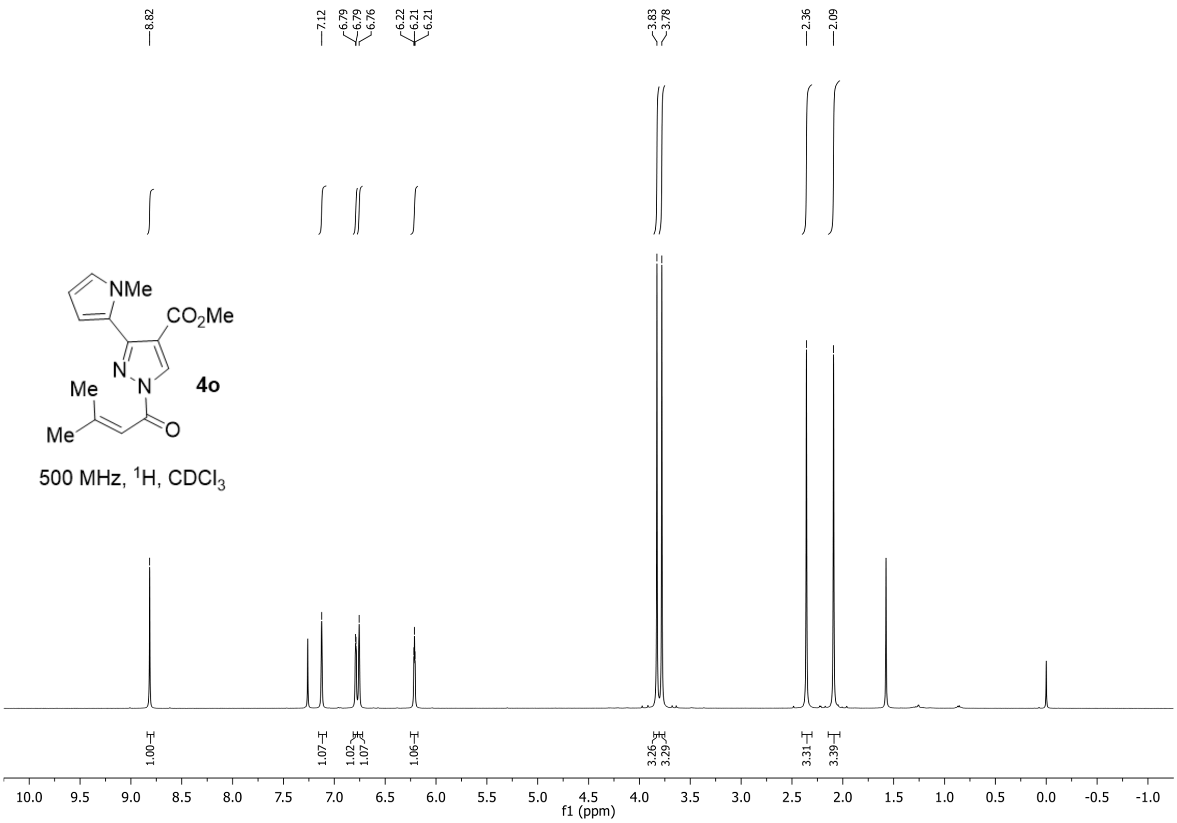
8.82
7.12
6.79
6.79
6.76
6.22
6.21
6.21
3.83
3.78
2.36
2.09
NMe
CO2Me
N
N
Me
Me
O
4o
500 MHz, 1H, CDCl3
1.00
1.07
1.02
1.07
1.06
3.26
3.29
3.31
3.39
f1 (ppm)

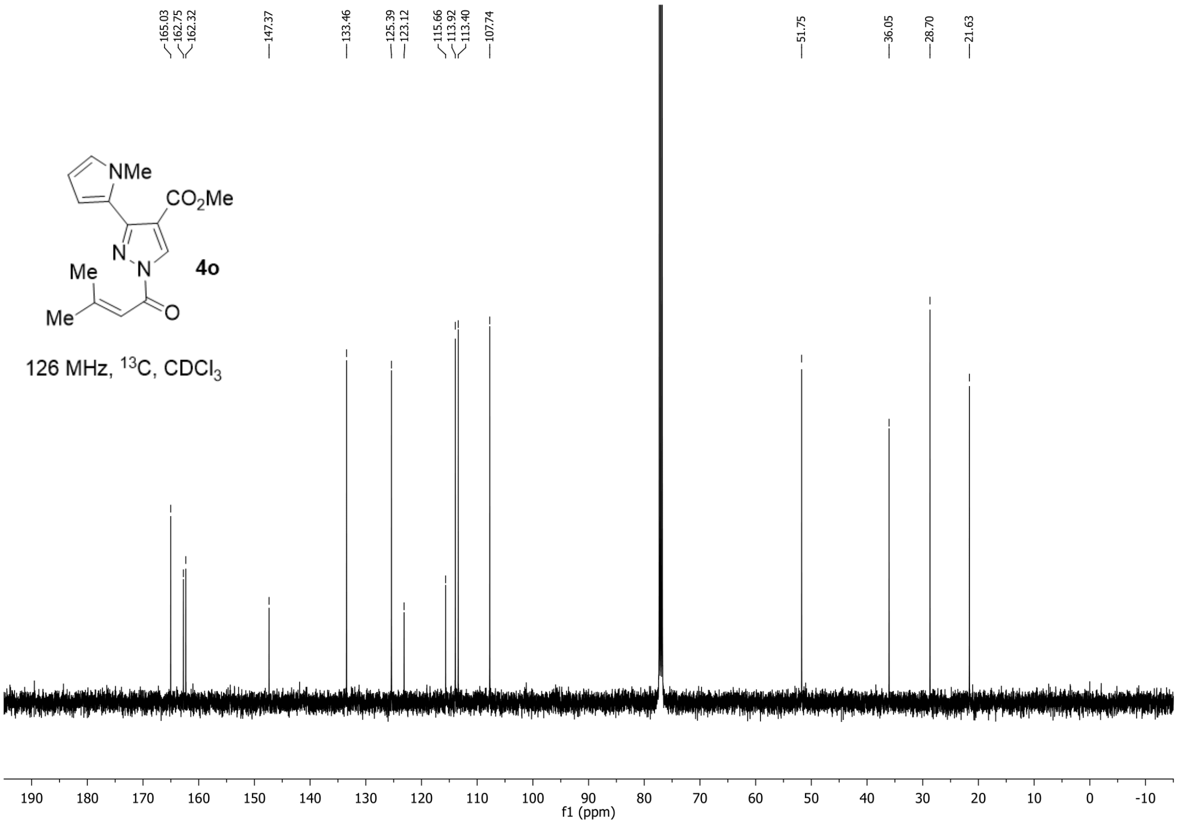
165.03
162.75
162.32
147.37
133.46
125.39
123.12
115.66
113.92
113.40
107.74
51.75
36.05
28.70
21.63
NMe
CO2Me
N
N
Me
Me
O
4o
126 MHz, 13C, CDCl3
f1 (ppm)

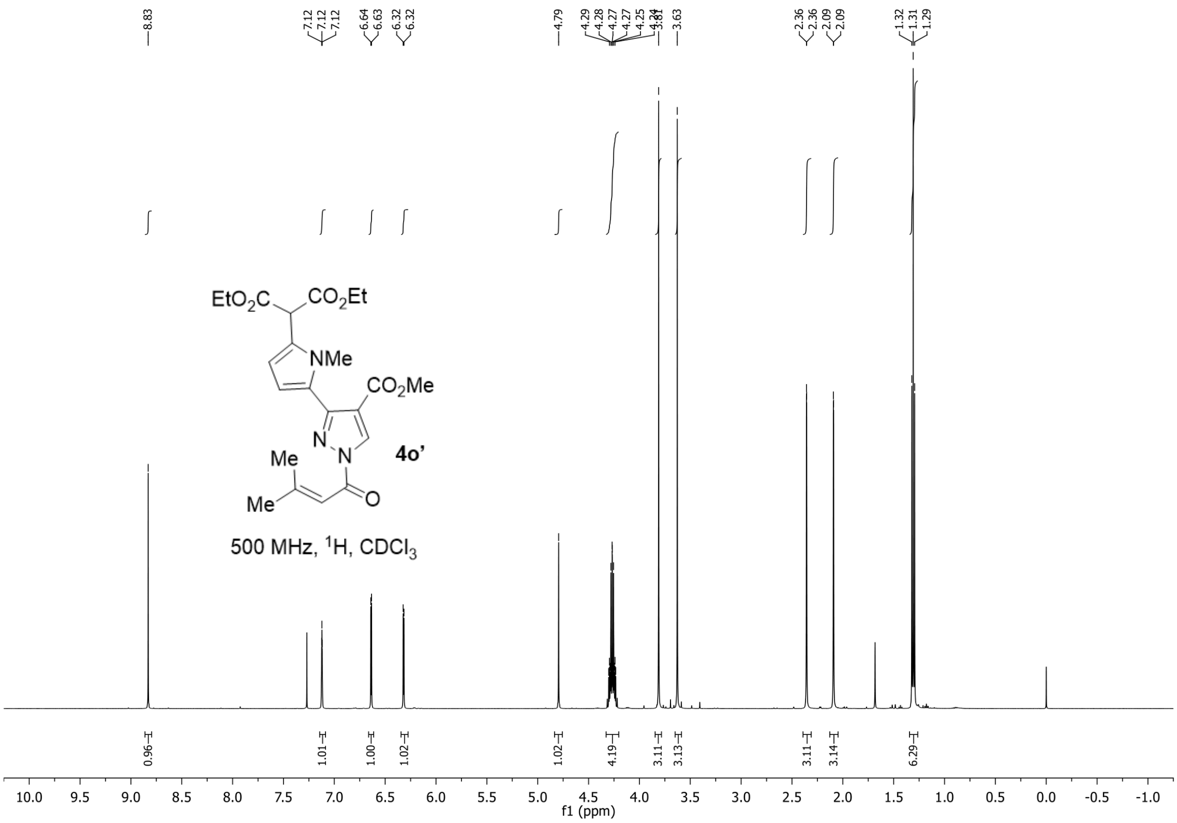
8.83
7.12
7.12
7.12
6.64
6.63
6.32
6.32
4.79
4.29
4.28
4.27
4.27
4.25
3.81
3.63
2.36
2.36
2.09
2.09
1.32
1.31
1.29
EtO2C
CO2Et
NMe
CO2Me
N
N
Me
Me
O
4o'
500 MHz, 1H, CDCl3
0.96
1.01
1.00
1.02
1.02
4.19
3.11
3.13
3.11
3.14
6.29
f1 (ppm)

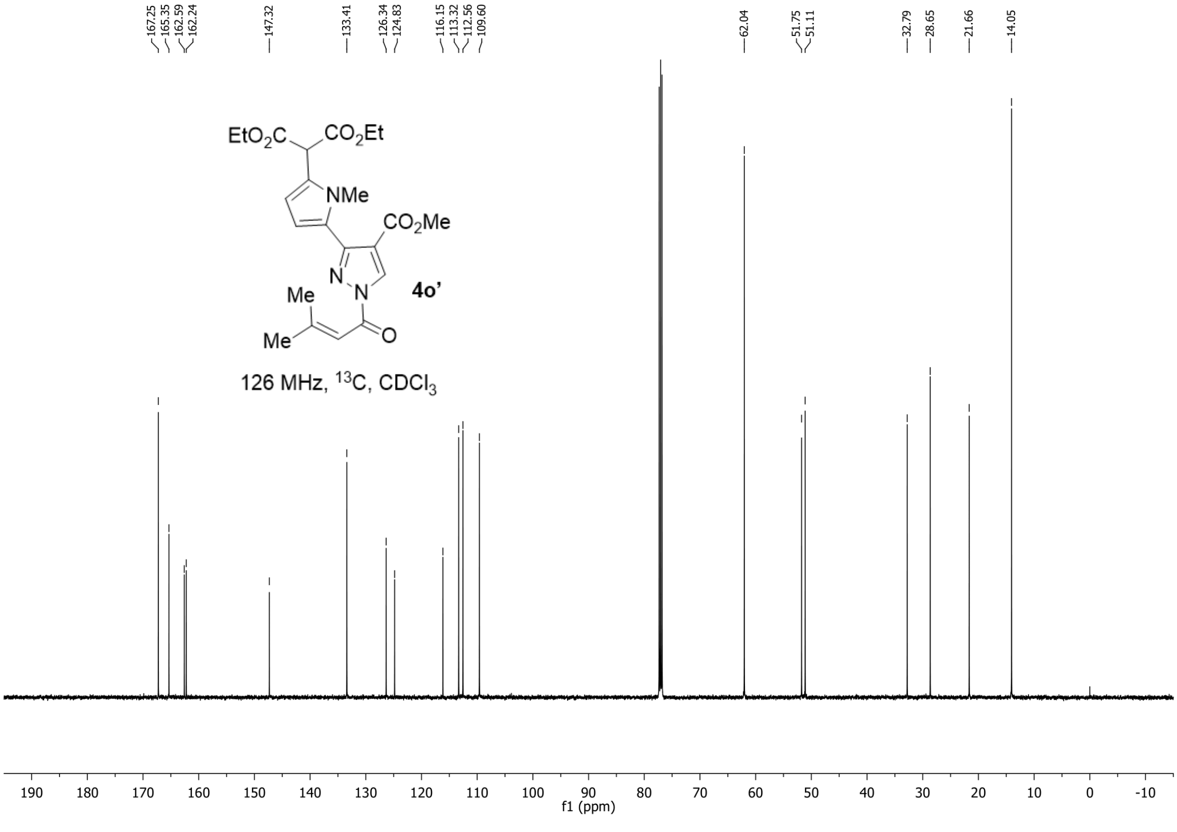
167.25
165.35
162.59
162.24
147.32
133.41
126.34
124.83
116.15
113.32
112.56
109.60
62.04
51.75
51.11
32.79
28.65
21.66
14.05
EtO2C
CO2Et
NMe
CO2Me
N
N
Me
Me
O
4o'
126 MHz, 13C, CDCl3
f1 (ppm)

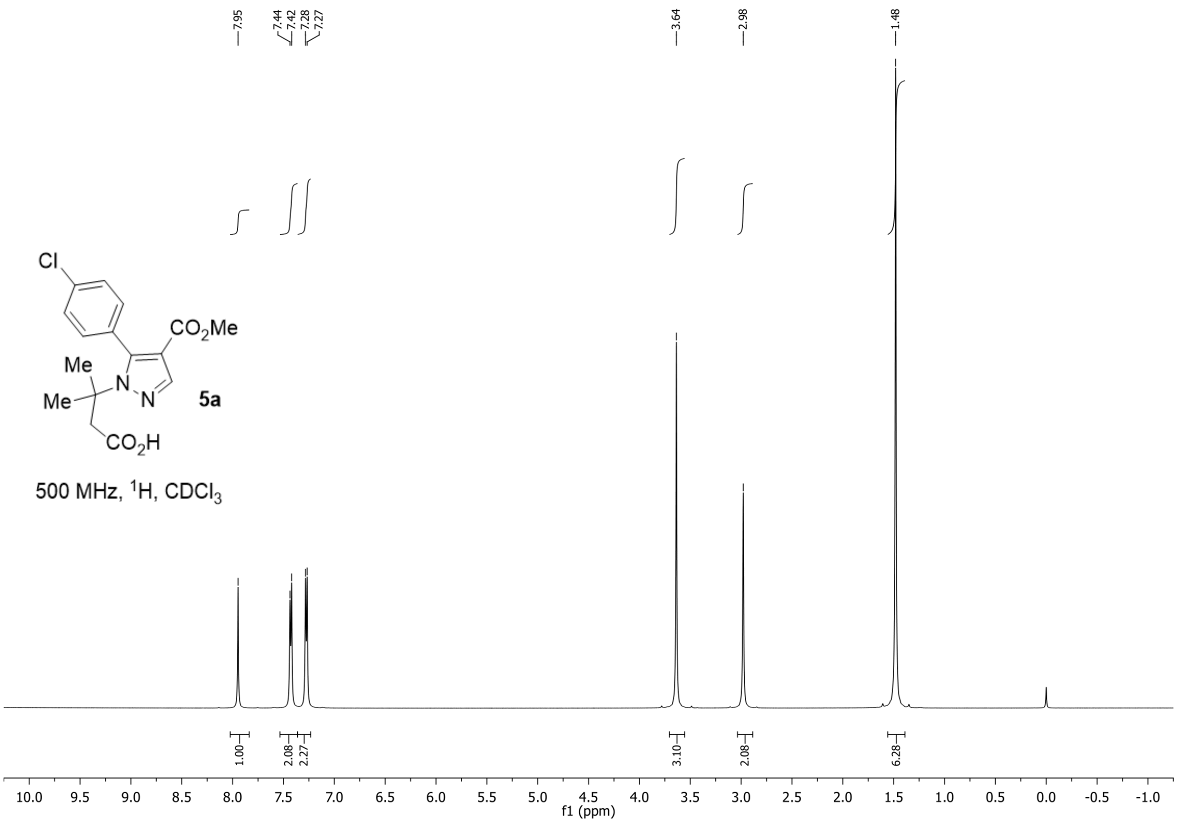
7.95
7.44
7.42
7.28
7.27
3.64
2.98
1.48
Cl
CO2Me
Me
Me
N
N
CO2H
5a
500 MHz, 1H, CDCl3
1.00
2.08
2.27
3.10
2.08
6.28
f1 (ppm)

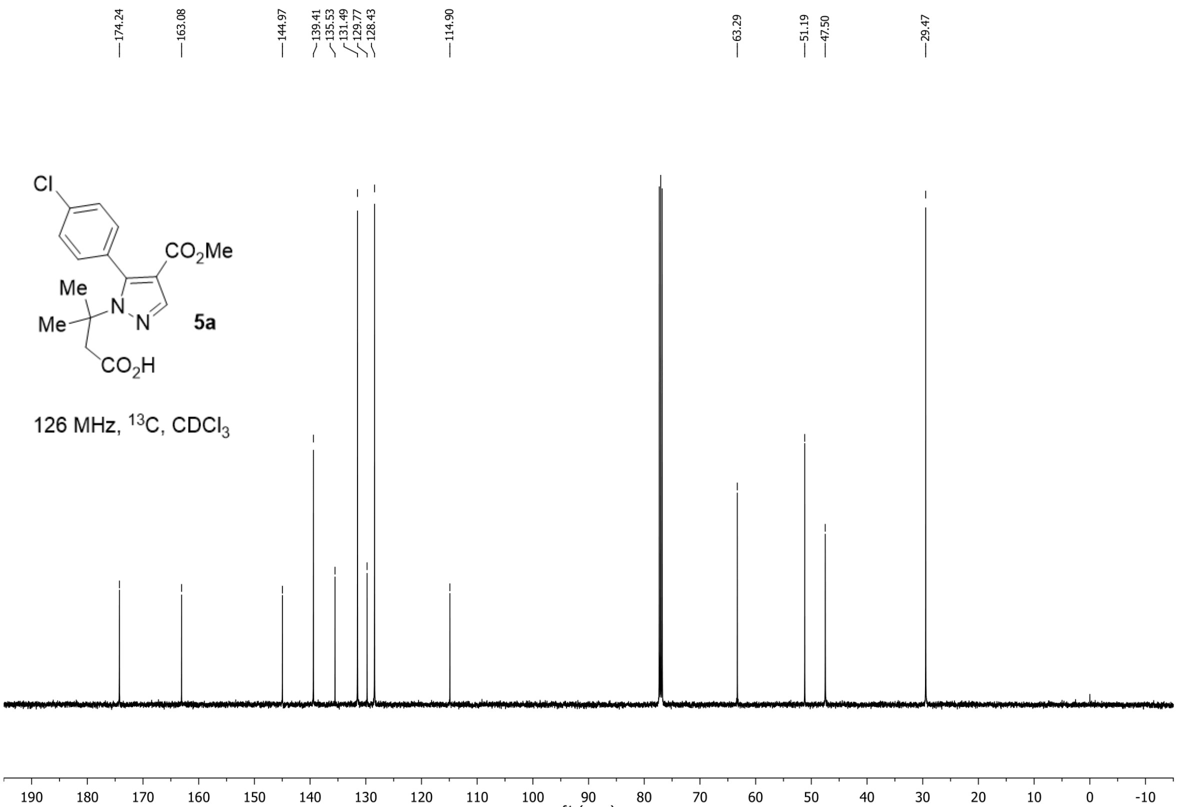
174.24
163.08
144.97
139.41
135.53
131.49
129.77
128.43
114.90
63.29
51.19
47.50
29.47
Cl
CO2Me
Me
Me
N
N
CO2H
5a
126 MHz, 13C, CDCl3
f1 (ppm)

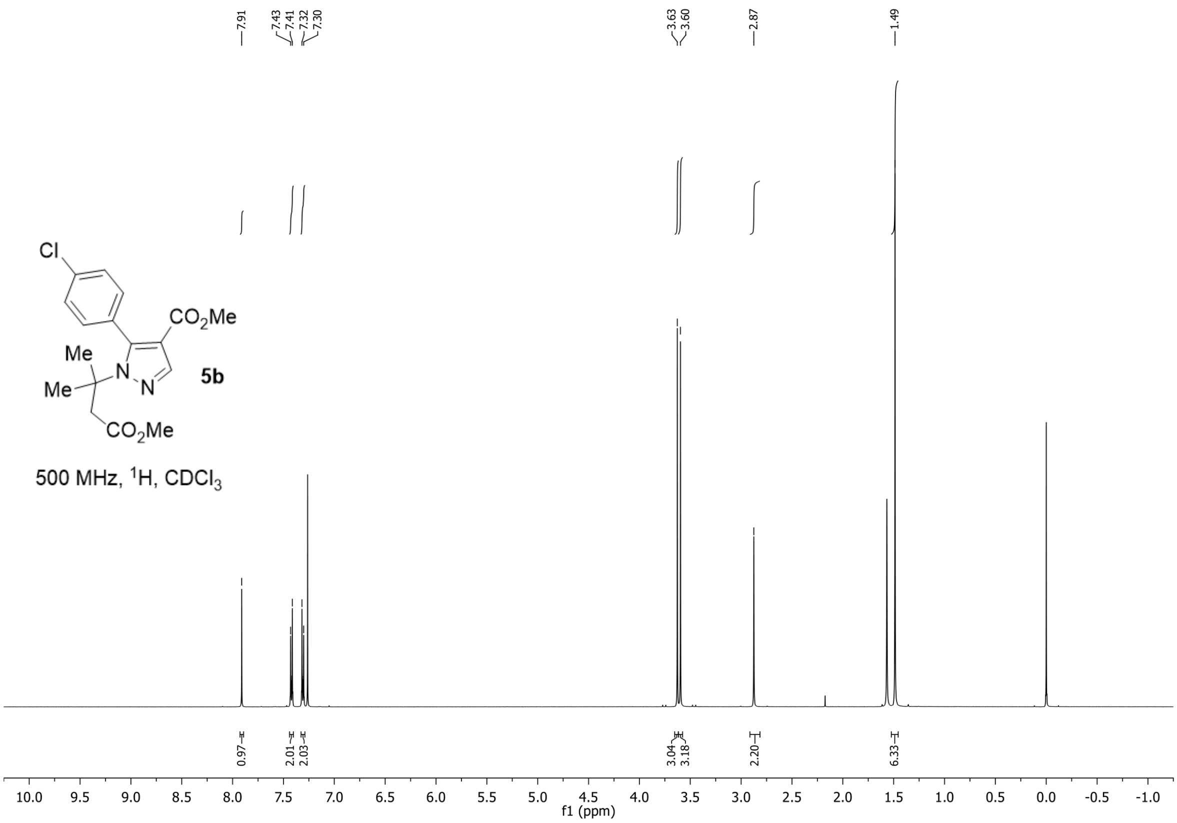

7.91
7.43
7.41
7.32
7.30
3.63
3.60
2.87
1.49
Cl
CO_2Me
Me
Me
N
N
5b
CO_2Me
500 MHz, ^1H, $CDCl_3$
0.97
2.01
2.03
3.04
3.18
2.20
6.33
10.0 9.5 9.0 8.5 8.0 7.5 7.0 6.5 6.0 5.5 5.0 4.5 4.0 3.5 3.0 2.5 2.0 1.5 1.0 0.5 0.0 -0.5 -1.0
f1 (ppm)

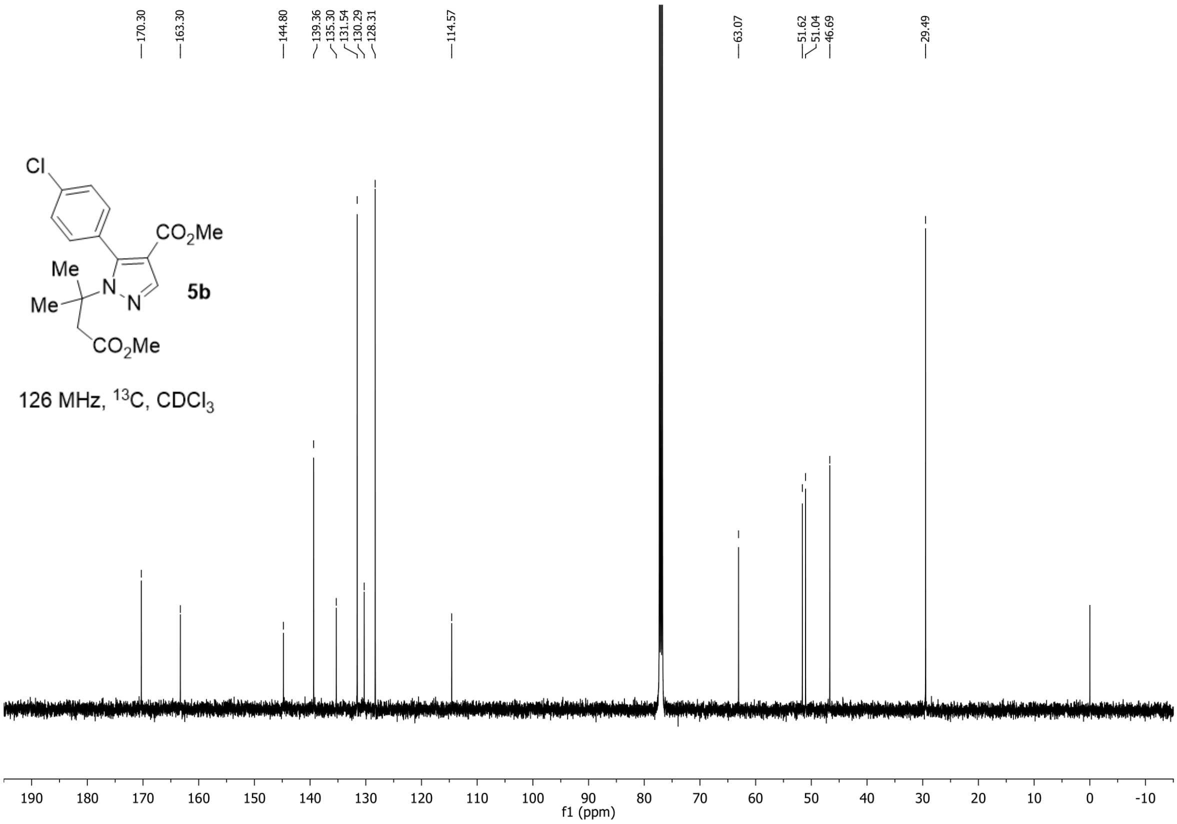

170.30
163.30
144.80
139.36
135.30
131.54
130.29
128.31
114.57
63.07
51.62
51.04
46.69
29.49
Cl
CO_2Me
Me
Me
N
N
5b
CO_2Me
126 MHz, ^{13}C, $CDCl_3$
190 180 170 160 150 140 130 120 110 100 90 80 70 60 50 40 30 20 10 0 -10
f1 (ppm)

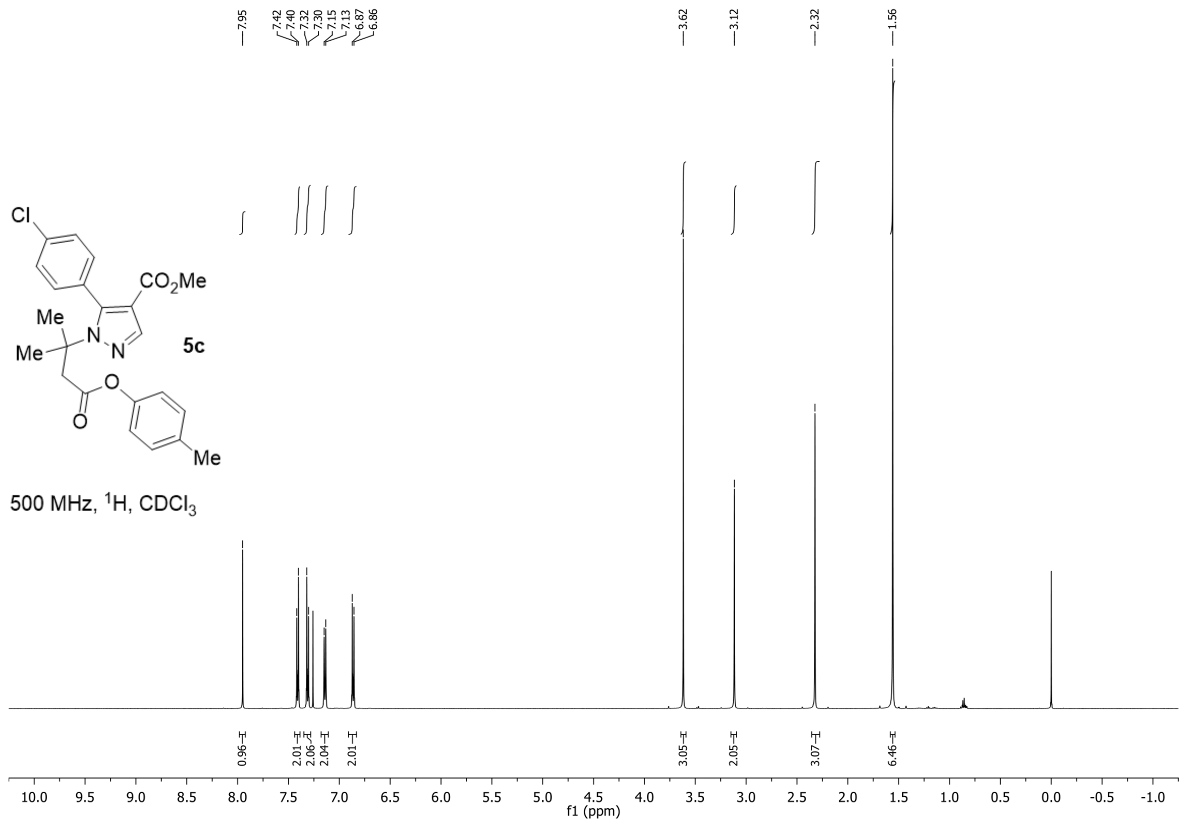
7.95
7.42
7.40
7.32
7.30
7.15
7.13
6.87
6.86
3.62
3.12
2.32
1.56
Cl
CO2Me
Me
Me
N
N
O
O
Me
5c
500 MHz, 1H, CDCl3
0.96
2.01
2.06
2.04
2.01
3.05
2.05
3.07
6.46
f1 (ppm)

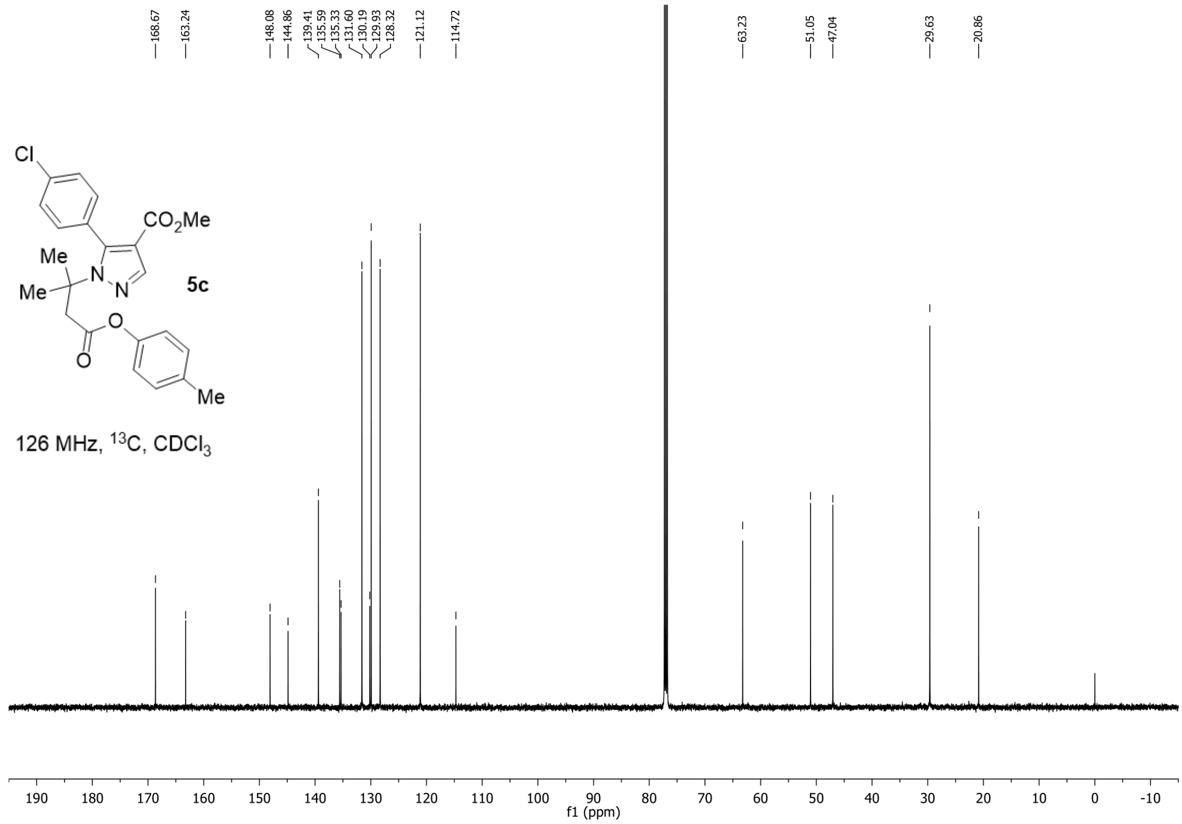
168.67
163.24
148.08
144.86
139.41
135.59
135.33
131.60
130.19
129.93
128.32
121.12
114.72
63.23
51.05
47.04
29.63
20.86
Cl
CO2Me
Me
Me
N
N
O
O
Me
5c
126 MHz, 13C, CDCl3
f1 (ppm)

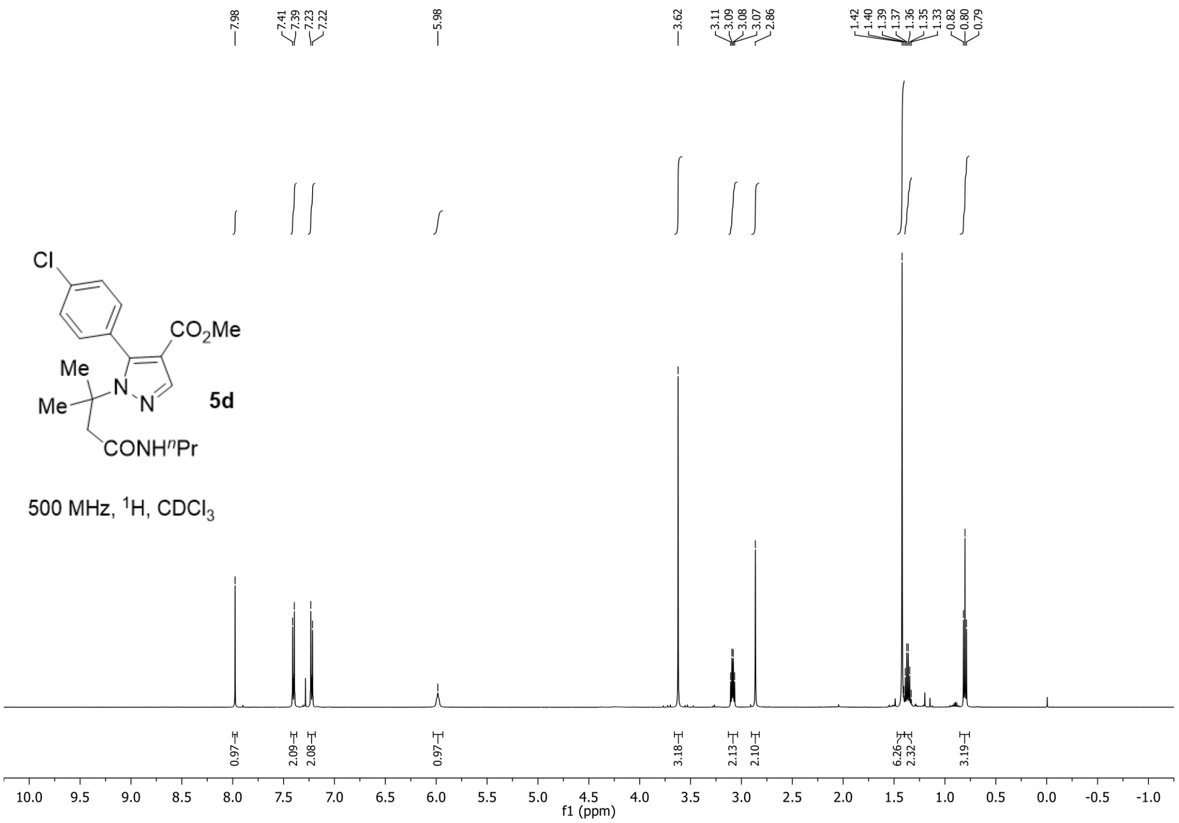
7.98
7.41
7.39
7.23
7.22
5.98
3.62
3.11
3.09
3.08
3.07
2.86
1.42
1.40
1.39
1.37
1.36
1.35
1.33
0.82
0.80
0.79
Cl
CO2Me
Me
Me
N
N
5d
CONHnPr
500 MHz, 1H, CDCl3
0.97
2.09
2.08
0.97
3.18
2.13
2.10
6.26
2.32
3.19
f1 (ppm)

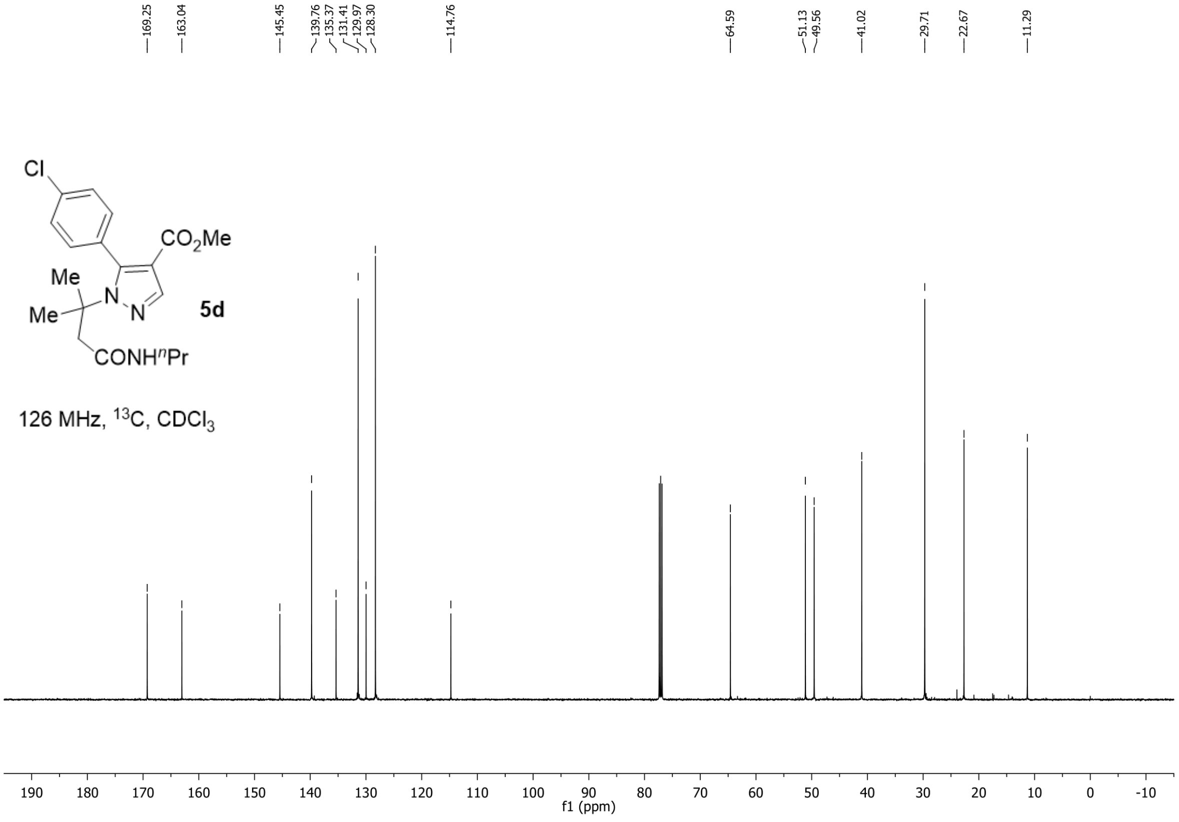
169.25
163.04
145.45
139.76
135.37
131.41
129.97
128.30
114.76
64.59
51.13
49.56
41.02
29.71
22.67
11.29
Cl
CO2Me
Me
Me
N
N
5d
CONHnPr
126 MHz, 13C, CDCl3
f1 (ppm)

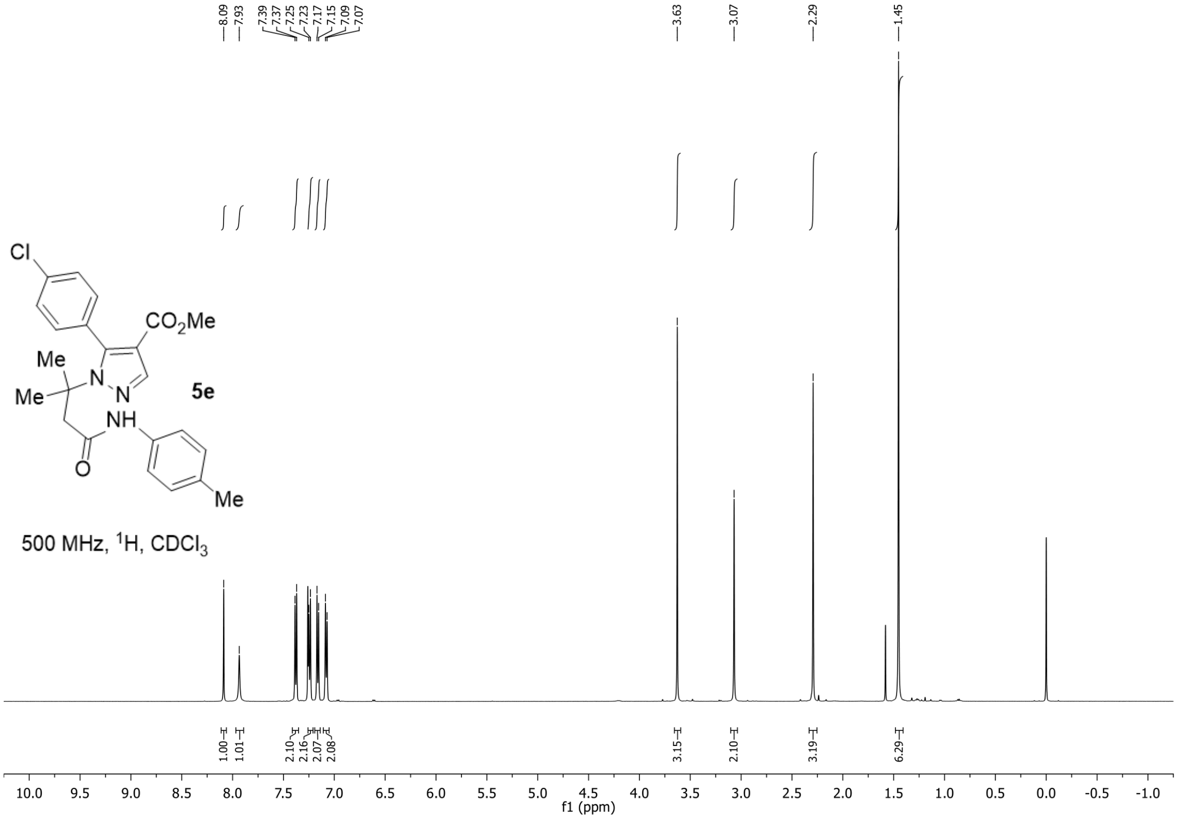
8.09
7.93
7.39
7.37
7.25
7.23
7.17
7.15
7.09
7.07
3.63
3.07
2.29
1.45
Cl
CO_2Me
Me
Me
N
N
NH
O
Me
5e
500 MHz, ¹H, $CDCl_3$
1.00
1.01
2.10
2.16
2.07
2.08
3.15
2.10
3.19
6.29
f1 (ppm)

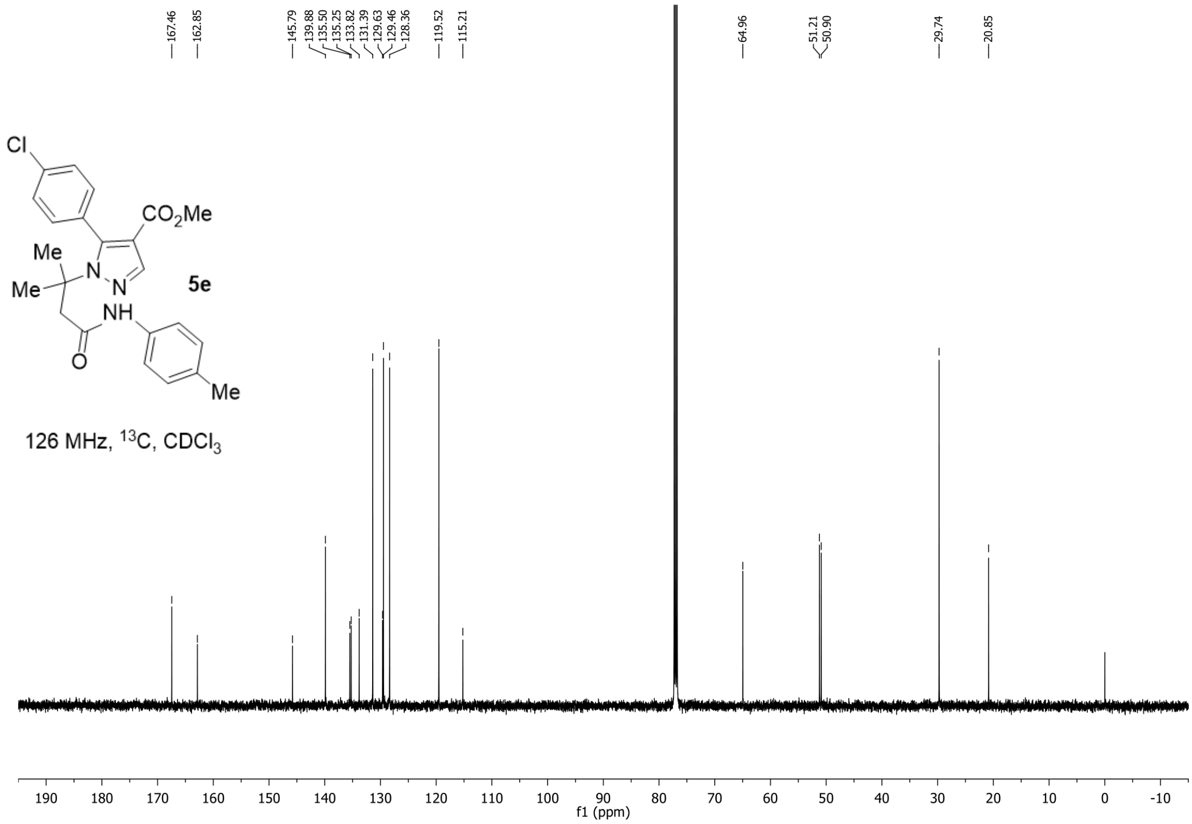
167.46
162.85
145.79
139.88
135.50
135.25
133.82
131.39
129.63
129.46
128.36
119.52
115.21
64.96
51.21
50.90
29.74
20.85
Cl
CO_2Me
Me
Me
N
N
NH
O
Me
5e
126 MHz, ¹³C, $CDCl_3$
f1 (ppm)

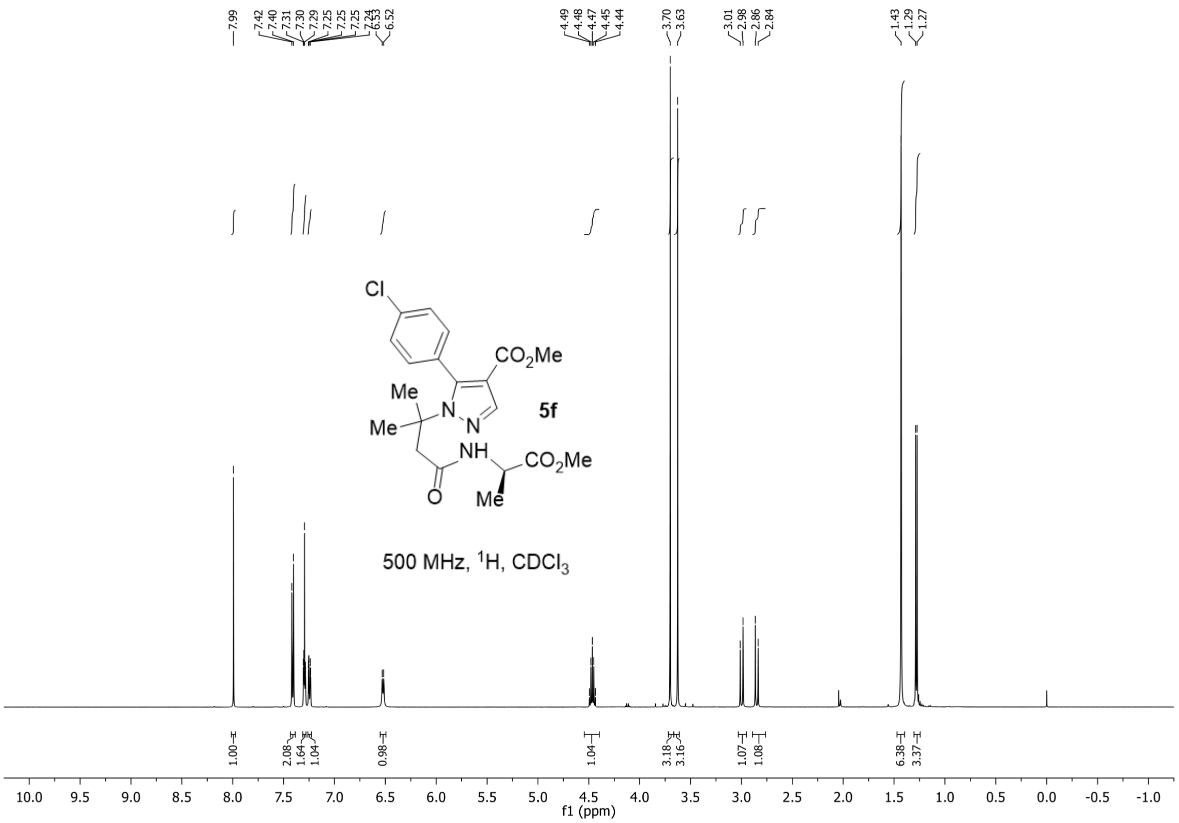

7.99 7.42 7.40 7.31 7.30 7.29 7.25 7.25 7.25 7.24 6.53 6.52
4.49 4.48 4.47 4.45 4.44
3.70 3.63
3.01 2.98 2.86 2.84
1.43 1.29 1.27
Cl
CO2Me
Me
Me
N
N
NH
CO2Me
O
Me
5f
500 MHz, 1H, CDCl3
1.00 2.08 1.64 1.04 0.98 1.04 3.18 3.16 1.07 1.08 6.38 3.37
f1 (ppm)

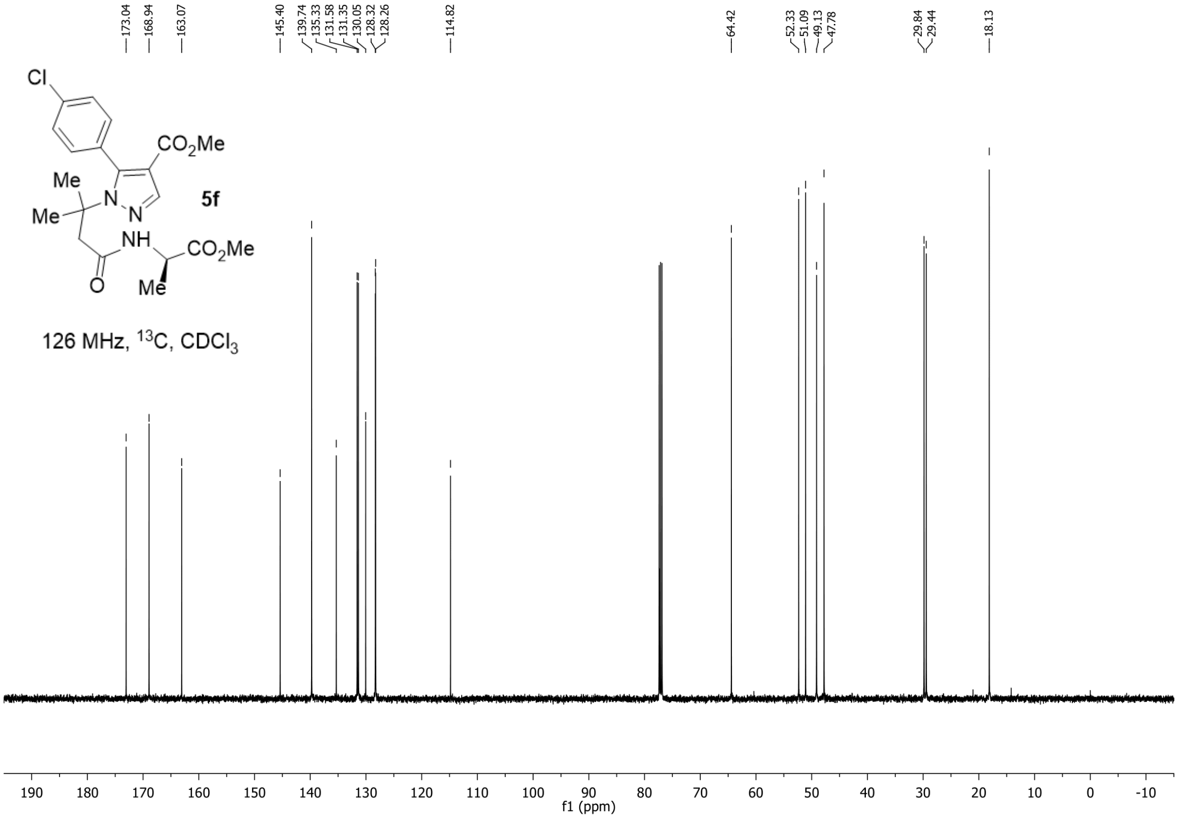

173.04 168.94 163.07 145.40 139.74 135.33 131.58 131.35 130.05 128.32 128.26 114.82 64.42 52.33 51.09 49.13 47.78 29.84 29.44 18.13
Cl
CO2Me
Me
Me
N
N
NH
CO2Me
O
Me
5f
126 MHz, 13C, CDCl3
f1 (ppm)

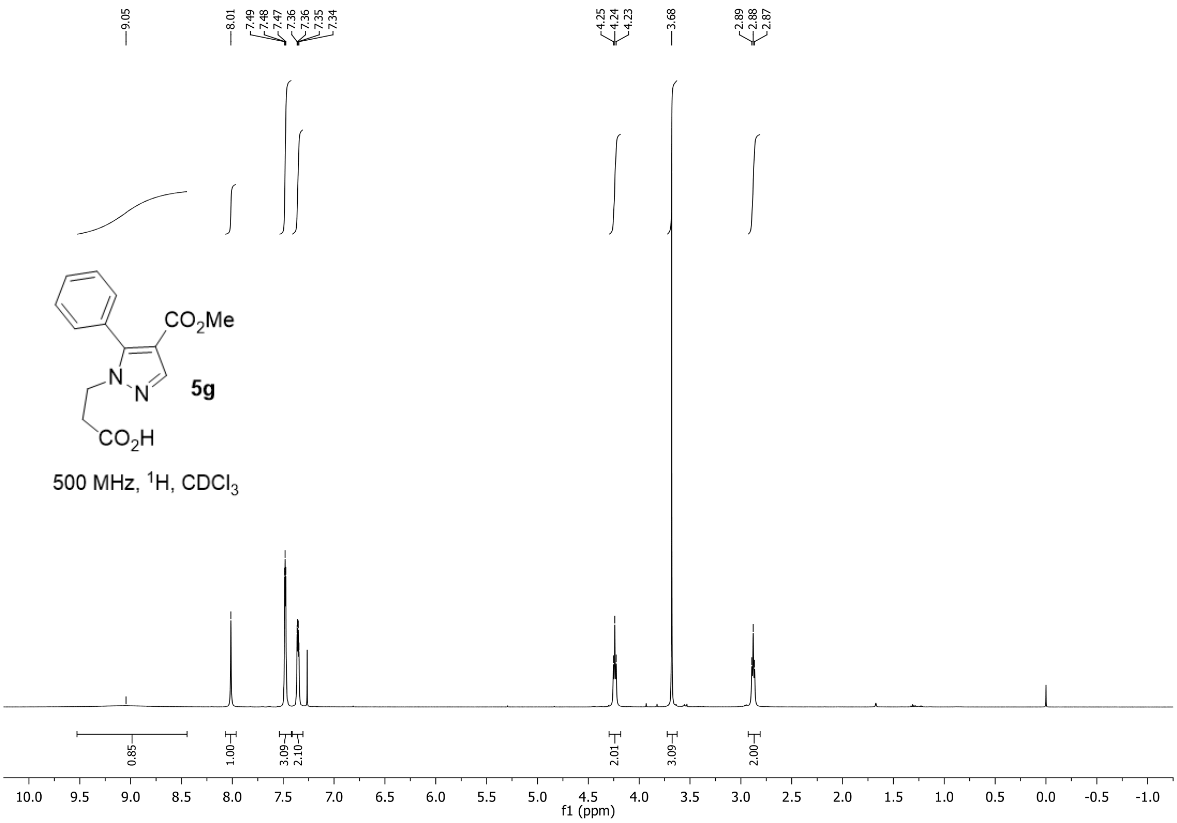
CO2Me
N
N
5g
CO2H
500 MHz, 1H, CDCl3
f1 (ppm)

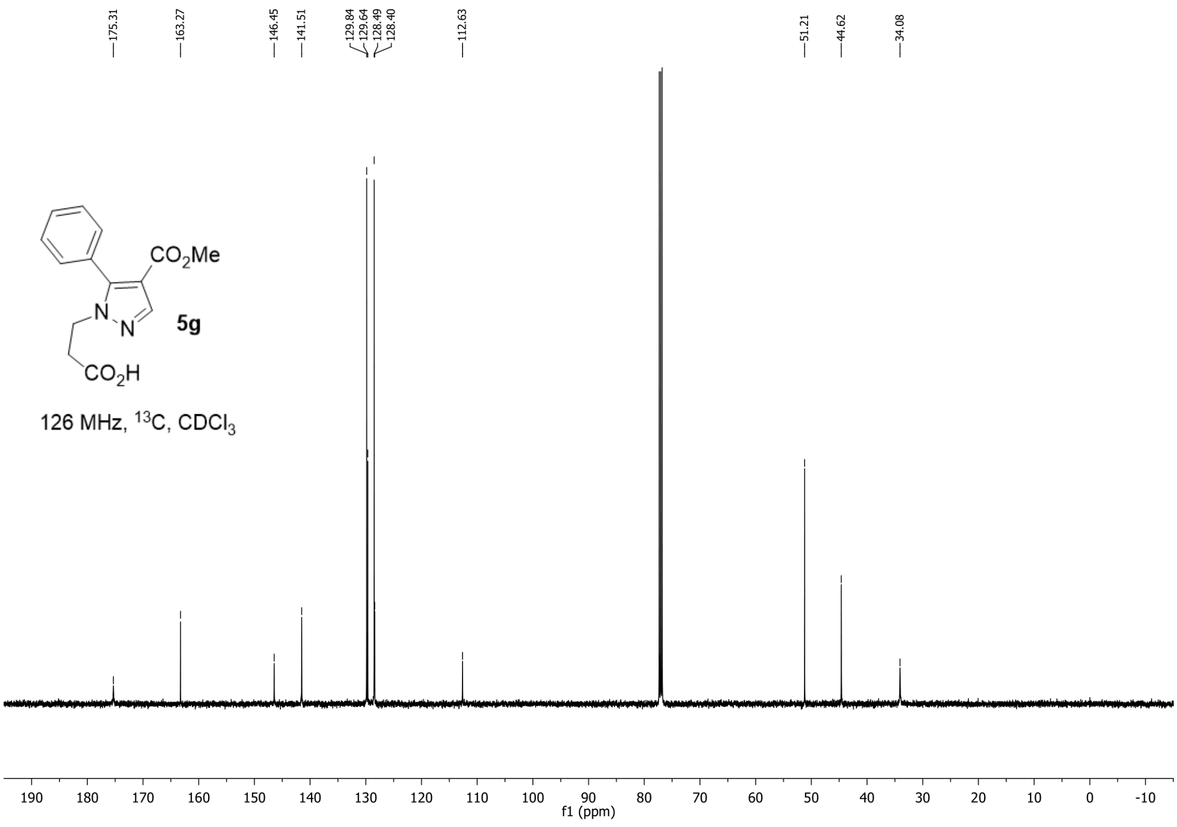
CO2Me
N
N
5g
CO2H
126 MHz, 13C, CDCl3
f1 (ppm)

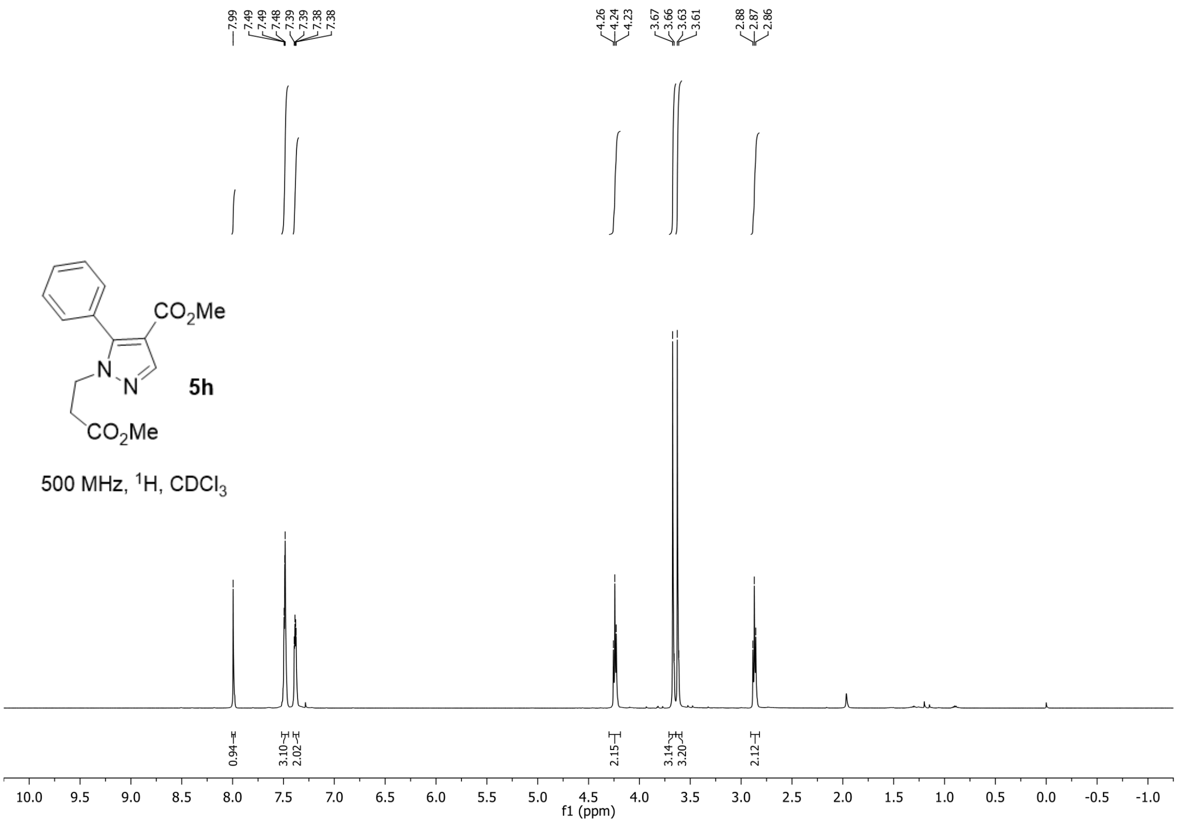
5h
500 MHz, ¹H, CDCl₃
f1 (ppm)

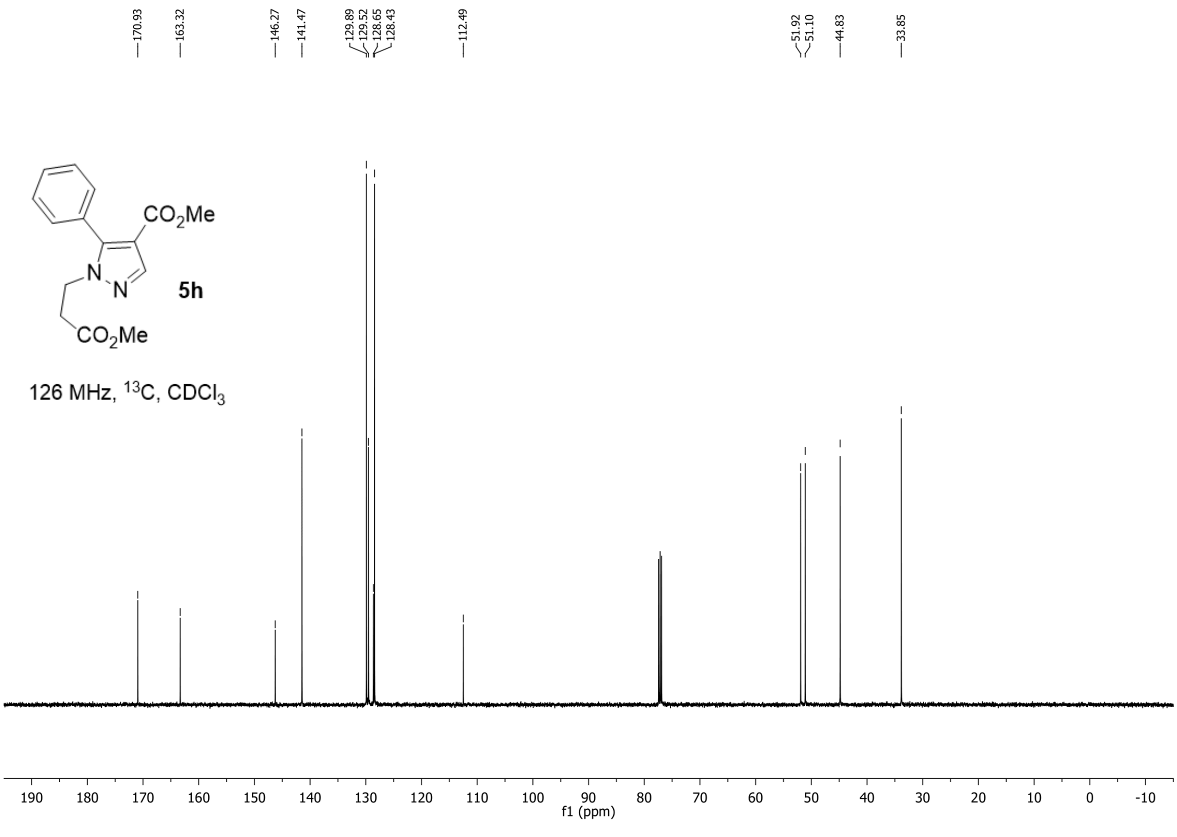
5h
126 MHz, ¹³C, CDCl₃
f1 (ppm)

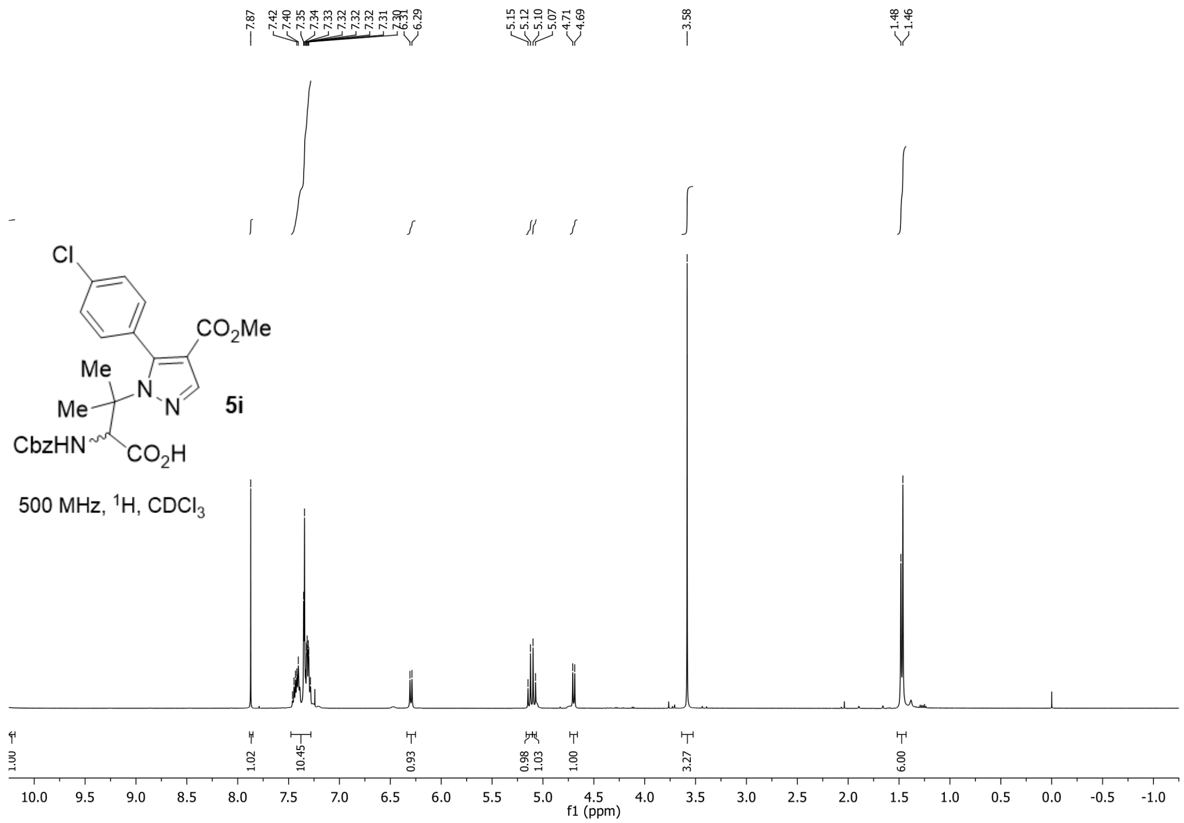
5i
500 MHz, ^{1}H, $CDCl_3$
f1 (ppm)

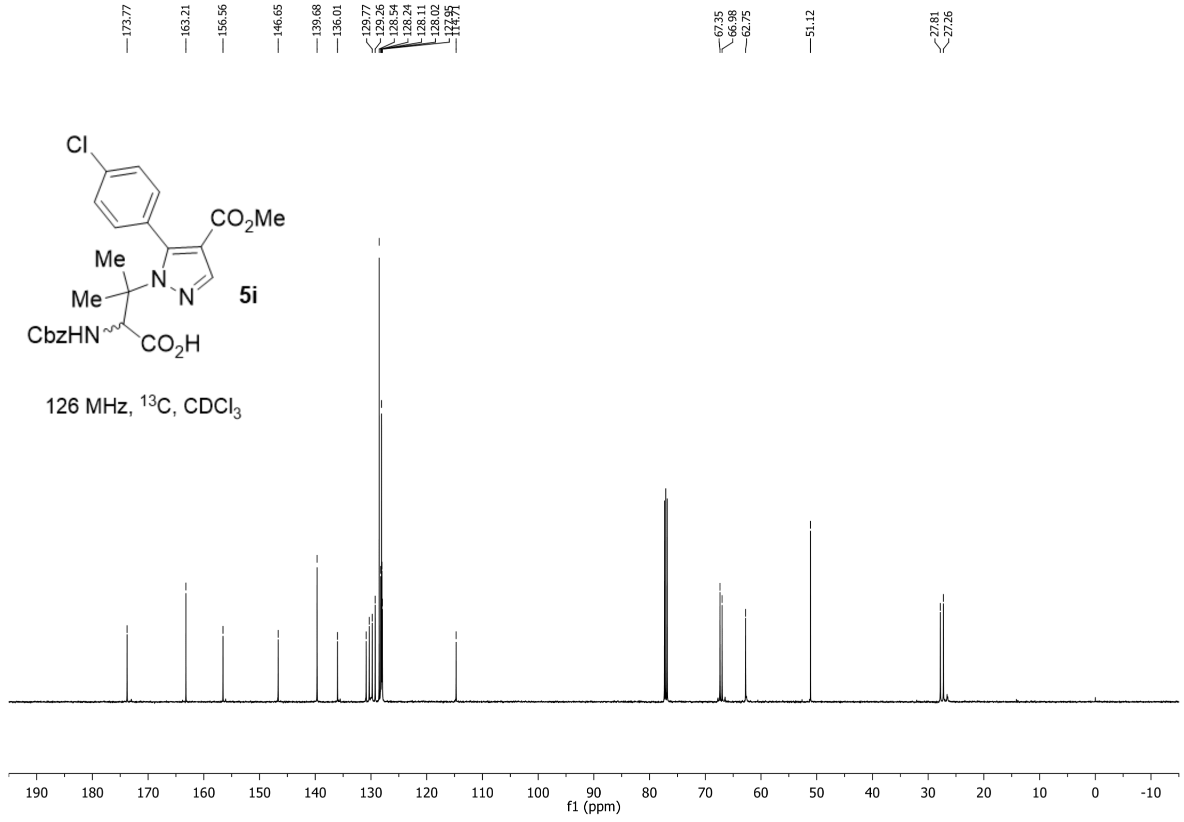
5i
126 MHz, ^{13}C, $CDCl_3$
f1 (ppm)

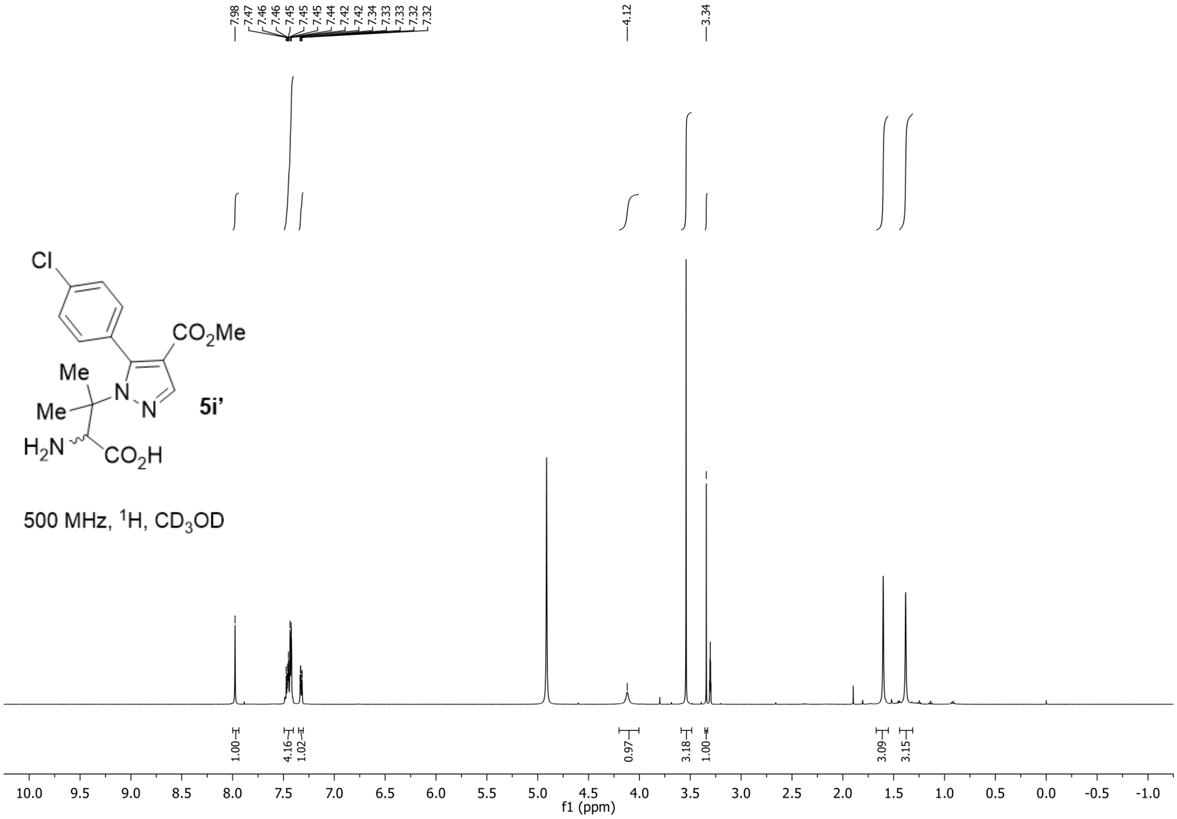
7.98
7.47
7.46
7.46
7.45
7.45
7.45
7.45
7.44
7.42
7.42
7.34
7.33
7.33
7.32
7.32
4.12
3.34
Cl
CO2Me
Me
Me
N
N
5i'
H2N
CO2H
500 MHz, 1H, CD3OD
1.00
4.16
1.02
0.97
3.18
1.00
3.09
3.15
f1 (ppm)

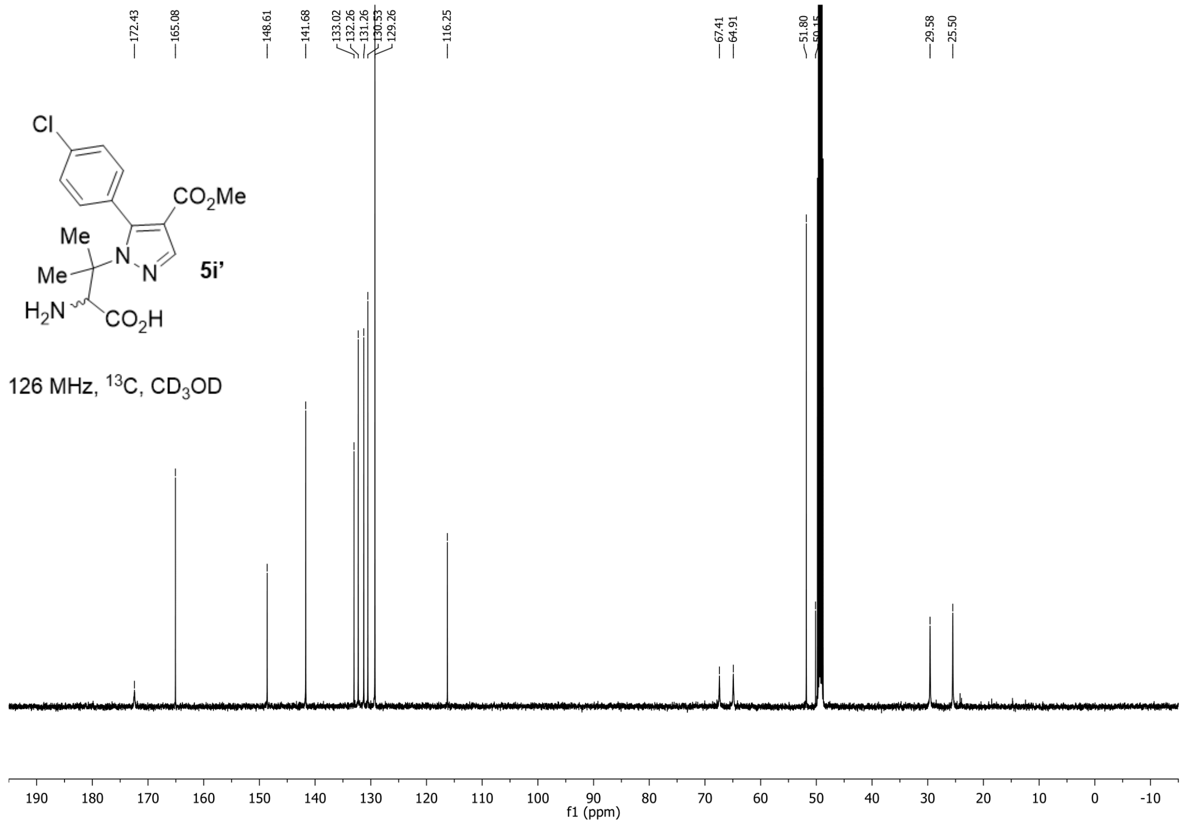
172.43
165.08
148.61
141.68
133.02
132.26
131.26
130.53
129.26
116.25
67.41
64.91
51.80
29.58
25.50
Cl
CO2Me
Me
Me
N
N
5i'
H2N
CO2H
126 MHz, 13C, CD3OD
f1 (ppm)

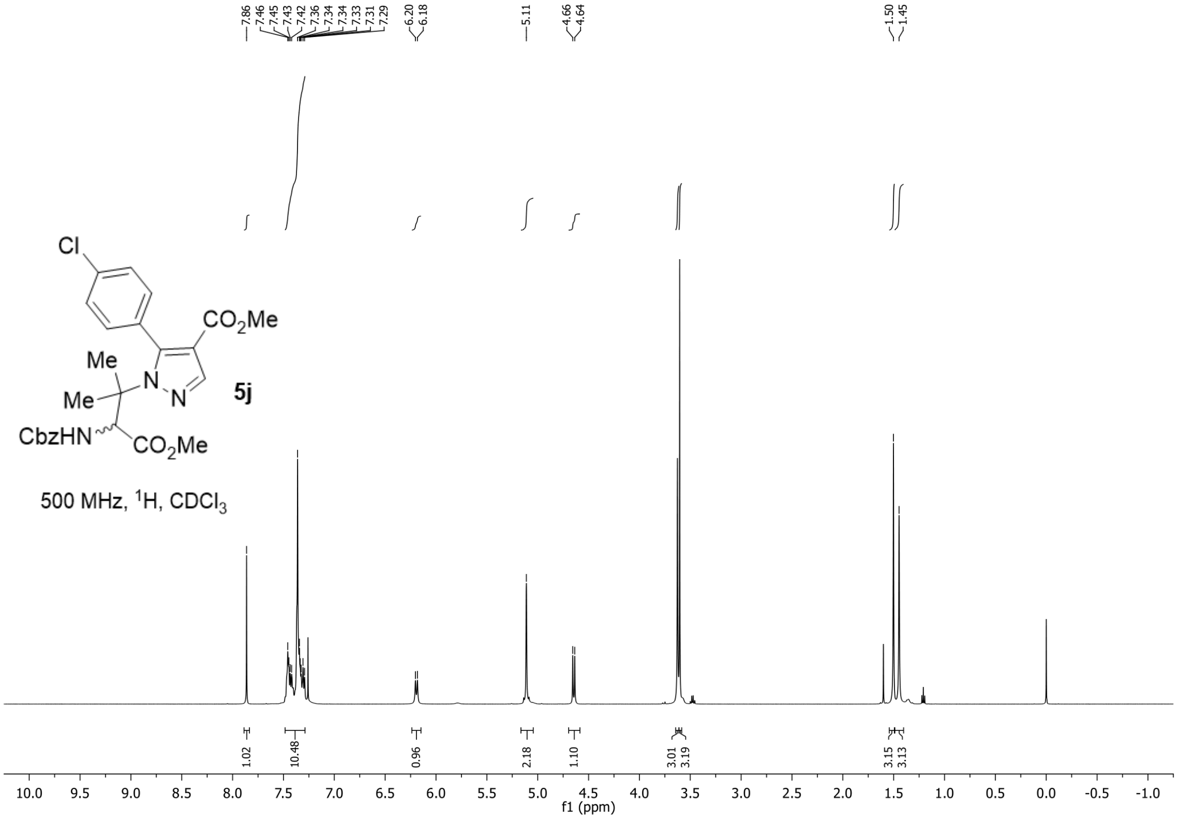

7.86 7.46 7.45 7.43 7.42 7.36 7.34 7.34 7.33 7.31 7.29
6.20 6.18
5.11
4.66 4.64
1.50 1.45
Cl
CO2Me
Me
Me
N
N
CbzHN
CO2Me
5j
500 MHz, 1H, CDCl3
1.02
10.48
0.96
2.18
1.10
3.01
3.19
3.15
3.13
10.0 9.5 9.0 8.5 8.0 7.5 7.0 6.5 6.0 5.5 5.0 4.5 4.0 3.5 3.0 2.5 2.0 1.5 1.0 0.5 0.0 -0.5 -1.0
f1 (ppm)

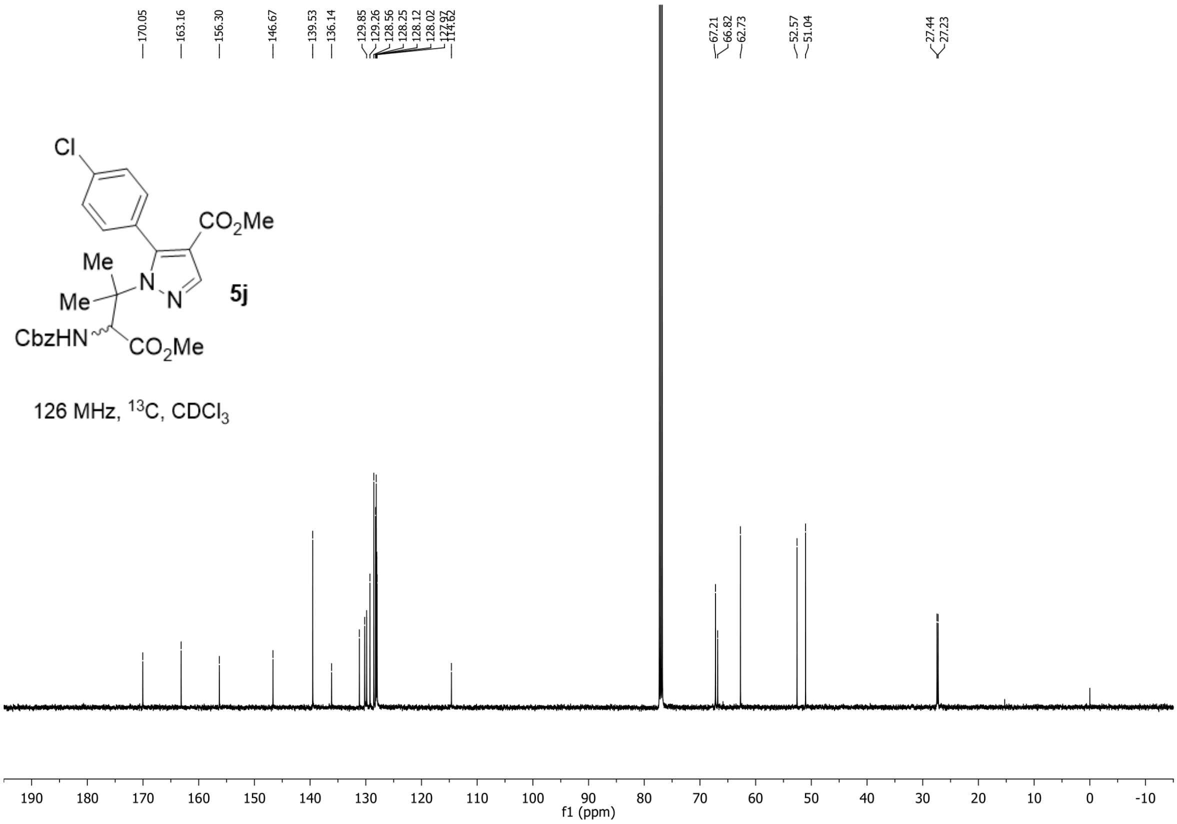

170.05
163.16
156.30
146.67
139.53
136.14
129.85 129.26 128.56 128.25 128.12 128.02 127.97 114.62
67.21 66.82 62.73
52.57 51.04
27.44 27.23
Cl
CO2Me
Me
Me
N
N
CbzHN
CO2Me
5j
126 MHz, 13C, CDCl3
190 180 170 160 150 140 130 120 110 100 90 80 70 60 50 40 30 20 10 0 -10
f1 (ppm)

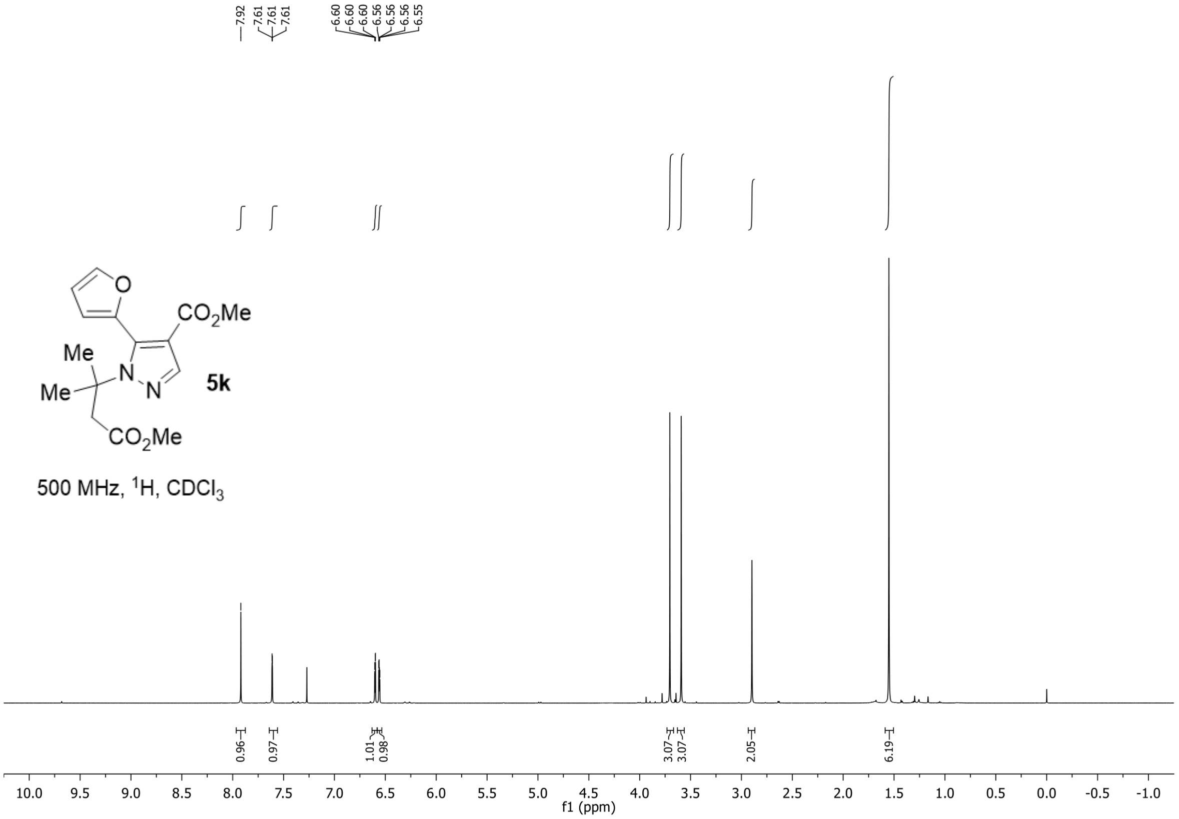

7.92
7.61
7.61
7.61
6.60
6.60
6.60
6.56
6.56
6.56
6.55
CO2Me
Me
Me
N
N
O
CO2Me
5k
500 MHz, 1H, CDCl3
0.96
0.97
1.01
0.98
3.07
3.07
2.05
6.19
10.0 9.5 9.0 8.5 8.0 7.5 7.0 6.5 6.0 5.5 5.0 4.5 4.0 3.5 3.0 2.5 2.0 1.5 1.0 0.5 0.0 -0.5 -1.0
f1 (ppm)

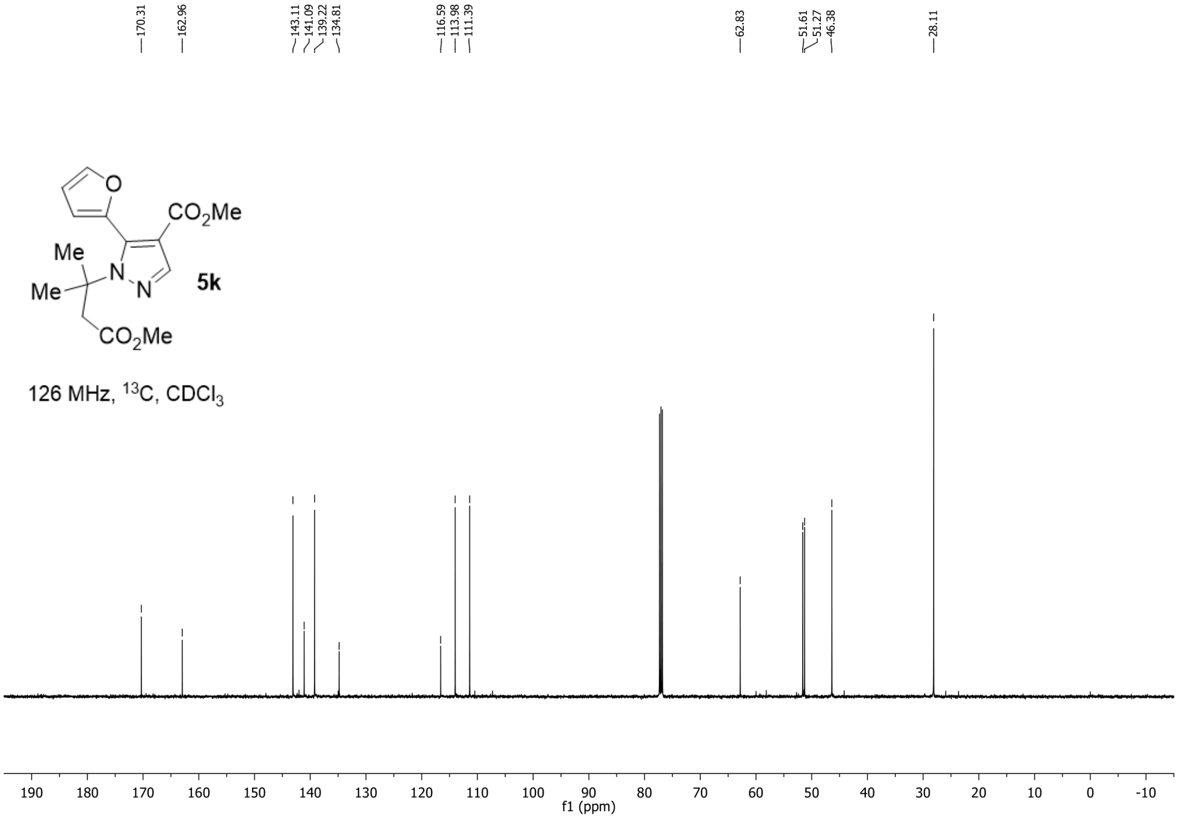

170.31
162.96
143.11
141.09
139.22
134.81
116.59
113.98
111.39
62.83
51.61
51.27
46.38
28.11
CO2Me
Me
Me
N
N
O
CO2Me
5k
126 MHz, 13C, CDCl3
190 180 170 160 150 140 130 120 110 100 90 80 70 60 50 40 30 20 10 0 -10
f1 (ppm)

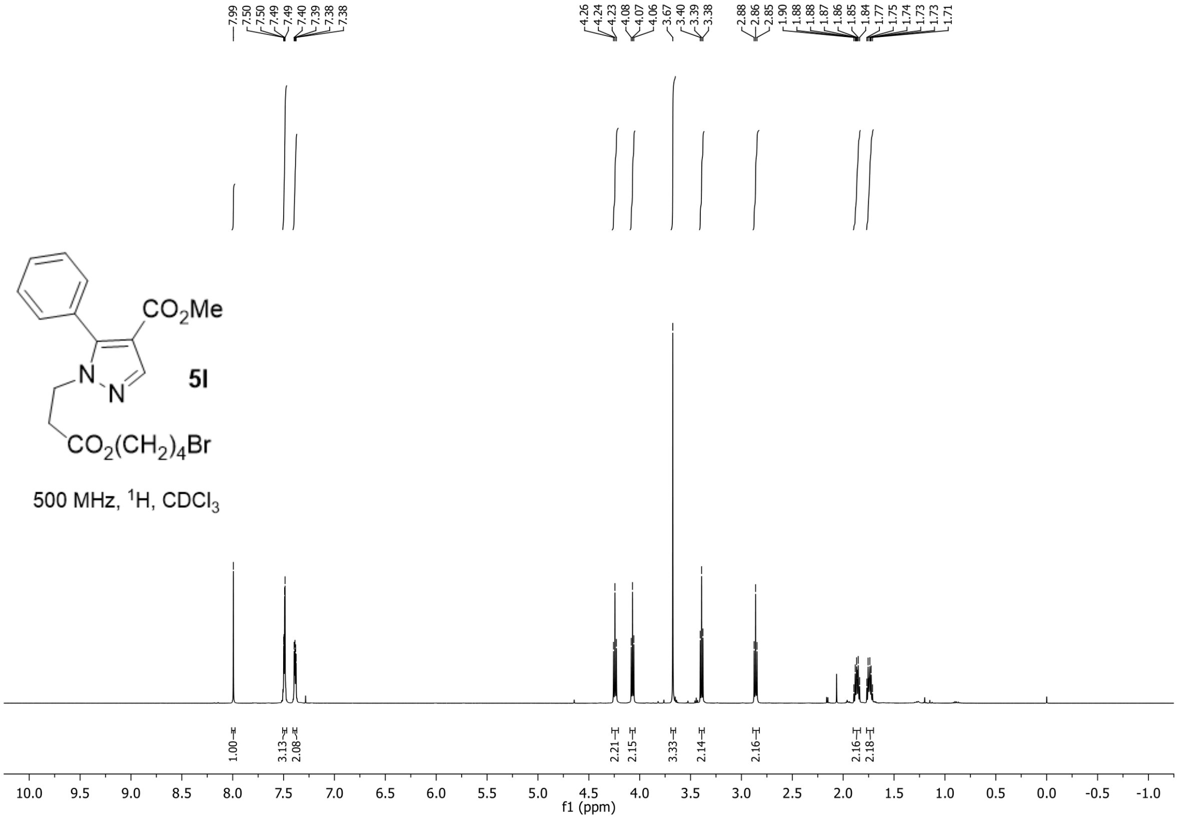

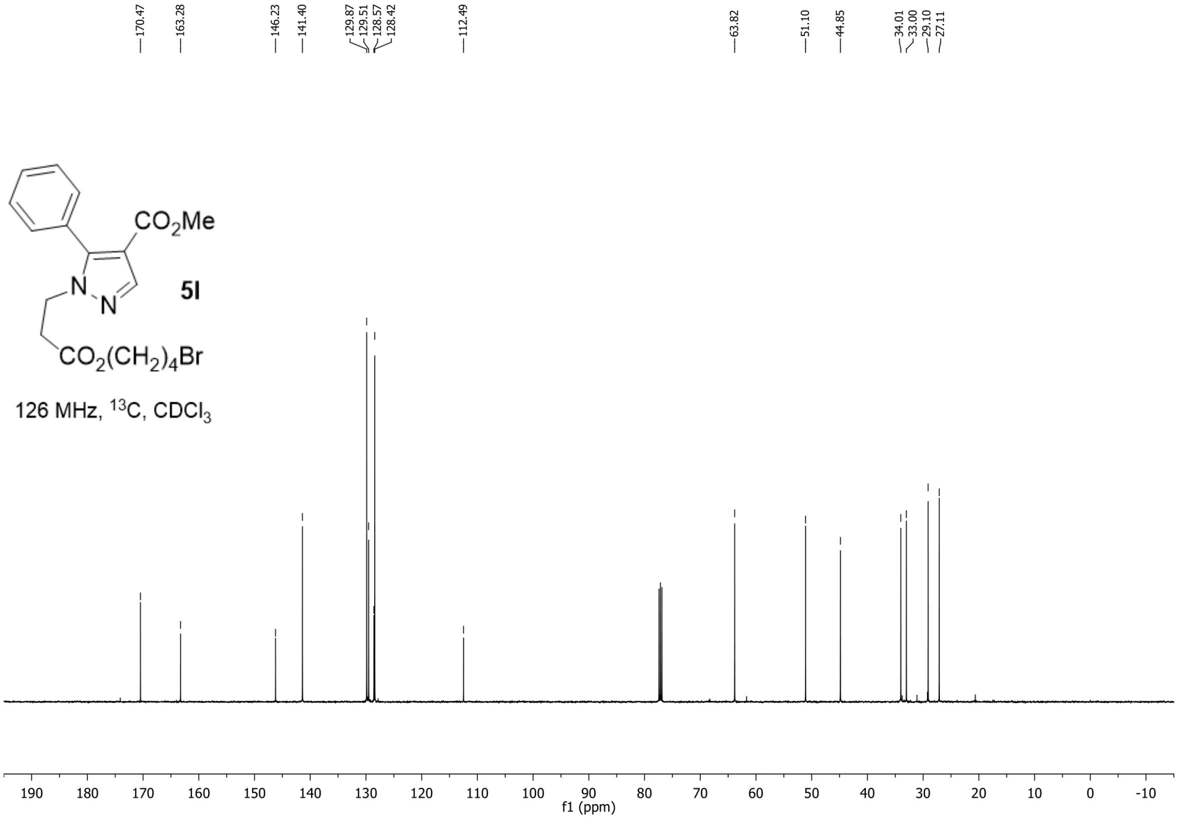

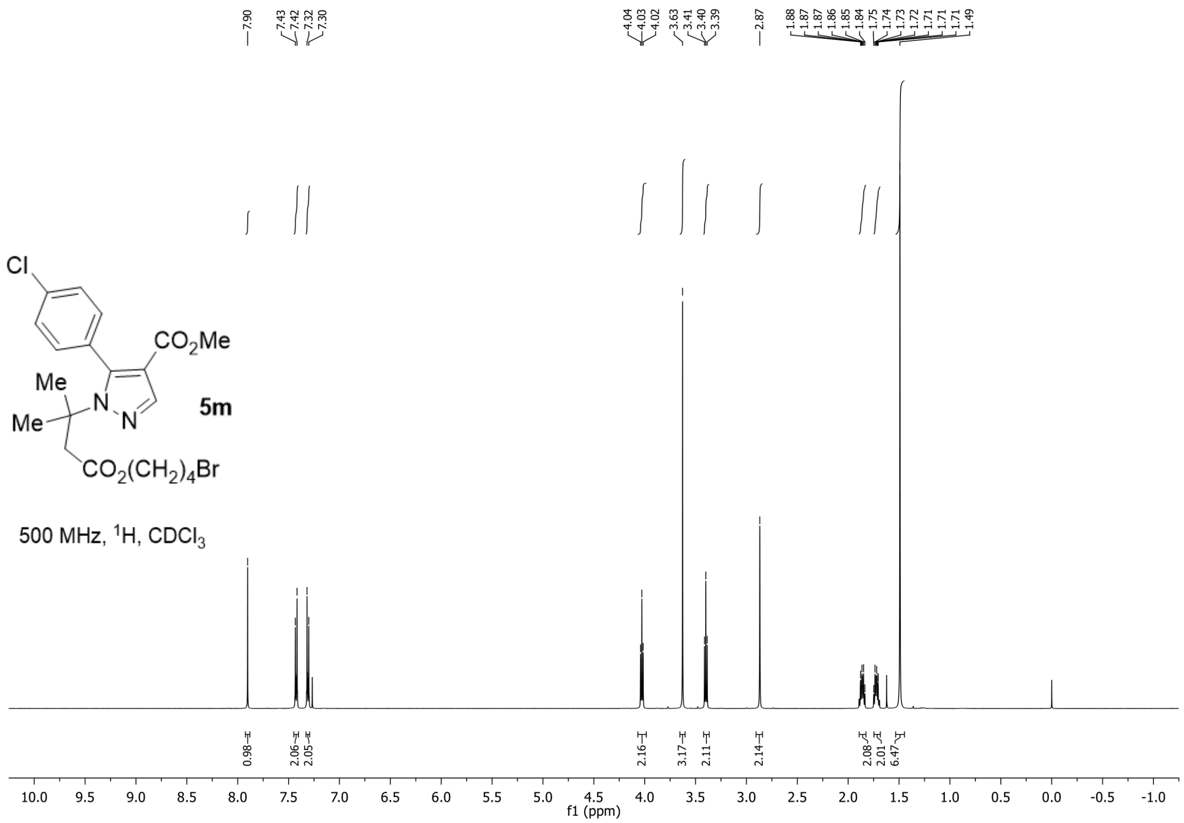
7.90
7.43 7.42 7.32 7.30
4.04 4.03 4.02 3.63 3.41 3.40 3.39
2.87
1.88 1.87 1.87 1.86 1.85 1.84 1.75 1.74 1.73 1.72 1.71 1.71 1.71 1.49
Cl
CO_2Me
Me
Me
N
N
5m
$CO_2(CH_2)_4Br$
500 MHz, ^1H, $CDCl_3$
0.98 2.06 2.05 2.16 3.17 2.11 2.14 2.08 2.01 6.47
f1 (ppm)

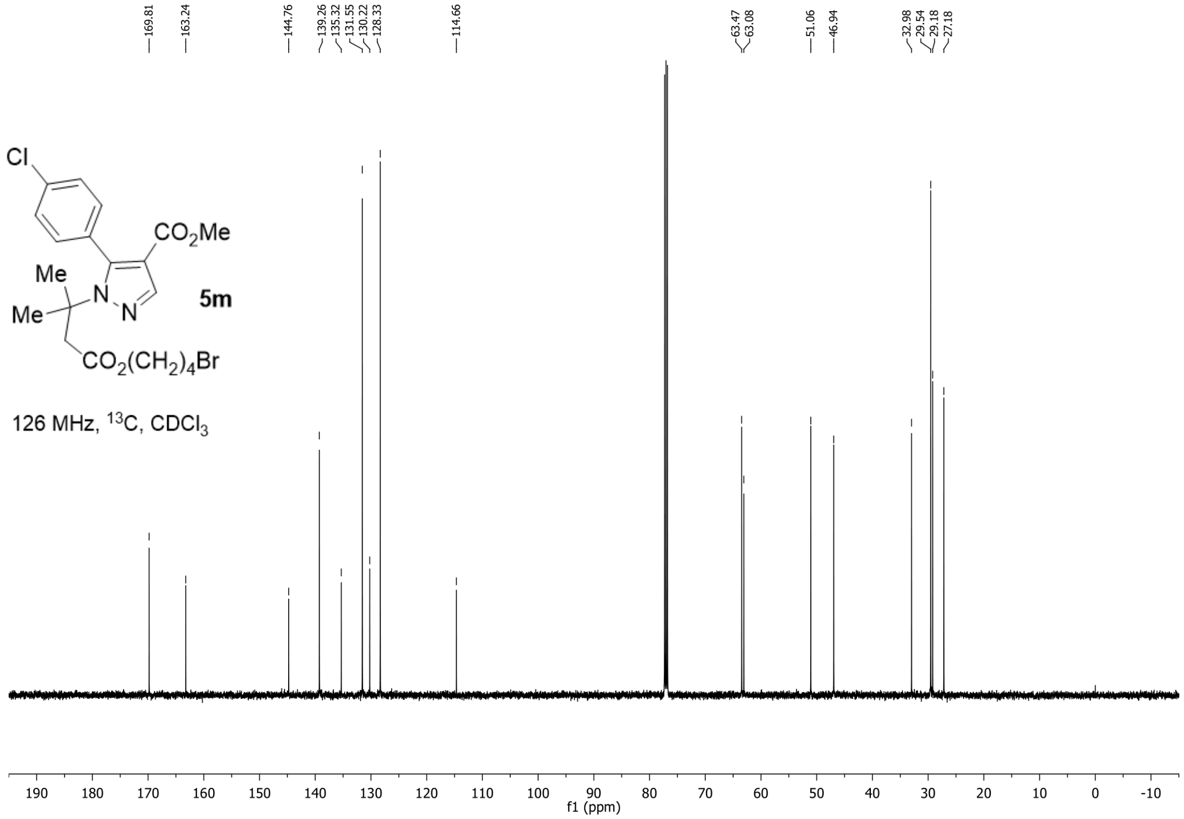
169.81
163.24
144.76
139.26 135.32 131.55 130.22 128.33
114.66
63.47 63.08
51.06
46.94
32.98 29.54 29.18 27.18
Cl
CO_2Me
Me
Me
N
N
5m
$CO_2(CH_2)_4Br$
126 MHz, ^{13}C, $CDCl_3$
f1 (ppm)

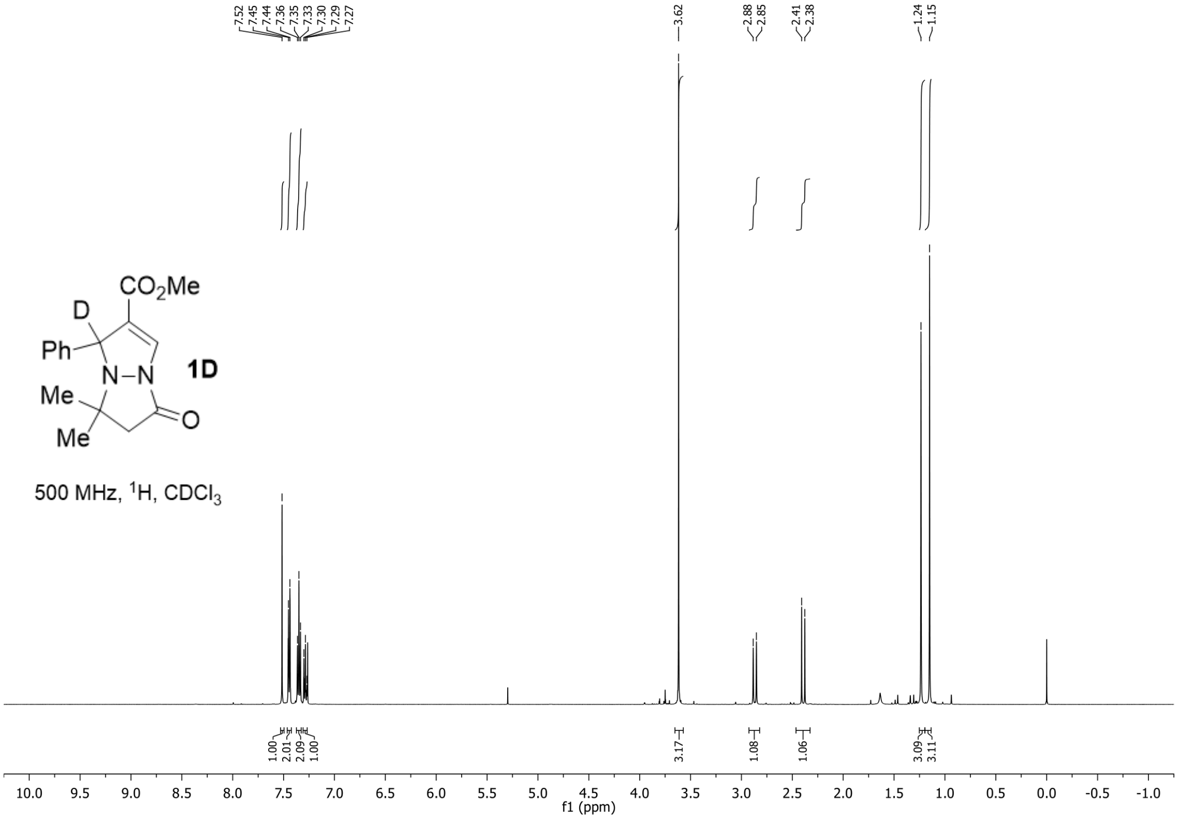
7.52
7.45
7.44
7.36
7.35
7.33
7.30
7.29
7.27
3.62
2.88
2.85
2.41
2.38
1.24
1.15
CO2Me
D
Ph
N–N
1D
Me
Me
O
500 MHz, 1H, CDCl3
1.00
2.01
2.09
1.00
3.17
1.08
1.06
3.09
3.11
10.0 9.5 9.0 8.5 8.0 7.5 7.0 6.5 6.0 5.5 5.0 4.5 4.0 3.5 3.0 2.5 2.0 1.5 1.0 0.5 0.0 -0.5 -1.0
f1 (ppm)

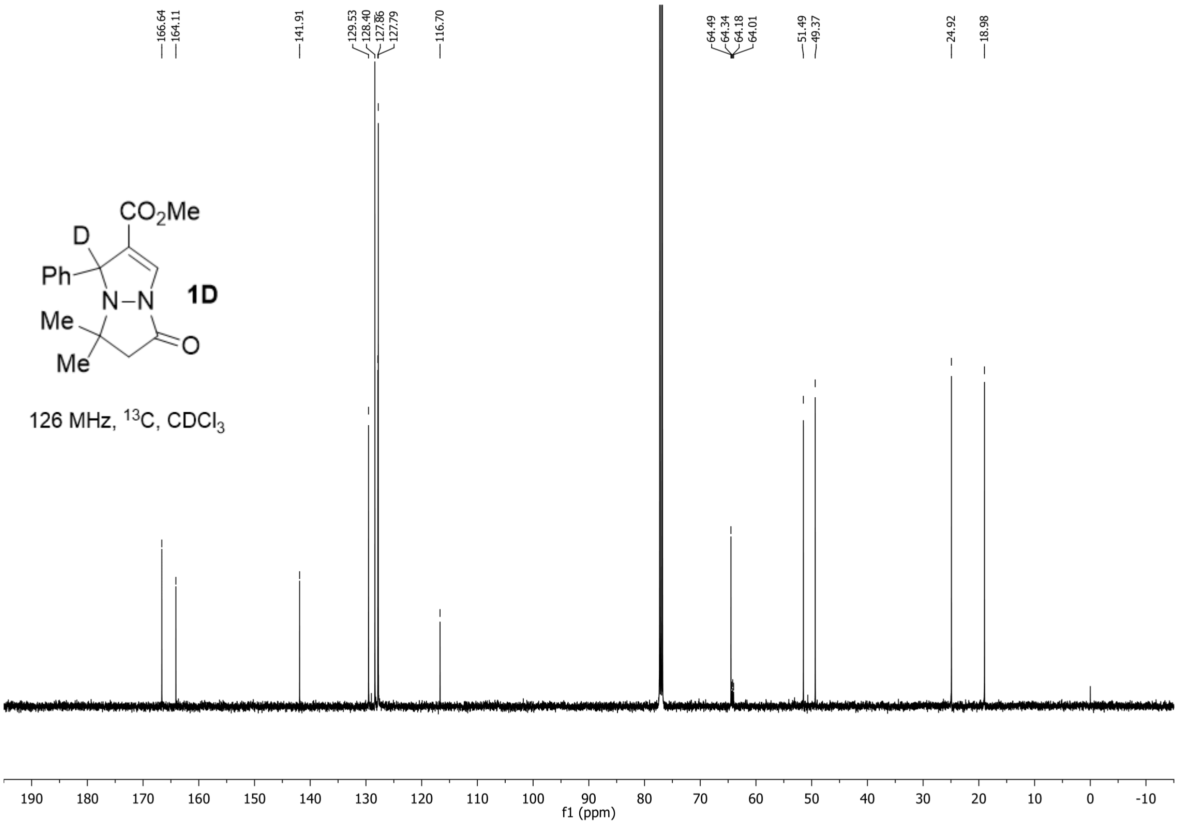
166.64
164.11
141.91
129.53
128.40
127.86
127.79
116.70
64.49
64.34
64.18
64.01
51.49
49.37
24.92
18.98
CO2Me
D
Ph
N–N
1D
Me
Me
O
126 MHz, 13C, CDCl3
190 180 170 160 150 140 130 120 110 100 90 80 70 60 50 40 30 20 10 0 -10
f1 (ppm)

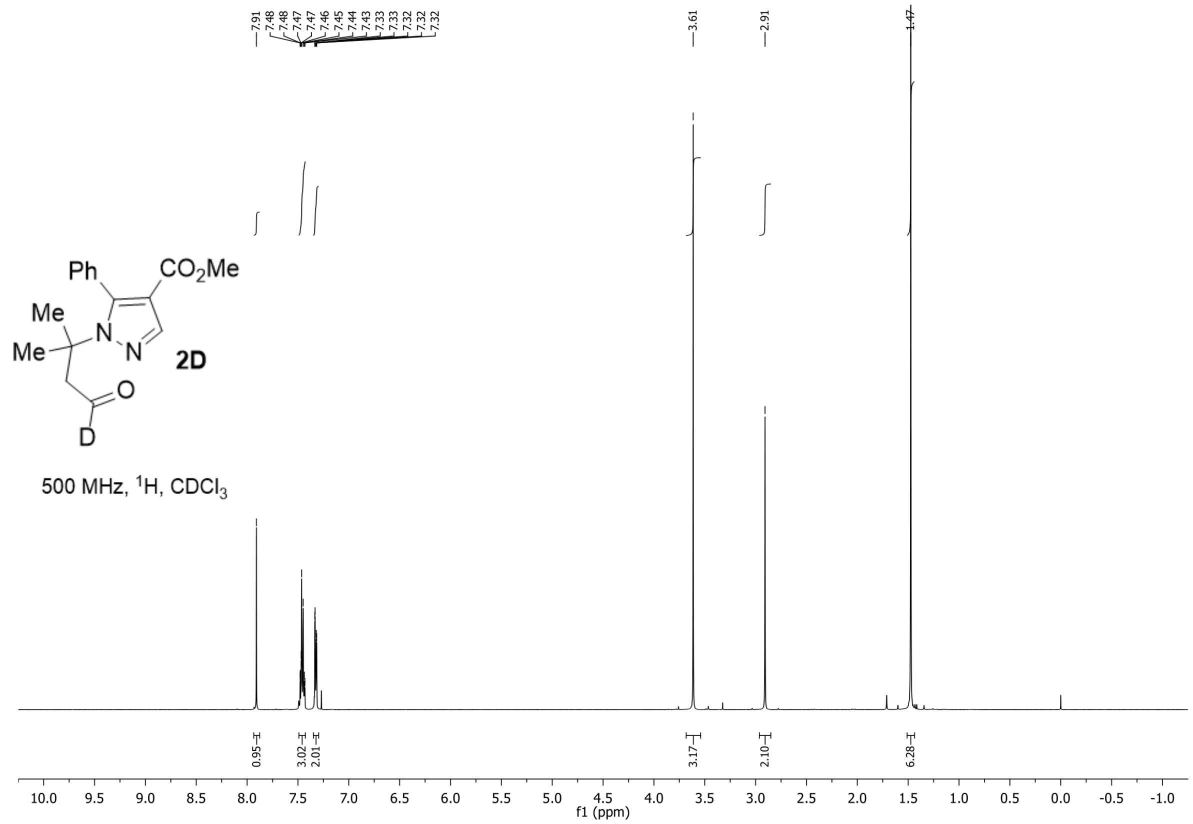
7.91
7.48
7.48
7.47
7.47
7.46
7.45
7.44
7.43
7.33
7.33
7.32
7.32
7.32
3.61
2.91
1.47
Ph
CO2Me
Me
Me
N
N
2D
O
D
500 MHz, 1H, CDCl3
0.95
3.02
2.01
3.17
2.10
6.28
f1 (ppm)

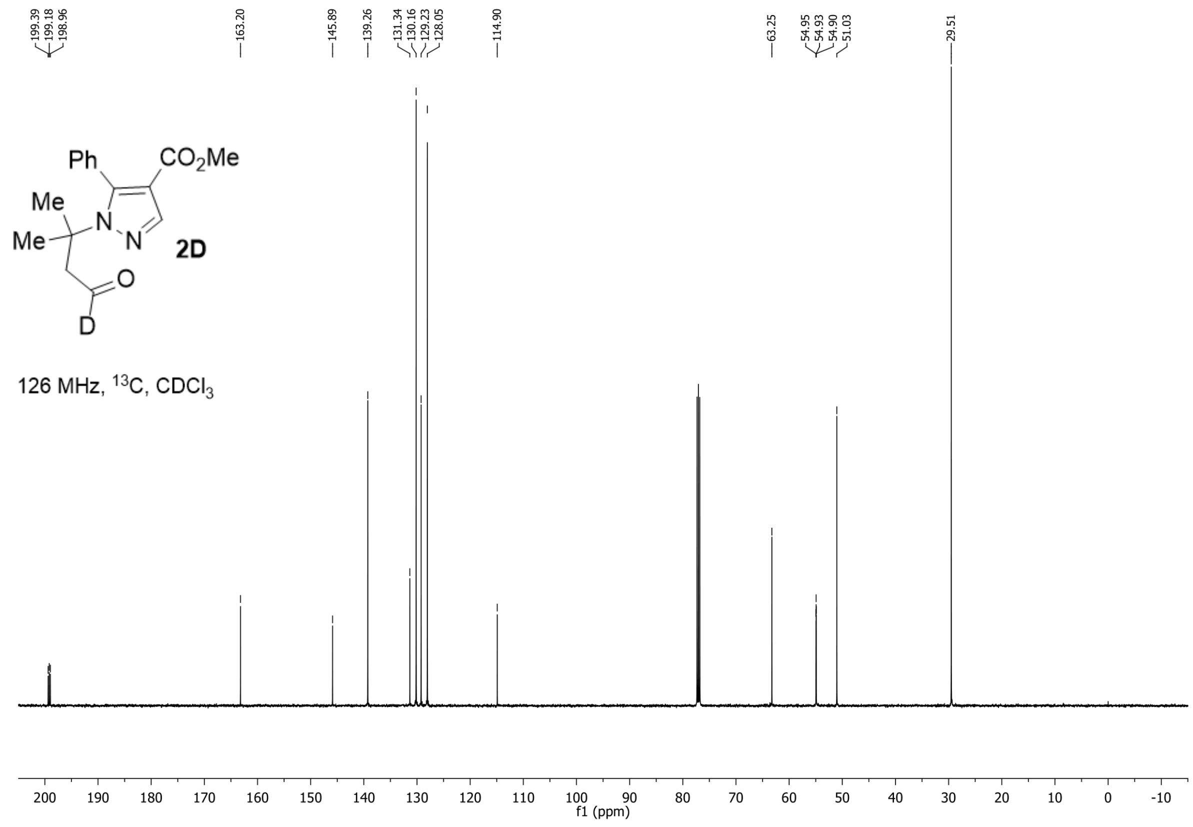
199.39
199.18
198.96
163.20
145.89
139.26
131.34
130.16
129.23
128.05
114.90
63.25
54.95
54.93
54.90
51.03
29.51
Ph
CO2Me
Me
Me
N
N
2D
O
D
126 MHz, 13C, CDCl3
f1 (ppm)

18. References

1. Sibi, M. P.; Rane, D.; Stanley, L. M.; Soeta, T., Copper(II)-Catalyzed Exo and Enantioselective Cycloadditions of Azomethine Imines. *Org. Lett.* **2008,** *10* (14), 2971-2974.
2. Schulz, M.; West, G., Photochemische Reaktionen von Pyrazolidon-(3)-betainen. II. Synthese der β-Hydrazino-isovaleriansäure. *J. Prakt. Chem.* **1973,** *315* (4), 711-716.
3. Petek, N.; Grošelj, U.; Svete, J.; Požgan, F.; Kočar, D.; Štefane, B., Eosin Y-Catalyzed Visible-Light-Mediated Aerobic Transformation of Pyrazolidine-3-One Derivatives. *Catalysts* **2020,** *10* (9), 981.
4. Koptelov, Y. B.; Sednev, M. V.; Kostikov, R. R., (Z)-1-alkylidene- and 1-arylmethylidene-5,5-dimethyl-3-oxopyrazolidin-1-ium-2-ides and their cycloaddition to N-arylmaleimides. *Russ. J. Org. Chem.* **2012,** *48* (6), 804-814.
5. Taylor, E. C.; Haley, N. F.; Clemens, R. J., Synthesis and properties of 3-oxo-1,2-diazetidinium ylides. *J. Am. Chem. Soc.* **1981,** *103* (26), 7743-7752.
6. Kawai, H.; Kusuda, A.; Nakamura, S.; Shiro, M.; Shibata, N., Catalytic Enantioselective Trifluoromethylation of Azomethine Imines with Trimethyl(trifluoromethyl)silane. *Angew. Chem. Int. Ed.* **2009,** *48* (34), 6324-6327.
7. Turk, C.; Svete, J.; Stanovnik, B.; Golič, L.; Golič-Grdadolnik, S.; Golobič, A.; Selič, L., Regioselective 1,3-Dipolar Cycloadditions of (1Z)-1-(Arylmethylidene)-5,5-dimethyl-3-oxopyrazolidin-1-ium-2-ide Azomethine Imines to Acetylenic Dipolarophiles. *Helv. Chim. Acta* **2001,** *84* (1), 146-156.
8. Novak, A.; Štefanič, M.; Grošelj, U.; Hrast, M.; Kasunič, M.; Gobec, S.; Stanovnik, B.; Svete, J., A Simple Synthesis of Polyfunctionalized 4-Aminopyrazolidin-3-ones as 'Aza-deoxa' Analogs of D-Cycloserine. *Helv. Chim. Acta* **2014,** *97* (2), 245-267.
9. Pušavec Kirar, E.; Grošelj, U.; Mirri, G.; Požgan, F.; Strle, G.; Štefane, B.; Jovanovski, V.; Svete, J., "Click" Chemistry: Application of Copper Metal in Cu-Catalyzed Azomethine Imine–Alkyne Cycloadditions. *J. Org. Chem.* **2016,** *81* (14), 5988-5997.
10. Takamichi, O.; Kazuaki, Y.; Kazuya, Y.; Noritaka, M., An Efficient Copper-mediated 1,3-Dipolar Cycloaddition of Pyrazolidinone-based Dipoles to Terminal Alkynes to Produce N,N-Bicyclic Pyrazolidinone Derivatives. *Chem. Lett.* **2010,** *39* (10), 1086-1087.
11. Pušavec Kirar, E.; Grošelj, U.; Golobič, A.; Požgan, F.; Pusch, S.; Weber, C.; Andernach, L.; Štefane, B.; Opatz, T.; Svete, J., Absolute Configuration Determination of 2,3-Dihydro-1H,5H-pyrazolo[1,2-a]pyrazoles Using Chiroptical Methods at Different Wavelengths. *J. Org. Chem.* **2016,** *81* (23), 11802-11812.
12. Mirnik, J.; Pušavec Kirar, E.; Ričko, S.; Grošelj, U.; Golobič, A.; Požgan, F.; Štefane, B.; Svete, J., Cu0-catalysed 1,3-dipolar cycloadditions of α-amino acid derived N,N-cyclic azomethine imines to ynones. *Tetrahedron* **2017,** *73* (24), 3329-3337.
13. Pušavec, E.; Mirnik, J.; Šenica, L.; Grošelj, U.; Stanovnik, B.; Svete, J., Cu(I)-catalyzed [3+2] Cycloadditions of tert-Butyl (S)-(3-Oxopent-4- yn-2-yl)carbamate to 1-Benzylidenepyrazole-3-one-derived Azomethine Imines. *Z. Naturforsch. B* **2014,** *69* (5), 615-626.
14. Fan, Z.; Yi, Y.; Chen, S.; Xi, C., Visible-Light-Induced Catalyst-Free Carboxylation of Acylsilanes with Carbon Dioxide. *Org. Lett.* **2021,** *23* (6), 2303-2307.
15. Williams, A. T. R.; Winfield, S. A.; Miller, J. N., Relative fluorescence quantum yields using a computer-controlled luminescence spectrometer. *Analyst* **1983,** *108* (1290), 1067-1071.
16. Ghosh, I.; Mukhopadhyay, A.; Koner, A. L.; Samanta, S.; Nau, W. M.; Moorthy, J. N., Excited-state properties of fluorenones: influence of substituents, solvent and macrocyclic encapsulation. *Phys. Chem. Chem. Phys.* **2014,** *16* (31), 16436-16445.
17. Aranzaes, J. R.; Daniel, M.-C.; Astruc, D., Metallocenes as references for the determination of redox potentials by cyclic voltammetry — Permethylated iron and cobalt sandwich complexes, inhibition by polyamine dendrimers, and the role of hydroxy-containing ferrocenes. *Can. J. Chem.* **2006,** *84* (2), 288-299.
18. *CrysAlisPRO, Agilent Technologies UK Ltd*, Yarnton, Oxfordshire, England, 2**011**.

19. Dolomanov, O. V.; Bourhis, L. J.; Gildea, R. J.; Howard, J. A. K.; Puschmann, H., OLEX2: a complete structure solution, refinement and analysis program. *J. Appl. Crystallogr.* **2009,** *42* (2), 339-341.
20. Sheldrick, G., A short history of SHELX. *Acta Crystallogr., Sect. A* **2008,** *64* (1), 112-122.
21. Sheldrick, G., SHELXT - Integrated space-group and crystal-structure determination. *Acta Crystallogr., Sect. A* **2015,** *71* (1), 3-8.
22. Sheldrick, G., Crystal structure refinement with SHELXL. *Acta Crystallogr., Sect. C* **2015,** *71* (1), 3-8.
23. Macrae, C. F.; Edgington, P. R.; McCabe, P.; Pidcock, E.; Shields, G. P.; Taylor, R.; Towler, M.; van de Streek, J., Mercury: visualization and analysis of crystal structures. *J. Appl. Crystallogr.* **2006,** *39* (3), 453-457.
24. Spek, A., Single-crystal structure validation with the program PLATON. *J. Appl. Crystallogr.* **2003,** *36* (1), 7-13.
